# Higher PUFA and *n*-3 PUFA, conjugated linoleic acid, *α*-tocopherol and iron, but lower iodine and selenium concentrations in organic milk: a systematic literature review and meta- and redundancy analyses

**DOI:** 10.1017/S0007114516000349

**Published:** 2016-03-28

**Authors:** Dominika Średnicka-Tober, Marcin Barański, Chris J. Seal, Roy Sanderson, Charles Benbrook, Håvard Steinshamn, Joanna Gromadzka-Ostrowska, Ewa Rembiałkowska, Krystyna Skwarło-Sońta, Mick Eyre, Giulio Cozzi, Mette Krogh Larsen, Teresa Jordon, Urs Niggli, Tomasz Sakowski, Philip C. Calder, Graham C. Burdge, Smaragda Sotiraki, Alexandros Stefanakis, Sokratis Stergiadis, Halil Yolcu, Eleni Chatzidimitriou, Gillian Butler, Gavin Stewart, Carlo Leifert

**Affiliations:** 1 Nafferton Ecological Farming Group (NEFG), School of Agriculture, Food and Rural Development, Newcastle University, Nafferton Farm, Stocksfield, Northumberland NE43 7XD, UK; 2 School of Agriculture, Food and Rural Development, Human Nutrition Research Centre, Newcastle University, Agriculture Building, Kings Road, Newcastle upon Tyne NE1 7RU, UK; 3 School of Biology, Newcastle University, Ridley Building, Newcastle upon Tyne NE1 7RU, UK; 4 Benbrook Consulting Services, 90063 Troy Road, Enterprise, OR 97828, USA; 5 Food and Agriculture Division-Grassland and Forage, Norwegian Institute of Bioeconomy Research (NIBIO), Gunnars veg 6, N-6630 Tingvoll, Norway; 6 Department of Dietetics, Faculty of Human Nutrition and Consumer Sciences, Warsaw University of Life Sciences, Nowoursynowska 159c, Warsaw 02-776, Poland; 7 Department of Functional and Organic Food and Commodities, Faculty of Human Nutrition and Consumer Sciences, Warsaw University of Life Sciences, Nowoursynowska 159c, Warsaw 02-776, Poland; 8 Department of Animal Physiology, Faculty of Biology, University of Warsaw, Miecznikowa 1, Warsaw 02-096, Poland; 9 Department of Animal Medicine, Production and Health, University of Padua, Viale dell’ Università 19, 35020 Legnaro, Italy; 10 Department of Food Science-Food Chemistry & Technology, Aarhus University, Blichers Allé 20, Building F20/8845, 8830 Tjele, Denmark; 11 Research Institute for Organic Agriculture (FiBL), Ackerstrasse 113, CH-5070 Frick, Switzerland; 12 Institute of Genetics and Animal Breeding, Polish Academy of Science, Jastrzębiec, Postępu 36, Magdalenka 05-552, Poland; 13 Human Development and Health Academic Unit, Faculty of Medicine, University of Southampton, Southampton SO16 6YD, UK; 14 National Agricultural Research Foundation (NAGREF), Veterinary Research Institute of Thessaloniki, Thermi 57001, Thessaloniki, Greece; 15 School of Agriculture, Policy and Development, Centre for Dairy Research, Food Production and Quality Division, University of Reading, PO Box 237, Earley Gate, Reading RG6 6AR, UK; 16 Kelkit Aydin Vocational Training School, Gumushane University, 29600 Kelkit, Gumushane, Turkey

**Keywords:** Organic products, Milk, Dairy products, Vitamins, Antioxidants, *n*-3 PUFA, Conjugated linoleic acid

## Abstract

Demand for organic milk is partially driven by consumer perceptions that it is more nutritious. However, there is still considerable uncertainty over whether the use of organic production standards affects milk quality. Here we report results of meta-analyses based on 170 published studies comparing the nutrient content of organic and conventional bovine milk. There were no significant differences in total SFA and MUFA concentrations between organic and conventional milk. However, concentrations of total PUFA and *n*-3 PUFA were significantly higher in organic milk, by an estimated 7 (95 % CI −1, 15) % and 56 (95 % CI 38, 74) %, respectively. Concentrations of *α*-linolenic acid (ALA), very long-chain *n*-3 fatty acids (EPA+DPA+DHA) and conjugated linoleic acid were also significantly higher in organic milk, by an 69 (95 % CI 53, 84) %, 57 (95 % CI 27, 87) % and 41 (95 % CI 14, 68) %, respectively. As there were no significant differences in total *n*-6 PUFA and linoleic acid (LA) concentrations, the *n*-6:*n*-3 and LA:ALA ratios were lower in organic milk, by an estimated 71 (95 % CI −122, −20) % and 93 (95 % CI −116, −70) %. It is concluded that organic bovine milk has a more desirable fatty acid composition than conventional milk. Meta-analyses also showed that organic milk has significantly higher *α*-tocopherol and Fe, but lower I and Se concentrations. Redundancy analysis of data from a large cross-European milk quality survey indicates that the higher grazing/conserved forage intakes in organic systems were the main reason for milk composition differences.

The demand for organic dairy products has increased rapidly over the past 20 years^(^ ^1^ ^)^. Dairy products currently account for 15 % of the total organic food market in the USA and up to 30 % in some European countries^(^ ^2^ ^,^ ^3^ ^)^. A main driver for the increase in demand has been the consumer perception that organic milk and dairy products typically contain higher concentrations of nutritionally desirable compounds, therefore making them ‘healthier’^(^ ^4^ ^,^ ^5^ ^)^. There is also concern among consumers about pesticide residues in milk^(^ ^6^ ^–^ ^8^ ^)^, although regulatory bodies in Europe maintain that there is no risk from pesticide residues in food^(^ ^9^ ^)^. However, there is still considerable uncertainty over whether, and to what extent, the use of organic production standards results in significant changes in the nutritional quality of milk and dairy products^(^ ^5^ ^,^ ^10^ ^–^ ^12^ ^)^.

Over the past 20 years, a large number of scientific studies have compared concentrations of nutritionally relevant compounds in milk from organic and conventional dairy production systems. Most of them focused on comparing milk fat composition, but there are also some published data on antioxidant, vitamin and/or mineral concentrations in milk and dairy products^(^ ^10^ ^,^ ^13^ ^,^ ^14^ ^)^. There has been a particular interest in comparing concentrations of nutritionally relevant, SFA, MUFA and PUFA. It is well documented that SFA and in particular myristic acid (14 : 0) and palmitic acid (16 : 0), and possibly also lauric acid (12 : 0), affect the relative proportions of HDL- and LDL-cholesterol and increase the risk of CVD in humans^(^ ^15^ ^)^. SFA in milk are therefore widely considered to have negative effects on human health^(^ ^15^ ^)^, although this is not universally accepted^(^ ^16^ ^–^ ^18^ ^)^. In contrast, the PUFA linoleic acid (LA) and *α*-linolenic acid (ALA), EPA, DPA and DHA have been shown to induce protective effects against CVD^(^ ^19^ ^)^. LA is known to reduce LDL production and enhance its clearance, whereas EPA and DHA reduce arrhythmia, blood pressure, platelet sensitivity, inflammation and serum TAG levels^(^ ^19^ ^)^.

Increased intakes of very long-chain (VLC) *n*-3 PUFA (EPA+DPA+DHA) have also been linked to other health benefits, including improved fetal brain development and function, delayed decline in cognitive function in elderly men and reduced risk of dementia (especially Alzheimer’s disease)^(^ ^20^ ^)^.

The PUFA conjugated linoleic acid (CLA) has been linked to anti-obesity, anti-carcinogenic, anti-atherogenic, anti-hypertension, anti-adipogenic and anti-diabetogenic effects, as well as improved immune system function and bone formation. However, most evidence for potential positive health impacts of CLA is from *in vitro* or animal studies, and there is considerable controversy over whether, and to what extent, increasing CLA intake will result in health benefits in humans^(^ ^21^ ^–^ ^25^ ^)^.

Three previous systematic literature reviews^(^ ^10^ ^,^ ^13^ ^,^ ^14^ ^)^ used meta-analyses methods to synthesise published information on composition differences between organic and conventional milk and/or dairy products, but report contrasting results and conclusions (see the online Supplementary data for a detailed description and discussion of the results of previous meta-analyses). As a result, they contributed substantially to the existing uncertainty about the impact of organic production methods on the nutritional composition of milk and dairy products. All three systematic reviews/meta-analyses were based on only a small proportion (<20 %) of the information published to date, limiting the statistical power of the meta-analyses, especially for parameters in which the number of data sets available was relatively small^(^ ^26^ ^)^. Results from two recent large milk quality surveys from the European Union and USA^(^ ^27^ ^,^ ^28^ ^)^ indicated that there is significant regional variation in the relative differences in fatty acid (FA) composition between organic and conventional milk, which may also reduce the statistical power of meta-analyses.

There has also been a recent qualitative literature review^(^ ^29^ ^)^ that discussed composition differences between organic and conventional milk reported in selected studies in the context of experiments focused on identifying the effect of management practices on milk composition.

Although meta-analyses of published comparative studies may quantify potential composition differences between organic and conventional dairy products, they cannot identify the contribution of specific agronomic drivers – for example animal diet, breed choice and other management parameters – used in organic and conventional livestock production. This is mainly because in most comparative studies the management practices used in both organic and conventional production systems are described in insufficient detail^(^ ^30^ ^,^ ^27^ ^)^. However, for the dairy sector, there are now five publications reporting data from a large cross-European milk quality survey in which bovine milk composition parameters and management practices, including breeds used, feeding regimens and milking systems, were recorded using common methods^(^ ^27^ ^,^ ^30^ ^–^ ^34^ ^)^. This unique data set allows, for the first time, the main agronomic drivers for differences in milk composition between organic and conventional farming systems to be investigated by redundancy analysis (RDA).

Therefore, the main objectives of the present study were to (1) carry out a systematic literature review of all available studies published before March 2014 that focused on quantifying composition differences between organic and conventional milk and dairy products; (2) conduct weighted and unweighted meta-analyses (WM and UM) of the published data; (3) carry out sensitivity analyses focused on identifying to what extent meta-analysis results are affected by data extraction (e.g. using data reported for different years/seasons as separate events or means of data from different years/seasons) or inclusion criteria (e.g. including or excluding comparisons involving milk composition data from non-standard conventional or organic systems; excluding data from the 20 % of studies with the least precise treatment effects, those having the largest variances identified in WM); and (4) perform redundancy and correlation analyses using data from a large cross-European farm survey^(^ ^27^ ^,^ ^30^ ^–^ ^34^ ^)^ of dairy cow management, milk yield and quality parameters to identify management parameters associated with differences in composition between organic and conventional milk and associations between productivity and milk quality in organic and conventional dairy systems.

## Methods

### Data acquisition: literature search strategy and inclusion criteria

**Fig. 1:**
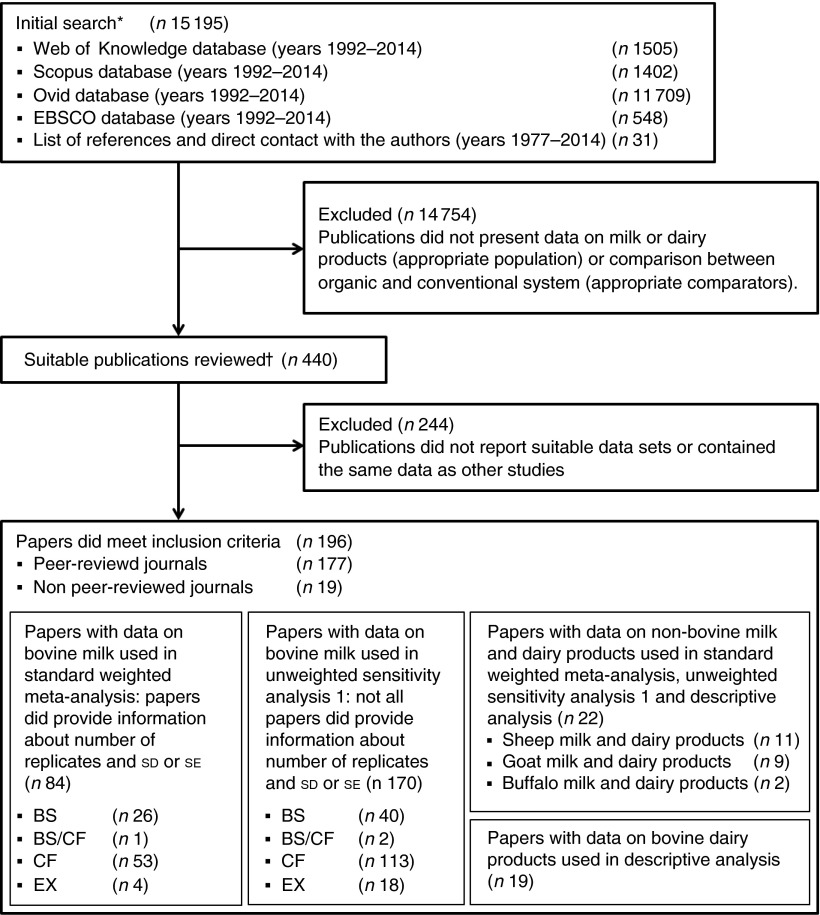
Summary of the search and selection protocols used to identify papers included in the systematic review and the meta-analyses. * Review carried out by one reviewer; † data extraction carried out by two reviewers. CF, comparison of matched farms; BS, basket studies; EX, controlled experiments.

The review methods were described in detail in a previously published meta-analysis by Barański *et al*.^(^ ^35^ ^)^, which assessed composition differences between organic and conventional crops. Relevant publications were identified through an initial search of literature in the Web of Knowledge, Scopus, Ovid and EBSCO databases using the search terms (organic* or ecologic* or biodynamic*) and (conventional* or integrated) and (livestock or dairy or milk or cheese or cream or curd or butter or yoghurt) (Fig. 1).

Papers in all languages, published in peer-reviewed and non-peer-reviewed journals reporting data on both desirable and undesirable compositional parameters, were considered relevant for inclusion in the meta-analyses. The search was restricted to the period between 1992 (the year when legally binding organic farming regulations were first introduced in the European Union) and the end of the project in March 2014 and provided 15 164 references. An additional thirty-one publications were found by studying lists of references or directly contacting authors of published papers and reviews identified in the initial literature search (Fig. 1). This included suitable data from scientific papers published before 1992 that were identified/used in previous systematic literature reviews/meta-analyses^(^ ^10^ ^,^ ^14^ ^)^.

The abstracts of all publications were then examined by two reviewers to determine whether they contained original data on milk or dairy products (appropriate population) obtained by comparing composition parameters in organic and conventional system (appropriate comparators). This identified 440 suitable publications, from which 244 were subsequently rejected, because they did not meet inclusion criteria or reported duplicated information.

Publications were eligible for inclusion if data for milk yield and/or at least one composition parameter in milk or dairy products were reported. As a result, 196 publications (177 peer-reviewed) were selected for data extraction (170 on bovine milk, nineteen on bovine dairy products, eleven on sheep milk and dairy products, nine on goat milk and dairy products, two on buffalo milk and dairy products). Data from eighty-nine publications (seventy-nine peer-reviewed) fulfilled the criteria for inclusion in random-effects WM. Because of the limited data available for sheep, goat and buffalo milk and dairy products, only data for bovine milk were included in meta-analyses presented in the main paper. Results from meta-analyses of pooled data for goat, sheep and buffalo milk, which was possible for only a small number of composition parameters, are presented in the Supplementary Information only (online Supplementary Fig. S35).

Previous systematic reviews/meta-analyses of comparative studies into milk quality by Dangour *et al.* ^(^ ^10^ ^)^, Palupi *et al.* ^(^ ^13^ ^)^ and Smith-Spangler *et al*.^(^ ^14^ ^)^ were based on a more limited proportion of the literature available (twelve, thirteen and thirty-seven publications, respectively). However, most publications included in these previous reviews were also used in the standard WM reported here, except for one publication on sheep and goats milk included by Palupi *et al.* ^(^ ^13^ ^)^ and one publication on milk included by Dangour *et al.* ^(^ ^10^ ^)^ that reported the same data as other publications selected for extraction in this study.

A Preferred Reporting Items for Systematic Reviews and Meta-Analyses (PRISMA) flow diagram illustrates the search and study inclusion strategies (Fig. 1). Eligibility assessment was performed by two independent reviewers, with discrepancies resolved by consensus and reference to a third reviewer as necessary.

### Data extraction

Data were extracted from three types of studies: (1) comparisons of matched farms (CF), farm surveys in which milk was obtained from organic and conventional farms in the same country or region; (2) basket studies (BS), retail product surveys in which organic and conventional milk was obtained in retail outlets; and (3) controlled experiments (EX) in which milk was obtained from experimental animals managed according to organic or conventional farming standards/protocols. Data from the three study types were subject to meta-analysis if the authors stated that (1) organic farms included in farm surveys were using organic farming methods; (2) organic milk collected in retail surveys were labelled as organic; or (3) animals from organically reared herds used in EX were managed according to organic farming standards, even if animals and land used for ‘organic treatments’ in experiments were not organically certified.

Several studies compared more than one organic or conventional system or treatment (online Supplementary Table S3). For example, additional conventional systems/treatments were described as ‘low input’, ‘intensive’ or ‘extensive’, and an additional organic system/treatment included in some studies was described as ‘biodynamic’. In such cases, only the organic and conventional system identified by the authors as closest to the typical, contemporary organic/conventional farming system was used in the meta-analysis, as recommended by Brandt *et al.* ^(^ ^11^ ^)^. Full references of the publications and summary descriptions of studies included in the meta-analyses are given in the online Supplementary Tables S1–S3.

Information and data were extracted from all selected publications and compiled in a Microsoft Access database. The database will be made freely available on the Newcastle University website (http://research.ncl.ac.uk/nefg/QOF) for use and scrutiny by others. A list of the information extracted from publications and recorded in the database is given in the online Supplementary Table S4.

Data reported as numerical values in the text or tables were copied directly into the database. Results only published in graphical form were enlarged, printed, measured (using a ruler) and then entered into the database, as previously described^(^ ^35^ ^)^.

Data reported in the same publication for different study types, countries and outcomes were treated as independent effects. However, data extracted from the same publication for (1) different years and (2) different regions, retail outlets or brands in the same country or (3) multiple time points within the same sampling year were averaged before use in the meta-analyses.

Risk of bias of individual studies was based on (1) study type and probability of confounding, (2) production system and magnitude of effect.

Two independent reviewers assessed publications for eligibility and extracted data. Discrepancies were detected for approximately 4 % of the data, and in these cases extraction was repeated following discussion.

Raw data from a previously published large cross-European farm survey^(^ ^27^ ^,^ ^30^ ^–^ ^34^ ^)^ were obtained directly from the authors and used in both the meta-analyses and RDA; this included some data sets (e.g. for individual SFA or carotenoids) that were not previously reported^(^ ^27^ ^,^ ^30^ ^–^ ^34^ ^)^.

Study characteristics, summaries of methods used for sensitivity analyses and ancillary information are given in the online Supplementary Tables S2–S7. They include information on (1) the number of papers from different countries and publication years used in meta-analyses (online Supplementary Fig. S1 and S2); (2) study type and locations identified in different studies (online Supplementary Table S2); (3) production system information for studies with more than two systems (online Supplementary Table S3); (4) the type of information extracted from papers (online Supplementary Table S4); (5) data-handling and inclusion criteria, and meta-analysis methods used in sensitivity analyses (online Supplementary Table S5); (6) the list of composition parameters included in meta-analyses (online Supplementary Table S6); and (7) the list of composition parameters for which meta-analyses were not possible (*n*<3) (online Supplementary Table S7).

**Fig. 2:**
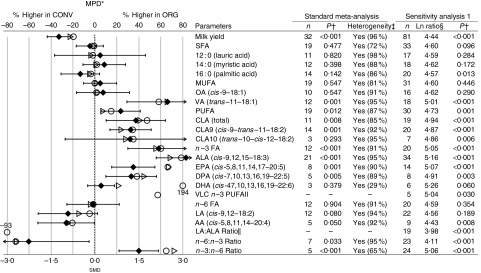
Results of the standard meta-analyses and sensitivity analysis 1 for fat composition in cows’ milk. * Numerical values for mean percentage difference (MPD) and 95 % CI are given in the online Supplementary Table S9. † Significantly different between organic samples (ORG) and conventional samples (CONV) (*P*<0·05). ‡ Heterogeneity and the *I* ^2^ statistic. § Ln ratio=Ln(ORG/CONV×100 %). || Calculated based on published fatty acid (FA) composition data. 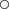, MPD calculated using data included in sensitivity analysis 1; 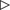, MPD calculated using data included in standard meta-analysis; 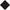, standardised mean difference (smd) from the standard meta-analysis with 95 % CI represented by horizontal bars. *n*, number of data points included in meta-analyses; OA, oleic acid; VA, vaccenic acid; CLA, conjugated linoleic acid; ALA, *α*-linolenic acid; VLC *n*-3 PUFA, very long-chain *n*-3 PUFA (EPA+DPA+DHA); LA, linoleic acid; AA, arachidonic acid.

**Fig. 3:**
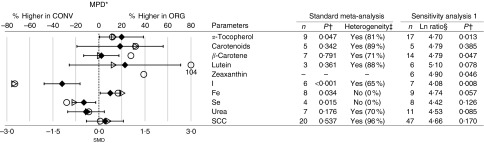
Results of the standard meta-analyses and sensitivity analysis 1 for antioxidants, minerals, urea and somatic cell count (SCC) in cows’ milk. * Numerical values for mean percentage difference (MPD) and 95 % CI are given in the online Supplementary Table S9. † Significantly different between organic samples (ORG) and conventional samples (CONV) (*P*<0·05). ‡ Heterogeneity and the *I* ^2^ statistic. § Ln ratio=Ln(ORG/CONV×100 %). || Calculated based on published fatty acid composition data. 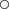, MPD calculated using data included in sensitivity analysis 1; 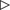, MPD calculated using data included in standard meta-analysis; 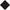, standardised mean difference (smd) from the standard meta-analysis with 95 % CI represented by horizontal bars; *n*, number of data points included in meta-analyses.

The online Supplementary Table S8 summarises basic statistics on the number of studies, individual comparisons, organic and conventional samples sizes, and comparisons showing statistically or numerically higher concentrations in organic or conventional milk samples for the composition parameters included in Fig. 2 and 3.

### Meta-analyses

Nine analyses were undertaken (online Supplementary Table S5). The methods used for random-effects WM and UM sensitivity analyses 1 were described by Barański *et al*.^(^ ^35^ ^)^ and compared only pragmatically chosen standard organic and conventional systems. Fig. 2 and 3 show the pooled effects obtained using random-effects meta-analysis weighted by inverse variance and a common random-effects variance component and unweighted analysis of differences in means. The WM analysis is the primary analysis, but it is useful to augment the results with UM (particularly to explore the impact of including data from the studies that do not report measures of variance and thus a wider range of studies).

Eight sensitivity analyses were carried out (online Supplementary Table S5). Four analyses (sensitivity analyses 2, 3, 6 and 7; online Supplementary Table S5) were designed to identify whether inclusion of data for individual experimental years as separate data points affected the results of meta-analyses. Four analyses (sensitivity analysis 4–7; online Supplementary Table S5) were carried out to identify whether exclusion of data for comparisons with non-standard organic or conventional systems affected the results of meta-analyses; in these analyses, comparative data for all organic and conventional production systems reported by authors were included (online Supplementary Table S3). In sensitivity analysis 8 we explored the effect of excluding 20 % of studies with the least precise treatment effects from the WM. Results of these sensitivity analyses are available in the appendix on the Newcastle University website (http://research.ncl.ac.uk/nefg/QOF).

Effect sizes for all WM were based on standardised mean differences (smd), as recommended for studies that include data measuring the same parameters on different scales^(^ ^36^ ^,^ ^37^ ^)^.

Both WM and UM were carried out using the R statistical programming environment (http://www.r-project.org/). WM, with the smd as the basic response variable, were conducted using standard methods and the open-source ‘metafor’ statistical package^(^ ^38^ ^–^ ^41^ ^)^. A detailed description of the methods and calculations is provided in the ‘Additional Methods Description’ published by Barański *et al.* ^(^ ^35^ ^)^ (available online).

A positive smd value indicates that mean concentrations of the observed constituents were greater in the organic milk samples, whereas a negative smd indicates that mean concentrations were higher in conventional (non-organic) samples. The statistical significance of a reported effect size (i.e. smd_tot_) and CI were estimated based on standard methods^(^ ^42^ ^)^ using ‘metafor’^(^ ^38^ ^)^. The influence of study type (CF, EX, BS) as a potential moderator was tested using mixed-effect models^(^ ^43^ ^)^ and subgroup analyses (online Supplementary Fig. 3–33).

We carried out tests of homogeneity (*Q* statistics and *I* ^2^ statistics) on all summary effect sizes. Homogeneity was indicated if *I* ^2^ was <25 % and the *P* value for the *Q* statistics was >0·010. Funnel plots, Egger tests of funnel plot asymmetry and fail-safe number tests were used to assess publication bias^(^ ^44^ ^)^ (see the online Supplementary Table S13 for further information).

For the UM, the ratio of organic means:conventional means (*X̅*_O_ */X̅*_C_) expressed as a percentage was ln-transformed, and values were used to determine whether the arithmetic average of the ln-transformed ratios was significantly greater than ln(100), using resampling^(^ ^45^ ^)^. Reported *P* values were derived from Fisher’s one-sample randomisation test^(^ ^46^ ^)^, and a *P*<0·05 was considered statistically significant.

For parameters that were calculated based on published information (total VLC *n*-3 PUFA, LA:ALA ratio), it was only possible to carry out UM (Fig. 2), as measures of variance were not available.

Forest plots were constructed to show pooled smd and corresponding 95 % CI for all compositional parameters investigated. Additional forest plots were presented for selected results to illustrate heterogeneity between individual studies and study types (see the online Supplementary Fig. 3–33).

The mean percentage difference (MPD) was calculated for all parameters for which statistically significant effects were detected by either UM or WM. This was done to facilitate value judgements regarding the biological importance of the relative effect magnitudes using the calculations described by Barański *et al*.^(^ ^35^ ^)^.

We also calculated MPD using data-pairs included in the UM and WM, to estimate the impact of excluding data, for which no measures of variance were reported, on the magnitude of difference. As the MPD can be expressed as ‘% higher’ in conventional or organic milk, they provide estimates for the magnitude of composition differences that are easier to relate to existing information on potential health impacts of changing dietary intakes for individual or groups of compounds than the smd values. The 95 % CI for MPD were estimated using a standard method^(^ ^42^ ^)^.

An overall assessment of the strength of evidence was made using an adaptation of the Grading of Recommendation Assessment, Development and Evaluation (GRADE)^(^ ^47^ ^)^ system (Table 1).

**Table 1:** Grading of Recommendation Assessment, Development and Evaluation (GRADE) assessment of the strength of evidence for standard meta-analysis for parameters shown in Fig. 2 and 3 (Standardised mean difference values (smd) and 95 % confidence intervals)

Parameters	smd	95 % CI	Effect magnitude*	Inconsistency†	Precision‡	Publication bias§	Overall reliability||
Milk yield	−1·23	−1·64, −0·81	Large	Medium	High	No	High
SFA	−0·17	−0·66, 0·31	Small	Medium	High	Strong	Low
12 : 0 (lauric acid)	0·18	−1·39, 1·75	Small	High	Poor	Medium	Very low
14 : 0 (myristic acid)	0·32	−0·42, 1·05	Small	High	Moderate	Medium	Very low
16 : 0 (palmitic acid)	−0·50	−1·17, 0·17	Moderate	Medium	Moderate	Strong	Low
MUFA	0·18	−0·4, 0·76	Small	Medium	Moderate	Strong	Very low
OA (*cis*-9-18 : 1)	0·28	−0·64, 1·2	Small	Low	Poor	Medium	Low
VA (*trans*-11-18 : 1)	2·48	1·08, 3·87	Large	Medium	Moderate	Medium	Moderate
PUFA	0·88	0·19, 1·56	Large	Medium	Moderate	No	Moderate
CLA (total)	1·40	0·37, 2·42	Large	Medium	Moderate	Medium	Moderate
CLA9 (*cis*-9-*trans*-11-18 : 2)	1·22	0·5, 1·95	Large	Low	Moderate	Medium	Moderate
CLA10 (*trans*-10-*cis*-12-18 : 2)	1·20	−1·03, 3·43	Large	Medium	Poor	Medium	Low
*n*-3 FA	2·18	1·11, 3·25	Large	Low	Moderate	Medium	Moderate
ALA (*cis*-9,12,15-18 : 3)	3·05	2·08, 4·02	Large	Medium	High	Medium	Moderate
EPA (*cis*-5,8,11,14,17-20 : 5)	1·31	0·56, 2·06	Large	Medium	Moderate	Medium	Moderate
DPA (*cis*-7,10,13,16,19-22 : 5)	1·24	0·37, 2·12	Large	Low	Moderate	Medium	Moderate
DHA (*cis*-4,7,10,13,16,19-22 : 6)	0·21	−0·26, 0·68	Small	Low	High	No	Moderate
VLC *n*-3 PUFA¶	–	–	–	–	–	–	–
*n*-6 FA	−0·06	−0·97, 0·86	Small	High	Moderate	Medium	Very low
LA (*cis*-9,12-18 : 2)	−0·92	−1·96, 0·11	Moderate	Medium	Poor	Medium	Low
AA (*cis*-5,8,11,14-20 : 4)	−0·98	−1·95, 0	Moderate	Medium	Poor	Strong	Very low
LA:ALA ratio¶	–	–	–	–	–	–	–
*n*-6:*n*-3 Ratio	−2·26	−4·34, −0·18	Large	High	Poor	Medium	Low
*n*-3:*n*-6 Ratio	1·50	0·81, 2·19	Large	Low	Moderate	Medium	Moderate
*α*-Tocopherol	0·74	0·01, 1·47	Moderate	Medium	Moderate	Medium	Low
Carotenoids	0·69	−0·73, 2·1	Moderate	High	Poor	No	Low
* β*-Carotene	0·08	−0·51, 0·67	Small	Low	Moderate	No	Moderate
Lutein	0·85	−0·98, 2·68	Large	Medium	Poor	No	Moderate
Zeaxanthin	–	–	–	–	–	–	–
I	−1·20	−1·8, −0·59	Large	Low	Moderate	No	High
Fe	0·37	0·03, 0·71	Moderate	Low	High	No	High
Se	−0·49	−0·89, −0·1	Moderate	Low	High	Medium	Moderate
Urea	−0·42	−1·04, 0·19	Moderate	Low	Moderate	No	Moderate
SCC	0·20	−0·43, 0·82	Small	Medium	Moderate	Medium	Low

OA, oleic acid; VA, vaccenic acid; CLA, conjugated linoleic acid; FA, fatty acids; ALA, *α*-linolenic acid; VLC *n*-3 PUFA, very long-chain *n*-3 PUFA (EPA+DPA+DHA); LA, linoleic acid; AA, arachidonic acid.

* Study quality was considered low because of high risks of bias and potential for confounding. However, we considered large effects to mitigate this *sensu* GRADE; large effects were defined as >20 %, moderate effects 10–20 and small <10 %.

† Inconsistency was based on the measure of heterogeneity and consistency of effect direction *sensu* GRADE.

‡ Precision was based on the width of the pooled effect CI and the extent of overlap in substantive interpretation of effect magnitude *sensu* GRADE.

§ Publication bias was assessed using visual inspection of funnel plots, the Egger tests, two tests of fail-safe *n*, and trim and fill (see the online Supplementary Table 13). Overall publication bias was considered high when indicated by two or more methods, moderate when indicated by one method and low when no methods suggested publication bias.

|| Overall quality of evidence was then assessed across domains as in standard GRADE appraisal; high when there was very high confidence that the true effects lie close to that of estimate, moderate when there was moderate confidence in effect estimate and the true effect is likely to be close to the estimate but there is a possibility that it is substantially different, low when the confidence in the effect estimate was limited and the true effect may be substantially different from the estimate, very low when there was very little confidence in the effect estimate and the true effect is likely to be substantially different from the estimate.

¶ Calculated based on published fatty acid composition data.

### Estimation of *n*-3 fatty acid and conjugated linoleic acid intakes

FA intakes were calculated using the following formula: total fat intake from milk×proportion of specific FA (*n*-3 PUFA, ALA, EPA, DHA, CLA) in total milk FA×0·933 (the proportion of FA in total milk lipids)^(^ ^48^ ^)^. To estimate the effect of switching from conventional to organic milk/dairy products, estimated dietary intakes of ALA and EPA+DHA from dairy products were compared with European Food Safety Authority (EFSA) recommended intakes of 1100 and 250 mg/d, respectively^(^ ^49^ ^)^. EFSA recommendations for ALA intake, given relative to total energy intake, were transformed into mg/d, assuming average dietary energy intakes of 8·4 MJ/d (2000 kcal/d)^(^ ^50^ ^)^ and FA energy content of 37·7 kJ/g (9 kcal/g)^(^ ^51^ ^)^.

### Redundancy analyses

The relationships between feeding/management practices and breed index (proportion of Holstein Friesian cows in the herd) and the nutritional composition of milk were investigated using published data from extensive cross-European dairy farm and milk quality surveys^(^ ^27^ ^,^ ^30^ ^–^ ^34^ ^)^. RDA were carried out using the CANOCO statistical package^(^ ^52^ ^)^. The importance of individual factors (breed index, feed composition parameters and milking system) was assessed using automatic forward selection within RDA, with no interaction terms, using Monte Carlo permutation tests (9999 permutations for each randomisation test). Organic and conventional production practices were included as passive drivers in the RDA carried out to produce the bi-plot in Fig. 5.

A number of conventional farms included in the cross-European farm and milk quality survey used low-input (low concentrate, high-grazing-based forage intake) feeding regimens that conform to organic production standards. We therefore carried out a separate RDA in which high- and low-input conventional and organic production practice were used as separate drivers, to test whether associations between milk composition, and organic and low-input, and conventional feeding practices were similar (online Supplementary Fig. S34).

## Results

### Characteristics of studies/data included in meta-analyses

Analyses were based on data from 196 publications reporting results from farm surveys (127 papers), EX (twenty-two papers), BS (fifty-one papers) or results from more than one type of study (EX, CF and/or BS) (online Supplementary Table S2).

Approximately 76 % of studies included in meta-analyses were from Europe, mainly from Germany, Sweden, Denmark, UK, Italy and Norway, with most of the balance coming from the USA and Brazil (online Supplementary Table S2 and Fig. S2). A total of 187 studies reported composition data on fresh milk, whereas a smaller number of papers reported data for cheese (thirteen papers), yoghurt (four papers), fermented milk (three papers), curd (one paper) and butter (four papers) (online Supplementary Table S2). Only studies reporting data on fresh milk were included in meta-analyses.

Publications reported data on 418 different composition parameters in fresh milk and dairy products, of which 120 were included in meta-analyses (online Supplementary Tables S6 and S7).

**Fig. 4:**
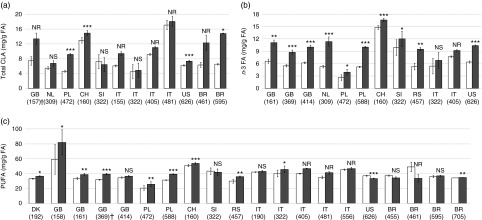
Summary of data presented in papers included in the standard meta-analysis for concentration of (a) total conjugated linoleic acid (CLA), (b) *n*-3 fatty acids (FA) and (c) PUFA content in cows’ milk. Values are means with, their standard errors for conventional (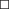) and organic (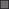) production system. Significant correlation: * *P*≤0·05; ** *P*≤0·01; *** *P*≤0·001; ^NS^ not significant; ^NR^ not reported. On x-axis country code according ISO 3166-2 (see http://www.iso.org/iso/home/standards/country_codes.htm) and study ID in parentheses (see the online Supplementary Table S1 for references). † Paper not included in standard meta-analysis for which values for measures of variance were obtained directly from authors.

**Fig. 5:**
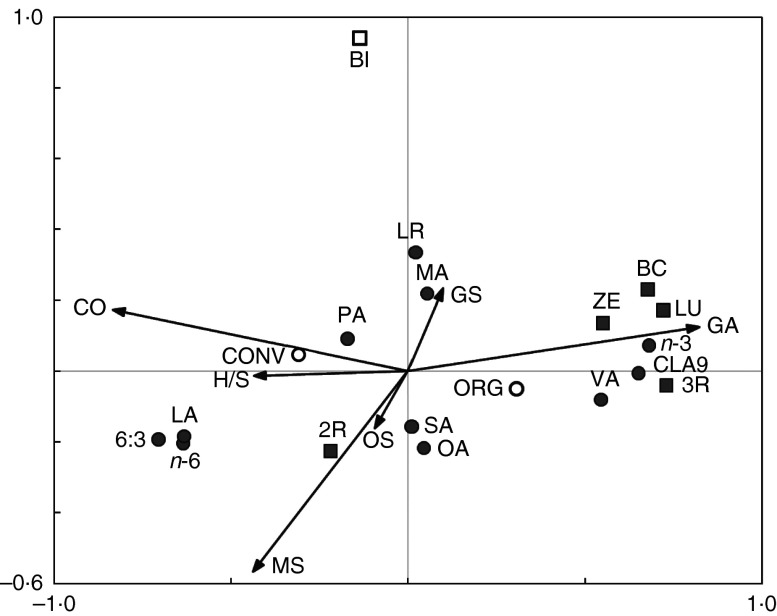
Bi-plot derived from the redundancy analysis showing the relationship between milk composition parameters (fatty acids (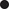) and antioxidants (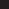)) and cows’ feeding and rearing parameters (categorical explanatory variables (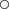, 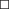)) and quantitative explanatory variables (**→**). 6:3, *n*-3:*n*-6 Fatty acid ratio; 2R, synthetic isomers of *α*-tocopherol; 3R, natural isomers of *α*-tocopherol; BC, *β*-carotene; BI, breed index; CLA9, rumenic acid (*cis*-9,*trans*-11-18 : 2); CO, concentrate feeds; CONV, conventional production system; GA, grazing intake; GS, grass silage; H/S, hay or straw; LA, linoleic acid (*cis*-9,12-18 : 2); LU, lutein; LR, lauristic acid (12 : 0); MA, myristic acid (14 : 0); MS, maize silage; OA, oleic acid (*cis*-9-18 : 1); ORG, organic production system; OS, other silage; PA, palmitic acid (16 : 0); SA, stearic acid (18 : 0); VA, vaccenic acid (*trans*-11-18 : 1); ZE, zeaxanthin.

Studies were universally judged to be at high/unclear risk of bias as a result of poor reporting. Insufficient detail was provided to assess probability of confounding as a source of heterogeneity (online Supplementary Table S2). The impact of the production system on the effect magnitude was ascertained where data were available using RDA (Fig. 5), but insufficient detail was reported in the majority of individual studies resulting in high/unclear risk of bias. However, country and production system did explain heterogeneity in meta-regressions, which may be related to risk of bias (Fig. 4). Overall risk of bias was considered high, but this was mitigated by large effect magnitudes for fourteen of thirty-one outcomes (Table 1).

### Milk yield per cow

WM showed that the average milk yield (kg milk/cow per d or kg milk/lactation) was significantly lower in organic (−23; 95 % CI −31, −15 %) compared with conventional production systems (Fig. 2; online Supplementary Table S9 and Fig. S3). However, no significant effect of production system was detected for the fat and protein content of milk. Total milk protein and fat yield per cow were therefore also estimated to be approximately 20 % lower for organic herds (online Supplementary Table S11).

### Composition of organic and conventional bovine milk

#### Fatty acid composition

For FA composition, a substantial evidence base (number of comparisons) was available and for most nutritionally relevant parameters more than ten comparative data-pairs were available for WM. The main exceptions were CLA (*trans*-10-*cis*-12-18 : 2), the VLC *n*-3 PUFA (EPA+DPA+DHA) and arachidonic acid (AA) for which less than eight data-pairs were available for WM (Fig. 2).

WM showed that organic and conventional milk had similar concentrations of total SFA and MUFA, but detected significantly higher concentrations of total PUFA in organic milk with an MPD of 7·3 (95 % CI −0·7, 15) %.

Among the PUFA, the largest differences were found for *n*-3 PUFA. WM detected significantly higher concentrations of total *n*-3 PUFA, ALA, EPA and DPA, in organic compared with conventional milk (Fig. 2). The MPD was 56 (95 % CI 38, 74) % for total *n*-3 PUFA, 68 (95 % CI 53, 84) % for ALA, 67 (95 % CI 32, 102) % for EPA, 45 (95 % CI 18, 71) % for DPA and 21 (95 % CI −3, 47) % for DHA (Fig. 2; online Supplementary Table S9).

WM also detected significantly higher total CLA (all CLA isomers) and CLA9 (*cis*-9,*trans*-11-18 : 2; the dominant CLA isomer found in milk) and vaccenic acid (VA, a MUFA metabolised to CLA9 by mammals, including humans) in organic milk (Fig. 2). The MPD were 41 (95 % CI 14, 68) % for total CLA, 24 (95 % CI 8, 39) % for CLA9 and 66 (95 % CI 20, 112) % for VA (Fig. 2; online Supplementary Table S9).

In contrast, no significant differences in the concentration of total *n*-6 PUFA and LA (the dominant *n*-6 FA found in milk) were found between organic and conventional milk (Fig. 2). However, WM detected significantly lower concentrations of the *n*-6 PUFA AA (another *n*-6 FA) in organic milk (Fig. 2). The LA:ALA and *n*-6:*n*-3 PUFA ratios were therefore significantly lower in organic compared with conventional milk (Fig. 2).

The LA:ALA ratio was 2·8 (95 % CI 2·0, 3·6) % in organic and 5·0 (95 % CI 1·1, 23·1) % in conventional milk and the *n*-6:*n*-3 ratio was 3·6 (95 % CI 1·9, 5·2) % in organic and 5·4 (95 % CI 3·4, 7·4) % in conventional milk (Fig. 2; online Supplementary Table S9).

UM (sensitivity analysis 1 carried out to assess the impact of including data from a larger number of studies) gave very similar results to WM (Fig. 2). UM was also carried out for total VLC *n*-3 PUFA (EPA+DPA+DHA) and detected significantly higher concentrations in organic milk with an MPD of 57 (95 % CI 27, 87) %.

For a range of specific SFA, MUFA and PUFA and other FA groups, WM did not detect significant differences, and this included 4 : 0 (butyric acid), 6 : 0 (caproic acid), 10 : 0 (capric acid), 13 : 0 (tridecylic acid), 18 : 0 (stearic acid), 12 : 0+14 : 0+16 : 0 (unsaturated fatty acids), 18 : 1, 18 : 2, 18 : 3, 10 : 1 (4-*cis*-decenoic acid), 12 : 1 (lauroleic acid), 14 : 1 (myristoleic acid), 16 : 1 (palmitoleic acid), 17 : 1 (heptadecenoic acid), *cis*-11-18 : 1 (*cis*-VA), *cis*-12-18 : 1, *cis*-13-18 : 1, *trans*-9-18 : 1 (elaidic acid), *trans*-12-18 : 1, *trans*-6-8-18 : 1, CLA (*trans*-7,9-18 : 2), CLA (*trans*-9,11-18 : 2), CLA (*trans*-11,13-18 : 2), CLA (*trans*-12,14-18 : 2), *cis*-11,14-20 : 2, eicosatrienoic acid (*cis*-11,14,17-20 : 3), long-chain FA, medium-chain FA and SCFA (online Supplementary Table S12).

Results of the unweighted sensitivity analysis 1 (UM) were broadly similar, but UM also detected significantly lower 16 : 0 and AA concentrations, significantly higher CLA (*trans*-10-*cis*-12-18 : 2) and total VLC *n*-3 PUFA (EPA+DPA+DHA) and a lower LA:ALA ratio in organic milk (Fig. 2).

#### Antioxidants/vitamins and minerals

The available evidence base for antioxidants/vitamins and minerals was smaller than for FA composition. With the exception of *α*-tocopherol, *β*-carotene, I and Fe (for which nine, seven, six and eight data-pairs were available for WM, respectively), the number of data-pairs available for WM was five or less (Fig. 3).

WM detected slightly, but significantly, higher *α*-tocopherol and Fe concentrations, but lower I and Se concentrations in organic compared with conventional milk (Fig. 3). The MPD was 13 (95 % CI 1, 26) % for *α*-tocopherol, 20 (95 % CI 0, 41) % for Fe, −74 (95 % CI −115, −33) % for I and −21 (95 % CI −49, 6) % for Se (Fig. 3; online Supplementary Table S9).

Results obtained by UM were broadly similar to those of the standard WM, but UM did detect significantly higher zeaxanthin concentrations in organic milk, but did not detect a significant difference for Fe (Fig. 3).

For a range of other vitamins/antioxidants and minerals, both WM and UM did not detect significant differences, including vitamin A, C, D_3_, vitamin E activity, Ca, Co, Cu, Mg, Mn, Mo, P, K, Na and Zn, as well as the toxic metals Ca and Pb, but the number of data-pairs available was low for most of these parameters (online Supplementary Tables S11 and S12).

#### Urea and somatic cell counts

For urea and somatic cell counts (SCC), a more substantial evidence base (seven and twenty-five data-pairs, respectively) was available for WM (Fig. 3). No significant differences in urea and SCC between organic and conventional milk could be detected (Fig. 3).

### Composition of organic and conventional sheep, goat and buffalo milk

There are currently very few published studies that report comparative yield (*n* 5) and/or composition data (*n* 3 or 4) for sheep, goat and/or buffalo milk. This makes it impossible to carry out accurate quantitative estimates of composition differences by meta-analysis. However, for parameters for which sufficient data (*n*≥3) were available, we carried out WM to test whether there may be similar trends to those detected for bovine milk (online Supplementary Fig. S35). When pooled data for sheep, goat and buffalo milk were compared by WM, no significant difference in milk yield per animal, PUFA and VA concentrations and SCC were detected. However, significantly higher concentrations of MUFA, CLA9 and ALA, and significantly lower concentrations of LA in organic milk, were detected and there was a trend (*P*=0·09) towards higher PUFA concentrations in organic milk.

### Effects of country/geographic region, study type and other sources of variation

Comparison of concentrations of total PUFA, *n*-3 PUFA and CLA in organic and conventional bovine milk from different countries/geographic regions showed considerable variation between countries (and in some cases also between different studies from the same country) (Fig. 4).

Heterogeneity was high (*I* ^2^>75 %) for approximately two-thirds of bovine milk composition parameters included in WM (nineteen of the thirty-one parameters shown in Fig. 2 and 3), with *I* ^2^ ranging from 98 % for lauric acid to 81 % for MUFA. On the other hand, for approximately one-third of composition parameters (twelve of the thirty-one parameters shown in Fig. 2 and 3), low or moderate heterogeneity was detected with *I* ^2^ ranging from 0 % for Fe and Se to 72 % for SFA (Fig. 2 and 3).

No substantive funnel plot asymmetry was detected for any parameters shown in Fig. 2 and 3, except for milk yield, palmitic acid, MUFA and AA, for which strong funnel plot asymmetry consistent with a publication bias was detected. However, it is not possible to definitively attribute discrepancies between large, precise studies and small imprecise studies to publication bias, which is strongly suspected, rather than detected, where asymmetry is severe (Table 1; online Supplementary Table S13).

When meta-analysis results obtained from different study types (BS, CF, EX) were compared, broadly similar results were obtained for most composition parameters included in Fig. 2 (online Supplementary Fig. S3–S33). However, differences between study types were detected for 12 : 0 (lauric acid) and oleic acid (OA) (online Supplementary Fig. S5 and S9). For many parameters, there was considerable variation between results obtained in different countries and in some cases also different studies carried out in the same country (online Supplementary Fig. 3–33).

For many parameters, MPD based on all available data produced values similar to those calculated using only data for which measures of variance were reported (i.e. those qualifying for WM) (Fig. 2 and 3; online Supplementary Table S9). However, for DHA, *β*-carotene and lutein, inclusion criteria had a large effect on the MPD.

In addition, when the calculated MPD were superimposed onto smd (with 95 % CI) results at an appropriate scale (−80 to +80 for MPD and −3 to +3 for smd), a reasonable match was observed, with MPD for most constituents falling within the 95 % CI for smd (Fig. 2 and 3). However, for some parameters (EPA, DHA, *n*-3:*n*-6 ratio and I), MPD fell outside the 95 % CI of smd and therefore ought to be seen as less reliable.

For the composition parameters included in Fig. 2 and 3, sensitivity analyses based on (1) different inclusion criteria/data-handling methods for UM or WM or (2) exclusion of 20 % of studies with the least precise treatment effects from the WM produced broadly similar results to the standard meta-analysis protocols.

Overall assessment of the strength of evidence using an adapted GRADE^(^ ^47^ ^)^ approach highlighted some uncertainties in the evidence base, but overall strength of evidence of WM results was high or moderate for seventeen of the thirty-one parameters shown in Fig. 2 and 3 (Table 1).

### Relationship between management and milk composition

The bi-plot derived from the RDA (Fig. 5) shows the relationships between diet components and the breed index (proportion on non-Holstein Friesian genetics in the herd), and the nutritional composition of milk. The horizontal axis 1 of the bi-plots explained 51 % of the variation and the vertical axis 2 a further 1·1 %. Variance in the RDA was explained by the intakes of concentrate feeds (*F=*241, *P=*0·002), hay and straw (*F=*64, *P=*0·002), maize silage (*F=*48, *P=*0·002), breed index (*F=*14, *P=*0·002), other silages (*F=*14, *P=*0·002) and grazing-based fresh forage intake (*F=*1, *P=*0·280).

RDA results indicated negative associations between concentrate, maize silage, other silages and hay and straw intakes and a number of nutritionally desirable FA (total PUFA, *n*-3 PUFA, ALA, CLA9) and antioxidants (3R stereoisomers of *α*-tocopherol, *β*-carotene, lutein and zeaxanthin) along axis 1. These milk composition parameters also showed strong positive associations with grazing intake (Fig. 5).

In contrast, there were positive associations between concentrate, maize silage, other silages and hay and straw intakes, and SFA, 16 : 0, total *n*-6 PUFA, LA, 2R stereoisomers of *α*-tocopherol and the *n*-6:*n*-3 PUFA ratio, along axis 1. The same milk composition parameters showed negative associations with grazing intake (Fig. 5).

Associations between the breed index and milk composition were generally weaker (Fig. 5).

Organic and conventional management were included as passive drivers in the RDA and aligned with the active drivers (1) grazing and grass silage intake, or (2) concentrate, maize and other silages and hay and straw intake, respectively, as well as associated milk quality parameters (Fig. 5).

A separate RDA was carried out in which data from conventional farms that used high-grazing-based feeding regimens (which conformed with organic feed regulations) were included as an additional passive driver (low-input conventional) (online Supplementary Fig. S34). Organic and low-input conventional systems are in a very similar position on the bi-plot, suggesting that they have a very similar impact on milk composition (Fig. 5).

## Discussion

### Milk yields in organic and conventional dairy production systems

The meta-analysis results showing that milk yields per cow were on average 20 % lower in organic compared with conventional systems confirms results from a previous meta-analysis^(^ ^13^ ^)^, which linked lower yields per cow to the use of high grazing/conserved forage diets used in organic dairy systems. This confirms previous studies that reported that grazing-based diets result in lower yield per cow than the higher-concentrate diets typically used in high-input conventional dairy production^(^ ^27^ ^,^ ^30^ ^–^ ^34^ ^,^ ^48^ ^,^ ^53^ ^)^. However, the study of Palupi *et al*.^(^ ^13^ ^)^ also reported higher total fat and protein content for organic milk, whereas the meta-analysis reported here found no significant difference in total fat and protein content between organic and conventional milk.

### Composition of milk from organic and conventional dairy production systems

#### Fatty acid composition

Results of the meta-analyses reported here showed that organic milk had a similar total SFA and MUFA content, but higher concentrations of total PUFA and *n*-3 PUFA compared with conventional milk, which is broadly consistent with results from three previous meta-analyses^(^ ^10^ ^,^ ^13^ ^,^ ^14^ ^)^.

The findings of higher concentrations of (1) individual *n*-3 PUFA (ALA, EPA and DPA), (2) VA, (3) CLA9 and higher *n*-3:*n*-6 ratios in organic milk in this study are also consistent with results reported by Palupi *et al*.^(^ ^13^ ^)^. Dangour *et al*.^(^ ^10^ ^)^ and Smith-Spangler *et al*.^(^ ^14^ ^)^ did not publish meta-analysis results for individual *n*-3 PUFA, CLA9 and *n*-3:*n*-6 or *n*-6:*n*-3 ratios in milk, but the higher VA concentrations in organic milk were also confirmed by Smith-Spangler *et al*.^(^ ^14^ ^)^.

Palupi *et al*.^(^ ^13^ ^)^ also detected significantly lower concentrations of total *n*-6 PUFA, LA and OA (the main MUFA in milk). For these parameters, no significant difference was detected in the meta-analyses reported here.

Sensitivity analyses showed that for most of the FA composition parameters discussed above the method of data synthesis did not have a large effect on results, in terms of both statistical significance and the magnitude of difference between organic and conventional milk. This indicates that there is now a sufficiently large body of published information on the FA composition of organic milk to identify substantive differences across study types and pedo-climatic and agronomic environments. It also increases confidence in conclusions drawn regarding potential nutritional impacts of switching from conventional to organic milk consumption (see also below).

RDA of data from a large cross-European farm and milk quality survey identified contrasting feeding regimens (especially the proportion of grazing, concentrate and conserved forage in the diet) used in organic and conventional production systems as the main drivers for differences in milk fat and antioxidant profiles. Most importantly, RDA results indicate that high fresh forage intakes by grazing animals (as prescribed by organic farming standards) increase concentrations of nutritionally desirable FA (e.g. PUFA, MUFA, *n*-3 PUFA, ALA, *cis*-9,*trans*-11-CLA) and antioxidants/vitamins (except for synthetic 2R stereoisomers of *α*-tocopherol) in milk, whereas high concentrate intakes have an opposite effect. Results from the RDA also indicated that high intakes of concentrate (and to a lesser extent grass and maize silages) increase concentrations of total *n*-6 FA, LA and AA in milk. When included as a passive driver in the RDA, the alignment of ‘organic management’ with grazing intake and conserved forage feeding and ‘conventional management’ with concentrate intake and vitamin supplementation further supports the conclusion that contrasting feeding regimens are the main reason for the composition differences between organic and conventional milk.

These results are consistent with the findings of a wide range of experimental studies that investigated contrasting dairy cow diets on rumen biohydrogenation and other processes influencing milk fat composition and demonstrated the benefits of high-forage diets on milk fat quality (e.g. concentrations of beneficial PUFA and antioxidants)^(^ ^53^ ^–^ ^56^ ^)^. A recent Norwegian study also showed that management and botanical composition of grassland significantly affects the *n*-3 PUFA concentration in milk from organic but not conventional farms^(^ ^57^ ^)^. It is also interesting to note that models to predict milk FA profiles, based on farming practice, especially feeding regimens, have recently been developed using data collected in on-farm surveys^(^ ^56^ ^)^.

The fat concentrations and FA profiles in milk from small ruminants (goats and sheep) and buffalo are known to differ from those of bovine milk^(^ ^58^ ^)^, and available data for goat, sheep and buffalo milk were therefore not pooled with data for bovine milk in meta-analyses. However, when comparative composition data for milk from small ruminants (sheep and goats) and buffalo were pooled, it was possible to carry out meta-analyses for certain fat composition parameters (e.g. total MUFA and PUFA, VA, CLA9 and LA). Although these showed some composition difference (e.g. higher CLA and ALA concentrations in organic milk) similar to those detected for bovine milk, there were also some differences (e.g. higher MUFA and lower LA concentrations in organic milk). Additional and more substantial comparative studies for non-bovine milk are therefore required to confirm results, before conclusions can be drawn as to potential health impacts of switching to organic milk and dairy products from small ruminants and buffalo.

There were insufficient published comparative data to carry out robust meta-analysis for FA concentrations in processed dairy products (e.g. fermented milk, yoghurt, cheese, curd, butter and whey). However, results in the small number of studies available showed similar trends to those found for milk for a range of fat composition parameters including for total *n*-3 PUFA, VLC *n*-3 PUFA and CLA9. This is not surprising, as previous studies suggest that processing has no or only a small impact on FA profiles in milk^(^ ^27^ ^)^.

#### Antioxidant/vitamin and minerals

Results indicated that organic milk has higher concentrations of *α*-tocopherol, which is consistent with the results of the only one previous meta-analysis comparing *α*-tocopherol concentrations in bovine milk^(^ ^13^ ^)^. A study from the UK in which concentrations of different stereoisomers of *α*-tocopherol were compared in organic and conventional milk indicated that this is because of 3R *α*-tocopherol (the dominant stereoisomer found in bovine milk) concentration being higher in organic milk, whereas concentration of the 2R stereoisomers were similar in organic and conventional milk^(^ ^30^ ^)^. This is not surprising, as (1) organic farming standards prescribe high intakes of fresh forage, which is the main, natural source for *α*-tocopherol in the dairy diet and nearly exclusively contains 3R stereoisomers of *α*-tocopherol; and (2) 2R stereoisomers are only found in synthetic vitamin E supplements, which are widely used in conventional dairy production, but prohibited under organic farming standards^(^ ^27^ ^,^ ^30^ ^)^. However, it should be pointed out that in some European countries (e.g. the Nordic countries) organic farmers can obtain derogations to use synthetic vitamins, especially during the winter indoor period^(^ ^13^ ^,^ ^30^ ^)^. Sensitivity analysis showed that the method of data synthesis did not have a large effect on results, in terms of both statistical significance and the magnitude of difference between organic and conventional milk.

Not surprisingly, RDA identified vitamin supplements as a strong driver for increased concentration of the 2R stereoisomers of *α*-tocopherol in milk, as the synthetic vitamin E in supplements contains a high proportion of the 2R stereoisomers^(^ ^30^ ^)^. In contrast, RDA identified fresh forage intake as a strong driver for concentrations of 3R stereoisomers of *α*-tocopherol and carotenoids in milk. The RDA therefore supports the findings of the meta-analyses, one other review/meta-analysis^(^ ^13^ ^)^ and a previous UK study^(^ ^30^ ^)^, which concluded that higher intake of natural *α*-tocopherol and carotenoids from fresh forage in organic dairy systems more than compensates for synthetic vitamin supplementation in conventional systems with respect to vitamin concentrations in milk.

The finding of lower I and Se concentrations in organic milk are more surprising, as mineral supplementation is permitted under organic farming standards, if necessary, and is widely used in both organic and conventional dairy productions, as they were shown to improve animal health^(^ ^59^ ^,^ ^60^ ^)^. There are published data on the relative use of mineral and I supplements in organic and conventional systems. However, the amounts of I supplements used in organic dairy systems is likely to be lower^(^ ^61^ ^)^ (P. Melchett, Soil Association, personal communication) than in conventional farming systems. This is may be due to (1) organic systems using less concentrate feeds, (2) mineral supplementation having to be specifically requested by farmers for organic feeds in many countries (whereas mineral supplements are routinely added to conventional concentrate feeds) and/or (3) the use of I teat disinfection (which is known to significantly increase I concentrations in milk^(^ ^59^ ^)^) being less common in organic production. I in milk is known to fluctuate seasonally^(^ ^62^ ^)^, reflecting greater supplementation of dairy cows in winter compared with summer. It is also strongly influenced by proximity to the sea, as I is deposited from marine evaporation, and can be lost during processing with high-temperature pasteurisation^(^ ^59^ ^)^. However, publications reporting comparative data on I concentrations provide insufficient information on the location, teat disinfection methods and details of mineral supplements used on farms that produced the milk samples, and it therefore remains unclear to what extent these factors affected the results of the meta-analyses. Although the I content of organic milk was significantly lower, concentrations in both organic (147 μg/l) and conventional (248 μg/l) milk fall within the range reported in a review of European farm surveys by Flachowsky *et al*.^(^ ^59^ ^)^ which suggested that current I concentrations in milk may be too high in animals receiving high levels of feed I. For this reason EFSA have proposed a reduction in the permitted levels of I in dairy cattle feed from 5 to 2 mg I/kg feed^(^ ^63^ ^)^. However, it should be pointed out that the I requirement in pregnant and breast-feeding women is higher (250 μg/d) than in other adults (150 μg/d)^(^ ^64^ ^)^. As dairy products are a major source of I, low levels of dairy consumption in these groups is therefore more likely to result in deficiency with organic dairy products, especially if I intakes are not increased by other means (e.g. consumption of fish, shellfish, I-fortified table salt or I supplements).

Se concentrations in milk reflect the Se intake by lactating cows, from that naturally occurring in their feed (largely dependent on soil Se status) and that added as supplements^(^ ^62^ ^)^. Although results of the meta-analysis show concentrations of Se in organic milk to be slightly but significantly lower than conventional milk, mean values for both fall between levels reported for milk from USA (considered to have a high Se status) and Norway (considered to be low in Se)^(^ ^62^ ^)^. Apart from mineral supplements, contrasting conditions (Se concentrations, fertilisation regimens and soil pH) and their impact on Se concentrations in forage and concentrate feeds may also contribute to the difference in Se concentrations between organic and conventional milk. For example, in Finland, mineral N fertiliser is supplemented with Se to compensate for the low Se concentrations in Finnish soils; however, as mineral N fertilisers are not permitted under organic farming standards, contrasting fertilisation regimens may at least partially explain differences in Se content of organic and conventional milk^(^ ^65^ ^)^.

The finding of marginally higher concentration of Fe in organic compared with conventional milk is largely inconsequential, as milk is widely recognised as a relatively poor source of dietary Fe^(^ ^66^ ^)^.

Mineral composition was not determined in the cross-European dairy management and milk yield and quality survey used from RDA. It would therefore be important to carry out mineral composition surveys across regions with different pedo-climatic conditions and dairy management practices to identify the main drivers for mineral composition in both organic and conventional dairy production.

Mineral supplementation standards and guidelines are currently reviewed by organic sector bodies and certification organisations; there is an ongoing R&D programme to evaluate strategies available for raising concentrations of certain minerals in UK organic milk (especially I and Se) and associate benefits and risks^(^ ^67^ ^)^. There are well-established relatively inexpensive sustainable methods (e.g. increased use of mineral supplement, use of I teat disinfectants, use of Se-fortified organic fertilisers or sustainably sourced seaweeds) to increase both I and Se concentrations, but the main challenge with both minerals is that both inadequate and excessive supply have negative health impacts and that the amounts for adequate and excessive supply are close^(^ ^59^ ^,^ ^65^ ^)^ (see also section on ‘Potential nutritional impacts of composition differences’).

### Potential nutritional impacts of composition differences

#### Dietary *n*-3 PUFA intakes

Adequate intakes (AI) for PUFA recommended for adults by the EFSA are 4–8 % of energy intake for LA, 0·5–0·75 % of energy intake for ALA and 250–550 mg/d for EPA+DHA^(^ ^49^ ^,^ ^68^ ^)^. EFSA also recommended an additional 100–200 mg/d DHA intake during pregnancy and lactation^(^ ^49^ ^,^ ^68^ ^)^. Current estimated mean intakes are known to be too high for LA, match AI recommendations for ALA, but reach less than half the AI for VLC *n*-3 PUFA^(^ ^49^ ^,^ ^68^ ^)^. North American and European agencies currently advise consumers to increase fish and especially oily fish (e.g. salmon and herring) consumption to improve VLC *n*-3 PUFA intake and reduce CVD risk^(^ ^69^ ^)^. Unfortunately, implementing these recommendation of higher fish consumption widely across the human population is thought to be impossible, as most of the world’s fish stocks are already fully or over-exploited. In addition, concerns about the sustainability/environmental impacts of fish farming, Hg/dioxin contamination levels in oil-rich fish in some regions of the world and recent studies linking very high intakes of oily fish/fish oil supplements with an increased prostate cancer risk^(^ ^69^ ^–^ ^71^ ^)^ cast further doubt on this approach. It is therefore thought to be essential to develop additional/complementary dietary approaches to increase long-chain *n*-3 FA supply in line with current AI recommendations.

On the basis of the meta-analyses results, concentrations of VLC *n*-3 PUFA were estimated to be 58 % higher in organic compared with conventional milk, and a switch from conventional to organic milk and dairy consumption could therefore be one such complementary dietary approach, especially as recent studies indicate that processing of milk into high-fat products such as butter and cheese (which account for a high proportion of milk fat intake) does not change the fat composition and the relative difference in *n*-3 PUFA between organic and conventional dairy products^(^ ^27^ ^,^ ^72^ ^)^. For example, consumption of half a litre of full-fat milk (or equivalent fat intakes with dairy products) can be estimated to provide 34 and 22 % of the actual and 16 % (39 mg) and 11 % (25 mg) of the recommended daily VLC *n*-3 PUFA intake with organic and conventional milk consumption, respectively.

The estimated additional VLC *n*-3 PUFA intake with organic milk does not take into account potential increases in the ALA to EPA conversion rates associated with the lower LA:ALA ratio in organic milk/dairy products (discussed below) and the relative capacity of individuals to convert/elongate ALA into longer-chain *n*-3 PUFA^(^ ^73^ ^–^ ^75^ ^)^. However, it should be pointed out that there is still considerable scientific uncertainty about the effect of LA intake on ALA to VLC *n*-3 conversion^(^ ^69^ ^,^ ^73^ ^–^ ^80^ ^)^.

#### Dietary *n*-6:*n*-3 and linoleic acid:*α*-linolenic acid ratios

It has been suggested that dietary intake of *n*-6 (especially LA) relative to *n*-3 FA is too high in typical Western European diets^(^ ^81^ ^)^; estimates for *n*-6:*n*-3 PUFA ratios are between 12:1 and 15:1, and for some individuals they are as high as 40:1^(^ ^49^ ^,^ ^68^ ^,^ ^82^ ^)^. Current recommendations are to achieve an *n*-6:*n*-3 ratio between 4:1 and 1:1^(^ ^83^ ^)^. Reductions in total *n*-6 and LA intake have been suggested because LA is the precursor of the pro-inflammatory FA AA and stimulates adipogenesis (and thereby the risk of obesity) to a greater extent than *n*-3 FA^(^ ^81^ ^)^. In addition, excessive LA intakes during pregnancy and the first years of life have been linked to a range of neurodevelopmental deficits and abnormalities^(^ ^84^ ^)^, and there is evidence that high *n*-6:*n*-3 PUFA and LA:ALA ratios in the diet increases the risk of a range of other chronic diseases including certain cancers, inflammatory and autoimmune diseases, and CVD^(^ ^49^ ^,^ ^68^ ^)^.

However, it is difficult to estimate to what extent the differences in FA profiles may affect human health, as there are only a small number of studies in which health impacts of switching from organic to conventional milk consumption were studied. One study focused on the effect of organic milk consumption on eczema in children under 2 years in the Netherlands (a country with relatively high milk consumption)^(^ ^85^ ^)^. It reported that eczema was significantly lower in children from families consuming organic rather than conventional milk. This may have been because of the higher *n*-3 PUFA concentrations and lower *n*-6:*n*-3 PUFA ratio in organic milk, as there is increasing evidence for anti-allergenic effects of *n*-3 FA^(^ ^76^ ^)^. For example, a recent animal study showed that increasing dietary VLC *n*-3 PUFA intake prevented allergic sensitisation to cows’ milk protein in mice^(^ ^77^ ^)^. Two other cohort studies (one in Denmark and one in Norway) investigated associations between milk/dairy product consumption during pregnancy and the incidence of hypospadias, the most common genital birth defect in boys^(^ ^86^ ^,^ ^87^ ^)^. The Danish study found that ‘frequent consumption of high-fat dairy products (milk, butter) while rarely or never choosing the organic alternative to these products during pregnancy was associated with increased odds of hypospadia’^(^ ^86^ ^)^. The more recent Norwegian study confirmed these results and reported that (1) organic food consumption was associated with lower odds of hypospadia, and (2) the closest associations were found with organic vegetable and milk/dairy product consumption^(^ ^87^ ^)^.

#### Conjugated linoleic acid

Milk and dairy products account for up to 67 % of total dietary CLA intake, as CLA is only found in ruminant fat^(^ ^88^ ^)^. Organic milk was found to have 39 % higher concentrations of CLA than conventional milk, but it also had 46 % higher concentrations of VA, which is converted to CLA by human desaturase enzymes. Thus, the potential increase in CLA supply with organic dairy consumption may be even higher^(^ ^31^ ^–^ ^33^ ^,^ ^88^ ^)^. CLA has been linked to anti-obesity, anti-diabetogenic, anti-carcinogenic and other potential health benefits. However, most evidence for beneficial health impacts of CLA consumption is from *in vitro* and animal studies in which diets were supplemented with synthetic CLA, and human dietary intervention studies often did not detect significant effects of increasing CLA intake^(^ ^21^ ^,^ ^22^ ^)^. As a result, there is still controversy about the exact health impacts of increased CLA intake in humans and the dose/intake levels required to demonstrate beneficial effects^(^ ^22^ ^)^.

A recent meta-analysis of eighteen human studies concluded that CLA supplementation produces a modest weight loss in humans, when very high doses of synthetic CLA (approximately 3·2 g/d) were used^(^ ^89^ ^)^. However, it is also important to point out that most *in vitro*, and both animal and human dietary intervention, studies were carried out using synthetic CLA, which has a different CLA isomer balance to the naturally occurring CLA found in milk^(^ ^30^ ^,^ ^31^ ^)^. As CLA isomers differ in their biological activity, results from animal and human dietary intervention studies based on synthetic CLA may not reflect the physiological effects of increasing CLA intake via a switch to organic milk consumption. For example, anti-obesity effects were mainly linked to CLA10 (*trans*-10-*cis*-12-18 : 2), which makes up 50 % of synthetic CLA^(^ ^21^ ^,^ ^22^ ^)^. In contrast, CLA in milk is over 80 % CLA9 (*cis*-9-*trans*-11-18 : 2), with CLA10 accounting for <10 % of total CLA^(^ ^30^ ^,^ ^31^ ^)^.

To our knowledge, no animal or human dietary intervention studies in which the effect of increasing CLA intake via milk and dairy products with a higher CLA content (e.g. organic milk) have been carried out, and until such studies have been completed it is not possible to estimate potential health impacts of increasing CLA consumption via switching to organic milk consumption.

### Antioxidants/vitamins and minerals

#### Antioxidants/vitamins

Increased dietary intakes of fat-soluble vitamins/antioxidants such as carotenoids and *α*-tocopherol are thought to be nutritionally desirable. Increased antioxidant intake has been shown to reduce oxidative stress, a known risk factor in a range of chronic health conditions such as CVD, certain cancers and reduced immune status^(^ ^90^ ^)^. However, as dairy products are not major sources of vitamin E and carotenoids in the human diet, it is unlikely that the slightly higher *α*-tocopherol concentrations found in organic milk will have a major health impact in humans.

#### Iodine

The daily recommended intake for I in UK is 140 µg/d^(^ ^91^ ^)^. Milk and dairy products are important dietary sources for I, and they have been reported to supply 30–60 % of intake^(^ ^59^ ^)^. On the basis of the results from the meta-analyses, a daily consumption of half a litre of milk is therefore estimated to provide 53 and 88 % of daily I intake from organic and conventional milk, respectively. At this level of milk/dairy consumption, both organic and conventional products would be expected to provide adequate but not excessive intakes.

Although there is a focus on overcoming I deficiency in some countries and sectors of society^(^ ^92^ ^,^ ^93^ ^)^, there is also concern that excessive concentrations of I in milk and dairy products could result in thyrotoxicosis and other adverse health effects in both livestock and humans^(^ ^94^ ^–^ ^96^ ^)^. This apparent contradiction arises from a combination of (1) the relatively narrow margin between dietary I deficiency (<140 μg/d) and excess (>500 μg/d), (2) the wide range in I concentrations found in milk and (3) variation in milk and dairy consumption. I intakes from both organic and conventional milk could be excessive in regions with very high milk and dairy consumption, such as Finland, Sweden and the Netherlands, where average daily consumption of milk is close to 1 litre/d^(^ ^97^ ^)^. A recent review on I also suggests that the widespread use of I as a teat disinfectant and high I supplementation of livestock feeds has led to excessive dietary intakes of I and negative effects on human health in some regions of the world (e.g. North America) and highlight recent recommendations to reduce permitted levels of I supplementation for livestock^(^ ^59^ ^)^. The lower I levels from organic production systems could therefore be considered beneficial and may soon be matched in conventional dairy production^(^ ^59^ ^)^.

On the other hand, it has also been suggested that a lower I content in organic milk could result in deficiency in population groups with a higher demand of I (e.g. pregnant, nursing and young women), low dairy consumption and/or insufficient supply of I from other foods^(^ ^98^ ^,^ ^99^ ^)^. However, it may not be sensible to strive to raise I levels in milk to accommodate population groups with a high I requirement or low dairy consumption, as this increases the risk of excessive intakes by population groups with an average I need and/or high milk consumption. Adjusting dairy I supplementation and concentrations in milk to meet ‘average’ or ‘slightly below average’ needs of consumers is thought to be a better strategy, as it (1) reduces the health risks from excessive supply for consumers with high dairy intakes and (2) is relatively easy for individuals with a high I demand and/or low dairy intake to raise their I intake to satisfactory levels via mineral supplements and/or the use of I-fortified table salt^(^ ^94^ ^,^ ^98^ ^,^ ^99^ ^)^.

#### Selenium

Se concentrations in animal feed and foods are increasingly recognised as being too low in many regions of the world. Insufficient Se supply was more frequently associated with livestock rather than human diets and can impair immune and antioxidant status^(^ ^62^ ^,^ ^66^ ^)^. Milk and dairy products are one source for Se in the human diet^(^ ^65^ ^)^, and results from the meta-analysis show lower concentrations of Se in organic compared with conventional milk. However, switching from conventional to organic milk/dairy product consumption is unlikely to have a major effect on Se intake, especially in regions with low to moderate dairy consumption. On the basis of UK nutrient requirements^(^ ^91^ ^)^, it can be estimated that consumption of half a litre of milk will be equivalent to 11 and 13 % of recommended intakes with organic and conventional milk/dairy products, respectively.

#### Iron

Different from meat, milk is not a major source of Fe in the human diet^(^ ^100^ ^)^. The slightly higher Fe intake with organic milk is therefore unlikely to have a major nutritional impact.

The need to optimise mineral supply in dairy production (especially with respect to Se) should be considered in future revisions of organic farming regulations for mineral supplementation of livestock and fortification of processed foods.

### Strength of evidence and exploration of heterogeneity

Risk of bias of individual studies was generally high and not universally mitigated by large effects. Publication bias was also strongly suspected for many outcomes. Overall strength of evidence was variable, but was judged as moderate for the primary outcomes (Table 1). Thus, some uncertainty surrounds the conclusions of this work, largely arising from poor reporting in the primary literature. We also speculate on the widespread problem of selective reporting, although this was not formally evaluated.

The finding of significant differences between countries/geographic regions, as well as production systems, is consistent with previous studies that explained similar findings with contrasting dairy management regimens being used for organic and/or conventional systems (e.g. length of outdoor grazing period, dietary regimens and breed choice/selection) between countries/regions^(^ ^27^ ^)^. Differences in dairy management practices are therefore thought to be a major source of variation. However, meta-regressions are subject to bias and confounding. Here, additional variation was likely because of pooling data across experimental approaches (retail surveys, farm surveys and experimental studies) in the meta-analyses, although there were no substantial differences in the results obtained with different experimental approaches. Other confounding factors cannot be discounted.

### The need to carry out dietary intervention and cohort studies

Overall, it can be concluded that a switch from intensive conventional to organic production standards will result in substantive improvements in milk fat composition, especially in the supply of nutritionally desirable VLC *n*-3 PUFA. Potential impacts of composition differences on human health currently have to be extrapolated from existing information about the effects of compounds such as VLC *n*-3 PUFA, the *n*-3:*n*-6 PUFA ratio, CLA, antioxidants/vitamins and minerals on human health, as there are virtually no studies in which impacts of organic food consumption on animal or human health or health-related biomarkers were assessed. However, the significant differences in nutritionally relevant compounds identified by the meta-analyses reported here demonstrate the need to carry out human dietary intervention and cohort studies designed to quantify the health impact of switching to milk and dairy products from organic or other ‘low-input’ grazing-based livestock production systems that deliver similar composition changes.

The argument for more rigorous human intervention studies to confirm health benefits is supported by recent human cohort studies, which suggest that a switch to organic milk consumption may reduce the risk of hypospadias in boys^(^ ^86^ ^,^ ^87^ ^)^ and eczema in children under 2 years of age^(^ ^85^ ^)^. Clearly, additional dietary intervention and cohort studies should be carried out to identify/quantify other potential human health impacts of switching to organic milk and dairy product consumption.

## Supplementary material

For supplementary material accompanying this paper visit http://dx.doi.org/10.1017/S0007114516000349. click here to view supplementary material
