# Supplementary material for: Higher PUFA and n-3 PUFA, conjugated linoleic acid, α-tocopherol and iron, but lower iodine and selenium concentrations in organic milk: a systematic literature review and meta- and redundancy analyses
Source: Br J Nutr. 2016 Mar 28;115(6):1043–60. doi: 10.1017/S0007114516000349 (PMC4838834; doi:10.1017/S0007114516000349)
Supplement: Supplementary file 1 [file S0007114516000349sup.zip › S0007114516000349sup001.docx]

Higher PUFA and omega-3 PUFA, CLA, α-tocopherol and iron, but lower iodine and selenium concentrations in organic milk: A Systematic Literature Review and Meta- and Redundancy Analyses

APPENDIX

Table of Contents

[**Table A1**. Results of meta-analysis comparing composition of organic (ORG) vs conventional (CONV) bovine milk using standard meta-analysis and sensitivity analyses protocols 1-7 2](#_Toc426549160)

[**Table A2**. Standard meta-analysis results after exclusion of 20% of studies with the least precise treatment effects (sensitivity anlaysis 8, see main article and online supplementary Table S5 for details) for parameters shown in Fig. 3 and 4 of the main paper. 18](#_Toc426549161)

| Table A1. Results of meta-analysis comparing composition of organic (ORG) vs conventional (CONV) bovine milk using standard meta-analysis and sensitivity analyses protocols 1-7**.** | | | | | | | | | | | | | | |
| --- | --- | --- | --- | --- | --- | --- | --- | --- | --- | --- | --- | --- | --- | --- |
|  |  | **Weighted meta-analysis** | | | | | | |  | **Unweighted meta-analysis** | | | | |
| **Parameter** | **an*** | ***n*** | **SMD** | **95% CI** | ***P***† | **Heterogen.**‡ | **MPD**§ | **95% CI** |  | ***n*** | **Ln ratio**\|\| | ***P***† | **MPD**§ | **95% CI** |
| *Major components* | | | |  |  |  |  |  |  |  |  |  |  |  |
| Fat | std,1 | 31 | -0.29 | -0.63, 0.05 | 0.092 | Yes (85%) | -1.37 | -3.66, 0.91 |  | 58 | 4.60 | 0.329 | -0.45 | -2.32, 1.43 |
|  | 2,3 | 31 | -0.35 | -0.66, -0.04 | 0.028 | Yes (85%) | -1.66 | -3.87, 0.55 |  | 74 | 4.60 | 0.127 | -0.86 | -2.34, 0.62 |
|  | 4,5 | 36 | -0.21 | -0.51, 0.09 | 0.174 | Yes (84%) | -1.13 | -3.19, 0.93 |  | 71 | 4.60 | 0.148 | -0.96 | -2.68, 0.75 |
|  | 6,7 | 36 | -0.26 | -0.54, 0.02 | 0.068 | Yes (84%) | -1.38 | -3.38, 0.62 |  | 87 | 4.59 | 0.045 | -1.22 | -2.62, 0.19 |
| Protein | std,1 | 29 | -0.17 | -0.55, 0.21 | 0.368 | Yes (88%) | -0.24 | -1.80, 1.33 |  | 56 | 4.60 | 0.146 | -0.64 | -1.84, 0.56 |
|  | 2,3 | 29 | -0.21 | -0.58, 0.16 | 0.272 | Yes (90%) | -0.34 | -1.91, 1.23 |  | 72 | 4.59 | 0.008 | -1.18 | -2.16, -0.21 |
|  | 4,5 | 33 | -0.13 | -0.47, 0.20 | 0.438 | Yes (87%) | -0.31 | -1.75, 1.12 |  | 69 | 4.60 | 0.077 | -0.75 | -1.78, 0.27 |
|  | 6,7 | 33 | -0.16 | -0.49, 0.17 | 0.335 | Yes (89%) | -0.40 | -1.84, 1.04 |  | 85 | 4.59 | 0.003 | -1.19 | -2.06, -0.33 |
| Solids | std,1 | 8 | 0.64 | -0.23, 1.52 | 0.149 | Yes (86%) | 1.05 | -0.45, 2.55 |  | 13 | 4.62 | 0.022 | 1.50 | 0.11, 2.89 |
|  | 2,3 | 8 | 0.64 | -0.23, 1.52 | 0.149 | Yes (86%) | 1.05 | -0.45, 2.55 |  | 13 | 4.62 | 0.021 | 1.50 | 0.11, 2.89 |
|  | 4,5 | 9 | 0.57 | -0.18, 1.31 | 0.135 | Yes (90%) | 0.97 | -0.36, 2.30 |  | 14 | 4.62 | 0.020 | 1.41 | 0.12, 2.71 |
|  | 6,7 | 9 | 0.57 | -0.18, 1.31 | 0.135 | Yes (90%) | 0.97 | -0.36, 2.30 |  | 14 | 4.62 | 0.020 | 1.41 | 0.12, 2.71 |
| Solids (no-fat) | std,1 | 4 | 0.24 | -0.03, 0.51 | 0.083 | Yes (0%) | 1.37 | -0.75, 3.49 |  | 7 | 4.62 | 0.094 | 1.08 | -0.30, 2.47 |
|  | 2,3 | 4 | 0.24 | -0.03, 0.51 | 0.083 | Yes (0%) | 1.37 | -0.75, 3.49 |  | 7 | 4.62 | 0.092 | 1.08 | -0.30, 2.47 |
|  | 4,5 | 5 | 0.23 | 0.03, 0.44 | 0.024 | Yes (0%) | 1.21 | -0.46, 2.88 |  | 8 | 4.62 | 0.074 | 1.02 | -0.19, 2.22 |
|  | 6,7 | 5 | 0.23 | 0.03, 0.44 | 0.024 | Yes (0%) | 1.21 | -0.46, 2.88 |  | 8 | 4.62 | 0.077 | 1.02 | -0.19, 2.22 |
| n, number of data points included in the comparison; MPD, mean percent difference; SMD, standardised mean difference of random-effect model. *Sensitivity analysis number: std – standard meta-analysis; 1-6 – sensitivity analysis 1 to 6 (see main article and online supplementary Table S5 for details); †*P* value <0.05 indicates significance of the difference in composition between organic and conventional milk; ‡Heterogeneity and the I^2^ Statistic; §Magnitude of difference between organic and conventional samples (value <0 indicate higher concentration in CONV, value >0 indicate higher concentration in ORG); \|\|Ln ratio = Ln(ORG/CONV × 100%). | | | | | | | | | | | | | | |

| **Table A1 cont.** Results of meta-analysis comparing composition of organic (ORG) vs conventional (CONV) bovine milk using standatd meta-analysis and sensitivity analyses protocols 1-7. | | | | | | | | | | | | | | |
| --- | --- | --- | --- | --- | --- | --- | --- | --- | --- | --- | --- | --- | --- | --- |
|  |  | **Weighted meta-analysis** | | | | | | |  | **Unweighted meta-analysis** | | | | |
| **Parameter** | **an*** | ***n*** | **SMD** | **95% CI** | ***P***† | **Heterogen.**‡ | **MPD**§ | **95% CI** |  | ***n*** | **Ln ratio**\|\| | ***P***† | **MPD**§ | **95% CI** |
| *Fatty acids* | | | |  |  |  |  |  |  |  |  |  |  |  |
| Free fatty acids | std,1 | - | - | - | - | - | - | - |  | 3 | 4.55 | 0.247 | -5.91 | -13.97, 2.15 |
|  | 2,3 | - | - | - | - | - | - | - |  | 3 | 4.55 | 0.242 | -5.91 | -13.97, 2.15 |
|  | 4,5 | - | - | - | - | - | - | - |  | 9 | 4.45 | 0.007 | -17.92 | -30.10, -5.74 |
|  | 6,7 | - | - | - | - | - | - | - |  | 9 | 4.45 | 0.007 | -17.92 | -30.10, -5.74 |
| SFA | std,1 | 19 | -0.17 | -0.66, 0.31 | 0.477 | Yes (72%) | -0.69 | -2.24, 0.86 |  | 33 | 4.60 | 0.096 | -0.80 | -1.96, 0.37 |
|  | 2,3 | 19 | -0.17 | -0.66, 0.31 | 0.477 | Yes (72%) | -0.69 | -2.24, 0.86 |  | 32 | 4.60 | 0.102 | -0.79 | -1.99, 0.42 |
|  | 4,5 | 21 | -0.15 | -0.56, 0.26 | 0.472 | Yes (64%) | -0.58 | -2.00, 0.84 |  | 46 | 4.60 | 0.116 | -0.58 | -1.52, 0.37 |
|  | 6,7 | 21 | -0.15 | -0.56, 0.26 | 0.472 | Yes (64%) | -0.58 | -2.00, 0.84 |  | 45 | 4.60 | 0.129 | -0.56 | -1.53, 0.40 |
| 8:0 | std,1 | 9 | -0.03 | -0.64, 0.59 | 0.936 | Yes (81%) | -1.44 | -7.56, 4.68 |  | 16 | 4.64 | 0.123 | 3.64 | -2.42, 9.70 |
| (caprylic acid) | 2,3 | 9 | -0.03 | -0.64, 0.59 | 0.936 | Yes (81%) | -1.44 | -7.56, 4.68 |  | 15 | 4.63 | 0.180 | 3.24 | -3.19, 9.66 |
|  | 4,5 | 10 | 0.14 | -0.48, 0.76 | 0.661 | Yes (88%) | -0.15 | -6.18, 5.88 |  | 21 | 4.65 | 0.031 | 5.20 | -0.05, 10.44 |
|  | 6,7 | 10 | 0.14 | -0.48, 0.76 | 0.661 | Yes (88%) | -0.15 | -6.18, 5.88 |  | 20 | 4.65 | 0.041 | 4.97 | -0.52, 10.46 |
| 12:0 | std,1 | 11 | 0.18 | -1.39, 1.75 | 0.820 | Yes (98%) | -3.59 | -10.22, 3.03 |  | 17 | 4.59 | 0.284 | -1.98 | -8.12, 4.16 |
| (lauric acid) | 2,3 | 12 | 0.05 | -1.34, 1.44 | 0.943 | Yes (97%) | -3.81 | -9.90, 2.28 |  | 18 | 4.59 | 0.247 | -2.21 | -8.03, 3.61 |
|  | 4,5 | 12 | 0.29 | -1.12, 1.70 | 0.685 | Yes (98%) | -2.09 | -8.82, 4.64 |  | 25 | 4.61 | 0.439 | 0.25 | -4.56, 5.05 |
|  | 6,7 | 13 | 0.17 | -1.10, 1.44 | 0.796 | Yes (98%) | -2.40 | -8.64, 3.84 |  | 26 | 4.61 | 0.487 | 0.00 | -4.65, 4.65 |
| n, number of data points included in the comparison; MPD, mean percent difference; SMD, standardised mean difference of random-effect model; SFA, saturated fatty acids. *Sensitivity analysis number: std – standard meta-analysis; 1-6 – sensitivity analysis 1 to 6 (see main article and online supplementary Table S5 for details); †*P* value <0.05 indicates significance of the difference in composition between organic and conventional milk; ‡Heterogeneity and the I^2^ Statistic; §Magnitude of difference between organic and conventional samples (value <0 indicate higher concentration in CONV, value >0 indicate higher concentration in ORG); \|\|Ln ratio = Ln(ORG/CONV × 100%). | | | | | | | | | | | | | | |

| **Table A1 cont.** Results of meta-analysis comparing composition of organic (ORG) vs conventional (CONV) bovine milk using standard meta-analysis and sensitivity analyses protocols 1-7. | | | | | | | | | | | | | | |
| --- | --- | --- | --- | --- | --- | --- | --- | --- | --- | --- | --- | --- | --- | --- |
|  |  | **Weighted meta-analysis** | | | | | | |  | **Unweighted meta-analysis** | | | | |
| **Parameter** | **an*** | ***n*** | **SMD** | **95% CI** | ***P***† | **Heterogen.**‡ | **MPD**§ | **95% CI** |  | ***n*** | **Ln ratio**\|\| | ***P***† | **MPD**§ | **95% CI** |
| 14:0 | std,1 | 12 | 0.32 | -0.42, 1.05 | 0.398 | Yes (88%) | 1.02 | -2.60, 4.63 |  | 18 | 4.62 | 0.172 | 1.57 | -1.60, 4.74 |
| (myristic acid) | 2,3 | 13 | 0.34 | -0.32, 0.99 | 0.318 | Yes (86%) | 1.10 | -2.24, 4.44 |  | 19 | 4.62 | 0.159 | 1.60 | -1.41, 4.60 |
|  | 4,5 | 14 | 0.62 | -0.14, 1.38 | 0.110 | Yes (92%) | 2.42 | -1.20, 6.05 |  | 27 | 4.63 | 0.017 | 2.83 | 0.31, 5.35 |
|  | 6,7 | 15 | 0.61 | -0.08, 1.30 | 0.083 | Yes (91%) | 2.40 | -0.98, 5.78 |  | 28 | 4.63 | 0.018 | 2.80 | 0.37, 5.24 |
| 15:0 | std,1 | 8 | 1.61 | -0.39, 3.60 | 0.115 | Yes (98%) | 7.15 | -0.26, 14.56 |  | 13 | 4.70 | 0.002 | 10.24 | 5.09, 15.39 |
| (pentadecanoic | 2,3 | 8 | 1.61 | -0.39, 3.60 | 0.115 | Yes (98%) | 7.15 | -0.26, 14.56 |  | 12 | 4.70 | 0.003 | 10.41 | 4.83, 15.99 |
| acid) | 4,5 | 9 | 1.59 | -0.01, 3.18 | 0.052 | Yes (98%) | 8.91 | 1.52, 16.30 |  | 21 | 4.70 | <0.001 | 10.34 | 6.42, 14.26 |
|  | 6,7 | 9 | 1.59 | -0.01, 3.18 | 0.052 | Yes (98%) | 8.91 | 1.52, 16.30 |  | 20 | 4.70 | <0.001 | 10.45 | 6.34, 14.57 |
| 16:0 | std,1 | 14 | -0.50 | -1.17, 0.17 | 0.142 | Yes (86%) | -4.65 | -8.45, -0.85 |  | 20 | 4.57 | 0.013 | -3.74 | -6.81, -0.67 |
| (palmitic acid) | 2,3 | 15 | -0.53 | -1.14, 0.08 | 0.089 | Yes (84%) | -4.80 | -8.36, -1.25 |  | 21 | 4.57 | 0.006 | -3.89 | -6.83, -0.96 |
|  | 4,5 | 16 | -0.45 | -1.11, 0.20 | 0.177 | Yes (90%) | -4.38 | -7.99, -0.77 |  | 29 | 4.58 | 0.033 | -2.75 | -5.56, 0.06 |
|  | 6,7 | 17 | -0.48 | -1.09, 0.13 | 0.121 | Yes (89%) | -4.53 | -7.94, -1.12 |  | 30 | 4.58 | 0.022 | -2.90 | -5.62, -0.17 |
| 17:0 | std,1 | 9 | 0.72 | -0.45, 1.89 | 0.226 | Yes (95%) | 9.71 | -2.09, 21.52 |  | 11 | 4.69 | 0.010 | 9.87 | 0.26, 19.48 |
| (heptadecanoic | 2,3 | 9 | 0.72 | -0.45, 1.89 | 0.226 | Yes (95%) | 9.71 | -2.09, 21.52 |  | 11 | 4.69 | 0.010 | 9.87 | 0.26, 19.48 |
| acid) | 4,5 | 10 | 0.68 | -0.32, 1.67 | 0.182 | Yes (96%) | 9.74 | -0.82, 20.3 |  | 13 | 4.69 | 0.003 | 9.62 | 1.53, 17.71 |
|  | 6,7 | 10 | 0.68 | -0.32, 1.67 | 0.182 | Yes (96%) | 9.74 | -0.82, 20.3 |  | 13 | 4.69 | 0.002 | 9.62 | 1.53, 17.71 |
| n, number of data points included in the comparison; MPD, mean percent difference; SMD, standardised mean difference of random-effect model. *Sensitivity analysis number: std – standard meta-analysis; 1-6 – sensitivity analysis 1 to 6 (see main article and online supplementary Table S5 for details); †*P* value <0.05 indicates significance of the difference in composition between organic and conventional milk; ‡Heterogeneity and the I^2^ Statistic; §Magnitude of difference between organic and conventional samples (value <0 indicate higher concentration in CONV, value >0 indicate higher concentration in ORG); \|\|Ln ratio = Ln(ORG/CONV × 100%). | | | | | | | | | | | | | | |

| **Table A1 cont.** Results of meta-analysis comparing composition of organic (ORG) vs conventional (CONV) bovine milk using standard meta-analysis and sensitivity analyses protocols 1-7. | | | | | | | | | | | | | | |
| --- | --- | --- | --- | --- | --- | --- | --- | --- | --- | --- | --- | --- | --- | --- |
|  |  | **Weighted meta-analysis** | | | | | | |  | **Unweighted meta-analysis** | | | | |
| **Parameter** | **an*** | ***n*** | **SMD** | **95% CI** | ***P***† | **Heterogen.**‡ | **MPD**§ | **95% CI** |  | ***n*** | **Ln ratio**\|\| | ***P***† | **MPD**§ | **95% CI** |
| 20:0 | std,1 | 4 | 0.73 | -0.76, 2.22 | 0.336 | Yes (96%) | 13.64 | -2.34, 29.61 |  | 9 | 4.70 | 0.042 | 10.72 | 0.40, 21.05 |
| (arachidic acid) | 2,3 | 4 | 0.73 | -0.76, 2.22 | 0.336 | Yes (96%) | 13.64 | -2.34, 29.61 |  | 8 | 4.70 | 0.065 | 11.10 | -0.58, 22.78 |
|  | 4,5 | 5 | 0.41 | -0.43, 1.24 | 0.341 | Yes (94%) | 13.13 | 0.72, 25.55 |  | 17 | 4.72 | 0.001 | 13.39 | 6.90, 19.88 |
|  | 6,7 | 5 | 0.41 | -0.43, 1.24 | 0.341 | Yes (94%) | 13.13 | 0.72, 25.55 |  | 16 | 4.73 | 0.001 | 13.75 | 6.88, 20.62 |
| Phytanic acid | std,1 | 3 | -3.27 | -6.81, 0.28 | 0.071 | Yes (93%) | -269.5 | -552.5, 13.5 |  | 4 | 3.63 | 0.064 | -216.6 | -442.0, 8.8 |
| diastereomers | 2,3 | 3 | -3.27 | -6.81, 0.28 | 0.071 | Yes (93%) | -269.5 | -552.5, 13.5 |  | 4 | 3.63 | 0.058 | -216.6 | -442.0, 8.8 |
| ratio (SRR/RRR) | 4,5 | 6 | -2.21 | -3.66, -0.75 | 0.003 | Yes (83%) | -202.6 | -353.4, -51.7 |  | 10 | 3.87 | 0.005 | -155.3 | -267.7, -42.8 |
|  | 6,7 | 6 | -2.21 | -3.66, -0.75 | 0.003 | Yes (83%) | -202.6 | -353.4, -51.7 |  | 10 | 3.87 | 0.006 | -155.3 | -267.7, -42.8 |
| 22:0 | std,1 | 3 | 1.27 | -0.85, 3.39 | 0.239 | Yes (94%) | 30.88 | -7.82, 69.59 |  | 7 | 4.75 | 0.158 | 17.70 | -12.19, 47.59 |
| (behenic acid) | 2,3 | 3 | 1.27 | -0.85, 3.39 | 0.239 | Yes (94%) | 30.88 | -7.82, 69.59 |  | 6 | 4.74 | 0.193 | 17.52 | -17.84, 52.89 |
|  | 4,5 | 3 | 1.27 | -0.85, 3.39 | 0.239 | Yes (94%) | 30.88 | -7.82, 69.59 |  | 11 | 4.81 | 0.033 | 25.66 | 2.25, 49.06 |
|  | 6,7 | 3 | 1.27 | -0.85, 3.39 | 0.239 | Yes (94%) | 30.88 | -7.82, 69.59 |  | 10 | 4.81 | 0.044 | 26.35 | 0.52, 52.18 |
| 24:0 | std,1 | - | - | - | - | - | - | - |  | 5 | 4.78 | 0.065 | 20.84 | 2.57, 39.11 |
| (lignoceric acid) | 2,3 | - | - | - | - | - | - | - |  | 4 | 4.79 | 0.123 | 21.88 | -1.56, 45.33 |
|  | 4,5 | - | - | - | - | - | - | - |  | 9 | 4.78 | 0.017 | 20.47 | 7.12, 33.82 |
|  | 6,7 | - | - | - | - | - | - | - |  | 8 | 4.78 | 0.030 | 20.94 | 5.84, 36.04 |
| n, number of data points included in the comparison; MPD, mean percent difference; SMD, standardised mean difference of random-effect model. *Sensitivity analysis number: std – standard meta-analysis; 1-6 – sensitivity analysis 1 to 6 (see main article and online supplementary Table S5 for details); †*P* value <0.05 indicates significance of the difference in composition between organic and conventional milk; ‡Heterogeneity and the I^2^ Statistic; §Magnitude of difference between organic and conventional samples (value <0 indicate higher concentration in CONV, value >0 indicate higher concentration in ORG); \|\|Ln ratio = Ln(ORG/CONV × 100%). | | | | | | | | | | | | | | |

| **Table A1 cont.** Results of meta-analysis comparing composition of organic (ORG) vs conventional (CONV) bovine milk using standard meta-analysis and sensitivity analyses protocols 1-7. | | | | | | | | | | | | | | |
| --- | --- | --- | --- | --- | --- | --- | --- | --- | --- | --- | --- | --- | --- | --- |
|  |  | **Weighted meta-analysis** | | | | | | |  | **Unweighted meta-analysis** | | | | |
| **Parameter** | **an*** | ***n*** | **SMD** | **95% CI** | ***P***† | **Heterogen.**‡ | **MPD**§ | **95% CI** |  | ***n*** | **Ln ratio**\|\| | ***P***† | **MPD**§ | **95% CI** |
| MUFA | std,1 | 19 | 0.18 | -0.40, 0.76 | 0.547 | Yes (81%) | 1.20 | -3.13, 5.53 |  | 31 | 4.60 | 0.446 | -0.15 | -3.34, 3.04 |
|  | 2,3 | 19 | 0.18 | -0.40, 0.76 | 0.547 | Yes (81%) | 1.20 | -3.13, 5.53 |  | 30 | 4.60 | 0.440 | -0.14 | -3.44, 3.16 |
|  | 4,5 | 21 | 0.16 | -0.34, 0.67 | 0.526 | Yes (77%) | 0.93 | -3.03, 4.89 |  | 44 | 4.60 | 0.322 | -0.53 | -3.00, 1.93 |
|  | 6,7 | 21 | 0.16 | -0.34, 0.67 | 0.526 | Yes (77%) | 0.93 | -3.03, 4.89 |  | 43 | 4.60 | 0.318 | -0.54 | -3.06, 1.98 |
| OA (cis-9-18:1) | std,1 | 10 | 0.28 | -0.64, 1.20 | 0.547 | Yes (91%) | 2.78 | -3.32, 8.88 |  | 16 | 4.62 | 0.290 | 1.41 | -3.29, 6.10 |
|  | 2,3 | 10 | 0.28 | -0.64, 1.20 | 0.547 | Yes (91%) | 2.78 | -3.32, 8.88 |  | 15 | 4.62 | 0.281 | 1.56 | -3.45, 6.57 |
|  | 4,5 | 12 | -0.01 | -0.97, 0.95 | 0.981 | Yes (95%) | 1.25 | -4.46, 6.96 |  | 24 | 4.60 | 0.328 | -0.89 | -4.78, 3.00 |
|  | 6,7 | 12 | -0.01 | -0.97, 0.95 | 0.981 | Yes (95%) | 1.25 | -4.46, 6.96 |  | 23 | 4.60 | 0.335 | -0.89 | -4.95, 3.17 |
| trans-18:1 | std,1 | 4 | 0.39 | -0.40, 1.18 | 0.337 | Yes (63%) | 50.43 | -24.9, 125.8 |  | 6 | 4.94 | 0.047 | 49.36 | -0.64, 99.37 |
|  | 2,3 | 4 | 0.39 | -0.40, 1.18 | 0.337 | Yes (63%) | 50.43 | -24.9, 125.8 |  | 6 | 4.94 | 0.050 | 49.36 | -0.64, 99.37 |
|  | 4,5 | 4 | 0.39 | -0.40, 1.18 | 0.337 | Yes (63%) | 50.43 | -24.9, 125.8 |  | 12 | 4.97 | 0.001 | 51.33 | 22.37, 80.29 |
|  | 6,7 | 4 | 0.39 | -0.40, 1.18 | 0.337 | Yes (63%) | 50.43 | -24.9, 125.8 |  | 12 | 4.97 | 0.001 | 51.33 | 22.37, 80.29 |
| VA | std,1 | 12 | 2.48 | 1.08, 3.87 | 0.001 | Yes (95%) | 65.91 | 19.7, 112.1 |  | 18 | 5.01 | <0.001 | 58.07 | 27.01, 89.12 |
| (trans-11-18:1) | 2,3 | 12 | 2.48 | 1.08, 3.87 | 0.001 | Yes (95%) | 65.91 | 19.7, 112.1 |  | 17 | 5.01 | <0.001 | 59.08 | 26.21, 91.95 |
|  | 4,5 | 15 | 1.82 | 0.83, 2.80 | <0.001 | Yes (95%) | 55.92 | 17.3, 94.6 |  | 22 | 4.99 | <0.001 | 55.17 | 28.10, 82.24 |
|  | 6,7 | 15 | 1.82 | 0.83, 2.80 | <0.001 | Yes (95%) | 55.92 | 17.3, 94.6 |  | 21 | 4.99 | <0.001 | 55.85 | 27.49, 84.21 |
| n, number of data points included in the comparison; MPD, mean percent difference; SMD, standardised mean difference of random-effect model; MUFA, monounsaturated fatty acids; OA, oleic acid; VA, vaccenic acid. *Sensitivity analysis number: std – standard meta-analysis; 1-6 – sensitivity analysis 1 to 6 (see main article and online supplementary Table S5 for details); †*P* value <0.05 indicates significance of the difference in composition between organic and conventional milk; ‡Heterogeneity and the I^2^ Statistic; §Magnitude of difference between organic and conventional samples (value <0 indicate higher concentration in CONV, value >0 indicate higher concentration in ORG); \|\|Ln ratio = Ln(ORG/CONV × 100%). | | | | | | | | | | | | | | |

| **Table A1 cont.** Results of meta-analysis comparing composition of organic (ORG) vs conventional (CONV) bovine milk using standard meta-analysis and sensitivity analyses protocols 1-7. | | | | | | | | | | | | | | |
| --- | --- | --- | --- | --- | --- | --- | --- | --- | --- | --- | --- | --- | --- | --- |
|  |  | **Weighted meta-analysis** | | | | | | |  | **Unweighted meta-analysis** | | | | |
| **Parameter** | **an*** | ***n*** | **SMD** | **95% CI** | ***P***† | **Heterogen.**‡ | **MPD**§ | **95% CI** |  | ***n*** | **Ln ratio**\|\| | ***P***† | **MPD**§ | **95% CI** |
| cis-9-20:1 | std,1 | - | - | - | - | - | - | - |  | 3 | 4.84 | 0.247 | 29.26 | -7.76, 66.27 |
|  | 2,3 | - | - | - | - | - | - | - |  | 3 | 4.84 | 0.253 | 29.26 | -7.76, 66.27 |
|  | 4,5 | - | - | - | - | - | - | - |  | 9 | 4.86 | 0.004 | 30.29 | 18.55, 42.03 |
|  | 6,7 | - | - | - | - | - | - | - |  | 9 | 4.86 | 0.004 | 30.29 | 18.55, 42.03 |
| PUFA | std,1 | 19 | 0.88 | 0.19, 1.56 | 0.012 | Yes (87%) | 7.30 | -0.73, 15.34 |  | 30 | 4.73 | <0.001 | 14.78 | 7.05, 22.51 |
|  | 2,3 | 19 | 0.88 | 0.19, 1.56 | 0.012 | Yes (87%) | 7.30 | -0.73, 15.34 |  | 29 | 4.73 | <0.001 | 14.47 | 6.49, 22.44 |
|  | 4,5 | 21 | 0.77 | 0.14, 1.39 | 0.016 | Yes (85%) | 6.69 | -0.63, 14.01 |  | 43 | 4.72 | <0.001 | 13.80 | 7.67, 19.93 |
|  | 6,7 | 21 | 0.77 | 0.14, 1.39 | 0.016 | Yes (85%) | 6.69 | -0.63, 14.01 |  | 42 | 4.72 | <0.001 | 13.56 | 7.30, 19.82 |
| CLA (total) | std,1 | 11 | 1.40 | 0.37, 2.42 | 0.008 | Yes (85%) | 41.13 | 14.19, 68.08 |  | 19 | 4.94 | <0.001 | 47.47 | 20.78, 74.16 |
|  | 2,3 | 11 | 1.40 | 0.37, 2.42 | 0.008 | Yes (85%) | 41.13 | 14.19, 68.08 |  | 19 | 4.94 | <0.001 | 47.47 | 20.78, 74.16 |
|  | 4,5 | 11 | 1.40 | 0.37, 2.42 | 0.008 | Yes (85%) | 41.13 | 14.19, 68.08 |  | 23 | 4.90 | <0.001 | 42.39 | 17.82, 66.96 |
|  | 6,7 | 11 | 1.40 | 0.37, 2.42 | 0.008 | Yes (85%) | 41.13 | 14.19, 68.08 |  | 23 | 4.90 | <0.001 | 42.39 | 17.82, 66.96 |
| CLA9 | std,1 | 14 | 1.22 | 0.50, 1.95 | 0.001 | Yes (92%) | 23.89 | 8.39, 39.39 |  | 20 | 4.87 | <0.001 | 34.36 | 17.93, 50.80 |
| (cis-9-trans-11- | 2,3 | 15 | 1.25 | 0.58, 1.92 | <0.001 | Yes (91%) | 24.59 | 10.04, 39.14 |  | 21 | 4.87 | <0.001 | 34.36 | 18.71, 50.02 |
| 18:2) | 4,5 | 17 | 1.06 | 0.40, 1.71 | 0.002 | Yes (93%) | 21.63 | 7.71, 35.55 |  | 30 | 4.86 | <0.001 | 34.10 | 19.31, 48.89 |
|  | 6,7 | 18 | 1.09 | 0.48, 1.71 | 0.001 | Yes (92%) | 22.34 | 9.10, 35.58 |  | 31 | 4.86 | <0.001 | 34.11 | 19.79, 48.43 |
| n, number of data points included in the comparison; MPD, mean percent difference; SMD, standardised mean difference of random-effect model; PUFA, polyunsaturated fatty acids; CLA, conjugated linoleic acid. *Sensitivity analysis number: std – standard meta-analysis; 1-6 – sensitivity analysis 1 to 6 (see main article and online supplementary Table S5 for details); †*P* value <0.05 indicates significance of the difference in composition between organic and conventional milk; ‡Heterogeneity and the I^2^ Statistic; §Magnitude of difference between organic and conventional samples (value <0 indicate higher concentration in CONV, value >0 indicate higher concentration in ORG); \|\|Ln ratio = Ln(ORG/CONV × 100%). | | | | | | | | | | | | | | |

| **Table A1 cont.** Results of meta-analysis comparing composition of organic (ORG) vs conventional (CONV) bovine milk using standard meta-analysis and sensitivity analyses protocols 1-7. | | | | | | | | | | | | | | |
| --- | --- | --- | --- | --- | --- | --- | --- | --- | --- | --- | --- | --- | --- | --- |
|  |  | **Weighted meta-analysis** | | | | | | |  | **Unweighted meta-analysis** | | | | |
| **Parameter** | **an*** | ***n*** | **SMD** | **95% CI** | ***P***† | **Heterogen.**‡ | **MPD**§ | **95% CI** |  | ***n*** | **Ln ratio**\|\| | ***P***† | **MPD**§ | **95% CI** |
| CLA10 | std,1 | 3 | 1.20 | -1.03, 3.43 | 0.293 | Yes (95%) | 28.24 | -20.92, 77.40 |  | 7 | 4.86 | 0.061 | 34.96 | 2.94, 66.98 |
| (trans-10-cis-12- | 2,3 | 3 | 1.20 | -1.03, 3.43 | 0.293 | Yes (95%) | 28.24 | -20.92, 77.40 |  | 6 | 4.91 | 0.065 | 40.79 | 5.39, 76.18 |
| 18:2) | 4,5 | 4 | 0.79 | -0.94, 2.52 | 0.371 | Yes (94%) | 17.39 | -23.36, 58.14 |  | 14 | 4.96 | 0.004 | 52.11 | 22.34, 81.88 |
|  | 6,7 | 4 | 0.79 | -0.94, 2.52 | 0.371 | Yes (94%) | 17.39 | -23.36, 58.14 |  | 13 | 4.99 | 0.003 | 56.12 | 25.11, 87.13 |
| 18:4 | std,1 | - | - | - | - | - | - | - |  | 3 | 4.99 | 0.251 | 68.89 | -59.65, 197.43 |
|  | 2,3 | - | - | - | - | - | - | - |  | 3 | 4.99 | 0.255 | 68.89 | -59.65, 197.43 |
|  | 4,5 | - | - | - | - | - | - | - |  | 9 | 5.04 | 0.008 | 67.41 | 16.20, 118.62 |
|  | 6,7 | - | - | - | - | - | - | - |  | 9 | 5.04 | 0.009 | 67.41 | 16.20, 118.62 |
| n-3 FA | std,1 | 12 | 2.18 | 1.11, 3.25 | <0.001 | Yes (91%) | 55.67 | 37.68, 73.66 |  | 20 | 5.05 | <0.001 | 60.14 | 45.07, 75.20 |
|  | 2,3 | 13 | 2.16 | 1.21, 3.11 | <0.001 | Yes (90%) | 55.72 | 39.10, 72.34 |  | 21 | 5.05 | <0.001 | 59.95 | 45.59, 74.32 |
|  | 4,5 | 13 | 2.39 | 1.29, 3.48 | <0.001 | Yes (92%) | 59.08 | 41.23, 76.93 |  | 29 | 5.05 | <0.001 | 60.71 | 45.85, 75.58 |
|  | 6,7 | 14 | 2.35 | 1.37, 3.34 | <0.001 | Yes (91%) | 58.88 | 42.29, 75.47 |  | 30 | 5.05 | <0.001 | 60.57 | 46.19, 74.95 |
| ALA | std,1 | 21 | 3.05 | 2.08, 4.02 | <0.001 | Yes (95%) | 68.62 | 53.04, 84.20 |  | 34 | 5.16 | <0.001 | 78.66 | 66.04, 91.29 |
| (cis-9,12,15- | 2,3 | 22 | 3.03 | 2.12, 3.95 | <0.001 | Yes (94%) | 68.33 | 53.47, 83.19 |  | 35 | 5.16 | <0.001 | 78.19 | 65.90, 90.48 |
| 18:3) | 4,5 | 24 | 3.00 | 2.14, 3.86 | <0.001 | Yes (95%) | 67.57 | 52.90, 82.24 |  | 44 | 5.12 | <0.001 | 72.89 | 59.04, 86.75 |
|  | 6,7 | 25 | 2.99 | 2.18, 3.81 | <0.001 | Yes (94%) | 67.35 | 53.28, 81.43 |  | 45 | 5.12 | <0.001 | 72.66 | 59.11, 86.21 |
| n, number of data points included in the comparison; MPD, mean percent difference; SMD, standardised mean difference of random-effect model; CLA, conjugated linoleic acid; FA, fatty acids; ALA, α-linolenic acid. *Sensitivity analysis number: std – standard meta-analysis; 1-6 – sensitivity analysis 1 to 6 (see main article and online supplementary Table S5 for details); †*P* value <0.05 indicates significance of the difference in composition between organic and conventional milk; ‡Heterogeneity and the I^2^ Statistic; §Magnitude of difference between organic and conventional samples (value <0 indicate higher concentration in CONV, value >0 indicate higher concentration in ORG); \|\|Ln ratio = Ln(ORG/CONV × 100%). | | | | | | | | | | | | | | |

| **Table A1 cont.** Results of meta-analysis comparing composition of organic (ORG) vs conventional (CONV) bovine milk using standard meta-analysis and sensitivity analyses protocols 1-7. | | | | | | | | | | | | | | |
| --- | --- | --- | --- | --- | --- | --- | --- | --- | --- | --- | --- | --- | --- | --- |
|  |  | **Weighted meta-analysis** | | | | | | |  | **Unweighted meta-analysis** | | | | |
| **Parameter** | **an*** | ***n*** | **SMD** | **95% CI** | ***P***† | **Heterogen.**‡ | **MPD**§ | **95% CI** |  | ***n*** | **Ln ratio**\|\| | ***P***† | **MPD**§ | **95% CI** |
| DPA | std,1 | 5 | 1.24 | 0.37, 2.12 | 0.005 | Yes (89%) | 44.83 | 18.23, 71.44 |  | 8 | 4.91 | 0.003 | 38.23 | 20.57, 55.89 |
| (cis-7,10,13,16, | 2,3 | 5 | 1.24 | 0.37, 2.12 | 0.005 | Yes (89%) | 44.83 | 18.23, 71.44 |  | 7 | 4.94 | 0.009 | 41.31 | 22.15, 60.47 |
| 19-22:5) | 4,5 | 6 | 1.06 | 0.37, 1.75 | 0.002 | Yes (90%) | 51.25 | 26.15, 76.35 |  | 13 | 4.92 | <0.001 | 39.34 | 23.99, 54.68 |
|  | 6,7 | 6 | 1.06 | 0.37, 1.75 | 0.002 | Yes (90%) | 51.25 | 26.15, 76.35 |  | 12 | 4.93 | <0.001 | 41.23 | 25.04, 57.42 |
| EPA | std,1 | 8 | 1.31 | 0.56, 2.06 | 0.001 | Yes (90%) | 67.14 | 32.4, 101.9 |  | 14 | 5.07 | <0.001 | 66.34 | 39.86, 92.82 |
| (cis-5,8,11,14, | 2,3 | 8 | 1.31 | 0.56, 2.06 | 0.001 | Yes (90%) | 67.14 | 32.4, 101.9 |  | 13 | 5.07 | <0.001 | 66.83 | 38.25, 95.41 |
| 17-20:5) | 4,5 | 9 | 1.17 | 0.48, 1.86 | 0.001 | Yes (93%) | 70.80 | 39.3, 102.3 |  | 20 | 5.06 | <0.001 | 62.63 | 43.17, 82.09 |
|  | 6,7 | 9 | 1.17 | 0.48, 1.86 | 0.001 | Yes (93%) | 70.80 | 39.3, 102.3 |  | 19 | 5.06 | <0.001 | 62.77 | 42.26, 83.28 |
| DHA | std,1 | 3 | 0.21 | -0.26, 0.68 | 0.379 | Yes (29%) | 21.48 | -3.71, 46.67 |  | 6 | 5.26 | 0.060 | 194.07 | -89.1, 477.3 |
| (cis-4,7,10,13, | 2,3 | 3 | 0.21 | -0.26, 0.68 | 0.379 | Yes (29%) | 21.48 | -3.71, 46.67 |  | 5 | 5.18 | 0.132 | 192.89 | -154.0, 539.7 |
| 16,19-22:6) | 4,5 | 3 | 0.21 | -0.26, 0.68 | 0.379 | Yes (29%) | 21.48 | -3.71, 46.67 |  | 10 | 5.48 | 0.007 | 241.44 | 50.8, 432.1 |
|  | 6,7 | 3 | 0.21 | -0.26, 0.68 | 0.379 | Yes (29%) | 21.48 | -3.71, 46.67 |  | 9 | 5.46 | 0.015 | 246.05 | 33.1, 459.0 |
| VLC n-3 PUFA¶ | std,1 | - | - | - | - | - | - | - |  | 5 | 5.04 | 0.030 | 57.16 | 27.25, 87.07 |
|  | 2,3 | - | - | - | - | - | - | - |  | 4 | 5.04 | 0.065 | 58.95 | 20.60, 97.30 |
|  | 4,5 | - | - | - | - | - | - | - |  | 9 | 5.07 | 0.002 | 61.45 | 43.70, 79.21 |
|  | 6,7 | - | - | - | - | - | - | - |  | 8 | 5.08 | 0.004 | 62.89 | 43.01, 82.76 |
| n, number of data points included in the comparison; MPD, mean percent difference; SMD, standardised mean difference of random-effect model; EPA, eicosapentaenoic acid; DPA, docosapentaenoic acid; DHA, docosahexaenoic acid; VLC n-3 PUFA, very long chain n-3 PUFA (EPA+DPA+DHA). *Sensitivity analysis number: std – standard meta-analysis; 1-6 – sensitivity analysis 1 to 6 (see main article and online supplementary Table S5 for details); †*P* value <0.05 indicates significance of the difference in composition between organic and conventional milk; ‡Heterogeneity and the I^2^ Statistic; §Magnitude of difference between organic and conventional samples (value <0 indicate higher concentration in CONV, value >0 indicate higher concentration in ORG); \|\|Ln ratio = Ln(ORG/CONV × 100%); ¶Calculated based on published fatty acids composition data. | | | | | | | | | | | | | | |

| **Table A1 cont.** Results of meta-analysis comparing composition of organic (ORG) vs conventional (CONV) bovine milk using standard meta-analysis and sensitivity analyses protocols 1-7. | | | | | | | | | | | | | | |
| --- | --- | --- | --- | --- | --- | --- | --- | --- | --- | --- | --- | --- | --- | --- |
|  |  | **Weighted meta-analysis** | | | | | | |  | **Unweighted meta-analysis** | | | | |
| **Parameter** | **an*** | ***n*** | **SMD** | **95% CI** | ***P***† | **Heterogen.**‡ | **MPD**§ | **95% CI** |  | ***n*** | **Ln ratio**\|\| | ***P***† | **MPD**§ | **95% CI** |
| n-6 FA | std,1 | 12 | -0.06 | -0.97, 0.86 | 0.904 | Yes (91%) | -4.03 | -13.83, 5.76 |  | 20 | 4.59 | 0.354 | -1.50 | -10.62, 7.61 |
|  | 2,3 | 13 | 0.03 | -0.82, 0.89 | 0.940 | Yes (91%) | -2.73 | -12.10, 6.64 |  | 21 | 4.60 | 0.428 | -0.82 | -9.59, 7.96 |
|  | 4,5 | 14 | -0.11 | -1.03, 0.81 | 0.814 | Yes (92%) | -3.44 | -15.66, 8.78 |  | 27 | 4.57 | 0.216 | -3.34 | -11.80, 5.13 |
|  | 6,7 | 15 | -0.03 | -0.89, 0.84 | 0.950 | Yes (91%) | -2.35 | -13.93, 9.23 |  | 28 | 4.58 | 0.239 | -2.76 | -10.99, 5.48 |
| LA | std,1 | 12 | -0.92 | -1.96, 0.11 | 0.080 | Yes (94%) | -14.40 | -29.51, 0.71 |  | 22 | 4.56 | 0.189 | -4.82 | -15.27, 5.64 |
| (cis-9,12-18:2) | 2,3 | 12 | -0.92 | -1.96, 0.11 | 0.080 | Yes (94%) | -14.40 | -29.51, 0.71 |  | 21 | 4.56 | 0.154 | -5.75 | -16.55, 5.04 |
|  | 4,5 | 14 | -1.10 | -2.00, -0.19 | 0.017 | Yes (95%) | -17.97 | -31.73, -4.22 |  | 31 | 4.57 | 0.155 | -4.50 | -13.08, 4.09 |
|  | 6,7 | 14 | -1.10 | -2.00, -0.19 | 0.017 | Yes (95%) | -17.97 | -31.73, -4.22 |  | 30 | 4.56 | 0.137 | -5.14 | -13.92, 3.64 |
| GLA | std,1 | 4 | 0.20 | -0.19, 0.59 | 0.311 | No (9%) | 741.67 | -605, 2088 |  | 7 | 5.29 | 0.032 | 430.60 | -344, 1205 |
| (cis-6,9,12-18:3) | 2,3 | 4 | 0.20 | -0.19, 0.59 | 0.311 | No (9%) | 741.67 | -605, 2088 |  | 7 | 5.29 | 0.028 | 430.60 | -344, 1205 |
|  | 4,5 | 5 | 0.27 | -0.12, 0.66 | 0.170 | No (15%) | 610.48 | -464, 1685 |  | 15 | 5.07 | 0.001 | 220.91 | -141, 582 |
|  | 6,7 | 5 | 0.27 | -0.12, 0.66 | 0.170 | No (15%) | 610.48 | -464, 1685 |  | 15 | 5.07 | <0.001 | 220.91 | -141, 582 |
| DGLA | std,1 | - | - | - | - | - | - | - |  | 4 | 4.40 | 0.122 | -23.89 | -40.50, -7.28 |
| (cis-8-11-14- | 2,3 | - | - | - | - | - | - | - |  | 4 | 4.40 | 0.124 | -23.89 | -40.50, -7.28 |
| C20:3) | 4,5 | - | - | - | - | - | - | - |  | 8 | 4.43 | 0.015 | -20.10 | -29.92, -10.28 |
|  | 6,7 | - | - | - | - | - | - | - |  | 8 | 4.43 | 0.015 | -20.10 | -29.92, -10.28 |
| n, number of data points included in the comparison; MPD, mean percent difference; SMD, standardised mean difference of random-effect model; FA, fatty acids; LA, linoleic acid; DGLA, dihomo-γ-linolenic acid; GLA, γ-linolenic acid. *Sensitivity analysis number: std – standard meta-analysis; 1-6 – sensitivity analysis 1 to 6 (see main article and online supplementary Table S5 for details); †*P* value <0.05 indicates significance of the difference in composition between organic and conventional milk; ‡Heterogeneity and the I^2^ Statistic; §Magnitude of difference between organic and conventional samples (value <0 indicate higher concentration in CONV, value >0 indicate higher concentration in ORG); \|\|Ln ratio = Ln(ORG/CONV × 100%). | | | | | | | | | | | | | | |

| **Table A1 cont.** Results of meta-analysis comparing composition of organic (ORG) vs conventional (CONV) bovine milk using standard meta-analysis and sensitivity analyses protocols 1-7. | | | | | | | | | | | | | | |
| --- | --- | --- | --- | --- | --- | --- | --- | --- | --- | --- | --- | --- | --- | --- |
|  |  | **Weighted meta-analysis** | | | | | | |  | **Unweighted meta-analysis** | | | | |
| **Parameter** | **an*** | ***n*** | **SMD** | **95% CI** | ***P***† | **Heterogen.**‡ | **MPD**§ | **95% CI** |  | ***n*** | **Ln ratio**\|\| | ***P***† | **MPD**§ | **95% CI** |
| AA | std,1 | 5 | -0.98 | -1.95, 0 | 0.050 | Yes (92%) | -24.15 | -41.0, -7.3 |  | 9 | 4.43 | 0.008 | -20.58 | -30.8, -10.3 |
| (cis-5,8,11,14- | 2,3 | 5 | -0.98 | -1.95, 0 | 0.050 | Yes (92%) | -24.15 | -41.0, -7.3 |  | 8 | 4.40 | 0.008 | -23.15 | -33.3, -13.0 |
| 20:4) | 4,5 | 6 | -0.81 | -1.57, -0.04 | 0.038 | Yes (93%) | -24.67 | -38.5, -10.9 |  | 14 | 4.42 | <0.001 | -21.76 | -29.8, -13.7 |
|  | 6,7 | 6 | -0.81 | -1.57, -0.04 | 0.038 | Yes (93%) | -24.67 | -38.5, -10.9 |  | 13 | 4.40 | <0.001 | -23.44 | -31.4, -15.5 |
| LA/ALA ratio¶ | std,1 | - | - | - | - | - | - | - |  | 19 | 3.98 | <0.001 | -93.34 | -116.4, -70.3 |
|  | 2,3 | - | - | - | - | - | - | - |  | 18 | 3.97 | <0.001 | -96.50 | -120.0, -73.0 |
|  | 4,5 | - | - | - | - | - | - | - |  | 28 | 4.05 | <0.001 | -86.46 | -112.0, -60.9 |
|  | 6,7 | - | - | - | - | - | - | - |  | 27 | 4.04 | <0.001 | -88.31 | -114.6, -62.0 |
| n-3/n-6 ratio | std,1 | 5 | 1.50 | 0.81, 2.19 | <0.001 | Yes (65%) | 72.21 | 36.1, 108.4 |  | 24 | 5.06 | <0.001 | 64.95 | 44.22, 85.67 |
|  | 2,3 | 6 | 1.43 | 0.88, 1.98 | 0.000 | Yes (56%) | 66.55 | 34.9, 98.2 |  | 25 | 5.05 | <0.001 | 63.88 | 43.88, 83.88 |
|  | 4,5 | 7 | 1.58 | 0.59, 2.58 | 0.002 | Yes (85%) | 74.95 | 27.8, 122.1 |  | 34 | 5.03 | <0.001 | 63.46 | 42.96, 83.97 |
|  | 6,7 | 8 | 1.50 | 0.69, 2.32 | <0.001 | Yes (82%) | 70.36 | 28.5, 112.2 |  | 35 | 5.03 | <0.001 | 62.74 | 42.78, 82.71 |
| n-6/n-3 ratio | std,1 | 7 | -2.26 | -4.34, -0.18 | 0.033 | Yes (95%) | -71.16 | -122.0, -20.3 |  | 23 | 4.11 | <0.001 | -72.07 | -92.9, -51.3 |
|  | 2,3 | 7 | -2.26 | -4.34, -0.18 | 0.033 | Yes (95%) | -71.16 | -122.0, -20.3 |  | 24 | 4.12 | <0.001 | -70.03 | -90.3, -49.8 |
|  | 4,5 | 7 | -2.26 | -4.34, -0.18 | 0.033 | Yes (95%) | -71.16 | -122.0, -20.3 |  | 33 | 4.15 | <0.001 | -68.39 | -89.2, -47.6 |
|  | 6,7 | 7 | -2.26 | -4.34, -0.18 | 0.033 | Yes (95%) | -71.16 | -122.0, -20.3 |  | 34 | 4.15 | <0.001 | -67.05 | -87.4, -46.7 |
| n, number of data points included in the comparison; MPD, mean percent difference; SMD, standardised mean difference of random-effect model; AA, arachidonic acid; ALA, α-linolenic acid. *Sensitivity analysis number: std – standard meta-analysis; 1-6 – sensitivity analysis 1 to 6 (see main article and online supplementary Table S5 for details); †*P* value <0.05 indicates significance of the difference in composition between organic and conventional milk; ‡Heterogeneity and the I^2^ Statistic; §Magnitude of difference between organic and conventional samples (value <0 indicate higher concentration in CONV, value >0 indicate higher concentration in ORG); \|\|Ln ratio = Ln(ORG/CONV × 100%); ¶Calculated based on published fatty acids composition data. | | | | | | | | | | | | | | |

| **Table A1 cont.** Results of meta-analysis comparing composition of organic (ORG) vs conventional (CONV) bovine milk using standard meta-analysis and sensitivity analyses protocols 1-7. | | | | | | | | | | | | | | |
| --- | --- | --- | --- | --- | --- | --- | --- | --- | --- | --- | --- | --- | --- | --- |
|  |  | **Weighted meta-analysis** | | | | | | |  | **Unweighted meta-analysis** | | | | |
| **Parameter** | **an*** | ***n*** | **SMD** | **95% CI** | ***P***† | **Heterogen.**‡ | **MPD**§ | **95% CI** |  | ***n*** | **Ln ratio**\|\| | ***P***† | **MPD**§ | **95% CI** |
| *N compounds* | | | |  |  |  |  |  |  |  |  |  |  |  |
| Urea | std,1 | 7 | -0.42 | -1.04, 0.19 | 0.176 | Yes (70%) | -9.67 | -24.7, 5.36 |  | 11 | 4.53 | 0.085 | -8.75 | -19.64, 2.14 |
|  | 2,3 | 7 | -0.42 | -1.04, 0.19 | 0.176 | Yes (70%) | -9.67 | -24.7, 5.36 |  | 11 | 4.53 | 0.079 | -8.75 | -19.64, 2.14 |
|  | 4,5 | 9 | -0.72 | -1.39, -0.05 | 0.035 | Yes (76%) | -13.34 | -26.36, -0.32 |  | 19 | 4.47 | 0.002 | -16.08 | -25.22, -6.94 |
|  | 6,7 | 9 | -0.72 | -1.39, -0.05 | 0.035 | Yes (76%) | -13.34 | -26.36, -0.32 |  | 19 | 4.47 | 0.003 | -16.08 | -25.22, -6.94 |
| *Vitamins and antioxidants* | | | |  |  |  |  |  |  |  |  |  |  |  |
| α-tocopherol | std,1 | 9 | 0.74 | 0.01, 1.47 | 0.047 | Yes (81%) | 12.98 | 0.51, 25.45 |  | 17 | 4.70 | 0.013 | 11.68 | 2.52, 20.84 |
|  | 2,3 | 9 | 0.74 | 0.01, 1.47 | 0.047 | Yes (81%) | 12.98 | 0.51, 25.45 |  | 17 | 4.70 | 0.013 | 11.68 | 2.52, 20.84 |
|  | 4,5 | 11 | 0.61 | -0.08, 1.29 | 0.085 | Yes (81%) | 10.67 | -0.37, 21.72 |  | 25 | 4.71 | 0.003 | 12.20 | 4.46, 19.95 |
|  | 6,7 | 11 | 0.61 | -0.08, 1.29 | 0.085 | Yes (81%) | 10.67 | -0.37, 21.72 |  | 25 | 4.71 | 0.002 | 12.20 | 4.46, 19.95 |
| 2R (synthetic) | std,1 | - | - | - | - | - | - | - |  | 5 | 4.27 | 0.062 | -46.38 | -86.65, -6.11 |
| isomers of | 2,3 | - | - | - | - | - | - | - |  | 5 | 4.27 | 0.066 | -46.38 | -86.65, -6.11 |
| α-tocopherol | 4,5 | - | - | - | - | - | - | - |  | 6 | 4.38 | 0.095 | -32.17 | -75.26, 10.93 |
|  | 6,7 | - | - | - | - | - | - | - |  | 6 | 4.38 | 0.098 | -32.17 | -75.26, 10.93 |
| 3R (natural) | std,1 | - | - | - | - | - | - | - |  | 6 | 4.81 | 0.032 | 24.75 | 1.59, 47.9 |
| isomers of | 2,3 | - | - | - | - | - | - | - |  | 6 | 4.81 | 0.031 | 24.75 | 1.59, 47.9 |
| α-tocopherol | 4,5 | 3 | 0.87 | -1.48, 3.21 | 0.469 | Yes (93%) | 12.32 | -18.59, 43.23 |  | 7 | 4.76 | 0.068 | 18.90 | -3.77, 41.58 |
|  | 6,7 | 3 | 0.87 | -1.48, 3.21 | 0.469 | Yes (93%) | 12.32 | -18.59, 43.23 |  | 7 | 4.76 | 0.071 | 18.90 | -3.77, 41.58 |
| n, number of data points included in the comparison; MPD, mean percent difference; SMD, standardised mean difference of random-effect model. *Sensitivity analysis number: std – standard meta-analysis; 1-6 – sensitivity analysis 1 to 6 (see main article and online supplementary Table S5 for details); †*P* value <0.05 indicates significance of the difference in composition between organic and conventional milk; ‡Heterogeneity and the I^2^ Statistic; §Magnitude of difference between organic and conventional samples (value <0 indicate higher concentration in CONV, value >0 indicate higher concentration in ORG); \|\|Ln ratio = Ln(ORG/CONV × 100%). | | | | | | | | | | | | | | |

| **Table A1 cont.** Results of meta-analysis comparing composition of organic (ORG) vs conventional (CONV) bovine milk using standard meta-analysis and sensitivity analyses protocols 1-7. | | | | | | | | | | | | | | |
| --- | --- | --- | --- | --- | --- | --- | --- | --- | --- | --- | --- | --- | --- | --- |
|  |  | **Weighted meta-analysis** | | | | | | |  | **Unweighted meta-analysis** | | | | |
| **Parameter** | **an*** | ***n*** | **SMD** | **95% CI** | ***P***† | **Heterogen.**‡ | **MPD**§ | **95% CI** |  | ***n*** | **Ln ratio**\|\| | ***P***† | **MPD**§ | **95% CI** |
| Carotenoids | std,1 | 5 | 0.69 | -0.73, 2.1 | 0.342 | Yes (89%) | 31.83 | -37.0, 100.7 |  | 5 | 4.79 | 0.385 | 31.83 | -37.0, 100.7 |
|  | 2,3 | 5 | 0.69 | -0.73, 2.1 | 0.342 | Yes (89%) | 31.83 | -37.0, 100.7 |  | 5 | 4.79 | 0.372 | 31.83 | -37.0, 100.7 |
|  | 4,5 | 7 | 0.64 | -0.96, 2.24 | 0.434 | Yes (92%) | 24.23 | -27.2, 75.7 |  | 7 | 4.75 | 0.254 | 24.23 | -27.2, 75.7 |
|  | 6,7 | 7 | 0.64 | -0.96, 2.24 | 0.434 | Yes (92%) | 24.23 | -27.2, 75.7 |  | 7 | 4.75 | 0.250 | 24.23 | -27.2, 75.7 |
| β-carotene | std,1 | 7 | 0.08 | -0.51, 0.67 | 0.791 | Yes (71%) | 0.64 | -14.55, 15.82 |  | 14 | 4.79 | 0.047 | 27.79 | -2.4, 57.97 |
|  | 2,3 | 7 | 0.08 | -0.51, 0.67 | 0.791 | Yes (71%) | 0.64 | -14.55, 15.82 |  | 14 | 4.79 | 0.050 | 27.79 | -2.4, 57.97 |
|  | 4,5 | 9 | 0.14 | -0.81, 1.08 | 0.778 | Yes (89%) | 1.74 | -15.61, 19.09 |  | 22 | 4.75 | 0.025 | 22.01 | 1.3, 42.72 |
|  | 6,7 | 9 | 0.14 | -0.81, 1.08 | 0.778 | Yes (89%) | 1.74 | -15.61, 19.09 |  | 22 | 4.75 | 0.021 | 22.01 | 1.3, 42.72 |
| Lutein | std,1 | 3 | 0.85 | -0.98, 2.68 | 0.361 | Yes (88%) | 12.71 | -46.12, 71.54 |  | 6 | 5.10 | 0.078 | 104.08 | -33.66, 241.82 |
|  | 2,3 | 3 | 0.85 | -0.98, 2.68 | 0.361 | Yes (88%) | 12.71 | -46.12, 71.54 |  | 6 | 5.10 | 0.078 | 104.08 | -33.66, 241.82 |
|  | 4,5 | 5 | 0.59 | -1.54, 2.72 | 0.587 | Yes (93%) | 14.17 | -37.16, 65.5 |  | 8 | 5.00 | 0.070 | 82.15 | -25.21, 189.5 |
|  | 6,7 | 5 | 0.59 | -1.54, 2.72 | 0.587 | Yes (93%) | 14.17 | -37.16, 65.5 |  | 8 | 5.00 | 0.068 | 82.15 | -25.21, 189.5 |
| Zeaxanthin | std,1 | - | - | - | - | - | - | - |  | 6 | 4.90 | 0.046 | 38.99 | 1.43, 76.55 |
|  | 2,3 | - | - | - | - | - | - | - |  | 6 | 4.90 | 0.042 | 38.99 | 1.43, 76.55 |
|  | 4,5 | 3 | -0.49 | -2.5, 1.51 | 0.630 | Yes (91%) | -2.42 | -49.36, 44.52 |  | 8 | 4.88 | 0.043 | 38.62 | 2.68, 74.56 |
|  | 6,7 | 3 | -0.49 | -2.5, 1.51 | 0.630 | Yes (91%) | -2.42 | -49.36, 44.52 |  | 8 | 4.88 | 0.042 | 38.62 | 2.68, 74.56 |
| n, number of data points included in the comparison; MPD, mean percent difference; SMD, standardised mean difference of random-effect model. *Sensitivity analysis number: std – standard meta-analysis; 1-6 – sensitivity analysis 1 to 6 (see main article and online supplementary Table S5 for details); †*P* value <0.05 indicates significance of the difference in composition between organic and conventional milk; ‡Heterogeneity and the I^2^ Statistic; §Magnitude of difference between organic and conventional samples (value <0 indicate higher concentration in CONV, value >0 indicate higher concentration in ORG); \|\|Ln ratio = Ln(ORG/CONV × 100%). | | | | | | | | | | | | | | |

| **Table A1 cont.** Results of meta-analysis comparing composition of organic (ORG) vs conventional (CONV) bovine milk using standard meta-analysis and sensitivity analyses protocols 1-7. | | | | | | | | | | | | | | |
| --- | --- | --- | --- | --- | --- | --- | --- | --- | --- | --- | --- | --- | --- | --- |
|  |  | **Weighted meta-analysis** | | | | | | |  | **Unweighted meta-analysis** | | | | |
| **Parameter** | **an*** | ***n*** | **SMD** | **95% CI** | ***P***† | **Heterogen.**‡ | **MPD**§ | **95% CI** |  | ***n*** | **Ln ratio**\|\| | ***P***† | **MPD**§ | **95% CI** |
| Vitamin A | std,1 | 4 | -2.59 | -7.81, 2.63 | 0.331 | Yes (99%) | -56.18 | -155.9, 43.5 |  | 10 | 4.43 | 0.019 | -27.31 | -67.2, 12.6 |
|  | 2,3 | 4 | -2.59 | -7.81, 2.63 | 0.331 | Yes (99%) | -56.18 | -155.9, 43.5 |  | 10 | 4.43 | 0.021 | -27.31 | -67.2, 12.6 |
|  | 4,5 | 4 | -2.59 | -7.81, 2.63 | 0.331 | Yes (99%) | -56.18 | -155.9, 43.5 |  | 16 | 4.48 | 0.007 | -18.71 | -43.9, 6.5 |
|  | 6,7 | 4 | -2.59 | -7.81, 2.63 | 0.331 | Yes (99%) | -56.18 | -155.9, 43.5 |  | 16 | 4.48 | 0.007 | -18.71 | -43.9, 6.5 |
| *Minerals and undesirable metals* | | | | |  |  |  |  |  |  |  |  |  |  |
| Copper (Cu) | std,1 | 8 | -0.57 | -1.16, 0.02 | 0.060 | Yes (60%) | -17.26 | -28.43, -6.10 |  | 10 | 4.50 | 0.049 | -12.37 | -25.04, 0.3 |
|  | 2,3 | 8 | -0.57 | -1.16, 0.02 | 0.060 | Yes (60%) | -17.26 | -28.43, -6.10 |  | 10 | 4.50 | 0.053 | -12.37 | -25.04, 0.3 |
|  | 4,5 | 9 | -0.54 | -1.04, -0.04 | 0.034 | Yes (55%) | -26.87 | -48.11, -5.62 |  | 11 | 4.44 | 0.025 | -20.67 | -40.58, -0.77 |
|  | 6,7 | 9 | -0.54 | -1.04, -0.04 | 0.034 | Yes (55%) | -26.87 | -48.11, -5.62 |  | 11 | 4.44 | 0.025 | -20.67 | -40.58, -0.77 |
| Iodine (I) | std,1 | 6 | -1.20 | -1.8, -0.59 | <0.001 | Yes (65%) | -73.85 | -115.2, -32.5 |  | 7 | 4.08 | 0.008 | -73.08 | -108.05, -38.1 |
|  | 2,3 | 10 | -1.37 | -1.78, -0.96 | <0.001 | Yes (55%) | -66.42 | -91.3, -41.6 |  | 11 | 4.11 | 0.001 | -66.61 | -89.08, -44.14 |
|  | 4,5 | 7 | -1.00 | -1.62, -0.38 | 0.002 | Yes (73%) | -63.04 | -103.9, -22.2 |  | 8 | 4.15 | 0.007 | -63.72 | -99.13, -28.31 |
|  | 6,7 | 11 | -1.23 | -1.68, -0.77 | <0.001 | Yes (67%) | -60.22 | -85.8, -34.7 |  | 12 | 4.16 | <0.001 | -60.91 | -84.27, -37.55 |
| Iron (Fe) | std,1 | 8 | 0.37 | 0.03, 0.71 | 0.034 | No (0%) | 20.18 | -0.1, 40.46 |  | 9 | 4.74 | 0.057 | 16.59 | -2.63, 35.81 |
|  | 2,3 | 8 | 0.37 | 0.03, 0.71 | 0.034 | No (0%) | 20.18 | -0.1, 40.46 |  | 9 | 4.74 | 0.057 | 16.59 | -2.63, 35.81 |
|  | 4,5 | 9 | 0.39 | 0.07, 0.7 | 0.016 | No (0%) | 32.05 | 2.71, 61.38 |  | 10 | 4.81 | 0.030 | 27.63 | 0, 55.26 |
|  | 6,7 | 9 | 0.39 | 0.07, 0.7 | 0.016 | No (0%) | 32.05 | 2.71, 61.38 |  | 10 | 4.81 | 0.028 | 27.63 | 0, 55.26 |
| n, number of data points included in the comparison; MPD, mean percent difference; SMD, standardised mean difference of random-effect model. *Sensitivity analysis number: std – standard meta-analysis; 1-6 – sensitivity analysis 1 to 6 (see main article and online supplementary Table S5 for details); †*P* value <0.05 indicates significance of the difference in composition between organic and conventional milk; ‡Heterogeneity and the I^2^ Statistic; §Magnitude of difference between organic and conventional samples (value <0 indicate higher concentration in CONV, value >0 indicate higher concentration in ORG); \|\|Ln ratio = Ln(ORG/CONV × 100%). | | | | | | | | | | | | | | |

| **Table A1 cont.** Results of meta-analysis comparing composition of organic (ORG) vs conventional (CONV) bovine milk using standard meta-analysis and sensitivity analyses protocols 1-7. | | | | | | | | | | | | | | |
| --- | --- | --- | --- | --- | --- | --- | --- | --- | --- | --- | --- | --- | --- | --- |
|  |  | **Weighted meta-analysis** | | | | | | |  | **Unweighted meta-analysis** | | | | |
| **Parameter** | **an*** | ***n*** | **SMD** | **95% CI** | ***P***† | **Heterogen.**‡ | **MPD**§ | **95% CI** |  | ***n*** | **Ln ratio**\|\| | ***P***† | **MPD**§ | **95% CI** |
| Potassium (K) | std,1 | 4 | 0.30 | -0.02, 0.62 | 0.063 | No (0%) | 4.49 | 1.35, 7.62 |  | 7 | 4.63 | 0.091 | 2.30 | -0.34, 4.94 |
|  | 2,3 | 4 | 0.30 | -0.02, 0.62 | 0.063 | No (0%) | 4.49 | 1.35, 7.62 |  | 7 | 4.63 | 0.093 | 2.30 | -0.34, 4.94 |
|  | 4,5 | 5 | 0.30 | 0.01, 0.59 | 0.045 | No (0%) | 5.10 | 2.39, 7.8 |  | 8 | 4.63 | 0.047 | 2.96 | 0.34, 5.58 |
|  | 6,7 | 5 | 0.30 | 0.01, 0.59 | 0.045 | No (0%) | 5.10 | 2.39, 7.8 |  | 8 | 4.63 | 0.046 | 2.96 | 0.34, 5.58 |
| Selenium (Se) | std,1 | 4 | -0.49 | -0.89, -0.1 | 0.015 | No (0%) | -21.42 | -48.93, 6.09 |  | 8 | 4.42 | 0.126 | -28.06 | -69.25, 13.13 |
|  | 2,3 | 4 | -0.49 | -0.89, -0.1 | 0.015 | No (0%) | -21.42 | -48.93, 6.09 |  | 8 | 4.42 | 0.127 | -28.06 | -69.25, 13.13 |
|  | 4,5 | 5 | -0.50 | -0.85, -0.14 | 0.006 | No (0%) | -18.06 | -40.36, 4.25 |  | 12 | 4.52 | 0.229 | -14.94 | -44.23, 14.36 |
|  | 6,7 | 5 | -0.50 | -0.85, -0.14 | 0.006 | No (0%) | -18.06 | -40.36, 4.25 |  | 12 | 4.52 | 0.233 | -14.94 | -44.23, 14.36 |
| *Other* | | | |  |  |  |  |  |  |  |  |  |  |  |
| Milk yield | std,1 | 32 | -1.23 | -1.64, -0.81 | <0.001 | Yes (96%) | -22.49 | -30.5, -14.5 |  | 81 | 4.44 | <0.001 | -19.57 | -23.62, -15.52 |
|  | 2,3 | 30 | -1.28 | -1.72, -0.84 | <0.001 | Yes (95%) | -23.51 | -31.9, -15.2 |  | 126 | 4.46 | <0.001 | -16.06 | -19.07, -13.06 |
|  | 4,5 | 36 | -1.20 | -1.58, -0.81 | <0.001 | Yes (95%) | -21.49 | -28.8, -14.2 |  | 96 | 4.44 | <0.001 | -18.94 | -22.73, -15.15 |
|  | 6,7 | 34 | -1.24 | -1.65, -0.84 | <0.001 | Yes (95%) | -22.33 | -29.9, -14.8 |  | 141 | 4.47 | <0.001 | -16.01 | -18.91, -13.11 |
| SCC | std,1 | 20 | 0.20 | -0.43, 0.82 | 0.537 | Yes (96%) | 8.19 | -12.98, 29.36 |  | 47 | 4.66 | 0.170 | 1.15 | -22.52, 24.82 |
|  | 2,3 | 19 | 0.16 | -0.49, 0.82 | 0.625 | Yes (96%) | 6.42 | -15.66, 28.5 |  | 71 | 4.68 | 0.025 | 5.44 | -10.61, 21.5 |
|  | 4,5 | 22 | 0.22 | -0.35, 0.8 | 0.446 | Yes (95%) | 8.11 | -11.13, 27.35 |  | 53 | 4.67 | 0.109 | 2.41 | -18.59, 23.41 |
|  | 6,7 | 21 | 0.20 | -0.41, 0.8 | 0.525 | Yes (95%) | 6.50 | -13.46, 26.47 |  | 77 | 4.68 | 0.013 | 5.98 | -8.83, 20.79 |
| n, number of data points included in the comparison; MPD, mean percent difference; SMD, standardised mean difference of random-effect model; SCC, somatic cell count. *Sensitivity analysis number: std – standard meta-analysis; 1-6 – sensitivity analysis 1 to 6 (see main article and online supplementary Table S5 for details); †*P* value <0.05 indicates significance of the difference in composition between organic and conventional milk; ‡Heterogeneity and the I^2^ Statistic; §Magnitude of difference between organic and conventional samples (value <0 indicate higher concentration in CONV, value >0 indicate higher concentration in ORG); \|\|Ln ratio = Ln(ORG/CONV × 100%). | | | | | | | | | | | | | | |

| **Table A1 cont.** Results of meta-analysis comparing composition of organic (ORG) vs conventional (CONV) bovine milk using standard meta-analysis and sensitivity analyses protocols 1-7. | | | | | | | | | | | | | | |
| --- | --- | --- | --- | --- | --- | --- | --- | --- | --- | --- | --- | --- | --- | --- |
|  |  | **Weighted meta-analysis** | | | | | | |  | **Unweighted meta-analysis** | | | | |
| **Parameter** | **an*** | ***n*** | **SMD** | **95% CI** | ***P***† | **Heterogen.**‡ | **MPD**§ | **95% CI** |  | ***n*** | **Ln ratio**\|\| | ***P***† | **MPD**§ | **95% CI** |
| *N compounds* | | | |  |  |  |  |  |  |  |  |  |  |  |
| Urea | std,1 | 7 | -0.42 | -1.04, 0.19 | 0.176 | Yes (70%) | -9.67 | -24.7, 5.36 |  | 11 | 4.53 | 0.085 | -8.75 | -19.64, 2.14 |
|  | 2,3 | 7 | -0.42 | -1.04, 0.19 | 0.176 | Yes (70%) | -9.67 | -24.7, 5.36 |  | 11 | 4.53 | 0.079 | -8.75 | -19.64, 2.14 |
|  | 4,5 | 9 | -0.72 | -1.39, -0.05 | 0.035 | Yes (76%) | -13.34 | -26.36, -0.32 |  | 19 | 4.47 | 0.002 | -16.08 | -25.22, -6.94 |
|  | 6,7 | 9 | -0.72 | -1.39, -0.05 | 0.035 | Yes (76%) | -13.34 | -26.36, -0.32 |  | 19 | 4.47 | 0.003 | -16.08 | -25.22, -6.94 |
| *Vitamins and antioxidants* | | | |  |  |  |  |  |  |  |  |  |  |  |
| α-tocopherol | std,1 | 9 | 0.74 | 0.01, 1.47 | 0.047 | Yes (81%) | 12.98 | 0.51, 25.45 |  | 17 | 4.70 | 0.013 | 11.68 | 2.52, 20.84 |
|  | 2,3 | 9 | 0.74 | 0.01, 1.47 | 0.047 | Yes (81%) | 12.98 | 0.51, 25.45 |  | 17 | 4.70 | 0.013 | 11.68 | 2.52, 20.84 |
|  | 4,5 | 11 | 0.61 | -0.08, 1.29 | 0.085 | Yes (81%) | 10.67 | -0.37, 21.72 |  | 25 | 4.71 | 0.003 | 12.20 | 4.46, 19.95 |
|  | 6,7 | 11 | 0.61 | -0.08, 1.29 | 0.085 | Yes (81%) | 10.67 | -0.37, 21.72 |  | 25 | 4.71 | 0.002 | 12.20 | 4.46, 19.95 |
| 2R (synthetic) | std,1 |  |  |  |  |  |  |  |  | 5 | 4.27 | 0.062 | -46.38 | -86.65, -6.11 |
| isomers of | 2,3 |  |  |  |  |  |  |  |  | 5 | 4.27 | 0.066 | -46.38 | -86.65, -6.11 |
| α-tocopherol | 4,5 |  |  |  |  |  |  |  |  | 6 | 4.38 | 0.095 | -32.17 | -75.26, 10.93 |
|  | 6,7 |  |  |  |  |  |  |  |  | 6 | 4.38 | 0.098 | -32.17 | -75.26, 10.93 |
| 3R (natural) | std,1 |  |  |  |  |  |  |  |  | 6 | 4.81 | 0.032 | 24.75 | 1.59, 47.9 |
| isomers of | 2,3 |  |  |  |  |  |  |  |  | 6 | 4.81 | 0.031 | 24.75 | 1.59, 47.9 |
| α-tocopherol | 4,5 | 3 | 0.87 | -1.48, 3.21 | 0.469 | Yes (93%) | 12.32 | -18.59, 43.23 |  | 7 | 4.76 | 0.068 | 18.90 | -3.77, 41.58 |
|  | 6,7 | 3 | 0.87 | -1.48, 3.21 | 0.469 | Yes (93%) | 12.32 | -18.59, 43.23 |  | 7 | 4.76 | 0.071 | 18.90 | -3.77, 41.58 |
| n, number of data points included in the comparison; MPD, mean percent difference; SMD, standardised mean difference of random-effect model. *Sensitivity analysis number: std – standard meta-analysis; 1-6 – sensitivity analysis 1 to 6 (see main article and online supplementary Table S5 for details); †*P* value <0.05 indicates significance of the difference in composition between organic and conventional milk; ‡Heterogeneity and the I^2^ Statistic; §Magnitude of difference between organic and conventional samples (value <0 indicate higher concentration in CONV, value >0 indicate higher concentration in ORG); \|\|Ln ratio = Ln(ORG/CONV × 100%). | | | | | | | | | | | | | | |

| **Table A1 cont.** Results of meta-analysis comparing composition of organic (ORG) vs conventional (CONV) bovine milk using standard meta-analysis and sensitivity analyses protocols 1-7. | | | | | | | | | | | | | | |
| --- | --- | --- | --- | --- | --- | --- | --- | --- | --- | --- | --- | --- | --- | --- |
|  |  | **Weighted meta-analysis** | | | | | | |  | **Unweighted meta-analysis** | | | | |
| **Parameter** | **an*** | ***n*** | **SMD** | **95% CI** | ***P***† | **Heterogen.**‡ | **MPD**§ | **95% CI** |  | ***n*** | **Ln ratio**\|\| | ***P***† | **MPD**§ | **95% CI** |
| *N compounds* | | | |  |  |  |  |  |  |  |  |  |  |  |
| Urea | std,1 | 7 | -0.42 | -1.04, 0.19 | 0.176 | Yes (70%) | -9.67 | -24.7, 5.36 |  | 11 | 4.53 | 0.085 | -8.75 | -19.64, 2.14 |
|  | 2,3 | 7 | -0.42 | -1.04, 0.19 | 0.176 | Yes (70%) | -9.67 | -24.7, 5.36 |  | 11 | 4.53 | 0.079 | -8.75 | -19.64, 2.14 |
|  | 4,5 | 9 | -0.72 | -1.39, -0.05 | 0.035 | Yes (76%) | -13.34 | -26.36, -0.32 |  | 19 | 4.47 | 0.002 | -16.08 | -25.22, -6.94 |
|  | 6,7 | 9 | -0.72 | -1.39, -0.05 | 0.035 | Yes (76%) | -13.34 | -26.36, -0.32 |  | 19 | 4.47 | 0.003 | -16.08 | -25.22, -6.94 |
| *Vitamins and antioxidants* | | | |  |  |  |  |  |  |  |  |  |  |  |
| α-tocopherol | std,1 | 9 | 0.74 | 0.01, 1.47 | 0.047 | Yes (81%) | 12.98 | 0.51, 25.45 |  | 17 | 4.70 | 0.013 | 11.68 | 2.52, 20.84 |
|  | 2,3 | 9 | 0.74 | 0.01, 1.47 | 0.047 | Yes (81%) | 12.98 | 0.51, 25.45 |  | 17 | 4.70 | 0.013 | 11.68 | 2.52, 20.84 |
|  | 4,5 | 11 | 0.61 | -0.08, 1.29 | 0.085 | Yes (81%) | 10.67 | -0.37, 21.72 |  | 25 | 4.71 | 0.003 | 12.20 | 4.46, 19.95 |
|  | 6,7 | 11 | 0.61 | -0.08, 1.29 | 0.085 | Yes (81%) | 10.67 | -0.37, 21.72 |  | 25 | 4.71 | 0.002 | 12.20 | 4.46, 19.95 |
| 2R (synthetic) | std,1 |  |  |  |  |  |  |  |  | 5 | 4.27 | 0.062 | -46.38 | -86.65, -6.11 |
| isomers of | 2,3 |  |  |  |  |  |  |  |  | 5 | 4.27 | 0.066 | -46.38 | -86.65, -6.11 |
| α-tocopherol | 4,5 |  |  |  |  |  |  |  |  | 6 | 4.38 | 0.095 | -32.17 | -75.26, 10.93 |
|  | 6,7 |  |  |  |  |  |  |  |  | 6 | 4.38 | 0.098 | -32.17 | -75.26, 10.93 |
| 3R (natural) | std,1 |  |  |  |  |  |  |  |  | 6 | 4.81 | 0.032 | 24.75 | 1.59, 47.9 |
| isomers of | 2,3 |  |  |  |  |  |  |  |  | 6 | 4.81 | 0.031 | 24.75 | 1.59, 47.9 |
| α-tocopherol | 4,5 | 3 | 0.87 | -1.48, 3.21 | 0.469 | Yes (93%) | 12.32 | -18.59, 43.23 |  | 7 | 4.76 | 0.068 | 18.90 | -3.77, 41.58 |
|  | 6,7 | 3 | 0.87 | -1.48, 3.21 | 0.469 | Yes (93%) | 12.32 | -18.59, 43.23 |  | 7 | 4.76 | 0.071 | 18.90 | -3.77, 41.58 |
| n, number of data points included in the comparison; MPD, mean percent difference; SMD, standardised mean difference of random-effect model. *Sensitivity analysis number: std – standard meta-analysis; 1-6 – sensitivity analysis 1 to 6 (see main article and online supplementary Table S5 for details); †*P* value <0.05 indicates significance of the difference in composition between organic and conventional milk; ‡Heterogeneity and the I^2^ Statistic; §Magnitude of difference between organic and conventional samples (value <0 indicate higher concentration in CONV, value >0 indicate higher concentration in ORG); \|\|Ln ratio = Ln(ORG/CONV × 100%). | | | | | | | | | | | | | | |

| Table A2. Standard meta-analysis results after exclusion of 20% of studies with the least precise treatment effects (sensitivity anlaysis 8, see main article and online supplementary Table S5 for details) for parameters shown in Fig. 3 and 4 of the main paper. | | | | | | | |
| --- | --- | --- | --- | --- | --- | --- | --- |
| **Parameter** | ***n*** | **SMD** | **95% CI** | ***P**** | **Heterogeneity**† | **MPD**‡ | **95% CI** |
| Milk yield | 26 | -1.24 | -1.62, -0.87 | <0.001 | Yes (95%) | -24.68 | -32.43, -16.92 |
| SFA | 15 | -0.18 | -0.67, 0.31 | 0.475 | Yes (74%) | -0.32 | -1.89, 1.25 |
| 12:0 (lauric acid) | 9 | -0.44 | -1.26, 0.38 | 0.294 | Yes (91%) | -4.75 | -12.68, 3.17 |
| 14:0 (myristic acid) | 10 | 0.21 | -0.52, 0.94 | 0.572 | Yes (88%) | 0.52 | -3.51, 4.55 |
| 16:0 (palmitic acid) | 11 | -0.47 | -1.14, 0.20 | 0.171 | Yes (88%) | -4.93 | -9.29, -0.56 |
| MUFA | 15 | 0.21 | -0.39, 0.81 | 0.493 | Yes (83%) | 0.48 | -4.10, 5.06 |
| OA (cis-9-18:1) | 8 | 0.21 | -0.88, 1.31 | 0.704 | Yes (94%) | 0.31 | -4.63, 5.25 |
| VA (trans-11-18:1) | 10 | 1.64 | 0.89, 2.39 | <0.001 | Yes (84%) | 62.04 | 6.43, 117.66 |
| PUFA | 15 | 0.65 | -0.06, 1.36 | 0.072 | Yes (87%) | 6.37 | -3.57, 16.31 |
| CLA (total) | 9 | 1.16 | 0.14, 2.19 | 0.027 | Yes (87%) | 30.10 | 4.60, 55.61 |
| CLA9 (cis-9-trans-11-18:2) | 11 | 0.75 | 0.38, 1.12 | <0.001 | Yes (64%) | 16.21 | 3.34, 29.07 |
| CLA10 (trans-10-cis-12-18:2) | - | - | - | - | - | - | - |
| n-3 FA | 10 | 1.55 | 0.89, 2.21 | <0.001 | Yes (76%) | 49.65 | 30.20, 69.10 |
| ALA (cis-9,12,15-18:3) | 17 | 2.44 | 1.64, 3.24 | <0.001 | Yes (93%) | 65.29 | 49.13, 81.44 |
| EPA (cis-5,8,11,14,17-20:5) | 6 | 1.18 | 0.32, 2.04 | 0.007 | Yes (93%) | 65.30 | 19.29, 111.32 |
| DPA (cis-7,10,13,16,19-22:5) | 4 | 1.18 | 0.14, 2.21 | 0.026 | Yes (93%) | 40.56 | 7.96, 73.17 |
| DHA (cis-4,7,10,13,16,19-22:6) | - | - | - | - | - | - | - |
| VLC n-3 PUFA§ | - | - | - | - | - | - | - |
| *n*, number of data points included in the comparison; SMD, standardised mean difference of fixed-effect model; MPD, mean percentage difference; SFA, saturated fatty acids; MUFA, monounsaturated fatty acids; OA, oleic acid; VA, vaccenic acid; PUFA, polyunsaturated fatty acids; FA, fatty acids; CLA, conjugated linoleic acid; ALA, α-linolenic acid; EPA, eicosapentaenoic acid; DPA, docosapentaenoic acid; DHA, docosahexaenoic acid; VLC n-3 PUFA, very long chain n-3 PUFA (EPA+DPA+DHA). **P* value <0.05 indicates significance of the difference in composition between organic and conventional milk; †Heterogeneity and the I^2^ Statistic; ‡Magnitude of difference between organic (ORG) and conventional (CONV) samples (value <0 indicate higher concentration in CONV, value >0 indicate higher concentration in ORG); §Calculated based on published fatty acids composition data. | | | | | | | |

| **Table A2 cont.** Standard meta-analysis results after exclusion of 20% of studies with the least precise treatment effects (sensitivity anlaysis 8, see main article and online supplementary Table S5 for details) for parameters shown in Fig. 3 and 4 of the main paper. | | | | | | | |
| --- | --- | --- | --- | --- | --- | --- | --- |
| **Parameter** | ***n*** | **SMD** | **95% CI** | ***P**** | **Heterogeneity**† | **MPD**‡ | **95% CI** |
| n-6 FA | 10 | -0.50 | -1.10, 0.10 | 0.103 | Yes (78%) | -7.88 | -17.78, 2.02 |
| LA (cis-9,12-18:2) | 10 | -0.95 | -1.98, 0.08 | 0.071 | Yes (94%) | -13.75 | -24.02, -3.48 |
| AA (cis-5,8,11,14-20:4) | 4 | -0.77 | -1.78, 0.24 | 0.137 | Yes (93%) | -16.86 | -28.38, -5.34 |
| LA/ALA ratio§ | - | - | - | - | - | - | - |
| n-6/n-3 ratio | 6 | -1.36 | -2.43, -0.28 | 0.013 | Yes (80%) | -61.65 | -117.62, -5.67 |
| n-3/n-6 ratio | 4 | 1.31 | 0.67, 1.94 | <0.001 | Yes (60%) | 70.91 | 24.37, 117.44 |
| Atherogenicity index§ | - | - | - | - | - | - | - |
| Thrombogenicity index§ | - | - | - | - | - | - | - |
| α-tocopherol | 7 | 0.62 | -0.13, 1.36 | 0.103 | Yes (82%) | 14.66 | -1.25, 30.58 |
| Carotenoids | 4 | 0.91 | -0.82, 2.64 | 0.304 | Yes (91%) | 40.25 | -46.02, 126.52 |
| β-carotene | 6 | 0.13 | -0.55, 0.80 | 0.716 | Yes (77%) | 1.15 | -16.78, 19.08 |
| Lutein | - | - | - | - | - | - | - |
| Zeaxanthin | - | - | - | - | - | - | - |
| Iodine (I) | 5 | -1.16 | -1.80, -0.53 | <0.001 | Yes (71%) | -55.51 | -80.54, -30.48 |
| Iron (Fe) | 6 | 0.42 | 0.06, 0.77 | 0.021 | No (0%) | 16.31 | 5.07, 27.56 |
| Selenium (Se) | 3 | -0.52 | -0.93, -0.10 | 0.014 | No (0%) | -26.94 | -62.71, 8.83 |
| Urea | 6 | -0.37 | -1.04, 0.30 | 0.284 | Yes (75%) | -3.64 | -14.65, 7.36 |
| *n*, number of data points included in the comparison; SMD, standardised mean difference of fixed-effect model; MPD, mean percentage difference; FA, fatty acids; LA, linoleic acid; AA, arachidonic acid; ALA, α-linolenic acid; SCC, somatic cell count. **P* value <0.05 indicates significance of the difference in composition between organic and conventional milk; †Heterogeneity and the I^2^ Statistic; ‡Magnitude of difference between organic (ORG) and conventional (CONV) samples (value <0 indicate higher concentration in CONV, value >0 indicate higher concentration in ORG); §Calculated based on published fatty acids composition data. | | | | | | | |
