# Supplementary material for: Higher PUFA and n-3 PUFA, conjugated linoleic acid, α-tocopherol and iron, but lower iodine and selenium concentrations in organic milk: a systematic literature review and meta- and redundancy analyses
Source: Br J Nutr. 2016 Mar 28;115(6):1043–60. doi: 10.1017/S0007114516000349 (PMC4838834; doi:10.1017/S0007114516000349)
Supplement: Supplementary file 1 [file S0007114516000349sup.zip › S0007114516000349sup002.docx]

Higher PUFA and omega-3 PUFA, CLA, α-tocopherol and iron, but lower iodine and selenium concentrations in organic milk: A Systematic Literature Review and Meta- and Redundancy Analyses

SUPPLEMENTARY DATA

**TABLE OF CONTENTS**

[1. INFORMATION ABOUT PAPERS INCLUDED IN THE SYSTEMATIC REVIEW AND THE META-ANALYSIS 4](#_Toc426549106)

[**Table S1.** List of papers included in the systematic review and the meta-analysis. 4](#_Toc426549107)

[**Figure S1.** Number of papers included in the systematic review and the meta-analysis by year of publication. 15](#_Toc426549108)

[**Figure S2.** Number of papers included in the systematic review and the meta-analysis by location of the experiment (country). 16](#_Toc426549109)

[**Table S2.** Study type, location, product and animal species information for studies included in the systematic review and the meta-analysis. 17](#_Toc426549110)

[**Table S3.** Production systems information for studies with more than two systems included in the meta-analysis. 25](#_Toc426549111)

[**Table S4.** Information extracted from papers and included in the database used for meta-analysis. 29](#_Toc426549112)

[**Table S5.** Summary of inclusion criteria used in the standard and the sensitivity analyses carried out. 30](#_Toc426549113)

[**Table S6.** List of composition parameters included in the meta-analysis. 31](#_Toc426549114)

[**Table S7.** List of composition parameters excluded from the meta-analysis. 32](#_Toc426549115)

[2. ADDITIONAL RESULTS 34](#_Toc426549116)

[**Table S8.** Basic information/statistics on the publications/data used for meta-analyses of composition parameters included in Fig. 2 and 3 in the main paper. 35](#_Toc426549117)

[**Table S9.** Mean percentage differences (MPD) and confidence intervals (CI) calculated using the data included in standard meta-analyses and sensitivity analysis 1 of composition parameters shown in Fig. 2 and 3 of the main paper (MPDs are also shown as symbols in Fig. 2). 37](#_Toc426549118)

[**Table S10.** Mean values and confidence intervals (CI) calculated using the data for all papers reporting means of composition parameters shown in Fig. 2 and 3 of the main paper. 39](#_Toc426549119)

[**Table S11.** Meta-analysis results for addition composition parameters for which significant differences were detected by the standard meta-analysis or one of the sensitivity analyses (see also Appendix Table A1 and A2 for results). 41](#_Toc426549120)

[**Figure S3.** Forest plot showing the results of studies examining the milk yield in organic and conventional bovine milk 43](#_Toc426549121)

[**Figure S4.** Forest plot showing the results of studies examining the saturated fatty acids (SFA) in organic and conventional bovine milk 44](#_Toc426549122)

[**Figure S5.** Forest plot showing the results of studies examining the 12:0 fatty acid (lauric acid) in organic and conventional bovine milk 45](#_Toc426549123)

[**Figure S6.** Forest plot showing the results of studies examining the 14:0 fatty acid (myristic acid) in organic and conventional bovine milk 46](#_Toc426549124)

[**Figure S7.** Forest plot showing the results of studies examining the 16:0 fatty acid (palmitic acid) in organic and conventional bovine milk 47](#_Toc426549125)

[**Figure S8.** Forest plot showing the results of studies examining the monounsaturated fatty acids (MUFA) in organic and conventional bovine milk 48](#_Toc426549126)

[**Figure S9.** Forest plot showing the results of studies examining the oleic fatty acid (cis-9-18:1, OA) in organic and conventional bovine milk 49](#_Toc426549127)

[**Figure S10.** Forest plot showing the results of studies examining the vaccenic fatty acid (trans-11-18:1, VA) in organic and conventional bovine milk 50](#_Toc426549128)

[**Figure S11.** Forest plot showing the results of studies examining the polyunsaturated fatty acids (PUFA) in organic and conventional bovine milk 51](#_Toc426549129)

[**Figure S12.** Forest plot showing the results of studies examining the total conjugated linoleic fatty acids (CLA total) in organic and conventional bovine milk 52](#_Toc426549130)

[**Figure S13.** Forest plot showing the results of studies examining the cis-9-trans-11-18:2 conjugated linoleic fatty acids (CLA) in organic and conventional bovine milk 53](#_Toc426549131)

[**Figure S14.** Forest plot showing the results of studies examining the trans-10-cis-12-18:2 conjugated linoleic fatty acids (CLA) in organic and conventional bovine milk 54](#_Toc426549132)

[**Figure S15.** Forest plot showing the results of studies examining the omega-3 fatty acids (n-3) in organic and conventional bovine milk 55](#_Toc426549133)

[**Figure S16.** Forest plot showing the results of studies examining the α-linolenic fatty acid (cis-9,12,15-18:3, ALA) in organic and conventional bovine milk 56](#_Toc426549134)

[**Figure S17.** Forest plot showing the results of studies examining the eicosapentaenoic fatty acid (cis-5,8,11,14,17-20:5, EPA) in organic and conventional bovine milk 57](#_Toc426549135)

[**Figure S18.** Forest plot showing the results of studies examining the docosapentaenoic fatty acid (cis-7,10,13,16,19-22:5, DPA) in organic and conventional bovine milk 58](#_Toc426549136)

[**Figure S19.** Forest plot showing the results of studies examining the docosahexaenoic fatty acid (cis-4,7,10,13,16,19-22:6, DHA) in organic and conventional bovine milk 59](#_Toc426549137)

[**Figure S20.** Forest plot showing the results of studies examining the omega-6 fatty acids (n-6) in organic and conventional bovine milk 60](#_Toc426549138)

[**Figure S21.** Forest plot showing the results of studies examining the linoleic fatty acid (cis-9,12-18:2, LA) in organic and conventional bovine milk 61](#_Toc426549139)

[**Figure S22.** Forest plot showing the results of studies examining the arachidonic fatty acid (cis-5,8,11,14-20:4, AA) in organic and conventional bovine milk 62](#_Toc426549140)

[**Figure S23.** Forest plot showing the results of studies examining the omega-6/omega-3 fatty acids ratio (n-6/n-3) in organic and conventional bovine milk 63](#_Toc426549141)

[**Figure S24.** Forest plot showing the results of studies examining the omega-3/omega-6 fatty acids ratio (n-3/n-6) in organic and conventional bovine milk 64](#_Toc426549142)

[**Figure S25.** Forest plot showing the results of studies examining the α-tocopherol in organic and conventional bovine milk 65](#_Toc426549143)

[**Figure S26.** Forest plot showing the results of studies examining the total carotenoids in organic and conventional bovine milk 66](#_Toc426549144)

[**Figure S27.** Forest plot showing the results of studies examining the β-carotene in organic and conventional bovine milk 67](#_Toc426549145)

[**Figure S28.** Forest plot showing the results of studies examining the lutein in organic and conventional bovine milk 68](#_Toc426549146)

[**Figure S29.** Forest plot showing the results of studies examining the iodine (I) in organic and conventional bovine milk 69](#_Toc426549147)

[**Figure S30.** Forest plot showing the results of studies examining the iron (Fe) in organic and conventional bovine milk 70](#_Toc426549148)

[**Figure S31.** Forest plot showing the results of studies examining the selenium (Se) in organic and conventional bovine milk 71](#_Toc426549149)

[**Figure S32.** Forest plot showing the results of studies examining the urea in organic and conventional bovine milk 72](#_Toc426549150)

[**Figure S33.** Forest plot showing the results of studies examining the somatic cell count (SCC) in organic and conventional bovine milk 73](#_Toc426549151)

[**Table S12.** Results of the standard meta-analysis and sensitivity analysis 1 for parameters where none of the protocols identified significant effects. 74](#_Toc426549152)

[**Table S13.** Results of the statistical test for publication bias reported in Table 1 of the main paper. 78](#_Toc426549153)

[**Figure S34**. Bi-plot derived from the redundancy analysis showing the relationship between milk composition parameters 80](#_Toc426549154)

[**Figure S35**. Results of standard meta-analysis and sensitivity analysis 1 for milk yield, fat composition and somatic cells in goat, sheep and buffalo milk 81](#_Toc426549155)

[**Table S14.** Mean percentage differences (MPD) for individual studies (study ID in parentheses, see Table S1 for references) calculated using the data for goat, sheep and buffalo milk and cheese of composition parameters shown in Fig. 2 and 3 of the main paper. 82](#_Toc426549156)

[**Table S15.** Mean percentage differences (MPD) for individual studies (study ID in parentheses, see Table S1 for references) calculated using the data for bovine dairy products of composition parameters shown in Fig. 2 and 3 of the main paper. 83](#_Toc426549157)

[3. ADDITIONAL DISCUSSION 86](#_Toc426549158)

[4. ADDITIONAL REFERENCES 88](#_Toc426549159)

# 1. INFORMATION ABOUT PAPERS INCLUDED IN THE SYSTEMATIC REVIEW AND THE META-ANALYSIS

| **Table S1.** List of papers included in the systematic review and the meta-analysis. | | |
| --- | --- | --- |
| **ID** | **Reference** |  |
| 69 | Burkitt LL, Wales WJ, McDonald JW et al. (2007) Comparing irrigated biodynamic and conventionally managed dairy farms. 2. Milk production and composition and animal health. Aust J Exp Agric 47, 489-494. | * |
| 125 | Di Renzo L, Di Pierro D, Bigioni M et al. (2007) Is antioxidant plasma status in humans a consequence of the antioxidant food content influence? Eur Rev Med Pharmacol Sci 11, 185-192. |  |
| 153 | Aulrich K & Molkentin J (2009) Potential of Near infrared Spectroscopy for differentiation of organically and conventionally produced milk. Landbauforschung Volkenrode 59, 301-307. | * |
| 155 | Bergamo P, Fedele E, Iannibelli L et al. (2003) Fat-soluble vitamin contents and fatty acid composition in organic and conventional Italian dairy products. Food Chem 82, 625-631. | ‡ |
| 157 | Butler G, Collomb M, Rehberger B et al. (2009) Conjugated linoleic acid isomer concentrations in milk from high and low input management dairy systems. J Sci Food Agric 89, 697-705. | * |
| 158 | Butler G, Nielsen JH, Slots T et al. (2008) Fatty acid and fat soluble antioxidant concetrations in milk from high and low input conventional and organic systems: seasonal variation. J Sci Food Agric 88, 1431-1441. | * |
| 160 | Collomb M, Bisig W, Butikofer U et al. (2008) Fatty acid composition of mountain milk from Switzerland: comparison of organic and integrated farming systems. Int Dairy J 18, 976-982. | * |
| 161 | Ellis KA, Innocent G, Grove-White D et al. (2006) Comparing the fatty acid composition of organic and conventional milk. J Dairy Sci 89, 1938-1950. | * |
| 162 | Ellis KA, Monteiro A, Innocent GT et al. (2007) Investigation of the vitamins A and E and beta-carotene content in milk from UK organic and conventional dairy farms. J Dairy Res 74, 484-491. | * |
| 169 | Hermansen JE, Badsberg JH, Kristensen T et al. (2005) Major and trace elements in organically or conventionally produced milk. J Dairy Res 72, 362-368. | * |
| 174 | Hoikkala A, Mustonen E, Saastamoinen I et al. (2007) High levels of equol in organic skimmed Finnish cow milk. Mol Nutr Food Res 51, 782-786. | * |
| 176 | Jahreis G, Fritsche J & Steinhart H (1996) Monthly variations of milk composition with special regard to fatty acids depending on season and farm management systems - conventional versus ecological. Fett/Lipid 98, 356-359. |  |
| 178 | Kraft J, Collomb M, Mockel P et al. (2003) Differences in CLA isomer distribution of cow's milk lipids. Lipids 38, 657-664. | * |
| 190 | Prandini A, Sigolo S & Piva G (2009) Conjugated linoleic acid (CLA) and fatty acid composition of milk, curd and Grana Padano cheese in conventional and organic farming systems. J Dairy Res 76, 178-282. | *‡ |
| 191 | Santos JSD, Beck L, Walter M et al. (2005) Nitrate and nitrite in milk produced by conventional and organic systems. Cienc Tecnol Aliment 25, 304-309. | * |
| 196 | Vicini J, Etherton T, Kris-Etherton P et al. (2008) Survey of retail milk composition as affectd by label claims regarding farm management practices. J Am Diet Assoc 108, 1109-1203. | * |
| 205 | Roesch M, Doherr MG & Blum JW (2006) Management, feeding, production, reproduction and udder health on organic and conventional Swiss dairy farms. Schweiz Arch Tierheilkd 148, 387-395. |  |
| 207 | Müller U & Sauerwein H (2010) A comparison of somatic cell count between organic and conventional dairy cow herds in West Germany stressing dry period related changes. Livest Sci 127, 30-37. | * |
| ID, Paper unique identification number. *Paper included in standard meta-analysis; †Paper with data on goat, sheep or buffalo milk or dairy products; ‡Paper with data on bovine dairy products. | | |

| **Table S1 cont.** List of papers included in the systematic review and the meta-analysis. | | |
| --- | --- | --- |
| **ID** | **Reference** |  |
| 216 | Sundberg T, Berglund B, Rydhmer L et al. (2009) Fertility, somatic cell count and milk production in Swedish organic and conventional dairy herds. Livest Sci 126, 176-182. |  |
| 217 | Vetter W & Schröder M (2010) Concentrations of phytanic acid and pristanic acid are higher in organic than in conventional dairy products from the German market. Food Chem 119, 746-752. | ‡ |
| 229 | Malmauret L, Parent-Massin D, Hardy JL et al. (2002) Contaminants in organic and conventional foodstuffs in France. Food Addit Contam Part A Chem Anal Control 19, 524-532. |  |
| 235 | Ghidini S, Zanardi E, Battaglia A et al. (2005) Comparison of contaminant and residue levels in organic and conventional milk and meat products from northern Italy. Food Addit Contam Part A Chem Anal Control 22, 9-14. |  |
| 257 | Zagorska J, Ciprovica I & Karklina D (2007) Heavy metals in organic milk. In Case studies in Food Safety and Environmental Health, pp. 75-79 [P Ho and VM Cortez, editors]: Springer US. | * |
| 266 | Lund P (1991) Characterization of alternatively produced milk. Milchwissenschaft 46, 166-169. |  |
| 293 | Arnold R (1984) A comparison of quality of liquid milk produced by conventional or alternative farming systems. Arch Lebensmittelhyg 35, 66-69. |  |
| 309 | Bloksma J, Adriaansen-Tennekes R, Huber M et al. (2008) Comparison of organic and conventional raw milk quality in The Netherlands. Biol Agric Hortic 26, 69-83. | * |
| 322 | Lavrencic A, Levart A & Salobir J (2007) Fatty acid composition of milk produced in organic and conventional dairy herds in Italy and Slovenia. Ital J Anim Sci 6, 437-439. | * |
| 329 | Olivo CJ, Beck LI, Gabbi AM et al. (2005) Composition and somatic cell count of milk in conventional and agro‐ecological farms: a comparative study in Depressão Central, Rio Grande do Sul state, Brazil. Livest Res Rural Dev 17, 72-78. | * |
| 350 | Molkentin J & Giesemann A (2007) Differentiation of organically and conventionally produced milk by stable isotope and fatty acid anlaysis. Anal Bioanal Chem 388, 297-305. |  |
| 352 | Nauta WJ, Veerkamp RF, Brascamp EW et al. (2006) Genotype by environment interaction for milk production traits between organic and conventional dairy cattle production in the Netherlands. J Dairy Sci 89, 2729-2737. | * |
| 353 | Nauta WJ, Baars T & Bovenhuis H (2006) Converting to organic dairy farming: consequences for production, somatic cell scores and calving interval of first parity Holstein cows. Livest Sci 99, 185-195. |  |
| 356 | Roesch M, Doherr MG & Blum JW (2005) Performance of dairy cows on Swiss farms with organic and integrated production. J Dairy Sci 88, 2462-2475. |  |
| 366 | O'Donnell AM, Spatny KP, Vicini JL et al. (2010) Survey of the fatty acid composition of retail milk differing in label claims based on production management practices. J Dairy Sci 93, 1918-1925. | * |
| 367 | Knoppler HO & Averdunk G (1986) A comparison of milk quality from conventional farms or from 'alternative' farms. Arch Lebensmittelhyg 37, 94-96. | * |
| 369 | Butler G, Stergiadis S, Seal C et al. (2011) Fat composition of organic and conventional retail milk in northeast England. J Dairy Sci 94, 24–36. | * |
| 383 | Schröder M & Vetter W (2010) GC/EI-MS Determination of the Diastereomer Distribution of Phytanic Acid in Food Samples. J Am Oil Chem Soc 88, 341-349. | ‡ |
| 384 | Schröder M, Yousefi F & Vetter W (2011) Investigating the day-to-day variations of potential marker fatty acids for organic milk in milk from conventionally and organically raised cows. Eur Food Res Technol 232, 167-174. |  |
| 385 | Tsiplakou E, Kotrotsios V, Hadjigeorgiou I et al. (2010) Differences in sheep and goats milk fatty acid profile between conventional and organic farming systems. J Dairy Res 77, 343-349. | † |
| ID, Paper unique identification number. *Paper included in standard meta-analysis; †Paper with data on goat, sheep or buffalo milk or dairy products; ‡Paper with data on bovine dairy products. | | |

| **Table S1 cont.** List of papers included in the systematic review and the meta-analysis. | | |
| --- | --- | --- |
| 386 | Tudisco R, Cutrignelli MI, Calabro S et al. (2010) Influence of organic systems on milk fatty acid profile and CLA in goats. Small Ruminant Res 88, 151-155. | † |
| 387 | Ellis K, McLean WG, Grove-White D et al. (2005) Studies comparing the composition of milk produced on organic and conventional dairy farms in the UK. Proceedings of the 4th SAFO Workshop: Systems development: quality and safety of organic livestock products, 41-45. | * |
| 388 | Ellis K (2005) Studies of the composition of milk produced on organic and conventional dairy farms. Org Stud Centr Techn Bull 8, 1-2. |  |
| 392 | Nielsen J, Lund-Nielsen T & Skibsted LH (2004) Higher antioxidant content in organic milk than in conventional milk due to feeding strategy. http://www.darcof.dk/enews/sep04/milk.html (accessed 4 September 2013) |  |
| 393 | Zagorska J & Ciprovica I (2008) The chemical composition of organic and conventional milk in Latvia. Proceedings of the 3rd Baltic Conference on Food Science and Technology, 10-14. |  |
| 394 | Hardeng F & Edge VL (2001) Mastitis, ketosis, and milk fever in 31 organic and 93 conventional Norwegian dairy herds. J Dairy Sci 84, 2673-2679. | * |
| 395 | Reksen O, Tverdal A & Ropstad E (1999) A comparative study of reproductive performance in organic and conventional dairy husbandry. J Dairy Sci 82, 2605-2610. |  |
| 396 | Allard G, Bregard P, Paquin D et al. (2002) Comparing milk components and quality on some organic and coventional dairy farms in Quebec. Proceedings of the 19th General Meeting of the European Grassland Federation. | * |
| 398 | Bennedsgaard TW, Thamsborg SM, Vaarst M et al. (2003) Eleven years of organic dairy production in Denmark: herd health and production related to time of conversion and compared to conventional production. (Special issue: Organic livestock production). Livest Prod Sci 80, 121-131. |  |
| 399 | Bennedsgaard TW, Klaas IC & Vaarst M (2010) Reducing use of antimicrobials - Experiences from an intervention study in organic dairy herds in Denmark. Livest Sci 131, 183-192. | * |
| 401 | Hovi M & Roderick S (2000) Mastitis and mastitis control strategies in organic milk. Cattle Practice 8, 259-264. |  |
| 402 | Molkentin J (2009) Authentication of Organic Milk Using delta C-13 and the alpha-Linolenic Acid Content of Milk Fat. J Agric Food Chem 57, 785-790. | * |
| 403 | Weber S, Pabst K, Ordolff D et al. (1993) Fünfjahrige Untersuchungen zur Umstellung auf ökologische Milcherzeugung. 2. Mitteilung: Milchqualität und Tiergesundheit. Zuchtungskunde 65, 338-347. | ‡ |
| 404 | Molkentin J (2008) Authentifizierung von Bio-Milch im Labor. Dtsch Milchwirtschaft 59, 873-874. | * |
| 405 | Miotello S (2007) Chemical, nutritional and technological characteristics of milk obtained from organic and conventional dairy farms located in the mountain area. Proceedings of the 58th Annual Meeting of the European Federation of Animal Science (EAAP). | * |
| 406 | Miotello S (2008) Chemical composition, fatty acids profile and sensory properties of cheese from organic and conventional milk. Proceedings of the 59th Annual Meeting of the European Federation of Animal Science (EAAP). | ‡ |
| 408 | Adriaansen-Tennekes R, Bloksma J, Huber MAS et al. (2005) Organic products and health. Results of milk research 2005: Universität Kassel/Witzenhausen. | * |
| 409 | Buchberger J (2002) Zur Milcheistug und Milchqualitaet aus oekologischer (biologischer) bzw. konventiontioneller Erzeugung. In Fleckvichwelt, Mitteilungen der Pruef und Besamungsstation, Muenchen-Grub, pp. 16-17. |  |
| 410 | Nogai K, Heide A, Grabowski NT et al. (2003) Production of pasteurised organic and conventional fresh milk. DMZ 124, 22-25. | * |
| 412 | Jonsson S (1996) Organic milk production - the first six years after changeover. Fakta-Husdjur 8, 4-11. |  |
| ID, Paper unique identification number. *Paper included in standard meta-analysis; †Paper with data on goat, sheep or buffalo milk or dairy products; ‡Paper with data on bovine dairy products. | | |

| **Table S1 cont.** List of papers included in the systematic review and the meta-analysis. | | |
| --- | --- | --- |
| 413 | Pellerin D, Allard G, Allard Y et al. (1997) Production laitière biologique: résultats d'un groupe de ferme de Lotbinière. Proceedings of the 21st Symposium sur les bovins laitiers, 49-60. |  |
| 414 | Stergiadis S, Leifert C, Seal C et al. (2012) Effect of Feeding Intensity and Milking System on Nutritionally Relevant Milk Components in Dairy Farming Systems in the North East of England. J Agric Food Chem 60, 7270−7281. | * |
| 418 | Bakutis B (2007) Quality analysis of milk production conditions in organic and conventional farms. Veterinarija Ir Zootechnika 39, 3-8. |  |
| 419 | Butler G, Nielsen AL, Larsen MK et al. (2011) The effects of dairy management and processing on quality characteristics of milk and dairy products. NJAS Wagening J Life Sci 58, 97-102. | * |
| 420 | Larsen MK, Nielsen AL, Butler G et al. (2010) Milk quality as affected by feeding regimes in a country with climatic variation. J Dairy Sci 93, 2863-2873. | * |
| 455 | Florence ACR, da Silva RC, do Espirito Santo AP et al. (2009) Increased CLA content in organic milk fermented by bifidobacteria or yoghurt cultures. Dairy Sci Technol 89, 541-553. | *‡ |
| 456 | Revilla I, Luruena-Martinez MA, Blanco-Lopez MA et al. (2009) Changes in Ewe's Milk Composition in Organic versus Conventional Dairy Farms. Czech J Food Sci 27, S263-S266. | † |
| 457 | Popovic-Vranjes A, Krajinovic M, Kecman J et al. (2010) Comparison of fatty acid composition in conventional and organic milk. Mljekarstvo 60, 59-66. |  |
| 458 | Gruber L, Steinwender R, Guggenberger T et al. (2001) Comparison of organic and conventional farming on a grassland farm - 2(nd) Communication: Feed intake, milk yield, health and fertility parameters. Bodenkultur 52, 55-70. | * |
| 461 | Fanti MGN, de Almeida KE, Rodrigues AM et al. (2008) Contribution to the study of physicochemical characteristics and lipid fraction of organic milk. Cienc Tecnol Aliment 28, 259-265. | * |
| 464 | Gabryszuk M, Sloniewski K & Sakowski T (2008) Macro- and microelements in milk and hair of cows from conventional vs. organic farms. Anim Sci Pap Rep 26, 199-209. | * |
| 465 | Fall N, Forslund K & Emanuelson U (2008) Reproductive performance, general health, and longevity of dairy cows at a Swedish research farm with both organic and conventional production. Livest Sci 118, 11-19. |  |
| 467 | Fall N & Emanuelson U (2011) Fatty acid content, vitamins and selenium in bulk tank milk from organic and conventional Swedish dairy herds during the indoor season. J Dairy Res 78, 287-292. |  |
| 472 | Bilik K & Lopuszanska-Rusek M (2010) Effect of organic and conventional feeding of red-and-white cows on productivity and milk composition. Ann Anim Sci 10, 441-458. | * |
| 474 | Di Francia A, Masucci F, De Rosa G et al. (2007) Feeding management and milk production in organic and conventional buffalo farms. Ital J Anim Sci 6, 571-574. | † |
| 481 | Battaglini LM, Renna M, Garda A et al. (2009) Comparing milk yield, chemical properties and somatic cell count from organic and conventional mountain farming systems. Ital J Anim Sci 8, 384-386. |  |
| 487 | Carpio A, Rodriguez-Estevez V, Sanchez-Rodriguez M et al. (2010) Differentiation of organic goat's milk based on its hippuric acid content as determined by capillary electrophoresis. Electrophoresis 31, 2211-2217. | † |
| 547 | Man C, Maerescu C, Lorincz P et al. (2008) Research concerning the quantity and quality of the sheep milk obtained in organic farms. Bull UASVM Animal Sci Biotech 65, 457. | † |
| 551 | Hamilton C, Emanuelson U, Forslund K et al. (2006) Mastitis and related management factors in certified organic dairy herds in Sweden. Acta Vet Scand 48, 11. |  |
| 552 | Ellis KA, Innocent G, Mihm M et al. (2007) Dairy cow cleanliness and milk quality on organic and conventional farms in the UK. J Dairy Res 74, 302-310. | * |
| ID, Paper unique identification number. *Paper included in standard meta-analysis; †Paper with data on goat, sheep or buffalo milk or dairy products; ‡Paper with data on bovine dairy products. | | |

| **Table S1 cont.** List of papers included in the systematic review and the meta-analysis. | | |
| --- | --- | --- |
| 553 | Zwald AG, Ruegg PL, Kaneene JB et al. (2004) Management practices and reported antimicrobial usage on conventional and organic dairy farms. J Dairy Sci 87, 191-201. | * |
| 554 | Sato K, Bartlett PC, Erskine RJ et al. (2005) A comparison of production and management between Wisconsin organic and conventional dairy herds. Livest Prod Sci 93, 105-115. | * |
| 555 | Pol M & Ruegg PL (2007) Treatment practices and quantification of antimicrobial drug usage in conventional and organic dairy farms in Wisconsin. J Dairy Sci 90, 249-261. |  |
| 556 | Renna M, Garda A, Lussiana C et al. (2009) Chemical, Nutritional and Microbiological Characterization of Organic Fontina Pdo Cheese. Ital J Food Sci 21, 287-303. | *‡ |
| 576 | Emanuelson U & Fall N (2007) Vitamins and selenium in bulk tank milk of organic and conventional dairy farms. Proceedings of the 58th Annual Meeting of the European Association for Animal Production (EAAP), 1-35. | * |
| 577 | Popovic-Vranjes A, Savic M, Pejanovic R et al. (2011) The effect of organic milk production on certain milk quality parameters. Acta Vet (Beogr) 61, 415-421. |  |
| 588 | Kuczynska BA (2011) Bioactive components and technological parameters of milk produced at ecological and conventional farms. In Treatises Monographs. Warsaw, Poland: Warsaw University of Life Sciences. | * |
| 589 | Fievez V & Vlaeminck B (2006) Fatty acid composition in milk from Flemish conventional and organic dairy farm management systems. J Anim Sci 84, 60. |  |
| 590 | Malbe M, Otstavel T, Kodis I et al. (2010) Content of selected micro and macro elements in dairy cows’ milk in Estonia. Agron Res 8, 323-326. |  |
| 591 | Florence ACR, Beal C, Silva RC et al. (2012) Fatty acid profile, trans-octadecenoic, alpha-linolenic and conjugated linoleic acid contents differing in certified organic and conventional probiotic fermented milks. Food Chem 135, 2207-2214. | ‡ |
| 592 | Pattono D, Battaglini LM, Barberio A et al. (2009) Presence of synthetic antioxidants in organic and conventional milk. Food Chem 115, 285-289. | * |
| 593 | Bath SC, Button S & Rayman MP (2012) Iodine concentration of organic and conventional milk: implications for iodine intake. Br J Nutr 107, 935-940. |  |
| 594 | Luzardo OP, Almeida-Gonzalez M, Henriquez-Hernandez LA et al. (2012) Polychlorobiphenyls and organochlorine pesticides in conventional and organic brands of milk: Occurrence and dietary intake in the population of the Canary Islands (Spain). Chemosphere 88, 307-315. |  |
| 595 | Florence ACR, Oliveira RPS, Silva RC et al. (2012) Organic milk improves Bifidobacterium lactis counts and bioactive fatty acids contents in fermented milk. LWT - Food Sci Technol 49, 89-95. | ‡ |
| 596 | Dahl L, Opsahl JA, Meltzer HM et al. (2003) Iodine Concentration in Norwegian Milk and Dairy Products. Br J Nutr 90, 679-685. | * |
| 597 | Huszti S (2009) Cercetări preliminare pentru producerea brânzeturilor organice. PhD thesis, Universitatea Dunărea de Jos din Galati |  |
| 598 | McBride WD & Greene C (2007) A comparison of conventional and organic milk production systems in the U. S. Proceeding of the American Agricultural Economics Association Annual Meeting. |  |
| 599 | Fall N & Emanuelson U (2009) Milk yield, udder health and reproductive performance in Swedish organic and conventional dairy herds. J Dairy Res 76, 402-410. |  |
| 600 | Kuczyńska B, Puppel K, Gołȩbiewski M et al. (2012) Differences in whey protein content between cow's milk collected in late pasture and early indoor feeding season from conventional and organic farms in Poland. J Sci Food Agric 92, 2899–2904. |  |
| 601 | Baars T, Schroeder M, Kusche D et al. (2012) Phytanic acid content and SRR/RRR diastereomer ratio in milk from organic and conventional farms at low and high level of fodder input. Org Agric 2, 13-21. |  |
| ID, Paper unique identification number. *Paper included in standard meta-analysis; †Paper with data on goat, sheep or buffalo milk or dairy products; ‡Paper with data on bovine dairy products. | | |

| **Table S1 cont.** List of papers included in the systematic review and the meta-analysis. | | |
| --- | --- | --- |
| 602 | Koehler M, Fechner A, Leiterer M et al. (2012) Iodine content in milk from German cows and in human milk: new monitoring study. Trace Elem Electrolytes 29, 119-126. | * |
| 616 | Pirisi A, Piredda G, Sitzia M et al. (2001) Organic and conventional systems: Composition and cheese-making aptitude of Sarda ewes' milk. Proceedings of the Joint International Conference - Organic Meat and Milk from Ruminants, 143-146. | † |
| 617 | Bilancia MT, Caponio F, Summo C et al. (2011) Comparison between organic and conventional goat yoghurts marketed in Italy. Milchwissenschaft 66, 65-68. | † |
| 618 | Pilarczyk B, Pilarczyk R, Tomza-Marciniak A et al. (2011) Selenium concentrations in the serum and milk of cows from organic and conventional farms in West Pomerania. Tieraerztl Umsch 66, 248-253. |  |
| 626 | Benbrook CM, Butler G, Latif MA et al. (2013) Organic Production Enhances Milk Nutritional Quality by Shifting Fatty Acid Composition: A United States-Wide, 18-Month Study. PLoS ONE 8, e82429. | * |
| 627 | Capuano E, Elgersma A, Tres A et al. (2014) Phytanic and pristanic acid content in Dutch farm milk and implications for the verification of the farming management system. Int Dairy J 35, 21-24. | * |
| 628 | Carpio A, Bonilla-Valverde D, Arce C et al. (2013) Evaluation of hippuric acid content in goat milk as a marker of feeding regimen. J Dairy Sci 96, 5426-5434. | *† |
| 629 | Cicconi-Hogan KM, Gamroth M, Richert R et al. (2013) Associations of risk factors with somatic cell count in bulk tank milk on organic and conventional dairy farms in the United States. J Dairy Sci 96, 3689-3702. |  |
| 630 | Cicconi-Hogan KM, Gamroth M, Richert R et al. (2013) Risk factors associated with bulk tank standard plate count, bulk tank coliform count, and the presence of Staphylococcus aureus on organic and conventional dairy farms in the United States. J Dairy Sci 96, 7578-7590. |  |
| 631 | da Silva JB, Fagundes GM, Guimaraes Soares JP et al. (2013) Dairy goat health management and milk production on organic and conventional system in Brazil. Semin-Cinac Agrar 34, 1273-1279. | † |
| 663 | Adler SA, Jensen SK, Thuen E et al. (2013) Effect of silage botanical composition on ruminal biohydrogenation and transfer of fatty acids to milk in dairy cows. J Dairy Sci 96, 1135-1147. |  |
| 664 | Adler SA, Jensen SK, Govasmark E et al. (2013) Effect of short-term versus long-term grassland management and seasonal variation in organic and conventional dairy farming on the composition of bulk tank milk. J Dairy Sci 96, 5793-5810. |  |
| 665 | Almeida-Gonzalez M, Luzardo OP, Zumbado M et al. (2012) Levels of organochlorine contaminants in organic and conventional cheeses and their impact on the health of consumers: an independent study in the Canary Islands (Spain). Food Chem Toxicol 50, 4325-4332. | ‡ |
| 666 | Bergamo P, Luongo D, Maurano F et al. (2005) Butterfat fatty acids differentially regulate growth and differentiation in Jurkat T-cells. J Cell Biochem 96, 349-360. | ‡ |
| 667 | Brambilla G, Abate V, De Filippis SP et al. (2011) Polychlorodibenzodioxin and -furan (PCDD and PCDF) and dioxin-like polychlorobiphenyl (DL-PCB) congener levels in milk of grazing sheep as indicators of the environmental quality of rural areas. J Agric Food Chem 59, 8513-8517. | † |
| 668 | Butikofer U, Meyer J, Sieber R et al. (2008) Occurrence of the angiotensin-converting enzyme inhibiting tripeptides Val-Pro-Pro and Ile-Pro-Pro in different cheese varieties of Swiss origin. J Dairy Sci 91, 29-38. | ‡ |
| 669 | Hoac T, Lundh T, Purup S et al. (2007) Separation of selenium, zinc, and copper compounds in bovine whey using size exclusion chromatography linked to inductively coupled plasma mass spectrometry. J Agric Food Chem 55, 4237-4243. | ‡ |
| ID, Paper unique identification number. *Paper included in standard meta-analysis; †Paper with data on goat, sheep or buffalo milk or dairy products; ‡Paper with data on bovine dairy products. | | |

| **Table S1 cont.** List of papers included in the systematic review and the meta-analysis. | | |
| --- | --- | --- |
| 670 | Malissiova E, Tsakalof A, Arvanitoyannis IS et al. (2013) Monitoring Aflatoxin M1 levels in ewe's and goat's milk in Thessaly, Greece; potential risk factors under organic and conventional production schemes. Food Control 34, 241-248. | † |
| 671 | Luukkonen J, Kemppinen A, Karki M et al. (2005) The effect of a protective culture and exclusion of nitrate on the survival of enterohemorrhagic E. coli and Listeria in Edam cheese made from Finnish organic milk. Int Dairy J 15, 449-457. | * |
| 672 | Mullen KAE, Sparks LG, Lyman RL et al. (2013) Comparisons of milk quality on North Carolina organic and conventional dairies. J Dairy Sci 96, 6753-6762. | * |
| 673 | Pape-Zambito DA, Roberts RF & Kensinger RS (2010) Estrone and 17beta-estradiol concentrations in pasteurized-homogenized milk and commercial dairy products. J Dairy Sci 93, 2533-2540. | * |
| 674 | Prema D, Pilfold JL, Krauchi J et al. (2013) Rapid Determination of Total Conjugated Linoleic Acid Content in Select Canadian Cheeses by H-1 NMR Spectroscopy. J Agric Food Chem 61, 9915-9921. | ‡ |
| 675 | Rey-Crespo F, Miranda M & Lopez-Alonso M (2013) Essential trace and toxic element concentrations in organic and conventional milk in NW Spain. Food Chem Toxicol 55, 513-518. | * |
| 676 | Tsakiris IN, Tzatzarakis MN, Alegakis AK et al. (2013) Risk assessment scenarios of children's exposure to aflatoxin M1 residues in different milk types from the Greek market. Food Chem Toxicol 56, 261-265. | * |
| 678 | Vetter W, Laure S, Wendlinger C et al. (2012) Determination of furan fatty acids in food samples. J Am Oil Chem Soc 89, 1501-1508. | ‡ |
| 679 | Ptasinska-Marcinkiewicz J, Halagarda M & Fijorek K (2012) Physicochemical properties of conventional and organic milk available on Polish market - comparative analysis. Milchwissenschaft 67, 242-245. | * |
| 680 | Cimen M, Yildirim N, Dikici A et al. (2010) Seasonal variations of biochemical taste parameters in milks from conventional and environment - Friendly organic farming. Bulg J Agri Sci 16, 728-732. |  |
| 681 | Hanus O, Vorlicek Z, Sojkova K et al. (2008) A comparison of selected milk indicators in organic herds with conventional herd as reference. Folia Veterinaria 52, 155-159. |  |
| 682 | Kastelic M & Kompan D (2008) Milk production of Bovska sheep in conventional and organic farming system. Acta Agric Slovenica 92, 41-46. | † |
| 683 | Kompan D & Kastelic M (2009) Productivity of Slovenian Alpine goat in the conventional and organic farming system. Proceedings of the 36th ICAR Biennial Session Identification - Breeding, Production, Health and Recording of Farm Animals, 137-142. | † |
| 684 | Kuhnen S, Moacyr JR, Trevisan R et al. (2013) Carotenoid content in cow milk from organic and conventional farms in Southern Brazil. J Food Agri Environ 11, 221-224. | * |
| 686 | Sarubbi F, Polimeno F, Auriemma G et al. (2013) Effects of season calving and managements on lactating curves in two different farms (organic vs conventional) in buffalo cows. Open J Anim Sci 3, 83-87. | † |
| 687 | Selegovska E & Spruzs J (2008) Welfare of goats and the production of products in organic and conventional farms. Latv J Agron 10, 287-292. | † |
| 688 | Klir Z, Potocnik K, Antunovic Z et al. (2013) Comparison of milk production traits by Istrian pramenka between conventional and organic systems in Slovenia. Agric Conspec Sci 78, 271-274. | † |
| 689 | Cermanova I, Hanus O, Roubal P et al. (2011) Effect of organic farming on selected raw cow milk components and properties. Acta Univ Agric Silvic Mendelianae Brun 59, 81-92. | * |
| 690 | Gutierrez R, Rosell P, Vega S et al. (2013) Self and foreign substances in organic and conventional milk produced in the eastern region of Mexico. Food Nutr Sci 4, 586-593. |  |
| ID, Paper unique identification number. *Paper included in standard meta-analysis; †Paper with data on goat, sheep or buffalo milk or dairy products; ‡Paper with data on bovine dairy products. | | |

| **Table S1 cont.** List of papers included in the systematic review and the meta-analysis. | | |
| --- | --- | --- |
| 692 | Kusche D, Ruebesam K & Baars T (2010) Fatty acids and antioxidant profiles in summer milk from different biodynamic and conventional systems in Southern Germany. Proceedings of the 23th General Meeting of the European Grassland Federation - Grassland Science in Europe 15, 604-606. |  |
| 693 | Zagorska J & Ciprovica I (2005) The comparison of chemical pollution between organic and conventional milk. Proceedings of the Research for rural development: International scientific conference, 196-198. | * |
| 694 | Zagorska J, Ciprovica I & Mikelsone V (2007) Evaluation of antibodies concentration in cow's milk from different agricultural systems. LLU Raksti 18, 45-50. | * |
| 695 | Almeida M, Boada LD, Zumbado M et al. (2009) High exposure level to dioxin-like carcinogens through intake of commercial milk from the Canary Islands market (Spain). J Vet Pharmacol Ther 32, 194-195. | * |
| 696 | Jahreis G, Leiterer M & Fechner A (2007) Appropriate nutrition eliminates iodine deficiency : the contribution of milk, seafood and iodized table salt to the iodine supply in Germany. Präv Gesundheitsf 2, 179-184. | * |
| 697 | Kaffarnik S, Schröder M, Lehnert K et al. (2014) δ13C values and phytanic acid diastereomer ratios: combined evaluation of two markers suggested for authentication of organic milk and dairy products. Eur Food Res Technol 238, 819-827. | *‡ |
| 698 | Man C, Hicea S & Ciupe M (2009) Data regarding the nutritional, functional and sensory quality of bio milk. Bull UASVM Animal Sci Biotech 66, 119-125. | † |
| 699 | Martínez-Fernández A, Vicente F, Morales-Almaráz E et al. (2009) Effects of conventional versus organic management system on perennial ryegrass-white clover rotational grazing pastures: Grass allowance, milk yield and quality of grass and milk. Ir J Agric Food Res 48, 264. |  |
| 700 | Venturoso RC, Almeida KEd, Rodrigues AM et al. (2007) Determination of the physical-chemical composition of dairy products: exploratory study to compare the results obtained by classic methodology and by ultra-sound. Rev Bras Cienc Farm 43, 607-613. | * |
| 702 | Stergiadis S, Leifert C, Seal CJ et al. (2013) Individual cow variation on milk polyunsaturated fatty acids. Proc Nutr Soc 72, 109. |  |
| 703 | Jakobsen J & Saxholt E (2009) Vitamin D metabolites in bovine milk and butter. J Food Compos Anal 22, 472-478. | * |
| 704 | Gustafson GM, Salomon E, Jonsson S et al. (2003) Fluxes of K, P, and Zn in a conventional and an organic dairy farming system through feed, animals, manure, and urine - a case study at Ojebyn, Sweden. Eur J Agron 20, 89-99. | * |
| 705 | Florence ACR, Beal C, Silva RCd et al. (2014) Survival of three Bifidobacterium animalis subsp. lactis strains is related to trans-vaccenic and alpha -linolenic acids contents in organic fermented milks. LWT - Food Sci Technol 56, 290-295. | *‡ |
| 706 | Chotyakul N, Pateiro-Moure M, Martinez-Carballo E et al. (2014) Development of an improved extraction and HPLC method for the measurement of ascorbic acid in cows' milk from processing plants and retail outlets. Int J Food Sci Technol 49, 679-688. | * |
| 707 | Chotyakul N, Pateiro-Moure M, Saraiva JA et al. (2014) Simultaneous HPLC-DAD quantification of vitamins A and E content in raw, pasteurized, and UHT cow's milk and their changes during storage. Eur Food Res Technol 238, 535-547. | * |
| 708 | Kelly T, Butcher N, Harrington K et al. (2005) Organic-conventional dairy systems trial in New Zealand: four years' results. Proceedings of the First Scientific Conference of the International Society of Organic Agriculture Research (ISOFAR), held in Cooperation with the International Federation of Organic Agriculture Movements (IFOAM) and the National Association for Sustainable Agriculture, Australia (NASAA), 268-271. |  |
| ID, Paper unique identification number. *Paper included in standard meta-analysis; †Paper with data on goat, sheep or buffalo milk or dairy products; ‡Paper with data on bovine dairy products. | | |

| **Table S1 cont.** List of papers included in the systematic review and the meta-analysis. | | |
| --- | --- | --- |
| 709 | Muntean D (2011) Impact organic products from sheep's milk on human health compared to conventional. Bull UASVM Agric 68, 339-343. | † |
| 710 | Rehberger B, Bisig W, Eberhard P et al. (2007) Assessment of processing technologies which may improve the nutritional composition of dairy products - overview of progress. Proceedings of the 3rd International Congress of the European Integrated Project Quality Low Input Food (QLIF) - Improving sustainability in organic and low input food production systems, 384-387. | ‡ |
| 712 | Smiechowska M (2001) The nitrate intake with food of animal and plant origin. Acta Pol Toxicol 9, 115-123. | * |
| 713 | Zervas G, Koutsotolis K, Theodoropoulos G et al. (2000) Comparison of organic with conventional feeding systems of lactating dairy ewes in Greece. In EAAP Publication No. 97, pp. 107-111 [D Gagnaux and JR Poffet, editors]. Netherlands. | † |
| 714 | Cubon J, Foltys V, Hascik P et al. (2008) The raw milk quality from organic and conventional agriculture. Acta Univ Agric Silvic Mendelianae Brun 56, 25-30. |  |
| 715 | Bennedsgaard TW, Thamsborg SM, Aarestrup FM et al. (2006) Resistance to penicillin of Staphylococcus aureus isolates from cows with high somatic cell counts in organic and conventional dairy herds in Denmark. Acta Vet Scand 48, 24. |  |
| 716 | Berentsen PBM, Kovacs K & van Asseldonk MAPM (2012) Comparing risk in conventional and organic dairy farming in the Netherlands: an empirical analysis. J Dairy Sci 95, 3803-3811. | * |
| 717 | Bidokhti MRM, Traven M, Fall N et al. (2009) Reduced likelihood of bovine coronavirus and bovine respiratory syncytial virus infection on organic compared to conventional dairy farms. Vet J 182, 436-440. |  |
| 720 | Blank B, Schaub D, Paulsen HM et al. (2013) Comparison of performance and feeding parameters in organic and conventional dairy farms in Germany. Landbauforschung Volkenrode 63, 21-28. |  |
| 721 | Boutet P, Detilleux J, Motkin M et al. (2005) A comparison of somatic cell count and antimicrobial susceptibility of subclinical mastitis pathogens in organic and conventional dairy herds. Ann Med Vet 149, 173-182. |  |
| 722 | Brenninkmeyer C, Dippel S, Brinkmann J et al. (2013) Hock lesion epidemiology in cubicle housed dairy cows across two breeds, farming systems and countries. Prev Vet Med 109, 236-245. |  |
| 723 | Burgoyne D, Levallois R, Perrier JP et al. (1995) A comparison of the profitability of conventional and organic milk production systems in Quebec. Can J Agri Econ 43, 435-442. |  |
| 724 | Stene O, Thuen E, Haug A et al. (2002) Conjugated linoleic acid (CLA) content of milk from cows in two different production systems. Meieriposten 91, 118-119. |  |
| 725 | Slots T, Sorensen J & Nielsen JH (2009) Tocopherol, carotenoids and fatty acid composition in organic and conventional milk. DMZ 130, 47-50. |  |
| 726 | Butler LJ (2002) The economics of organic milk production in California: a comparison with conventional costs. Am J Alternative Agr 17, 83-91. |  |
| 727 | Cazer CL, Mitchell RM, Cicconi-Hogan KM et al. (2013) Associations between Mycobacterium avium subsp paratuberculosis antibodies in bulk tank milk, season of sampling and protocols for managing infected cows. BMC Vet Res 9, 1-7. |  |
| 728 | Cho S, Diez-Gonzalez F, Fossler CP et al. (2006) Prevalence of shiga toxin-encoding bacteria and shiga toxin-producing Escherichia coli isolates from dairy farms and county fairs. Vet Microbiol 118, 289-298. |  |
| 729 | Flysjo A, Cederberg C, Henriksson M et al. (2012) The interaction between milk and beef production and emissions from land use change - critical considerations in life cycle assessment and carbon footprint studies of milk. J Clean Prod 28, 134-142. |  |
| ID, Paper unique identification number. *Paper included in standard meta-analysis; †Paper with data on goat, sheep or buffalo milk or dairy products; ‡Paper with data on bovine dairy products. | | |

| **Table S1 cont.** List of papers included in the systematic review and the meta-analysis. | | |
| --- | --- | --- |
| 730 | Garmo RT, Waage S, Sviland S et al. (2010) Reproductive performance, udder health, and antibiotic resistance in mastitis bacteria isolated from Norwegian Red cows in conventional and organic farming. Acta Vet Scand 52, 11. | * |
| 731 | Guerci M, Knudsen MT, Bava L et al. (2013) Parameters affecting the environmental impact of a range of dairy farming systems in Denmark, Germany and Italy. J Clean Prod 54, 133-141. |  |
| 732 | Honorato LA, Machado Filho LCP, Barbosa Silveira ID et al. (2014) Strategies used by dairy family farmers in the south of Brazil to comply with organic regulations. J Dairy Sci 97, 1319-1327. | * |
| 733 | Kristensen T & Kristensen ES (1998) Analysis and simulation modelling of the production in Danish organic and conventional dairy herds. Livest Prod Sci 54, 55-65. | * |
| 734 | Kristensen T & Mogensen L (1999) Danish organic dairy cattle production systems - feeding and feed efficiency. Proceedings of the NJF-seminar Np. 303 - DARCOF Report No. 2, 173-178. | * |
| 735 | Kristensen T, Mogensen L, Knudsen MT et al. (2011) Effect of production system and farming strategy on greenhouse gas emissions from commercial dairy farms in a life cycle approach. Livest Sci 140, 136-148. |  |
| 736 | Mayen CD, Balagtas JV & Alexander CE (2010) Technology adoption and technical efficiency: organic and conventional dairy farms in the United States, 1 ed., pp. 181-195. New Mexico, USA: Department of Agricultural Economics and Agricultural Business, New Mexico State University. | * |
| 737 | McBride WD & Greene C (2009) Characteristics, costs, and issues for organic dairy farming. Economic Research Report Number 82. USA: Economic Research Service/United States Department of Agriculture. |  |
| 738 | McLeod KLM, Holmes CW, Morel PCH et al. (2008) Comparison of mastitis prevalence between an organic and a conventional dairy herd from 2004 to 2006. Proceedings of the New Zealand Society of Animal Production 68th Conference, 8-11. |  |
| 739 | Nielsen AH & Kristensen IS (2005) Nitrogen and phosphorus surpluses on Danish dairy and pig farms in relation to farm characteristics. In EAAP Publication No. 4/2005, 1 ed., pp. 97-107 [JE Hermansen and G Zervas, editors]. Netherlands. |  |
| 740 | Nordqvist M, Holtenius K & Sporndly R (2014) Methods for assessing phosphorus overfeeding on organic and conventional dairy farms. Animal 8, 286-292. |  |
| 741 | Ogini YO, Stonehouse DP & Clark EA (1999) Comparison of organic and conventional dairy farms in Ontario. Am J Alternative Agr 14, 122-128. |  |
| 742 | Richert RM, Cicconi KM, Gamroth MJ et al. (2013) Risk factors for clinical mastitis, ketosis, and pneumonia in dairy cattle on organic and small conventional farms in the United States. J Dairy Sci 96, 4269-4285. |  |
| 743 | Roesch M, G Doherr M, Scharen W et al. (2007) Subclinical mastitis in dairy cows in Swiss organic and conventional production systems. J Dairy Res 74, 86-92. |  |
| 744 | Rozzi P, Miglior F & Hand KJ (2007) A total merit selection index for Ontario organic dairy farmers. J Dairy Sci 90, 1584-1593. |  |
| 745 | Shadbolt N, Kelly T, Horne D et al. (2009) Comparisons between organic & conventional pastoral dairy farming systems: cost of production and profitability. Proceedings of the 17th International Farm Management Congress 13, 671-685. |  |
| 746 | Sholubi YO, Stonehouse DP & Clark EA (1997) Profile of organic dairy farming in Ontario. Am J Alternative Agr 12, 133-139. |  |
| 747 | Silva KE, Quinn AK, Morel PCH et al. (2005) A study of mastitis in two small experimental dairy herds managed either organically or conventionally, during one year. Proceedings of the New Zealand Society of Animal Production 65th conference, 148-152. |  |
| ID, Paper unique identification number. *Paper included in standard meta-analysis; †Paper with data on goat, sheep or buffalo milk or dairy products; ‡Paper with data on bovine dairy products. | | |

| **Table S1 cont.** List of papers included in the systematic review and the meta-analysis. | | |
| --- | --- | --- |
| 748 | Silverlas C & Blanco-Penedo I (2013) Cryptosporidium spp. in calves and cows from organic and conventional dairy herds. Epidemiol Infect 141, 529-539. |  |
| 749 | Steinwidder A & Guggenberger T (2003) Investigations on feed intake and nutrient supply of dairy cows as well as nutrient balance studies on farms in grassland regions of Austria. Bodenkultur 54, 49-66. | * |
| 750 | Werf HMGvd, Kanyarushoki C & Corson MS (2009) An operational method for the evaluation of resource use and environmental impacts of dairy farms by life cycle assessment. J Environ Manage 90, 3643-3652. | * |
| 751 | Valle PS, Lien G, Flaten O et al. (2007) Herd health and health management in organic versus conventional dairy herds in Norway. Livest Sci 112, 123-132. | * |
| 752 | Thatcher A, Petrovski K, Holmes C et al. (2008) A longitudinal study of mastitis on an experimental farm with two herds, one managed organically, the other conventionally. Proceedings of the 16th IFOAM Organic World Conference in Cooperation with the International Federation of Organic Agriculture Movements (IFOAM) and the Consorzio ModenaBio. Cultivating the future based on science. Volume 2: Livestock, socio-economy and cross disciplinary research in organic agriculture. Proceedings of the Second Scientific Conference of the International Society of Organic Agriculture Research (ISOFAR). 70-73. |  |
| 753 | Stiglbauer KE, Cicconi-Hogan KM, Richert R et al. (2013) Assessment of herd management on organic and conventional dairy farms in the United States. J Dairy Sci 96, 1290-1300. |  |
| 754 | Svensson C, Hessle A & Höglund J (2000) Parasite control methods in organic and conventional dairy herds in Sweden. Livest Prod Sci 66, 57-69. | * |
| 755 | Thomassen MA, van Calker KJ, Smits MCJ et al. (2008) Life cycle assessment of conventional and organic milk production in the Netherlands. Agric Sys 96, 95-107. | * |
| ID, Paper unique identification number. *Paper included in standard meta-analysis; †Paper with data on goat, sheep or buffalo milk or dairy products; ‡Paper with data on bovine dairy products. | | |

**
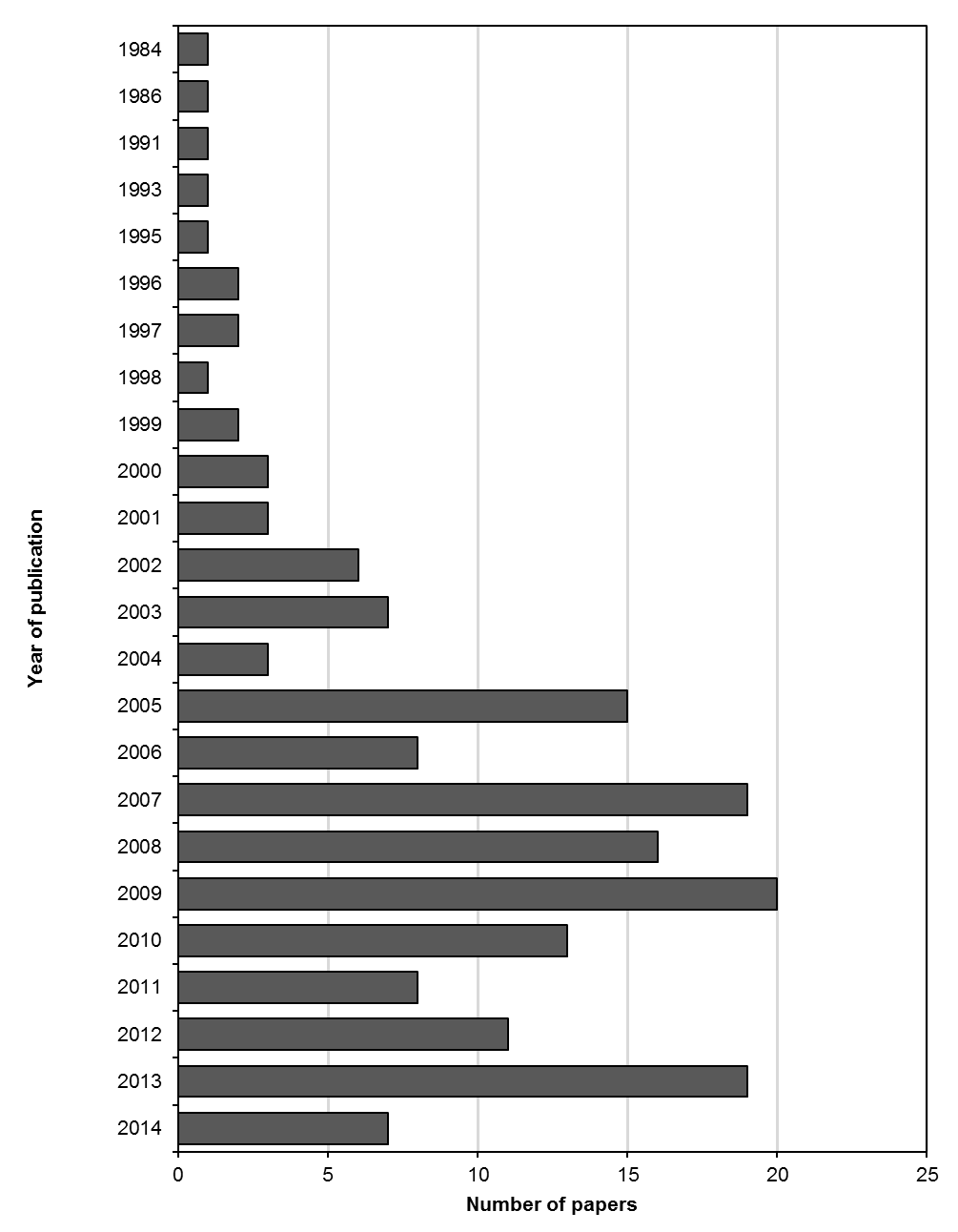
**

## **Figure S1.** Number of papers included in the systematic review and the meta-analysis by year of publication.

**
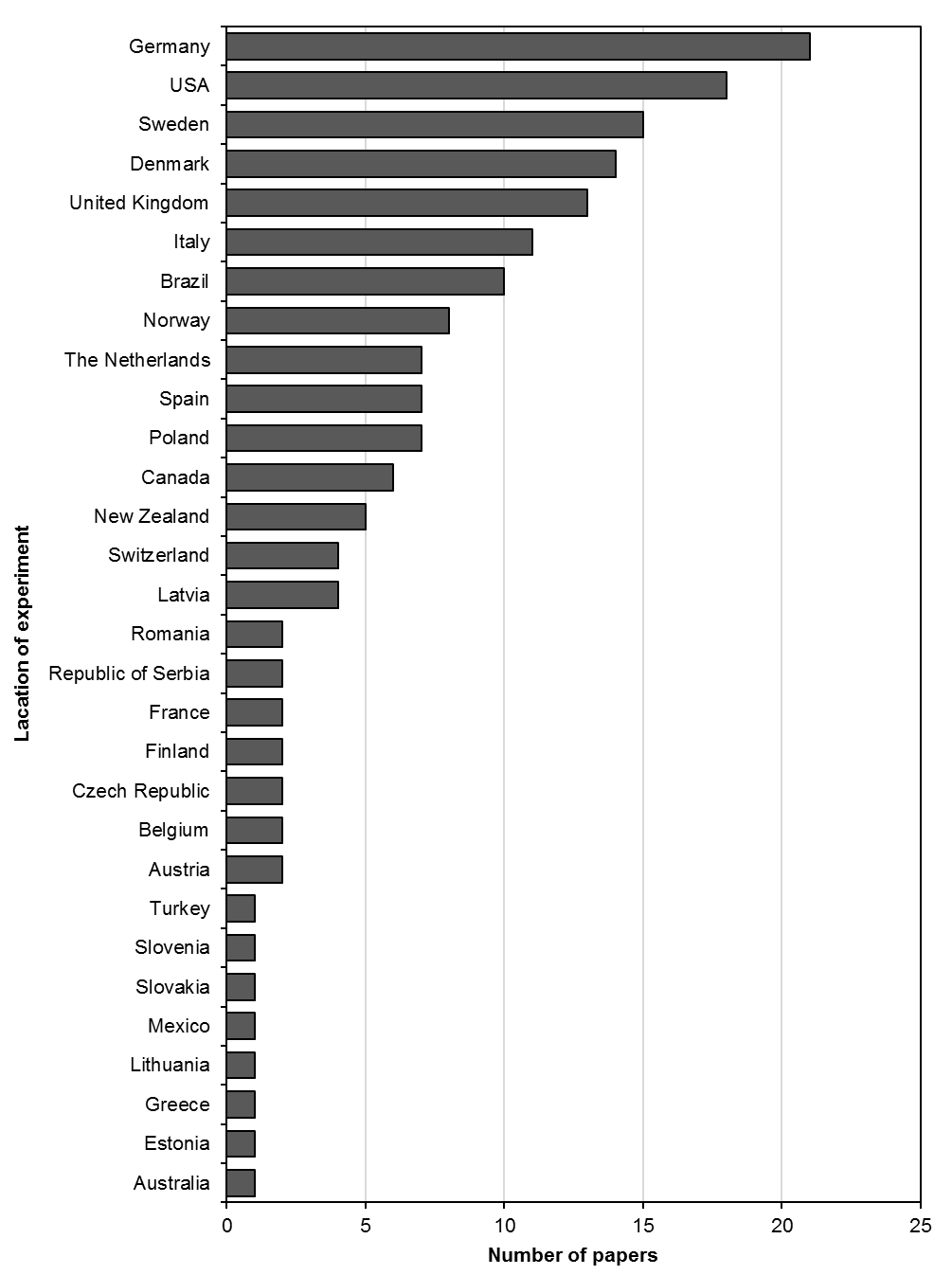
**

## **Figure S2.** Number of papers included in the systematic review and the meta-analysis by location of the experiment (country).

| **Table S2.** Study type, location, product and animal species information for studies included in the systematic review and the meta-analysis. | | | | |
| --- | --- | --- | --- | --- |
| **ID** | **ST** | **Location** | **Product** | **Animal species** |
| 69 | CF | Australia | milk | cow |
| 125 | BS | Italy | milk | cow |
| 153 | BS | Germany | milk | cow |
| 155 | BS | Italy | milk | cow |
|  |  |  | cheese (crescenza, fontina, mozzarella, parmigiano, ricotta)* | cow |
|  |  |  | butter* | cow |
|  |  |  | dairy products (milk, butter, cheeses)* | cow |
|  | CF | Italy | milk | buffalo* |
|  |  |  | cheese (mozzarella) | buffalo* |
| 157 | CF | United Kingdom | milk | cow |
| 158 | CF | United Kingdom | milk | cow |
| 160 | CF | Switzerland | milk | cow |
| 161 | CF | United Kingdom | milk | cow |
| 162 | CF | United Kingdom | milk | cow |
| 169 | CF | Denmark | milk | cow |
| 174 | BS | Finland | milk | cow |
| 176 | CF | Germany | milk | cow |
| 178 | CF | Germany | milk | cow |
| 190 | BS | Italy | curd* | cow |
|  |  |  | cheese* | cow |
|  |  |  | milk | cow |
| 191 | CF | Brazil | milk | cow |
| 192 | CF | Denmark | milk | cow |
| 194 | CF | Sweden | milk | cow |
| 196 | BS | USA | milk | cow |
| 205 | CF | Switzerland | milk | cow |
| 207 | CF | Germany | milk | cow |
| 216 | CF | Sweden | milk | cow |
| 217 | BS | Germany | cheese (crescenza, fontina, mozzarella, parmigiano, ricotta)* | cow |
| 229 | CF | France | milk | cow |
| 235 | CF | Italy | milk | cow |
| ID, Paper unique identification number (see Table S1 for references); ST, Study type (CF – Comparison of Farms, BS – Basket Study, EX – Controlled Experiment). *Data for products other than milk and animals other than cow was described in the main paper and summarised in Figure S35, Table S14 and S15. | | | | |

| **Table S2 cont.** Study type, location, product and animal species information for studies included in the systematic review and the meta-analysis. | | | | |
| --- | --- | --- | --- | --- |
| **ID** | **ST** | **Location** | **Product** | **Animal species** |
| 257 | CF | Latvia | milk | cow |
| 266 | CF | Denmark | milk | cow |
| 293 | BS | Germany | milk | cow |
| 309 | CF | The Netherlands | milk | cow |
| 322 | CF | Italy | milk | cow |
|  |  | Slovenia | milk | cow |
| 329 | CF | Brazil | milk | cow |
| 350 | BS | Germany | milk | cow |
|  | CF | Germany | milk | cow |
| 352 | CF | The Netherlands | milk | cow |
| 353 | CF | The Netherlands | milk | cow |
| 356 | CF | Switzerland | milk | cow |
| 366 | BS | USA | milk | cow |
| 367 | CF | Germany | milk | cow |
| 369 | BS | United Kingdom | milk | cow |
| 383 | EX | Germany | milk | cow |
|  | BS | Germany | cheese (crescenza, fontina, mozzarella, parmigiano, ricotta)* | cow |
|  |  |  | milk | cow |
| 384 | EX | Germany | milk | cow |
| 385 | CF | Greece | milk | sheep* |
|  |  |  | milk | goat* |
| 386 | EX | Italy | milk | goat* |
| 387 | CF | United Kingdom | milk | cow |
|  | BS | United Kingdom | milk | cow |
| 388 | CF | United Kingdom | milk | cow |
| 392 | BS | Denmark | milk | cow |
| 393 | CF | Latvia | milk | cow |
| 394 | CF | Norway | milk | cow |
| 395 | CF | Norway | milk | cow |
| 396 | CF | Canada | milk | cow |
| ID, Paper unique identification number (see Table S1 for references); ST, Study type (CF – Comparison of Farms, BS – Basket Study, EX – Controlled Experiment). *Data for products other than milk and animals other than cow was described in the main paper and summarised in Figure S35, Table S14 and S15. | | | | |

| **Table S2 cont.** Study type, location, product and animal species information for studies included in the systematic review and the meta-analysis. | | | | |
| --- | --- | --- | --- | --- |
| **ID** | **ST** | **Location** | **Product** | **Animal species** |
| 398 | CF | Denmark | milk | cow |
| 399 | CF | Denmark | milk | cow |
| 401 | CF | United Kingdom | milk | cow |
| 402 | BS | Germany | milk | cow |
| 403 | EX | Germany | milk | cow |
|  |  |  | cheese* | cow |
| 404 | BS | Germany | milk | cow |
| 405 | CF | Italy | milk | cow |
| 406 | CF | Italy | cheese (latteria)* | cow |
|  |  |  | milk | cow |
| 408 | CF | The Netherlands | milk | cow |
| 409 | CF | Germany | milk | cow |
| 410 | BS | Germany | milk | cow |
| 412 | EX | Sweden | milk | cow |
| 413 | CF | Canada | milk | cow |
| 414 | CF | United Kingdom | milk | cow |
| 418 | CF | Lithuania | milk | cow |
| 419 | CF | Italy | milk | cow |
|  |  | Sweden | milk | cow |
| 420 | CF | Sweden | milk | cow |
| 455 | BS | Brazil | milk | cow |
|  |  |  | fermented milk* | cow |
|  |  |  | yoghurt* | cow |
| 456 | BS | Spain | milk | sheep* |
| 457 | CF | Republic of Serbia | milk | cow |
| 458 | EX | Austria | milk | cow |
| 461 | BS | Brazil | milk | cow |
| 464 | CF | Poland | milk | cow |
| 465 | EX | Sweden | milk | cow |
| 467 | CF | Sweden | milk | cow |
| 472 | EX | Poland | milk | cow |
| ID, Paper unique identification number (see Table S1 for references); ST, Study type (CF – Comparison of Farms, BS – Basket Study, EX – Controlled Experiment). *Data for products other than milk and animals other than cow was described in the main paper and summarised in Figure S35, Table S14 and S15. | | | | |

| **Table S2 cont.** Study type, location, product and animal species information for studies included in the systematic review and the meta-analysis. | | | | |
| --- | --- | --- | --- | --- |
| **ID** | **ST** | **Location** | **Product** | **Animal species** |
| 474 | CF | Italy | milk | buffalo* |
| 481 | CF | Italy | milk | cow |
| 487 | EX | Spain | milk | goat* |
| 547 | CF | Romania | milk | sheep* |
| 551 | CF | Sweden | milk | cow |
| 552 | CF | United Kingdom | milk | cow |
| 553 | CF | USA | milk | cow |
| 554 | CF | USA | milk | cow |
| 555 | CF | USA | milk | cow |
| 556 | BS | Italy | cheese (fontina)* | cow |
|  |  |  | milk | cow |
| 576 | CF | Sweden | milk | cow |
| 577 | CF | Republic of Serbia | milk | cow |
| 588 | CF | Poland | milk | cow |
| 589 | BS | Belgium | milk | cow |
| 590 | CF | Estonia | milk | cow |
| 591 | BS | Brazil | milk | cow |
|  |  |  | fermented milk* | cow |
|  |  |  | yoghurt* | cow |
| 592 | BS/CF | Italy | milk | cow |
| 593 | BS | United Kingdom | milk | cow |
| 594 | BS | Spain | milk | cow |
| 595 | BS | Brazil | milk | cow |
|  |  |  | yoghurt* | cow |
| 596 | CF | Norway | milk | cow |
| 597 | CF | Romania | milk | cow |
| 598 | BS/CF | USA | milk | cow |
| 599 | CF | Sweden | milk | cow |
| 600 | CF | Poland | milk | cow |
| 601 | CF | Germany | milk | cow |
| ID, Paper unique identification number (see Table S1 for references); ST, Study type (CF – Comparison of Farms, BS – Basket Study, EX – Controlled Experiment). *Data for products other than milk and animals other than cow was described in the main paper and summarised in Figure S35, Table S14 and S15. | | | | |

| **Table S2 cont.** Study type, location, product and animal species information for studies included in the systematic review and the meta-analysis. | | | | |
| --- | --- | --- | --- | --- |
| **ID** | **ST** | **Location** | **Product** | **Animal species** |
| 602 | BS | Germany | milk | cow |
| 616 | EX | Italy | milk | sheep* |
| 617 | BS | Italy | yoghurt | goat* |
| 618 | CF | Poland | milk | cow |
| 626 | BS | USA | milk | cow |
| 627 | CF | The Netherlands | milk | cow |
| 628 | BS | Spain, United Kingdom | milk | cow |
|  |  |  | milk | goat* |
|  | CF | Spain | milk | goat* |
| 629 | CF | USA | milk | cow |
| 630 | CF | USA | milk | cow |
| 631 | CF | Brazil | milk | goat* |
| 663 | EX | Norway | milk | cow |
| 664 | CF | Norway | milk | cow |
| 665 | BS | Spain | cheese* | cow |
| 666 | BS | Italy | butter* | cow |
| 667 | CF | Italy | milk | sheep* |
| 668 | BS | Switzerland | cheese (emmentaler)* | cow |
| 669 | CF | Denmark | desalted milk* | cow |
|  |  |  | whey* | cow |
| 670 | CF | Greece | milk | goat and sheep* |
| 671 | CF | Finland | milk | cow |
| 672 | CF | USA | milk | cow |
| 673 | BS | USA | milk | cow |
| 674 | BS | Canada | cheese (cheddar)* | cow |
|  |  |  | cheese (feta)* | cow |
|  |  |  | cheese (gouda)* | cow |
|  |  |  | cheese (feta) | not specified* |
|  |  |  | cheese (mozzarella) | not specified* |
| 675 | CF | Spain | milk | cow |
| ID, Paper unique identification number (see Table S1 for references); ST, Study type (CF – Comparison of Farms, BS – Basket Study, EX – Controlled Experiment). *Data for products other than milk and animals other than cow was described in the main paper and summarised in Figure S35, Table S14 and S15. | | | | |

| **Table S2 cont.** Study type, location, product and animal species information for studies included in the systematic review and the meta-analysis. | | | | |
| --- | --- | --- | --- | --- |
| **ID** | **ST** | **Location** | **Product** | **Animal species** |
| 676 | BS | Greece | milk | cow |
| 678 | BS | Germany | butter* | cow |
| 679 | BS | Poland | milk | cow |
| 680 | BS | Turkey | milk | cow |
| 681 | CF | Czech Republic | milk | cow |
| 682 | CF | Slovenia | milk | sheep* |
| 683 | CF | Slovenia | milk | goat* |
| 684 | CF | Brazil | milk | cow |
| 686 | CF | Italy | milk | buffalo* |
| 687 | CF | Latvia | milk | goat* |
| 688 | CF | Slovenia | milk | sheep* |
| 689 | CF | Czech Republic | milk | cow |
| 690 | CF | Mexico | milk | cow |
| 692 | CF | Germany | milk | cow |
| 693 | CF | Latvia | milk | cow |
| 694 | CF | Latvia | milk | cow |
| 695 | BS | Spain | milk | cow |
| 696 | BS | Germany | milk | cow |
| 697 | BS | Germany | cheese* | cow |
|  | EX | Germany | milk | cow |
| 698 | CF | Romania | milk | cow |
|  |  |  | milk | sheep* |
| 699 | EX | Spain | milk | cow |
| 700 | BS | Brazil | milk | cow |
| 702 | EX | United Kingdom | milk | cow |
| 703 | BS | Denmark | milk | cow |
| 704 | EX | Sweden | milk | cow |
| 705 | BS | Brazil | milk | cow |
|  |  |  | fermented milk* | cow |
| ID, Paper unique identification number (see Table S1 for references); ST, Study type (CF – Comparison of Farms, BS – Basket Study, EX – Controlled Experiment). *Data for products other than milk and animals other than cow was described in the main paper and summarised in Figure S35, Table S14 and S15. | | | | |

| **Table S2 cont.** Study type, location, product and animal species information for studies included in the systematic review and the meta-analysis. | | | | |
| --- | --- | --- | --- | --- |
| **ID** | **ST** | **Location** | **Product** | **Animal species** |
| 706 | BS | Spain | milk | cow |
| 707 | BS | Spain | milk | cow |
| 708 | EX | New Zealand | milk | cow |
| 709 | BS | Romania | cheese* | sheep* |
| 710 | BS | Germany | butter* | cow |
|  |  |  | cream* | cow |
| 712 | BS | Poland | milk | cow |
| 713 | EX | Greece | milk | sheep* |
| 714 | CF | Slovakia | milk | cow |
| 715 | CF | Denmark | milk | cow |
| 716 | CF | The Netherlands | milk | cow |
| 717 | CF | Sweden | milk | cow |
| 720 | CF | Germany | milk | cow |
| 721 | CF | Belgium | milk | cow |
| 722 | CF | Germany | milk | cow |
| 723 | CF | Canada | milk | cow |
| 724 | EX | Norway | milk | cow |
| 725 | BS | Denmark | milk | cow |
| 726 | CF | USA | milk | cow |
| 727 | CF | USA | milk | cow |
| 728 | CF | USA | milk | cow |
| 729 | CF | Sweden | milk | cow |
| 730 | CF | Norway | milk | cow |
| 731 | CF | Denmark | milk | cow |
| 732 | CF | Brazil | milk | cow |
| 733 | CF | Denmark | milk | cow |
| 734 | CF | Denmark | milk | cow |
| 735 | CF | Denmark | milk | cow |
| 736 | CF | USA | milk | cow |
| 737 | CF | USA | milk | cow |
| ID, Paper unique identification number (see Table S1 for references); ST, Study type (CF – Comparison of Farms, BS – Basket Study, EX – Controlled Experiment). *Data for products other than milk and animals other than cow was described in the main paper and summarised in Figure S35, Table S14 and S15. | | | | |

| **Table S2 cont.** Study type, location, product and animal species information for studies included in the systematic review and the meta-analysis. | | | | |
| --- | --- | --- | --- | --- |
| **ID** | **ST** | **Location** | **Product** | **Animal species** |
| 738 | EX | New Zealand | milk | cow |
| 739 | CF | Denmark | milk | cow |
| 740 | CF | Sweden | milk | cow |
| 741 | CF | Canada | milk | cow |
| 742 | CF | USA | milk | cow |
| 743 | CF | Switzerland | milk | cow |
| 744 | CF | Canada | milk | cow |
| 745 | EX | New Zealand | milk | cow |
| 746 | CF | Canada | milk | cow |
| 747 | EX | New Zealand | milk | cow |
| 748 | CF | Sweden | milk | cow |
| 749 | CF | Austria | milk | cow |
| 750 | CF | France | milk | cow |
| 751 | CF | Norway | milk | cow |
| 752 | EX | New Zealand | milk | cow |
| 753 | CF | USA | milk | cow |
| 754 | CF | Sweden | milk | cow |
| 755 | CF | The Netherlands | milk | cow |
| ID, Paper unique identification number (see Table S1 for references); ST, Study type (CF – Comparison of Farms, BS – Basket Study, EX – Controlled Experiment). *Data for products other than milk and animals other than cow was described in the main paper and summarised in Figure S35, Table S14 and S15. | | | | |

| **Table S3.** Production systems information for studies with more than two systems included in the meta-analysis. | | | | |  |
| --- | --- | --- | --- | --- | --- |
| **ID** | **Location** | **SI** | **Production system as described by authors** | **Additional comparisons used in the sensitivity analyses 3 to 6*** | |
| 157 | United Kingdom | 1 | organic (low-input)† | 1 and 3 | |
|  |  | 2 | conventional (high input, no more than 50% concentrate)† |  | |
|  |  | 3 | non-organic low-input (New Zealand-type) |  | |
| 158 | United Kingdom | 1 | organic (low-input)† | 1 and 3 | |
|  |  | 2 | conventional (high input, no more than 50% concentrate)† |  | |
|  |  | 3 | non-organic low-input (New Zealand-type) |  | |
| 176 | Germany | 1 | organic† | 1 and 3 | |
|  |  | 2 | conventional (pasture)† |  | |
|  |  | 3 | conventional (indoor) |  | |
| 178 | Germany | 1 | organic† |  | |
|  |  | 2 | conventional† |  | |
|  |  | 3 | conventional (Swiss-type 1)‡ |  | |
|  |  | 4 | conventional (Swiss-type 2)‡ |  | |
| 192 | Denmark | 1 | organic† |  | |
|  |  | 2 | conventional† |  | |
|  |  | 3 | conventional (extensive, Danish-type)‡ |  | |
| 196 | USA | 1 | organic (labelled)† | 1 and 3 | |
|  |  | 2 | conventional† |  | |
|  |  | 3 | recombinant bovine somatotropin free (rbST-free) milk |  | |
| ID, Paper unique identification number (see Table S1 for references); SI, system identifier. *Numbers refer to the SI within the same study; †Used as a standard system in the standard meta-analysis; ‡Results from these treatments were removed from the meta-analysis; §Results from these treatments were averaged and used as a standard system in the meta-analysis. | | | | |  |

| **Table S3 cont.** Production systems information for studies with more than two systems included in the meta-analysis. | | | | |  |
| --- | --- | --- | --- | --- | --- |
| **ID** | **Location** | **SI** | **Production system as described by authors** | **Additional comparisons used in the sensitivity analyses 3 to 6*** | |
| 366 | USA | 1 | organic (labelled)† | 1 and 3 | |
|  |  | 2 | conventional† |  | |
|  |  | 3 | recombinant bovine somatotropin free (rbST-free) milk |  | |
| 414 | United Kingdom | 1 | organic (grazing-based, outdoor Apr-Oct)† | 1 and 3 | |
|  |  | 2 | conventional (grazing-based, standard milking)† | 1 and 4 | |
|  |  | 3 | conventional (grazing-based, robotic milking) |  | |
|  |  | 4 | conventional (indoor, high concentrate) |  | |
| 464 | Poland | 1 | organic (certified)† | 1 and 3 | |
|  |  | 2 | conventional (modern intensive)† |  | |
|  |  | 3 | conventional (extensive) |  | |
| 601 | Germany | 1 | organic (biodynamic, high-input)† | 1 and 4 | |
|  |  | 2 | conventional (high-input)† | 2 and 3 | |
|  |  | 3 | organic (biodynamic, low-input) | 3 and 4 | |
|  |  | 4 | conventional (low-input) |  | |
| 627 | The Netherlands | 1 | organic (biodynamic, continuous grazing)† | 1 and 3 | |
|  |  | 2 | conventional (daytime grazing)† | 1 and 4 | |
|  |  | 3 | conventional (no fresh grass) | 1 and 5 | |
|  |  | 4 | conventional (indoor with cut fresh grass) |  | |
|  |  | 5 | conventional (continuous grazing) |  | |
| ID, Paper unique identification number (see Table S1 for references); SI, system identifier. *Numbers refer to the SI within the same study; †Used as a standard system in the standard meta-analysis; ‡Results from these treatments were removed from the meta-analysis; §Results from these treatments were averaged and used as a standard system in the meta-analysis. | | | | |  |

| **Table S3 cont.** Production systems information for studies with more than two systems included in the meta-analysis. | | | | |  |
| --- | --- | --- | --- | --- | --- |
| **ID** | **Location** | **SI** | **Production system as described by authors** | **Additional comparisons used in the sensitivity analyses 3 to 6*** | |
| 629 | USA | 1 | organic† | 1 and 3 | |
|  |  | 2 | conventional (no grazing)† |  | |
|  |  | 3 | conventional (grazing) |  | |
| 630 | USA | 1 | organic† | 1 and 3 | |
|  |  | 2 | conventional (no grazing)† |  | |
|  |  | 3 | conventional (grazing) |  | |
| 663 | Norway | 1 | organic (short-term grassland with timothy and red clover)† | 1 and 4 | |
|  |  | 2 | conventional (ley with timothy)† | 2 and 3 | |
|  |  | 3 | organic (long-term grassland with a high proportion of unsown species) | 3 and 4 | |
|  |  | 4 | conventional (ley with perennial ryegrass) |  | |
| 664 | Norway | 1 | organic (short-term grassland)† | 1 and 4 | |
|  |  | 2 | conventional (short-term grassland)† | 2 and 3 | |
|  |  | 3 | organic (long-term grassland) | 3 and 4 | |
|  |  | 4 | conventional (long-term grassland) |  | |
| 692 | Germany | 1 | organic (high-input)† | 1 and 4 | |
|  |  | 2 | conventional (high-input)† | 2 and 3 | |
|  |  | 3 | organic (low-input) | 3 and 4 | |
|  |  | 4 | conventional (low-input) |  | |
| ID, Paper unique identification number (see Table S1 for references); SI, system identifier. *Numbers refer to the SI within the same study; †Used as a standard system in the standard meta-analysis; ‡Results from these treatments were removed from the meta-analysis; §Results from these treatments were averaged and used as a standard system in the meta-analysis. | | | | |  |

| **Table S3 cont.** Production systems information for studies with more than two systems included in the meta-analysis. | | | | |  |
| --- | --- | --- | --- | --- | --- |
| **ID** | **Location** | **SI** | **Production system as described by authors** | **Additional comparisons used in the sensitivity analyses 3 to 6*** | |
| 723 | Canada | 1 | organic (certified)† |  | |
|  |  | 2 | conventional (global extensive, production less than 4225 L per ha fodder)§ |  | |
|  |  | 3 | conventional (global intensive, production more than 8336 L per ha fodder)§ |  | |
|  |  | 4 | conventional (extensive dairy, production less than 5719 L per cow)§ |  | |
|  |  | 5 | conventional (intensive dairy, production more than 7338 L per cow)§ |  | |
|  |  | 6 | conventional (low-input, production less than 257 kg per cow protein concentrate)§ |  | |
|  |  | 7 | conventional (high-input, production more than 490 kg per cow protein concentrate)§ |  | |
| 742 | USA | 1 | organic† | 1 and 3 | |
|  |  | 2 | conventional (no grazing)† |  | |
|  |  | 3 | conventional (grazing) |  | |
| 753 | USA | 1 | organic† | 1 and 3 | |
|  |  | 2 | conventional (no grazing)† |  | |
|  |  | 3 | conventional (grazing) |  | |
| ID, Paper unique identification number (see Table S1 for references); SI, system identifier. *Numbers refer to the SI within the same study; †Used as a standard system in the standard meta-analysis; ‡Results from these treatments were removed from the meta-analysis; §Results from these treatments were averaged and used as a standard system in the meta-analysis. | | | | |  |

| **Table S4.** Information extracted from papers and included in the database used for meta-analysis. | |
| --- | --- |
| **Information  about the paper** | Paper ID, authors, publication year, title, journal/publisher, type of paper (journal article, conference proceedings, conference paper, report, book chapter, thesis), corresponding author, language of publication, information about peer-review, source of paper (electronic databases, contact with authors, reference list of reviews and original publications). |
| **Study characteristics** | Study type (CF, comparison of farms; BS, basket study; EX, controlled experiment), product, species, breed, production system description, experimental year(s), location of the study by country*. |
| **Data** | Name of the compositional parameter, number of replicates, mean, standard error (SE), standard deviation (SD), measurement unit, data type (numeric, graphical). |
| *Country codes according ISO 3166-2 (see [*http://www.iso.org/iso/home/standards/country_codes.htm*](http://www.iso.org/iso/home/standards/country_codes.htm)) | |

| **Table S5.** Summary of inclusion criteria used in the standard and the sensitivity analyses carried out. Results of the sensitivity analyses 2-8 are shown in the Appendix on the Newcastle University website ([*http://research.ncl.ac.uk/nefg/QOF*](http://research.ncl.ac.uk/nefg/QOF)) | | | | | | | | | | |
| --- | --- | --- | --- | --- | --- | --- | --- | --- | --- | --- |
| **Analysis** | **Data available** | |  | **Experimental years** | |  | **Production systems compared** | |  | **20% of studies with the least precise treatment effects excluded** |
|  | Only papers  reporting N, mean, SD/SE | All papers  reporting means |  | One data point from one paper* | Individual year as separate data points† |  | Standard organic  with standard conventional‡ | Each organic  with each conventional |  |  |
| Standard§ |  |  |  |  |  |  |  |  |  |  |
| WM | + |  |  | + |  |  | + |  |  |  |
| Sensitivity\|\| |  |  |  |  |  |  |  |  |  |  |
| 1 (UM)§ |  | + |  | + |  |  | + |  |  |  |
| 2 (WM) | + |  |  |  | + |  | + |  |  |  |
| 3 (UM) |  | + |  |  | + |  | + |  |  |  |
| 4 (WM) | + |  |  | + |  |  |  | + |  |  |
| 5 (UM) |  | + |  | + |  |  |  | + |  |  |
| 6 (WM) | + |  |  |  | + |  |  | + |  |  |
| 7 (UM) |  | + |  |  | + |  |  | + |  |  |
| 8 (WM) | + |  |  | + |  |  | + |  |  | + |
| *If data from more than one experimental years were presented separately in the paper, average was calculated and included in the meta-analysis; †If data from more than one experimental years were presented separately in the paper, they were analysed separately, as individual data points; ‡A pragmatic choice was made to compare standard organic with a standard conventional comparator; §Results of the standard meta-analysis and sensitivity analysis 1 are presented in the main paper; \|\|Sensitivity analysis was conducted to explore the robustness of the arbitrary decisions and to illustrate all effects (see Supplementary Table S3 for details and Appendix Table A1 and A2 for results). WM, weighted meta-analysis; UM, unweighted meta-analysis. | | | | | | | | | | |

| **Table S6.** List of composition parameters included in the meta-analysis.* | |
| --- | --- |
| **Category** | **Parameters** |
| **Major components** | Ash, Casein, Fat, Lactose, Protein, Protein (whey), Solids, Solids (no-fat),  α-lactalbumin, β-lactoglobulin |
| **Fatty acids** | 18:1, 18:2, 18:3, 18:4, 10:0 (capric acid), 10:1 (4-cis-decenoic acid), 12:0 (lauric acid),  12:0+14:0+16:0†, 12:1 (lauroleic acid), 13:0 (tridecylic acid), 14:0 (myristic acid),  14:1 (myristoleic acid), 15:0 (pentadecanoic acid), 16:0 (palmitic acid), 16:1 (palmitoleic acid), 17:0 (heptadecanoic acid), 17:1 (heptadecenoic acid), 18:0 (stearic acid), 20:0 (arachidic acid), 22:0 (behenic acid), 24:0 (lignoceric acid), 4:0 (butyric acid), 6:0 (caproic acid), 8:0 (caprylic acid), AA (cis-5,8,11,14-20:4), ALA (cis-9,12,15-18:3), cis-11,14-20:2, cis-11-18:1 (cis-vaccenic acid), cis-11-20:1 (eicosenoic acid), cis-12-18:1, cis-13-18:1, cis-9-20:1, CLA9 (cis-9-trans-11-18:2), CLA (total), CLA10 (trans-10-cis-12-18:2), CLA (trans-11,13-18:2), CLA (trans-12,14-18:2), CLA (trans-7,9-18:2), CLA (trans-9,11-18:2), DGLA (cis-8-11-14-C20:3), DHA (cis-4,7,10,13,16,19-22:6), DPA (cis-7,10,13,16,19-22:5), EPA (cis-5,8,11,14,17-20:5), ETE (cis-11,14,17-20:3), Free fatty acids, GLA (cis-6,9,12-18:3), LA (cis-9,12-18:2), LA/ALA ratio†, Long chain FA, Medium chain FA, MUFA, n-3 FA, n-3/n-6 ratio, n-6 FA, n-6/n-3 ratio, OA (cis-9-18:1), Phytanic acid diastereomers ratio (SRR/RRR), PUFA, SFA, Short chain FA, trans-12-18:1, trans-18:1, trans-6-8-18:1, trans-9,12-18:2, trans-9-18:1 (elaidic acid), USFA, VA (trans-11-18:1), VLC n-3 PUFA (EPA+DPA+DHA)† |
| **N components** | Urea |
| **Vitamins and antioxidants** | 2R (synthetic) isomers of α-tocopherol, 3R (natural) isomers of α-tocopherol, Carotenoids, Lutein, Vitamin A, Vitamin C, Vitamin D, Vitamin E activity, Zeaxanthin, α-tocopherol, β-carotene |
| **Minerals and undesirable metals** | Cadmium (Cd), Calcium (Ca), Cobalt (Co), Copper (Cu), Iodine (I), Iron (Fe),  Lead (Pb), Magnesium (Mg), Manganese (Mn), Molybdenum (Mo), Phosphorus (P), Potassium (K), Selenium (Se), Sodium (Na), Zinc (Zn) |
| **Pesticides, mycotoxins  and other contaminants** | Aflatoxin M1, Dieldrin, Hexachlorobenzene (HCB), α-esachlorciclohexane (α-HCH),  γ-esachlorciclohexane (γ-HCH) |
| **Other** | Atherogenicity Index, Bacteria count, Dry mass, Lactoferrin, Lysozyme, Milk yield, pH, SCC, Thrombogenicity index, Titratable acidity |
| *Compounds for which number of comparisons organic vs. conventional was ≥ 3, †Calculated based on published fatty acids composition data. | |

| **Table S7.** List of composition parameters excluded from the meta-analysis.* | |
| --- | --- |
| **Category** | **Parameters** |
| **Major components** | Butterfat, Protein (crude), Protein (true), Solids (other), Water, α-casein, β-casein,  β-lactoglobulin A (βLgA), β-lactoglobulin B (βLgB), κ-casein |
| **Fatty acids** | 9:0, 11:0, 15:1, 19:0, 20:1, 20:3, 21:0, 22:1, 22:2, 18:2 ttNMID, 23:0 (cerotic acid), 3R,7R,11R,15-phytanic acid (RRR), 3R,7R,11R,15-phytanic acid (RRR), 3S,7R,11R,15-phytanic acid (SRR) (% total phytanic acid isomers), 5:0 (valeric acid), 6:0+15:1+17:0+cis-9,12,15-18:3, 7:0 (enanthic acid), ALA+CLA, ALA+GLA, Anteiso-12:0, Anteiso-13:0, Anteiso-14:0, Anteiso-15:0, Anteiso-16:0, Anteiso-17:0, Branched 15:0, Branched 17:0, Branched chain FA (total), Cis fatty acids, cis-11-22:1, cis-12:1+13:0, cis-13,16-22:2, cis-13-22:1, cis-14:1, cis-14-18:1+trans-16-18:1, cis-15-18:1, cis-15-24:1, cis-4,7,10,13,16-22:5 (total), cis-5-20:1, cis-7-10-18:1, cis-7-14:1, cis-7-16:1, cis-9,15-18:2, cis-9-16:1, cis-9-17:1 (margaroleic acid), cis-9-20:1, cis-9-trans-12-18:2, cis-9-trans12-18:2+cis-cis-MID+trans-8-cis-13-18:2, cis-9-trans-13-18:2+trans-8-cis-12-18:2, CLA (cis/trans-12,14-18:2), CLA (cis-10-trans-12/trans-10-cis-12-18:2), CLA (cis-11-trans-13/trans-11-cis-13-18:2), CLA (cis-11-trans-13-18:2), CLA (cis-18:2), CLA (cis-7-trans-9/trans-7-cis-9-18:2), CLA (cis-9-trans-11/trans-9-cis-11-18:2), CLA (cis-9-trans-11-18:2) + (trans-8-cis-10-18:2) + (trans-7-cis-9-18:2), CLA (cis-9-trans-11-18:2) + (trans-9-cis-11-18:2), CLA (cis-trans/trans-cis-18:2), CLA (trans-8,10-18:2), CLA (trans-10,12-18:2), CLA (trans-11-cis-13-18:2), CLA (trans-11-cis-13-18:2) / (trans-7-cis-9-18:2) ratio, CLA (trans-11-cis-13-18:2) + (cis-9,11-18:2), CLA (trans-6,8-18:2), CLA (trans-7-cis-9-18:2), CLA (trans-8-cis-10-18:2), CLA (trans-trans-18:2), CLA/LA ratio, DTA (cis-7,10,13,16-22:4), Fatty acids (total), Functional Fatty Acid, Furan fatty acid DiMeF(11,5), Furan fatty acid DiMeF(9,5), Furan fatty acid MeF(11,5), Furan fatty acid MeF(7,5), Furan fatty acid MeF(9,5), Furan fatty acids, Iso-12:0, Iso-13:0, Iso-14:0, Iso-15:0, Iso-16:0, Iso-17:0, LA/ALA ratio, Long chain n-3 FA, n-6/(n-3+CLA) ratio, Pristanic acid, Serum albumin, SFA/USFA ratio, Trans FA, Trans fatty acids without CLA, trans-10-18:1, trans-10-18:1+trans-11-18:1, trans-11-cis-15-18:2+trans-9-cis-12-18:2, trans-13-14-18:1+cis-6-8-18:1, trans-13-18:1, trans-14:1, trans-15-18:1, trans-16:1, trans-16-18:1, trans-17:1, trans-18:2 (trans-octadecadienoic acid), trans-20:1, trans-4-18:1, trans-5-18:1, trans-7-16:1, trans-9,12-18:2+cis-16-18:1, trans-9-14:1, trans-9-cis-12-18:2, Triglycerides |
| **N components** | Alanine, Ammonia, Arginine, Aspartic acid, Cysteine, Glutamine, Glycine, Histidine, Isoleucine, Leucine, Lysine, Methionine, Nitrate, Nitrite, Nitrogen, Nitrogen (non-protein), Nitrogen (non-protein), Phenylalanine, Proline, Protein N (% total N), Serine, TCA-soluble nitrogen, Threonine, Tyrosine, Valine, Val-Pro-Pro (VPP) peptide, VPP + IPP peptides |
| **Vitamins and antioxidants** | Antioxidant Capacity (Trolox equivalent), Biochanin (isoflavone), Butylated hydroxytoluene (BHT) (synthetic antioxidant), Butylated hydroxytoluene (BHT) (synthetic antioxidant), Canthaxanthin, Citric acid, Coumestrol (coumestans), Daidzein, Enterodiol, Enterolactone, Formononetin, Genistein, Hippuric acid, Matairesinol, Prunetin, Riboflavin, Secoisolariciresinol, Total antioxidant status (TAS), Vitamin B1, Vitamin K, α-carotene, β-carotene isomers, β-cryptoxanthin |
| **Minerals and undesirable metals** | Aluminium (Al), Arsenic (As), Barium (Ba), Bismuth (Bi), Boron (B), Chromium (Cr), Europium (Eu), Germanium (Ge), Lithium (Li), Mercury (Hg), Minerals (total), Neodymium (Nd), Nickel (Ni), Rhenium (Re), Rhodium (Rh), Silicon (Si), Strontium (Sr), Sulfur (S), Tin (Sn), Vanadium (V) |
| *Compounds for which number of comparisons organic vs. conventional was < 3. | |

| **Table S7 cont.** List of composition parameters excluded from the meta-analysis.* | |
| --- | --- |
| **Category** | **Parameters** |
| **Pesticides, mycotoxins  and other contaminants** | 1,2,3,4,6,7,8-HpCDD, 1,2,3,4,6,7,8-HpCDF, 1,2,3,4,7,8,9-HpCDF, 1,2,3,4,7,8-HxCDD, 1,2,3,4,7,8-HxCDF, 1,2,3,6,7,8-HxCDD, 1,2,3,6,7,8-HxCDF, 1,2,3,7,8,9-HxCDD, 1,2,3,7,8,9-HxCDF, 1,2,3,7,8-PeCDD, 1,2,3,7,8-PeCDF, 2,3,4,6,7,8-HxCDF, 2,3,4,7,8-PeCDF, 2,3,7,8-TCDD, 2,3,7,8-TCDF, Aflatoxin, Aldrin, Chlordanes, Cis-clordane, Cis-clordane, Cyclodienes, DDE, DDT, Dioxin-like-polychlorinated biphenyls, Dioxins, Dioxins + furans, Endosulfans, Endrin, Eptachlor, Esachlorciclohexane (HCH), Furans, H-epoxide, Heptachlor, Marker-polychlorinated biphenyls, Methoxychlor, Mirex, Non-ortho polychlorinated biphenyls (Non-ortho-PCB), o,p'DDD, o,p'DDE, o,p'DDT, Ochratoxin A, Octaclorostyrene, Organochlorine pesticides, Ortho polychlorinated biphenyls (Ortho-PCB), Ossiclordane, p,p'DDD, p,p'DDE, p,p'DDT, Pesticide residues, Polybrominated diphenyl ethers (PBDE), Polychlorinated biphenyl 101 (PCB 101), Polychlorinated biphenyl 105 (PCB 105), Polychlorinated biphenyl 114 (PCB 114), Polychlorinated biphenyl 118 (PCB 118), Polychlorinated biphenyl 123 (PCB 123), Polychlorinated biphenyl 126 (PCB 126), Polychlorinated biphenyl 138 (PCB 138), Polychlorinated biphenyl 153 (PCB 153), Polychlorinated biphenyl 156 (PCB 156), Polychlorinated biphenyl 157 (PCB 157), Polychlorinated biphenyl 167 (PCB 167), Polychlorinated biphenyl 169 (PCB 169), Polychlorinated biphenyl 180 (PCB 180), Polychlorinated biphenyl 189 (PCB 189), Polychlorinated biphenyl 28 (PCB 28), Polychlorinated biphenyl 52 (PCB 52), Polychlorinated biphenyl 77 (PCB 77), Polychlorinated biphenyl 81 (PCB 81), Polychlorinated biphenyls (PCB), Polychlorinated Biphenyls toxicity equivalents (TEQ-PCB), Quintozene, Trans-clordane, Trans-clordene, Trans-nonachlor,  WHO-Toxic Equivalents (TEQ), α+β-esachlorciclohexane (α+β-HCH), α-endosulfan,  β-endosulfan, β-esachlorciclohexane (β-HCH), Δ-esachlorciclohexane (Δ-HCH) |
| **Volatile compounds** | Acetone, Ether extract |
| **Other** | Acidity, Alcohol stability, Bovine serum albumin (BSA), Bovine somatotropin, Coliforms bacteria, Equol, Estradiol, Estrone (E1), Hypocholesterolemic/ hypercholesterolemic FA ratio, Ile-Pro-Pro (IPP) peptide, Immunoglobulin A (IgA), Immunoglobulin G (IgG), Immunoglobulin M (IgM), Insulin-like growth factor-1 (IGF-1), Lactate, Lactic acid, Lactoperoxidase, Lymphocyte stimulation index (in vitro), Plate loop count, Progesterone, Sodium chloride (NaCl), Spontaneous lymphocyte activity (in vitro), Stable carbon isotope 13C, Δ-9 desaturase 14:1/14:0 activity index, Δ-9 desaturase 16:1/16:0 activity index, Δ-9 desaturase 18:1/18:0 activity index, Δ-9 desaturase CLA (cis-9-trans-11-18:2) / VA activity index |
| *Compounds for which number of comparisons organic vs. conventional was < 3. | |

# 2. ADDITIONAL RESULTS

| **Table S8.** Basic information/statistics on the publications/data used for meta-analyses of composition parameters included in Fig. 2 and 3 in the main paper. | | | | | | | | | | |
| --- | --- | --- | --- | --- | --- | --- | --- | --- | --- | --- |
|  |  |  |  |  | **Number of comparisons reporting that concentrations were** | | | | | |
|  |  |  | **Total sample size*** | | **Numerically higher in** | | **Identical** | **Significantly higher in** | | **Not significantly different**§ |
| **Parameter** | **Studies** | ***n*** | **ORG** | **ORG** | **ORG** | **CONV** |  | **ORG**† | **CONV**‡ |  |
| Milk yield | 81 | 81 | 4237 | 23587 | 8 | 73 | 0 | 2 | 29 | 9 |
| SFA | 32 | 33 | 390 | 384 | 16 | 17 | 0 | 3 | 3 | 16 |
| 12:0 (lauric acid) | 17 | 17 | 260 | 250 | 8 | 9 | 0 | 7 | 3 | 4 |
| 14:0 (myristic acid) | 18 | 18 | 265 | 255 | 11 | 7 | 0 | 7 | 2 | 6 |
| 16:0 (palmitic acid) | 20 | 20 | 279 | 269 | 7 | 13 | 0 | 4 | 6 | 7 |
| MUFA | 30 | 31 | 365 | 360 | 13 | 18 | 0 | 2 | 9 | 10 |
| OA (cis-9-18:1) | 16 | 16 | 252 | 242 | 8 | 8 | 0 | 3 | 4 | 6 |
| VA (trans-11-18:1) | 18 | 18 | 219 | 266 | 18 | 0 | 0 | 9 | 0 | 4 |
| PUFA | 29 | 30 | 595 | 581 | 25 | 4 | 1 | 11 | 1 | 9 |
| CLA (total) | 18 | 19 | 159 | 141 | 17 | 2 | 0 | 4 | 0 | 6 |
| CLA9 (cis-9-trans-11-18:2) | 20 | 20 | 557 | 590 | 19 | 1 | 0 | 7 | 0 | 5 |
| CLA10 (trans-10-cis-12-18:2) | 7 | 7 | 109 | 120 | 4 | 0 | 2 | 1 | 0 | 4 |
| n-3 FA | 19 | 20 | 289 | 281 | 20 | 0 | 0 | 13 | 0 | 1 |
| ALA (cis-9,12,15-18:3) | 33 | 34 | 678 | 698 | 34 | 0 | 0 | 17 | 0 | 3 |
| EPA (cis-5,8,11,14,17-20:5) | 13 | 14 | 287 | 281 | 13 | 0 | 1 | 9 | 0 | 2 |
| DPA (cis-7,10,13,16,19-22:5) | 8 | 8 | 198 | 192 | 8 | 0 | 0 | 6 | 0 | 2 |
| DHA (cis-4,7,10,13,16,19-22:6) | 6 | 6 | 187 | 181 | 4 | 0 | 2 | 2 | 0 | 3 |
| VLC n-3 PUFA\|\| | 5 | 5 | 175 | 169 | 5 | 0 | 0 | 0 | 0 | 0 |
| *n*, numbers of data points (comparisons) included in the meta-analysis; ORG, organic samples; CONV, conventional samples; SFA, saturated fatty acids; MUFA, monounsaturated fatty acids; OA, oleic acid; VA, vaccenic acid; PUFA, polyunsaturated fatty acids; FA, fatty acids; CLA, conjugated linoleic acid; ALA, α-linolenic acid; EPA, eicosapentaenoic acid; DPA, docosapentaenoic acid; DHA, docosahexaenoic acid; VLC n-3 PUFA, very long chain n-3 PUFA (EPA+DPA+DHA). *Total number of samples analysed in different publications; †The number of comparisons in which statistically significant difference was found with higher level in ORG; ‡The number of comparisons in which statistically significant difference was found with higher level in CONV; §The number of comparisons in which there was no significant difference between ORG and CONV; \|\|Calculated based on published fatty acids composition data. | | | | | | | | | | |

| **Table S8 cont.** Basic information/statistics on the publications/data used for meta-analyses of composition parameters included in Fig. 2 and 3 in the main paper. | | | | | | | | | | |
| --- | --- | --- | --- | --- | --- | --- | --- | --- | --- | --- |
|  |  |  |  |  | **Number of comparisons reporting that concentrations were** | | | | | |
|  |  |  | **Total sample size*** | | **Numerically higher in** | | **Identical** | **Significantly higher in** | | **Not significantly different**§ |
| **Parameter** | **Studies** | ***n*** | **ORG** | **CONV** | **ORG** | **CONV** |  | **ORG**† | **CONV**‡ |  |
| n-6 FA | 19 | 20 | 545 | 526 | 8 | 11 | 1 | 3 | 4 | 5 |
| LA (cis-9,12-18:2) | 22 | 22 | 311 | 323 | 7 | 15 | 0 | 4 | 7 | 6 |
| AA (cis-5,8,11,14-20:4) | 9 | 9 | 194 | 188 | 0 | 7 | 2 | 0 | 6 | 3 |
| LA/ALA ratio\|\| | 19 | 19 | 269 | 285 | 0 | 19 | 0 | 0 | 0 | 0 |
| n-6/n-3 ratio | 22 | 23 | 308 | 304 | 0 | 23 | 0 | 0 | 9 | 3 |
| n-3/n-6 ratio | 23 | 24 | 310 | 307 | 23 | 1 | 0 | 9 | 0 | 0 |
| α-tocopherol | 16 | 17 | 123 | 147 | 12 | 4 | 1 | 3 | 0 | 4 |
| Carotenoids | 4 | 5 | 38 | 60 | 2 | 3 | 0 | 1 | 0 | 2 |
| β-carotene | 13 | 14 | 129 | 159 | 8 | 5 | 1 | 3 | 1 | 4 |
| Lutein | 5 | 6 | 38 | 60 | 5 | 1 | 0 | 2 | 0 | 2 |
| Zeaxanthin | 5 | 6 | 38 | 60 | 5 | 1 | 0 | 3 | 1 | 0 |
| Iodine (I) | 7 | 7 | 194 | 172 | 0 | 7 | 0 | 0 | 4 | 1 |
| Iron (Fe) | 9 | 9 | 85 | 67 | 6 | 3 | 0 | 2 | 0 | 2 |
| Selenium (Se) | 8 | 8 | 100 | 85 | 2 | 6 | 0 | 0 | 1 | 2 |
| Urea | 11 | 11 | 208 | 217 | 4 | 7 | 0 | 0 | 2 | 6 |
| SCC | 47 | 47 | 3012 | 18429 | 32 | 15 | 0 | 4 | 3 | 18 |
| *n*, numbers of data points (comparisons) included in the meta-analysis; ORG, organic samples; CONV, conventional samples; FA, fatty acids; LA, linoleic acid; AA, arachidonic acid; ALA, α-linolenic acid; SCC, somatic cell count. *The number of comparisons in which statistically significant difference was found with higher level in ORG; †The number of comparisons in which statistically significant difference was found with higher level in CONV; ‡The number of comparisons in which there was no significant difference between ORG and CONV; §Calculated based on published fatty acids composition data. | | | | | | | | | | |

| **Table S9.** Mean percentage differences (MPD) and confidence intervals (CI) calculated using the data included in standard meta-analyses and sensitivity analysis 1 of composition parameters shown in Fig. 2 and 3 of the main paper (MPDs are also shown as symbols in Fig. 2). | | | | | | | |
| --- | --- | --- | --- | --- | --- | --- | --- |
|  | **Standard meta-analysis** | | |  | **Sensitivity analysis 1** | | |
| **Parameter** | ***n*** | **MPD*** | **95% CI** |  | ***n*** | **MPD*** | **95% CI** |
| Milk yield | 32 | -22.49 | -30.47, -14.52 |  | 81 | -19.57 | -23.62, -15.52 |
| SFA | 19 | -0.69 | -2.24, 0.86 |  | 33 | -0.80 | -1.96, 0.37 |
| 12:0 (lauric acid) | 11 | -3.59 | -10.22, 3.03 |  | 17 | -1.98 | -8.12, 4.16 |
| 14:0 (myristic acid) | 12 | 1.02 | -2.60, 4.63 |  | 18 | 1.57 | -1.60, 4.74 |
| 16:0 (palmitic acid) | 14 | -4.65 | -8.45, -0.85 |  | 20 | -3.74 | -6.81, -0.67 |
| MUFA | 19 | 1.20 | -3.13, 5.53 |  | 31 | -0.15 | -3.34, 3.04 |
| OA (cis-9-18:1) | 10 | 2.78 | -3.32, 8.88 |  | 16 | 1.41 | -3.29, 6.10 |
| VA (trans-11-18:1) | 12 | 65.91 | 19.70, 112.12 |  | 18 | 58.07 | 27.01, 89.12 |
| PUFA | 19 | 7.30 | -0.73, 15.34 |  | 30 | 14.78 | 7.05, 22.51 |
| CLA (total) | 11 | 41.13 | 14.19, 68.08 |  | 19 | 47.47 | 20.78, 74.16 |
| CLA9 (cis-9-trans-11-18:2) | 14 | 23.89 | 8.39, 39.39 |  | 20 | 34.36 | 17.93, 50.80 |
| CLA10 (trans-10-cis-12-18:2) | 3 | 28.24 | -20.92, 77.40 |  | 7 | 34.96 | 2.94, 66.98 |
| n-3 FA | 12 | 55.67 | 37.68, 73.66 |  | 20 | 60.14 | 45.07, 75.20 |
| ALA (cis-9,12,15-18:3) | 21 | 68.62 | 53.04, 84.20 |  | 34 | 78.66 | 66.04, 91.29 |
| EPA (cis-5,8,11,14,17-20:5) | 8 | 67.14 | 32.35, 101.94 |  | 14 | 66.34 | 39.86, 92.82 |
| DPA (cis-7,10,13,16,19-22:5) | 5 | 44.83 | 18.23, 71.44 |  | 8 | 38.23 | 20.57, 55.89 |
| DHA (cis-4,7,10,13,16,19-22:6) | 3 | 21.48 | -3.71, 46.67 |  | 6 | 194.07 | -89.14, 477.29 |
| VLC n-3 PUFA† | - | - | - |  | 5 | 57.16 | 27.25, 87.07 |
| *n*, number of data points included in the comparison; MPD, mean percentage difference; SFA, saturated fatty acids; MUFA, monounsaturated fatty acids; OA, oleic acid; VA, vaccenic acid; PUFA, polyunsaturated fatty acids; FA, fatty acids; CLA, conjugated linoleic acid; ALA, α-linolenic acid; EPA, eicosapentaenoic acid; DPA, docosapentaenoic acid; DHA, docosahexaenoic acid; VLC n-3 PUFA, very long chain n-3 PUFA (EPA+DPA+DHA). *Magnitude of difference between organic (ORG) and conventional (CONV) samples (value <0 indicate higher concentration in CONV, value >0 indicate higher concentration in ORG); †Calculated based on published fatty acids composition data. | | | | | | | |

| **Table S9 cont.** Mean percentage differences (MPD) and confidence intervals (CI) calculated using the data included in standard meta-analyses and sensitivity analysis 1 of composition parameters shown in Fig. 2 and 3 of the main paper (MPDs are also shown as symbols in Fig. 2). | | | | | | | |
| --- | --- | --- | --- | --- | --- | --- | --- |
|  | **Standard meta-analysis** | | |  | **Sensitivity analysis 1** | | |
| **Parameter** | ***n*** | **MPD*** | **95% CI** |  | ***n*** | **MPD*** | **95% CI** |
| n-6 FA | 12 | -4.03 | -13.83, 5.76 |  | 20 | -1.50 | -10.62, 7.61 |
| LA (cis-9,12-18:2) | 12 | -14.40 | -29.51, 0.71 |  | 22 | -4.82 | -15.27, 5.64 |
| AA (cis-5,8,11,14-20:4) | 5 | -24.15 | -41.00, -7.30 |  | 9 | -20.58 | -30.83, -10.32 |
| LA/ALA ratio† | - | - | - |  | 19 | -93.34 | -116.41, -70.28 |
| n-6/n-3 ratio | 7 | -71.16 | -122.01, -20.31 |  | 23 | -72.07 | -92.86, -51.29 |
| n-3/n-6 ratio | 5 | 72.21 | 36.08, 108.35 |  | 24 | 64.95 | 44.22, 85.67 |
| α-tocopherol | 9 | 12.98 | 0.51, 25.45 |  | 17 | 11.68 | 2.52, 20.84 |
| Carotenoids | 5 | 31.83 | -37.01, 100.66 |  | 5 | 31.83 | -37.01, 100.66 |
| β-carotene | 7 | 0.64 | -14.55, 15.82 |  | 14 | 27.79 | -2.40, 57.97 |
| Lutein | 3 | 12.71 | -46.12, 71.54 |  | 6 | 104.08 | -33.66, 241.82 |
| Zeaxanthin | - | - | - |  | 6 | 38.99 | 1.43, 76.55 |
| Iodine (I) | 6 | -73.85 | -115.19, -32.5 |  | 7 | -73.08 | -108.05, -38.10 |
| Iron (Fe) | 8 | 20.18 | -0.10, 40.46 |  | 9 | 16.59 | -2.63, 35.81 |
| Selenium (Se) | 4 | -21.42 | -48.93, 6.09 |  | 8 | -28.06 | -69.25, 13.13 |
| Urea | 7 | -9.67 | -24.70, 5.36 |  | 11 | -8.75 | -19.64, 2.14 |
| SCC | 20 | 8.19 | -12.98, 29.36 |  | 47 | 1.15 | -22.52, 24.82 |
| *n*, number of data points included in the comparison; MPD, mean percentage difference; FA, fatty acids; LA, linoleic acid; AA, arachidonic acid; ALA, α-linolenic acid; SCC, somatic cell count. *Magnitude of difference between organic (ORG) and conventional (CONV) samples (value <0 indicate higher concentration in CONV, value >0 indicate higher concentration in ORG); †Calculated based on published fatty acids composition data. | | | | | | | |

| **Table S10.** Mean values and confidence intervals (CI) calculated using the data for all papers reporting means of composition parameters shown in Fig. 2 and 3 of the main paper. | | | | | | | |
| --- | --- | --- | --- | --- | --- | --- | --- |
|  |  |  | **Organic** | |  | **Conventional** | |
| **Parameter** | **Unit** | ***n*** | **Mean** | **95% CI** |  | **Mean** | **95% CI** |
| Milk yield* | kg/cow/day | 51 | 18.76 | 17.49, 20.03 |  | 22.53 | 20.99, 24.06 |
| Milk yield* | kg/cow/lactation | 16 | 6451 | 5976, 6926 |  | 7550 | 6969, 8132 |
| SFA | mg/g FA | 32 | 678.41 | 665.44, 691.39 |  | 683.82 | 669.78, 697.85 |
| 12:0 (lauric acid) | mg/g FA | 17 | 33.32 | 29.93, 36.70 |  | 33.79 | 30.75, 36.83 |
| 14:0 (myristic acid) | mg/g FA | 18 | 113.08 | 106.61, 119.55 |  | 111.23 | 105.60, 116.85 |
| 16:0 (palmitic acid) | mg/g FA | 20 | 304.35 | 289.80, 318.90 |  | 315.14 | 300.57, 329.71 |
| MUFA | mg/g FA | 30 | 271.96 | 261.46, 282.46 |  | 272.25 | 262.79, 281.71 |
| OA (cis-9-18:1) | mg/g FA | 16 | 225.00 | 205.20, 244.80 |  | 222.38 | 202.05, 242.71 |
| VA (trans-11-18:1) | mg/g FA | 18 | 23.82 | 20.57, 27.08 |  | 16.30 | 14.02, 18.58 |
| PUFA | mg/g FA | 29 | 41.42 | 37.57, 45.26 |  | 36.31 | 33.48, 39.14 |
| CLA (total) | mg/g FA | 18 | 9.72 | 8.00, 11.43 |  | 6.98 | 5.55, 8.41 |
| CLA9 (cis-9-trans-11-18:2) | mg/g FA | 20 | 8.66 | 6.87, 10.46 |  | 6.71 | 5.17, 8.25 |
| CLA10 (trans-10-cis-12-18:2) | mg/g FA | 7 | 0.55 | 0.16, 0.94 |  | 0.38 | 0.15, 0.62 |
| n-3 FA | mg/g FA | 19 | 10.22 | 9.04, 11.41 |  | 6.69 | 5.53, 7.84 |
| ALA (cis-9,12,15-18:3) | mg/g FA | 33 | 7.73 | 7.02, 8.43 |  | 4.38 | 3.96, 4.80 |
| EPA (cis-5,8,11,14,17-20:5) | mg/g FA | 13 | 0.87 | 0.72, 1.02 |  | 0.56 | 0.45, 0.66 |
| DPA (cis-7,10,13,16,19-22:5) | mg/g FA | 8 | 1.02 | 0.85, 1.18 |  | 0.76 | 0.59, 0.93 |
| DHA (cis-4,7,10,13,16,19-22:6) | mg/g FA | 6 | 0.29 | 0.00, 0.58 |  | 0.09 | 0.05, 0.13 |
| VLC n-3 PUFA† | mg/g FA | 5 | 2.10 | 1.68, 2.52 |  | 1.38 | 1.04, 1.71 |
| *n*, number of data points included in the comparison; MPD, mean percentage difference; SFA, saturated fatty acids; MUFA, monounsaturated fatty acids; OA, oleic acid; VA, vaccenic acid; PUFA, polyunsaturated fatty acids; FA, fatty acids; CLA, conjugated linoleic acid; ALA, α-linolenic acid; EPA, eicosapentaenoic acid; DPA, docosapentaenoic acid; DHA, docosahexaenoic acid; VLC n-3 PUFA, very long chain n-3 PUFA (EPA+DPA+DHA). *Data for energy-, fat-, protein- corrected milk yield and milk solids yield were removed from calculations; †Calculated based on published fatty acids composition data. | | | | | | | |

| **Table S10 cont.** Mean values and confidence intervals (CI) calculated using the data for all papers reporting means of composition parameters shown in Fig. 2 and 3 of the main paper. | | | | | | | |
| --- | --- | --- | --- | --- | --- | --- | --- |
|  |  |  | **Organic** | |  | **Conventional** | |
| **Parameter** | **Unit** | ***n*** | **Mean** | **95% CI** |  | **Mean** | **95% CI** |
| n-6 FA | mg/g FA | 19 | 22.45 | 20.28, 24.61 |  | 22.79 | 20.47, 25.1 |
| LA (cis-9,12-18:2) | mg/g FA | 22 | 19.33 | 17.93, 20.73 |  | 20.63 | 18.15, 23.1 |
| AA (cis-5,8,11,14-20:4) | mg/g FA | 9 | 1.03 | 0.88, 1.18 |  | 1.24 | 1.06, 1.41 |
| LA/ALA ratio† | - | 19 | 2.76 | 1.95, 3.56 |  | 4.95 | 4.02, 5.88 |
| n-6/n-3 ratio | - | 22 | 3.56 | 1.9, 5.23 |  | 5.42 | 3.42, 7.42 |
| n-3/n-6 ratio | - | 23 | 0.42 | 0.34, 0.5 |  | 0.26 | 0.2, 0.31 |
| α-tocopherol | µg/g fat | 16 | 21.85 | 17.55, 26.15 |  | 20.13 | 16, 24.26 |
| Carotenoids | µg/g fat | 4 | 6.70 | 4.26, 9.15 |  | 6.75 | 2.89, 10.61 |
| β-carotene | µg/g fat | 13 | 5.37 | 4.3, 6.45 |  | 4.78 | 3.56, 5.99 |
| Lutein | µg/g fat | 5 | 0.56 | 0.36, 0.76 |  | 0.47 | 0.11, 0.82 |
| Zeaxanthin | µg/g fat | 5 | 0.23 | 0, 0.47 |  | 0.24 | -0.08, 0.56 |
| Iodine (I) | µg/L | 7 | 147.32 | 87.65, 207 |  | 247.63 | 153.04, 342.22 |
| Iron (Fe) | mg/kg | 9 | 1.03 | 0.38, 1.68 |  | 0.98 | 0.26, 1.71 |
| Selenium (Se) | µg/kg | 8 | 13.84 | 11.25, 16.42 |  | 17.61 | 10.85, 24.37 |
| Urea* | mg/kg | 10 | 218.97 | 205.65, 232.3 |  | 237.30 | 210.29, 264.3 |
| SCC | cells/ml ×10^3^ | 47 | 218.62 | 177.22, 260.01 |  | 211.06 | 164.87, 257.25 |
| *n*, number of data points included in the comparison; MPD, mean percentage difference; FA, fatty acids; LA, linoleic acid; AA, arachidonic acid; ALA, α-linolenic acid; SCC, somatic cell count. *One outlying value (1000 times greater than other values) was removed; †Calculated based on published fatty acids composition data. | | | | | | | |

| **Table S11.** Meta-analysis results for addition composition parameters for which significant differences were detected by the standard meta-analysis or one of the sensitivity analyses (see also Appendix Table A1 and A2 for results). | | | | | | | | | | | | | |
| --- | --- | --- | --- | --- | --- | --- | --- | --- | --- | --- | --- | --- | --- |
|  | **Standard meta-analysis** | | | | | | |  | **Sensitivity analysis 1** | | | | |
| **Parameter** | ***n*** | **SMD** | **95% CI** | ***P**** | **Heterogen.**† | **MPD**‡ | **95% CI** |  | ***n*** | **Ln ratio**§ | ***P**** | **MPD**‡ | **95% CI** |
| Fat | 31 | -0.29 | -0.63, 0.05 | 0.092 | Yes (85%) | -1.37 | -3.66, 0.91 |  | 58 | 4.60 | 0.329 | -0.45 | -2.32, 1.43 |
| Protein | 29 | -0.17 | -0.55, 0.21 | 0.368 | Yes (88%) | -0.24 | -1.80, 1.33 |  | 56 | 4.60 | 0.146 | -0.64 | -1.84, 0.56 |
| Solids | 8 | 0.64 | -0.23, 1.52 | 0.149 | Yes (86%) | 1.05 | -0.45, 2.55 |  | 13 | 4.62 | 0.022 | 1.50 | 0.11, 2.89 |
| Solids (no-fat) | 4 | 0.24 | -0.03, 0.51 | 0.083 | No (0%) | 1.37 | -0.75, 3.49 |  | 7 | 4.62 | 0.094 | 1.08 | -0.30, 2.47 |
| Free fatty acids | - | - | - | - | - | - | - |  | 3 | 4.55 | 0.247 | -5.91 | -13.97, 2.15 |
| 8:0 (caprylic acid) | 9 | -0.03 | -0.64, 0.59 | 0.936 | Yes (81%) | -1.44 | -7.56, 4.68 |  | 16 | 4.64 | 0.123 | 3.64 | -2.42, 9.70 |
| 15:0 (pentadecanoic acid) | 8 | 1.61 | -0.39, 3.60 | 0.115 | Yes (98%) | 7.15 | -0.26, 14.56 |  | 13 | 4.70 | 0.002 | 10.24 | 5.09, 15.39 |
| 17:0 (heptadecanoic acid) | 9 | 0.72 | -0.45, 1.89 | 0.226 | Yes (95%) | 9.71 | -2.09, 21.52 |  | 11 | 4.69 | 0.010 | 9.87 | 0.26, 19.48 |
| 20:0 (arachidic acid) | 4 | 0.73 | -0.76, 2.22 | 0.336 | Yes (96%) | 13.64 | -2.34, 29.61 |  | 9 | 4.70 | 0.042 | 10.72 | 0.40, 21.05 |
| SRR/RRR | 3 | -3.27 | -6.81, 0.28 | 0.071 | Yes (93%) | -269.48 | -552.49, 13.53 |  | 4 | 3.63 | 0.064 | -216.60 | -441.96, 8.76 |
| 22:0 (behenic acid) | 3 | 1.27 | -0.85, 3.39 | 0.239 | Yes (94%) | 30.88 | -7.82, 69.59 |  | 7 | 4.75 | 0.158 | 17.70 | -12.19, 47.59 |
| 24:0 (lignoceric acid) | - | - | - | - | - | - | - |  | 5 | 4.78 | 0.065 | 20.84 | 2.57, 39.11 |
| *n*, number of data points included in the comparison; SMD, standardised mean difference; MPD, mean percentage difference; SRR/RRR, Phytanic acid diastereomers ratio. **P* value <0.05 indicates significance of the difference in composition between organic and conventional milk; †Heterogeneity and the I^2^ Statistic; ‡Magnitude of difference between organic (ORG) and conventional (CONV) samples (value <0 indicate higher concentration in CONV, value >0 indicate higher concentration in ORG); §Ln ratio = Ln(ORG/CONV × 100%). | | | | | | | | | | | | | |

| **Table S11 cont.** Meta-analysis results for addition composition parameters for which significant differences were detected by the standard meta-analysis or one of the sensitivity analyses (see also Appendix Table A1 and A2 for results). | | | | | | | | | | | | | |
| --- | --- | --- | --- | --- | --- | --- | --- | --- | --- | --- | --- | --- | --- |
|  | **Standard meta-analysis** | | | | | | |  | **Sensitivity analysis 1** | | | | |
| **Parameter** | ***n*** | **SMD** | **95% CI** | ***P**** | **Heterogen.**† | **MPD**‡ | **95% CI** |  | ***n*** | **Ln ratio**§ | ***P**** | **MPD**‡ | **95% CI** |
| trans-18:1 | 4 | 0.39 | -0.4, 1.18 | 0.337 | Yes (63%) | 50.43 | -24.94, 125.80 |  | 6 | 4.94 | 0.047 | 49.36 | -0.64, 99.37 |
| cis-9-20:1 | - | - | - | - | - | - | - |  | 3 | 4.84 | 0.247 | 29.26 | -7.76, 66.27 |
| DGLA (cis-8-11-14-C20:3) | - | - | - | - | - | - | - |  | 4 | 4.40 | 0.122 | -23.89 | -40.50, -7.28 |
| GLA (cis-6,9,12-18:3) | 4 | 0.20 | -0.19, 0.59 | 0.311 | No (9%) | 741.67 | -605.31, 2088.65 |  | 7 | 5.29 | 0.032 | 430.60 | -344.65, 1205.84 |
| 18:4 | - | - | - | - | - | - | - |  | 3 | 4.99 | 0.251 | 68.89 | -59.65, 197.43 |
| 2R | - | - | - | - | - | - | - |  | 5 | 4.27 | 0.062 | -46.38 | -86.65, -6.11 |
| 3R | - | - | - | - | - | - | - |  | 6 | 4.81 | 0.032 | 24.75 | 1.59, 47.90 |
| Vitamin A | 4 | -2.59 | -7.81, 2.63 | 0.331 | Yes (99%) | -56.18 | -155.88, 43.53 |  | 10 | 4.43 | 0.019 | -27.31 | -67.22, 12.60 |
| Copper (Cu) | 8 | -0.57 | -1.16, 0.02 | 0.060 | Yes (60%) | -17.26 | -28.43, -6.10 |  | 10 | 4.50 | 0.049 | -12.37 | -25.04, 0.30 |
| Potassium (K) | 4 | 0.30 | -0.02, 0.62 | 0.063 | No (0%) | 4.49 | 1.35, 7.62 |  | 7 | 4.63 | 0.091 | 2.30 | -0.34, 4.94 |
| *n*, number of data points included in the comparison; SMD, standardised mean difference; MPD, mean percentage difference; DGLA, dihomo-γ-linolenic acid; GLA, γ-linolenic acid; 2R, synthetic isomers of α-tocopherol; 3R, natural isomers of α-tocopherol. **P* value <0.05 indicates significance of the difference in composition between organic and conventional milk; †Heterogeneity and the I^2^ Statistic; ‡Magnitude of difference between organic (ORG) and conventional (CONV) samples (value <0 indicate higher concentration in CONV, value >0 indicate higher concentration in ORG); §Ln ratio = Ln(ORG/CONV × 100%). | | | | | | | | | | | | | |


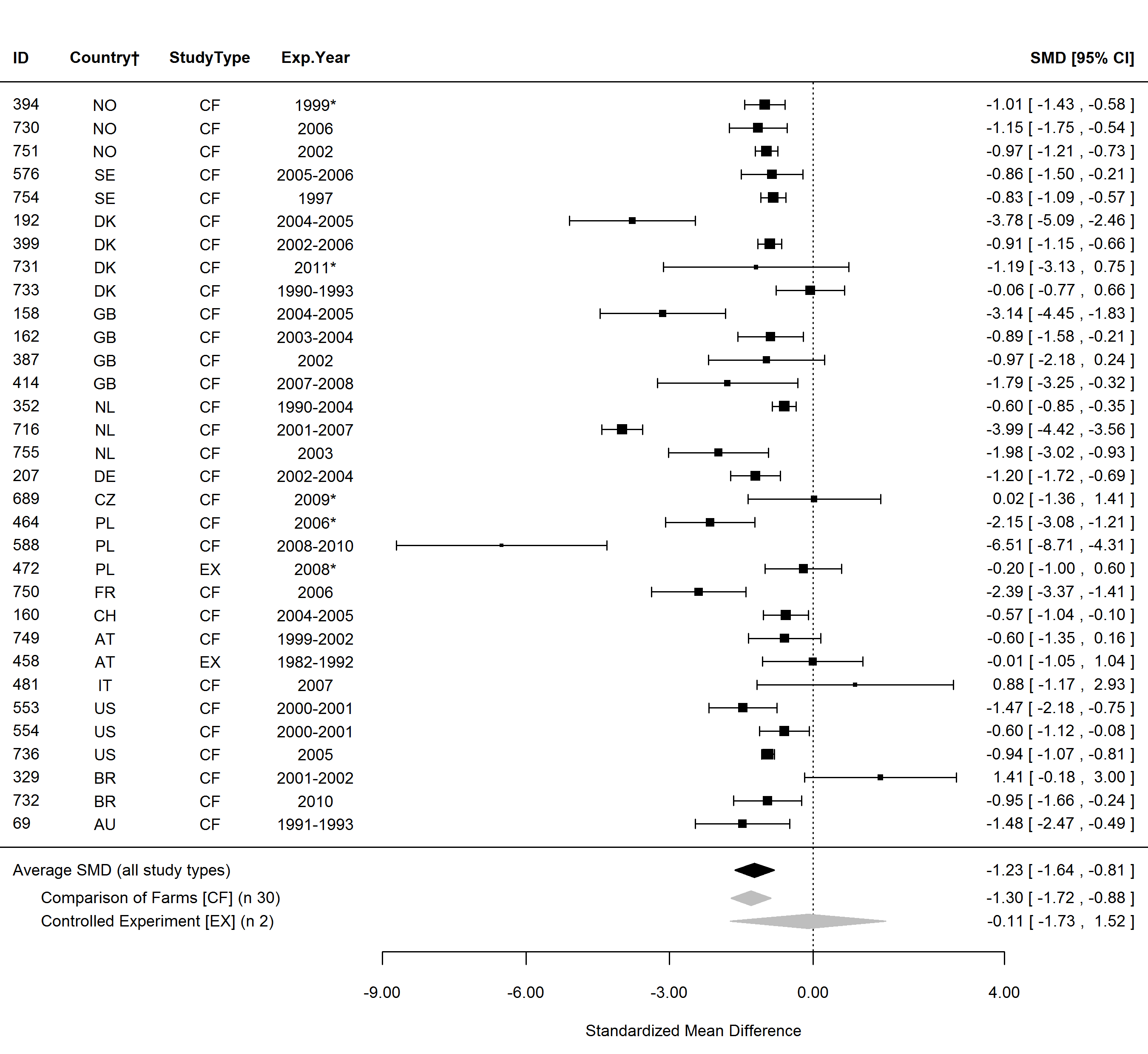


**Figure S3.** Forest plot showing the results of studies examining the milk yield in organic and conventional bovine milk. The figure shows the standardised mean differences (SMDs) with 95% confidence intervals, for studies included in standard meta-analysis. The estimated average SMD for all studies and SMDs for different study types are indicated at the bottom of the figure. Sign of the SMD indicates if the analysed parameter is higher (+) or lower (-) in organic milk. ID, Paper unique identification number (see supplementary Table S1 for references); CF, comparison of farms, BS, basket study, EX, controlled experiment. *No information about the experimental year (estimated as publication year -2), †Country codes according ISO 3166-2 (see [*http://www.iso.org/iso/home/standards/country_codes.htm*](http://www.iso.org/iso/home/standards/country_codes.htm)).


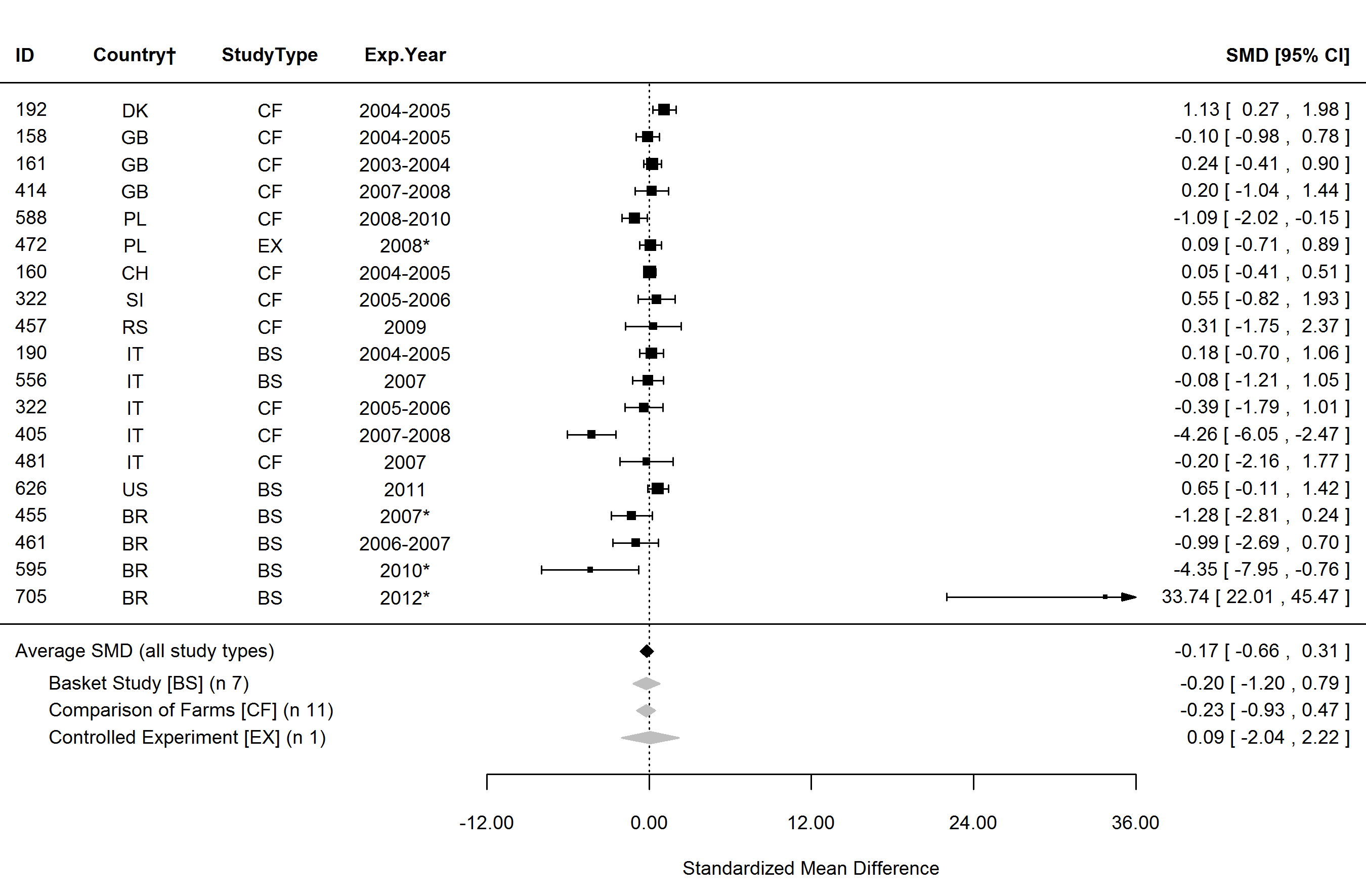


**Figure S4.** Forest plot showing the results of studies examining the saturated fatty acids (SFA) in organic and conventional bovine milk. The figure shows the standardised mean differences (SMDs) with 95% confidence intervals, for studies included in standard meta-analysis. The estimated average SMD for all studies and SMDs for different study types are indicated at the bottom of the figure. Sign of the SMD indicates if the analysed parameter is higher (+) or lower (-) in organic milk. ID, Paper unique identification number (see supplementary Table S1 for references); CF, comparison of farms, BS, basket study, EX, controlled experiment. *No information about the experimental year (estimated as publication year -2), †Country codes according ISO 3166-2 (see [*http://www.iso.org/iso/home/standards/country_codes.htm*](http://www.iso.org/iso/home/standards/country_codes.htm)).


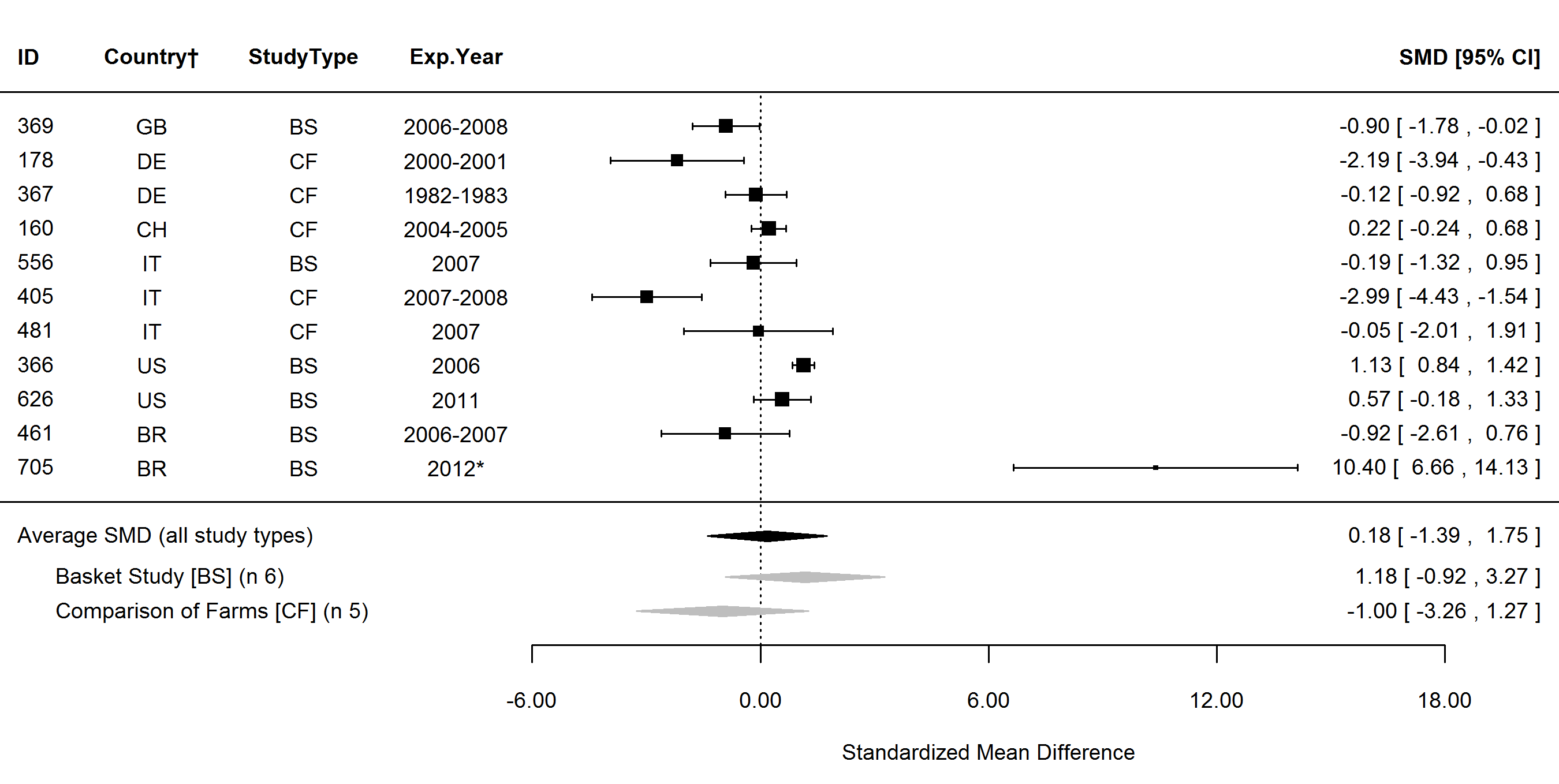


**Figure S5.** Forest plot showing the results of studies examining the 12:0 fatty acid (lauric acid) in organic and conventional bovine milk. The figure shows the standardised mean differences (SMDs) with 95% confidence intervals, for studies included in standard meta-analysis. The estimated average SMD for all studies and SMDs for different study types are indicated at the bottom of the figure. Sign of the SMD indicates if the analysed parameter is higher (+) or lower (-) in organic milk. ID, Paper unique identification number (see supplementary Table S1 for references); CF, comparison of farms, BS, basket study, EX, controlled experiment. *No information about the experimental year (estimated as publication year -2), †Country codes according ISO 3166-2 (see [*http://www.iso.org/iso/home/standards/country_codes.htm*](http://www.iso.org/iso/home/standards/country_codes.htm)).


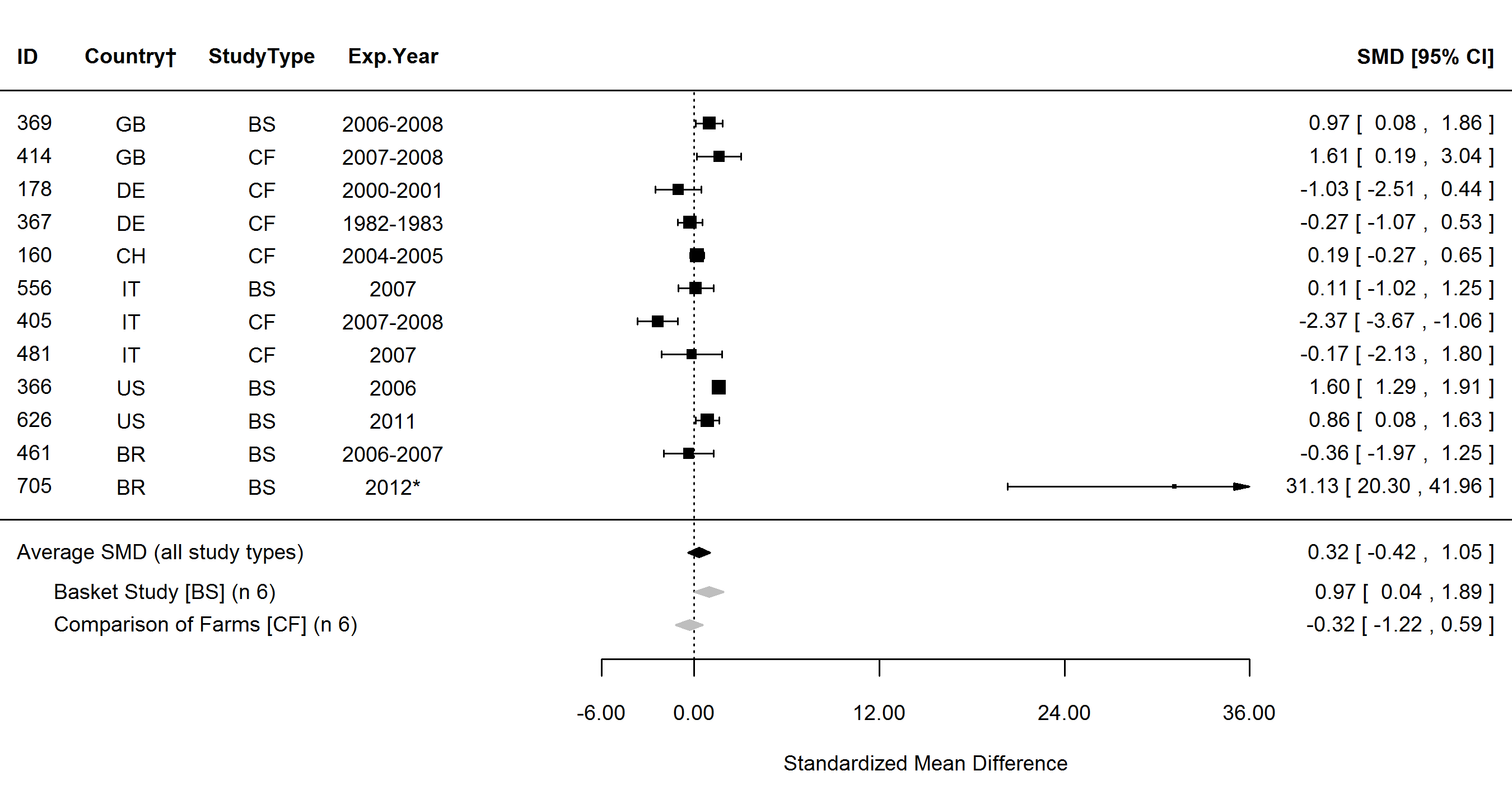


**Figure S6.** Forest plot showing the results of studies examining the 14:0 fatty acid (myristic acid) in organic and conventional bovine milk. The figure shows the standardised mean differences (SMDs) with 95% confidence intervals, for studies included in standard meta-analysis. The estimated average SMD for all studies and SMDs for different study types are indicated at the bottom of the figure. Sign of the SMD indicates if the analysed parameter is higher (+) or lower (-) in organic milk. ID, Paper unique identification number (see supplementary Table S1 for references); CF, comparison of farms, BS, basket study, EX, controlled experiment. *No information about the experimental year (estimated as publication year -2), †Country codes according ISO 3166-2 (see [*http://www.iso.org/iso/home/standards/country_codes.htm*](http://www.iso.org/iso/home/standards/country_codes.htm)).


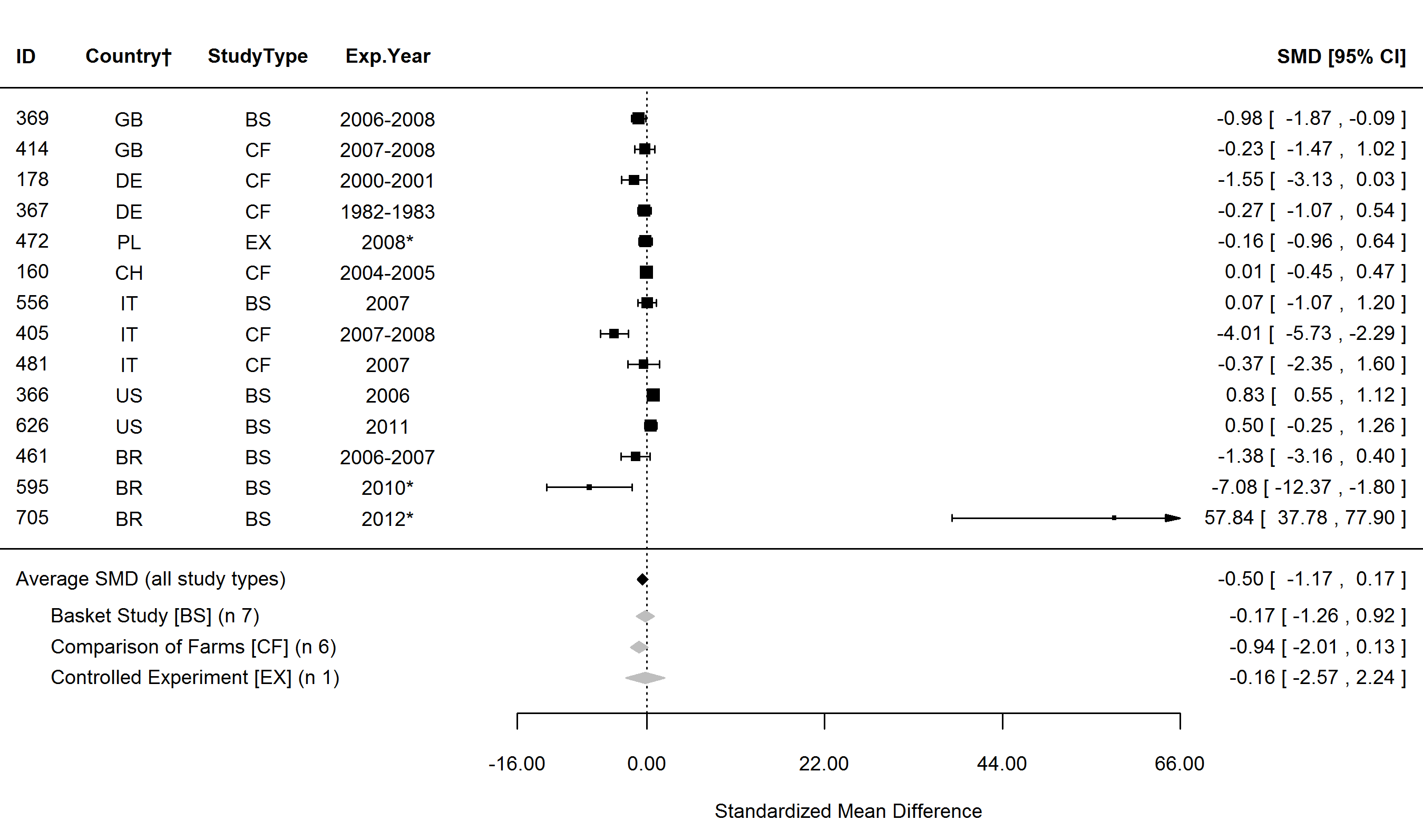


**Figure S7.** Forest plot showing the results of studies examining the 16:0 fatty acid (palmitic acid) in organic and conventional bovine milk. The figure shows the standardised mean differences (SMDs) with 95% confidence intervals, for studies included in standard meta-analysis. The estimated average SMD for all studies and SMDs for different study types are indicated at the bottom of the figure. Sign of the SMD indicates if the analysed parameter is higher (+) or lower (-) in organic milk. ID, Paper unique identification number (see supplementary Table S1 for references); CF, comparison of farms, BS, basket study, EX, controlled experiment. *No information about the experimental year (estimated as publication year -2), †Country codes according ISO 3166-2 (see [*http://www.iso.org/iso/home/standards/country_codes.htm*](http://www.iso.org/iso/home/standards/country_codes.htm)).


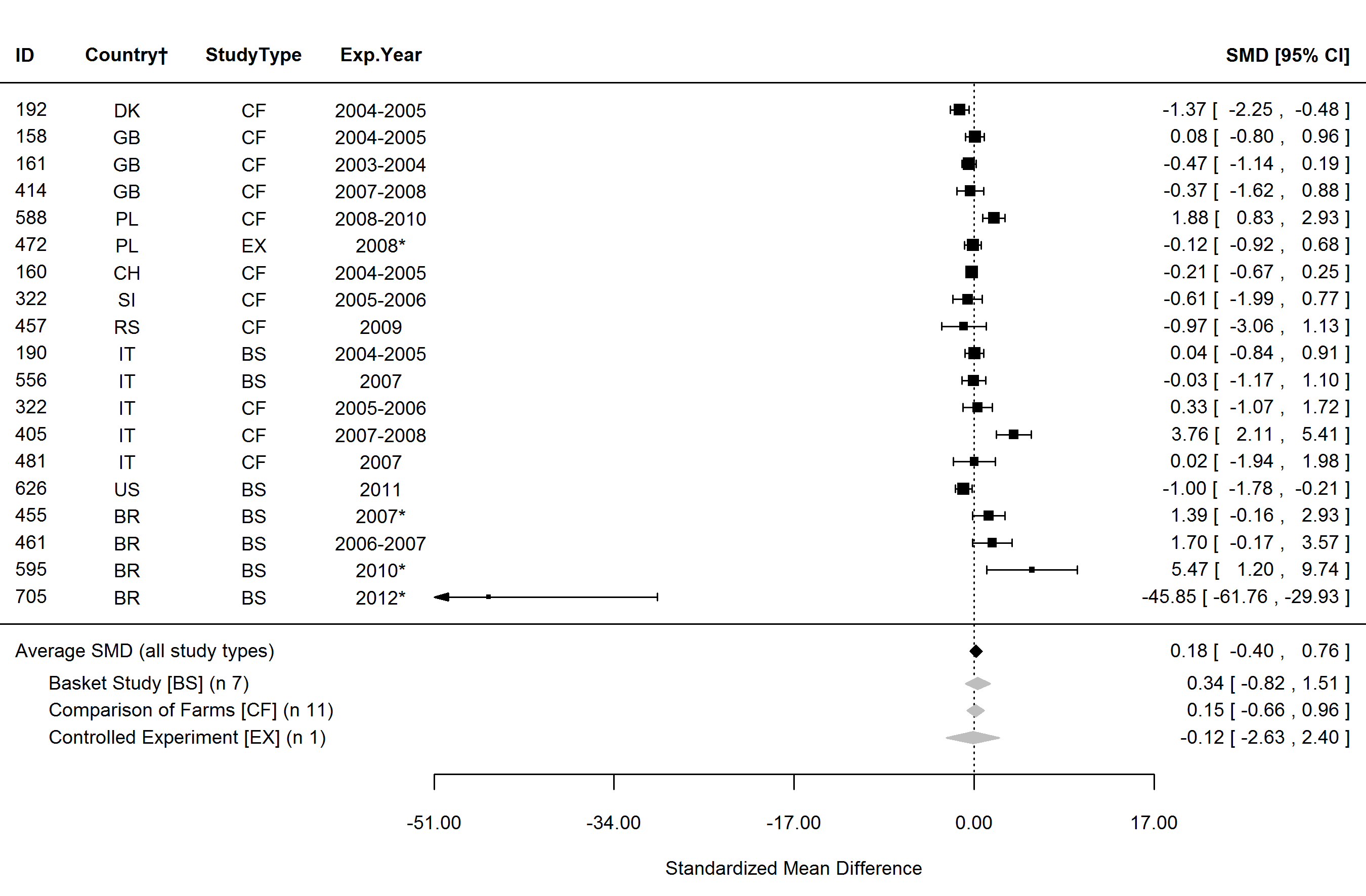


**Figure S8.** Forest plot showing the results of studies examining the monounsaturated fatty acids (MUFA) in organic and conventional bovine milk. The figure shows the standardised mean differences (SMDs) with 95% confidence intervals, for studies included in standard meta-analysis. The estimated average SMD for all studies and SMDs for different study types are indicated at the bottom of the figure. Sign of the SMD indicates if the analysed parameter is higher (+) or lower (-) in organic milk. ID, Paper unique identification number (see supplementary Table S1 for references); CF, comparison of farms, BS, basket study, EX, controlled experiment. *No information about the experimental year (estimated as publication year -2), †Country codes according ISO 3166-2 (see [*http://www.iso.org/iso/home/standards/country_codes.htm*](http://www.iso.org/iso/home/standards/country_codes.htm)).


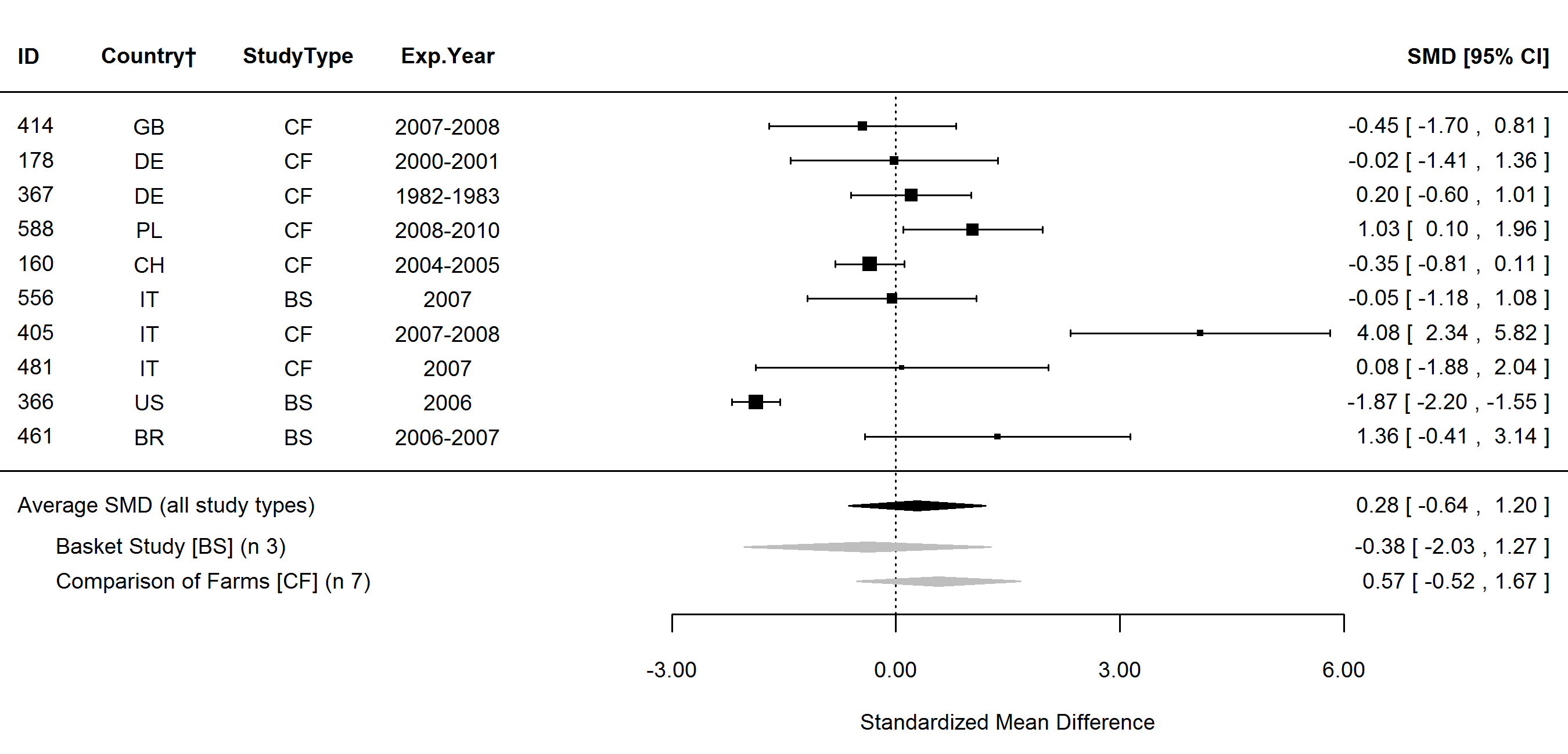


**Figure S9.** Forest plot showing the results of studies examining the oleic fatty acid (*cis*-9-18:1, OA) in organic and conventional bovine milk. The figure shows the standardised mean differences (SMDs) with 95% confidence intervals, for studies included in standard meta-analysis. The estimated average SMD for all studies and SMDs for different study types are indicated at the bottom of the figure. Sign of the SMD indicates if the analysed parameter is higher (+) or lower (-) in organic milk. ID, Paper unique identification number (see supplementary Table S1 for references); CF, comparison of farms, BS, basket study, EX, controlled experiment. *No information about the experimental year (estimated as publication year -2), †Country codes according ISO 3166-2 (see [*http://www.iso.org/iso/home/standards/country_codes.htm*](http://www.iso.org/iso/home/standards/country_codes.htm)).


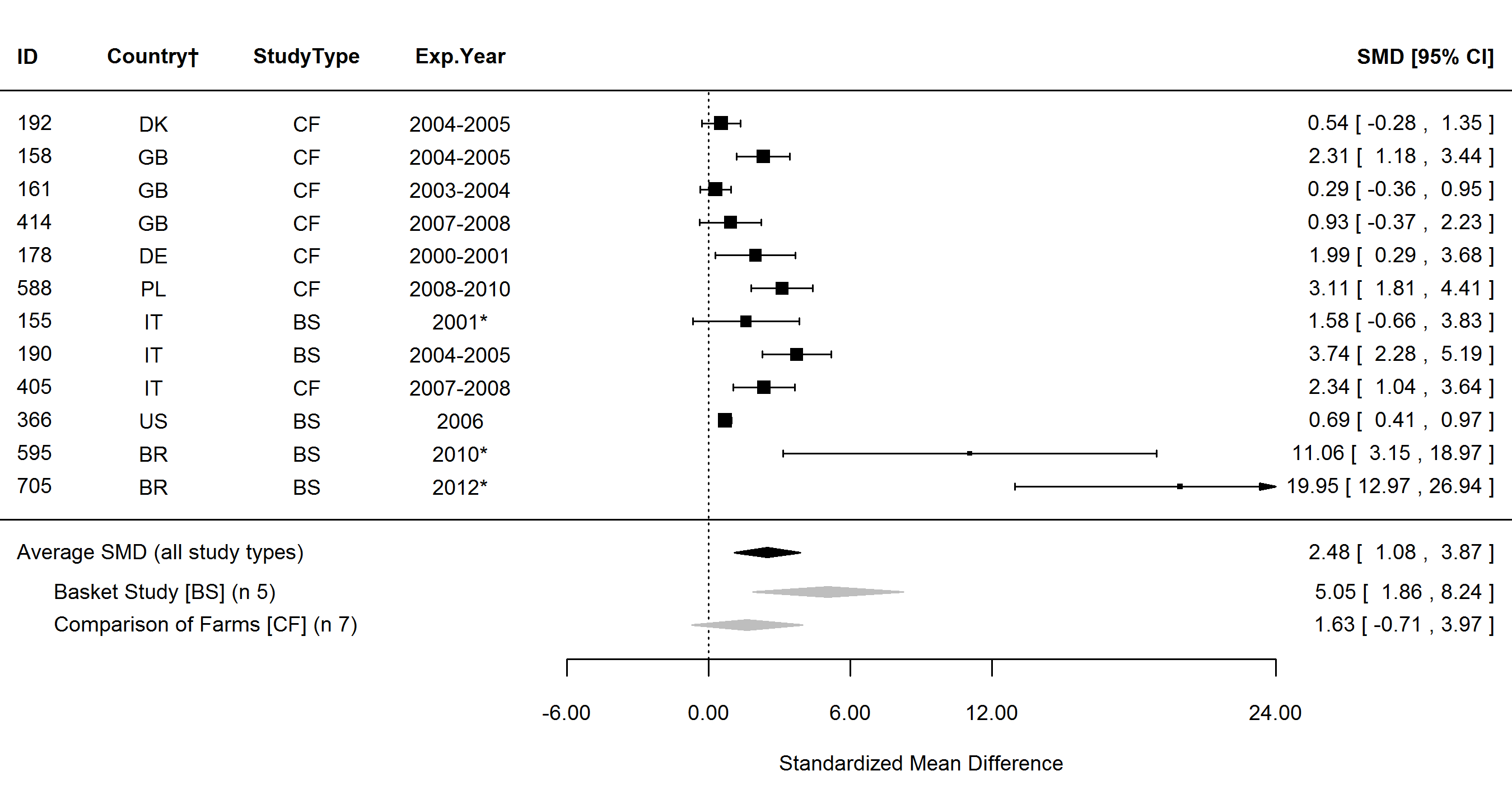


**Figure S10.** Forest plot showing the results of studies examining the vaccenic fatty acid (*trans*-11-18:1, VA) in organic and conventional bovine milk. The figure shows the standardised mean differences (SMDs) with 95% confidence intervals, for studies included in standard meta-analysis. The estimated average SMD for all studies and SMDs for different study types are indicated at the bottom of the figure. Sign of the SMD indicates if the analysed parameter is higher (+) or lower (-) in organic milk. ID, Paper unique identification number (see supplementary Table S1 for references); CF, comparison of farms, BS, basket study, EX, controlled experiment. *No information about the experimental year (estimated as publication year -2), †Country codes according ISO 3166-2 (see [*http://www.iso.org/iso/home/standards/country_codes.htm*](http://www.iso.org/iso/home/standards/country_codes.htm)).


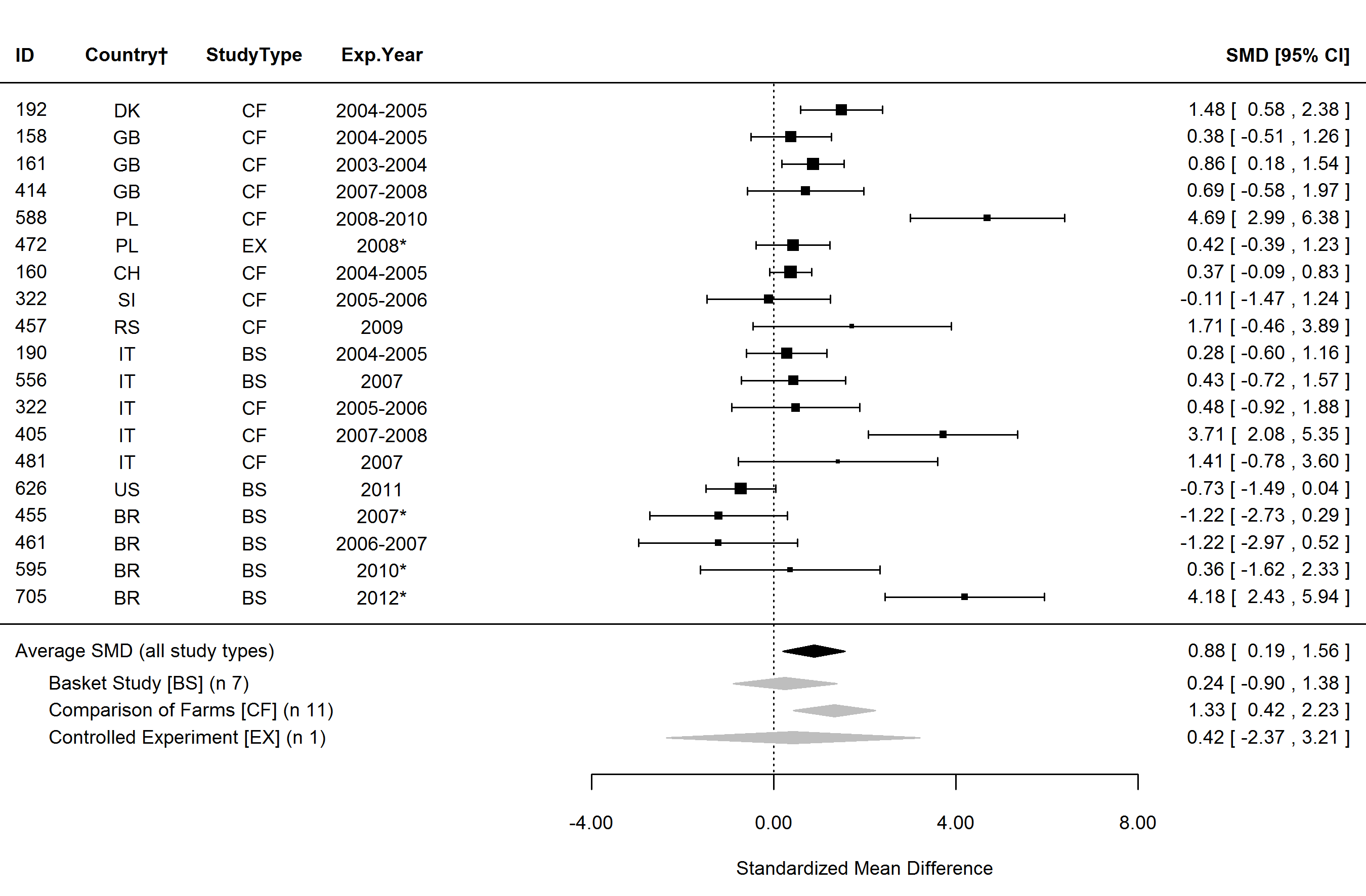


**Figure S11.** Forest plot showing the results of studies examining the polyunsaturated fatty acids (PUFA) in organic and conventional bovine milk. The figure shows the standardised mean differences (SMDs) with 95% confidence intervals, for studies included in standard meta-analysis. The estimated average SMD for all studies and SMDs for different study types are indicated at the bottom of the figure. Sign of the SMD indicates if the analysed parameter is higher (+) or lower (-) in organic milk. ID, Paper unique identification number (see supplementary Table S1 for references); CF, comparison of farms, BS, basket study, EX, controlled experiment. *No information about the experimental year (estimated as publication year -2), †Country codes according ISO 3166-2 (see [*http://www.iso.org/iso/home/standards/country_codes.htm*](http://www.iso.org/iso/home/standards/country_codes.htm)).


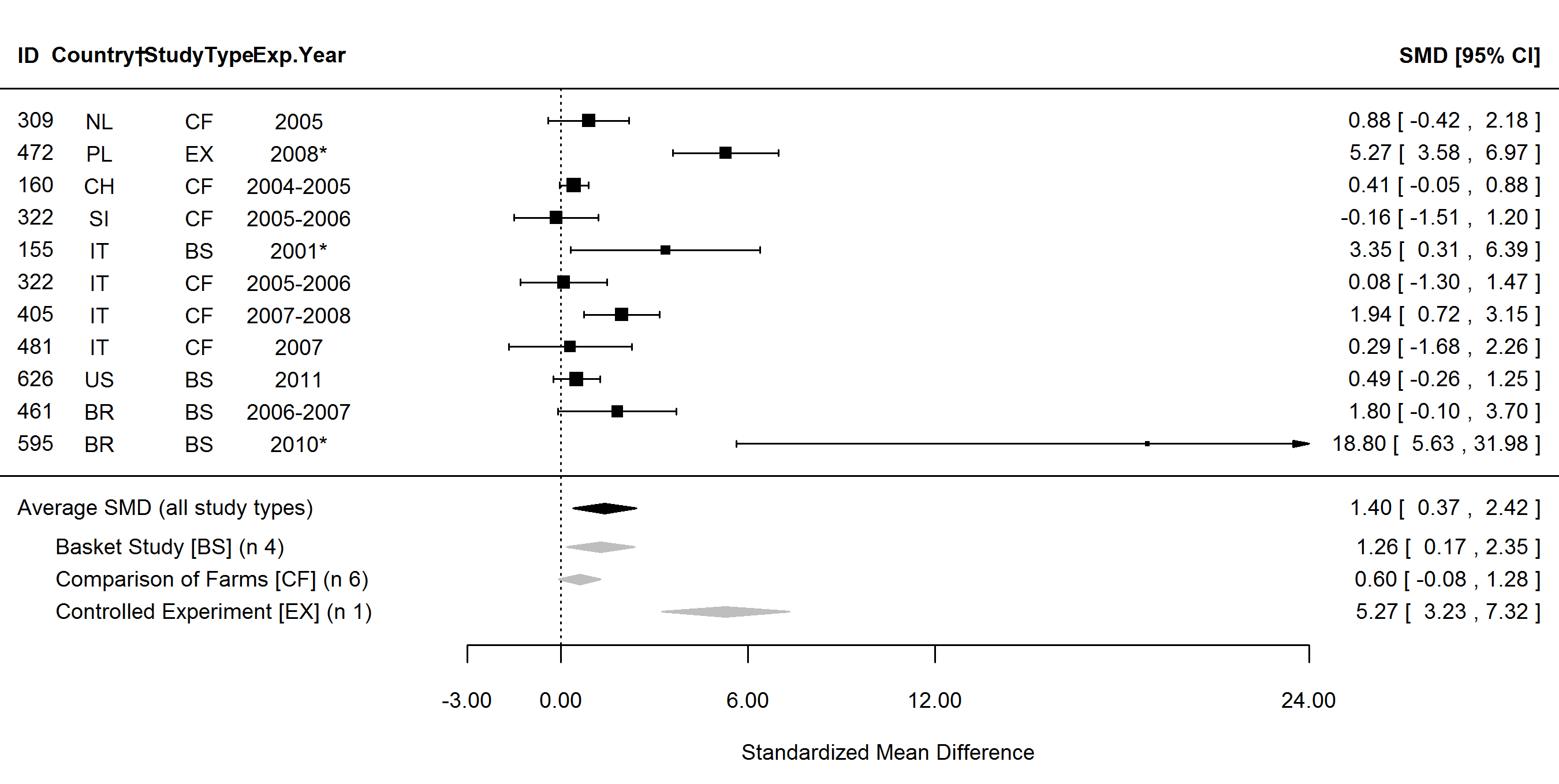


**Figure S12.** Forest plot showing the results of studies examining the total conjugated linoleic fatty acids (CLA total) in organic and conventional bovine milk. The figure shows the standardised mean differences (SMDs) with 95% confidence intervals, for studies included in standard meta-analysis. The estimated average SMD for all studies and SMDs for different study types are indicated at the bottom of the figure. Sign of the SMD indicates if the analysed parameter is higher (+) or lower (-) in organic milk. ID, Paper unique identification number (see supplementary Table S1 for references); CF, comparison of farms, BS, basket study, EX, controlled experiment. *No information about the experimental year (estimated as publication year -2), †Country codes according ISO 3166-2 (see [*http://www.iso.org/iso/home/standards/country_codes.htm*](http://www.iso.org/iso/home/standards/country_codes.htm)).


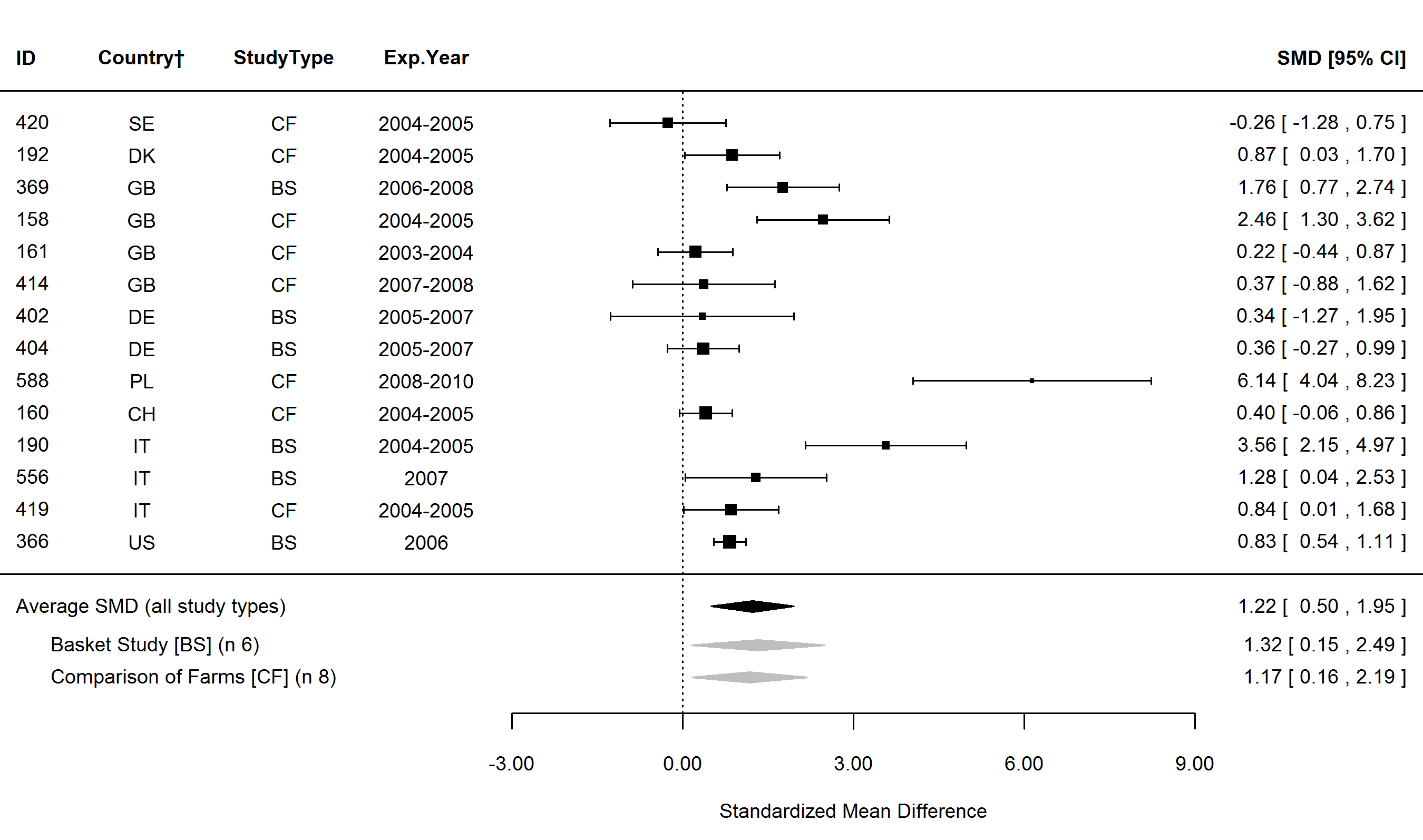


**Figure S13.** Forest plot showing the results of studies examining the *cis*-9-*trans*-11-18:2 conjugated linoleic fatty acids (CLA) in organic and conventional bovine milk. The figure shows the standardised mean differences (SMDs) with 95% confidence intervals, for studies included in standard meta-analysis. The estimated average SMD for all studies and SMDs for different study types are indicated at the bottom of the figure. Sign of the SMD indicates if the analysed parameter is higher (+) or lower (-) in organic milk. ID, Paper unique identification number (see supplementary Table S1 for references); CF, comparison of farms, BS, basket study, EX, controlled experiment. *No information about the experimental year (estimated as publication year -2), †Country codes according ISO 3166-2 (see [*http://www.iso.org/iso/home/standards/country_codes.htm*](http://www.iso.org/iso/home/standards/country_codes.htm)).


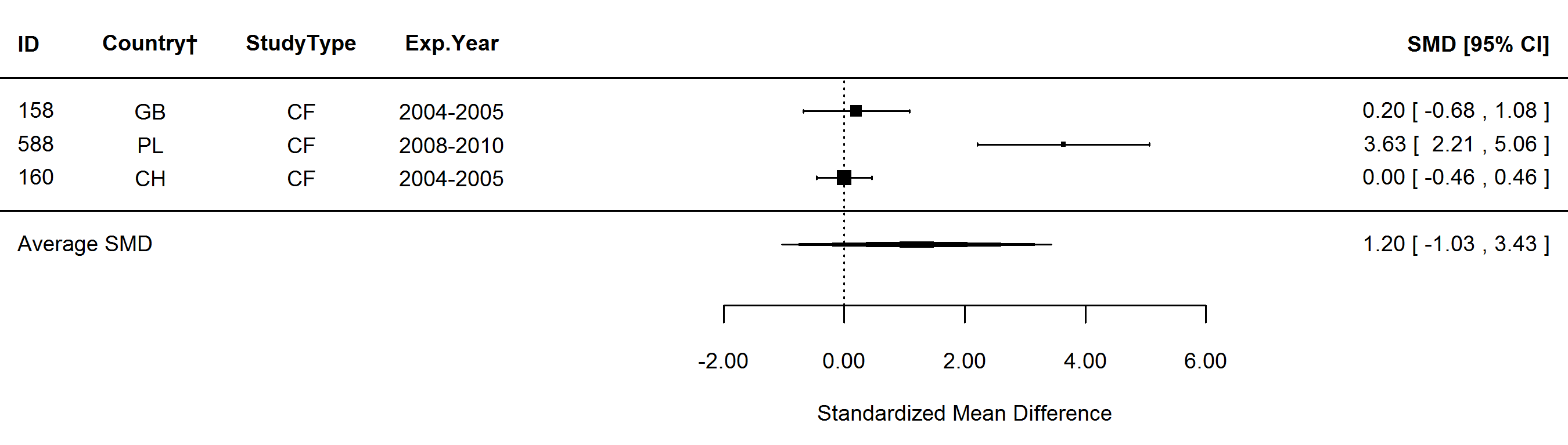


**Figure S14.** Forest plot showing the results of studies examining the *trans*-10-*cis*-12-18:2 conjugated linoleic fatty acids (CLA) in organic and conventional bovine milk. The figure shows the standardised mean differences (SMDs) with 95% confidence intervals, for studies included in standard meta-analysis. The estimated average SMD for all studies and SMDs for different study types are indicated at the bottom of the figure. Sign of the SMD indicates if the analysed parameter is higher (+) or lower (-) in organic milk. ID, Paper unique identification number (see supplementary Table S1 for references); CF, comparison of farms, BS, basket study, EX, controlled experiment. *No information about the experimental year (estimated as publication year -2), †Country codes according ISO 3166-2 (see [*http://www.iso.org/iso/home/standards/country_codes.htm*](http://www.iso.org/iso/home/standards/country_codes.htm)).


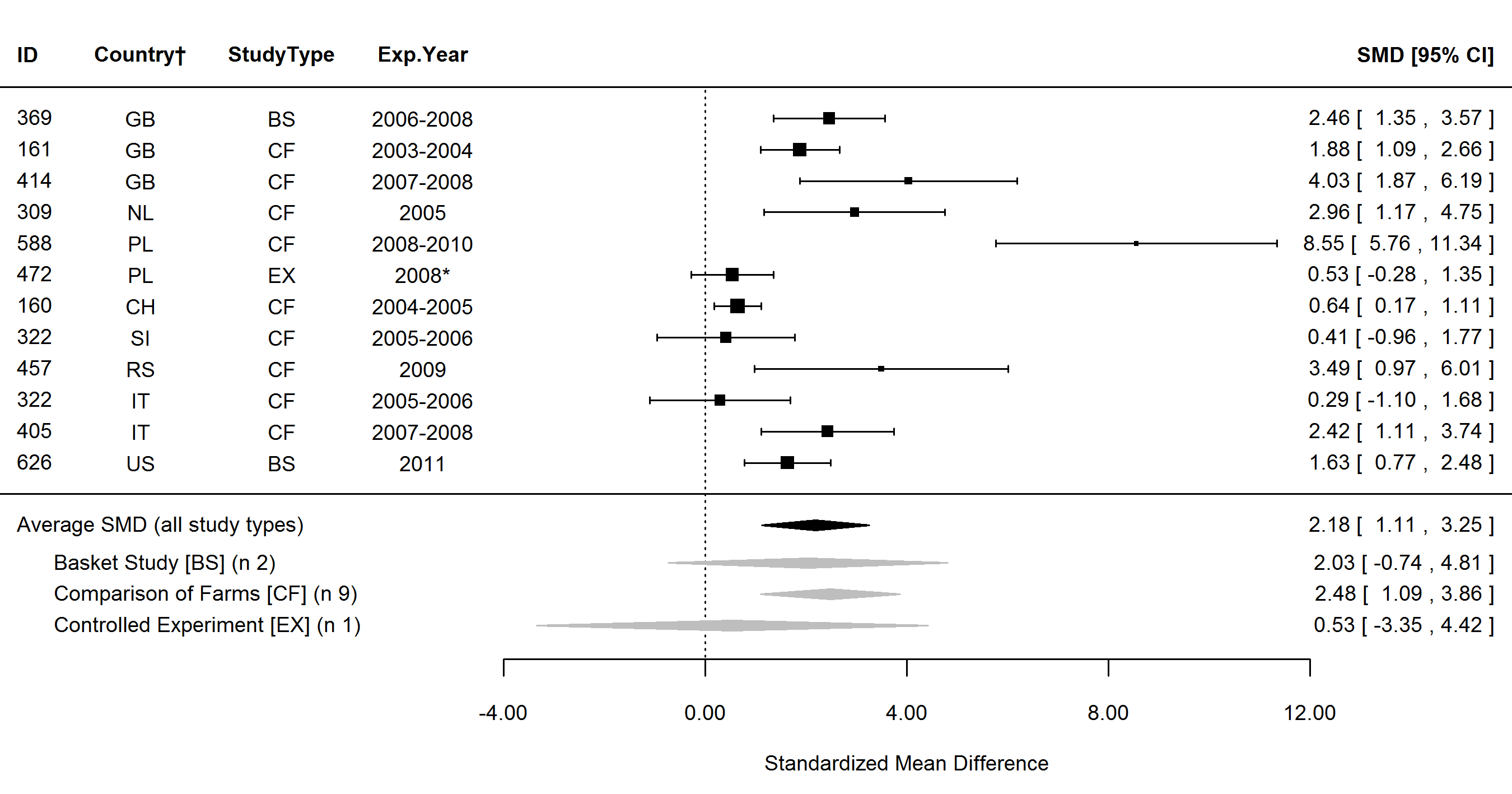


**Figure S15.** Forest plot showing the results of studies examining the omega-3 fatty acids (*n*-3) in organic and conventional bovine milk. The figure shows the standardised mean differences (SMDs) with 95% confidence intervals, for studies included in standard meta-analysis. The estimated average SMD for all studies and SMDs for different study types are indicated at the bottom of the figure. Sign of the SMD indicates if the analysed parameter is higher (+) or lower (-) in organic milk. ID, Paper unique identification number (see supplementary Table S1 for references); CF, comparison of farms, BS, basket study, EX, controlled experiment. *No information about the experimental year (estimated as publication year -2), †Country codes according ISO 3166-2 (see [*http://www.iso.org/iso/home/standards/country_codes.htm*](http://www.iso.org/iso/home/standards/country_codes.htm)).


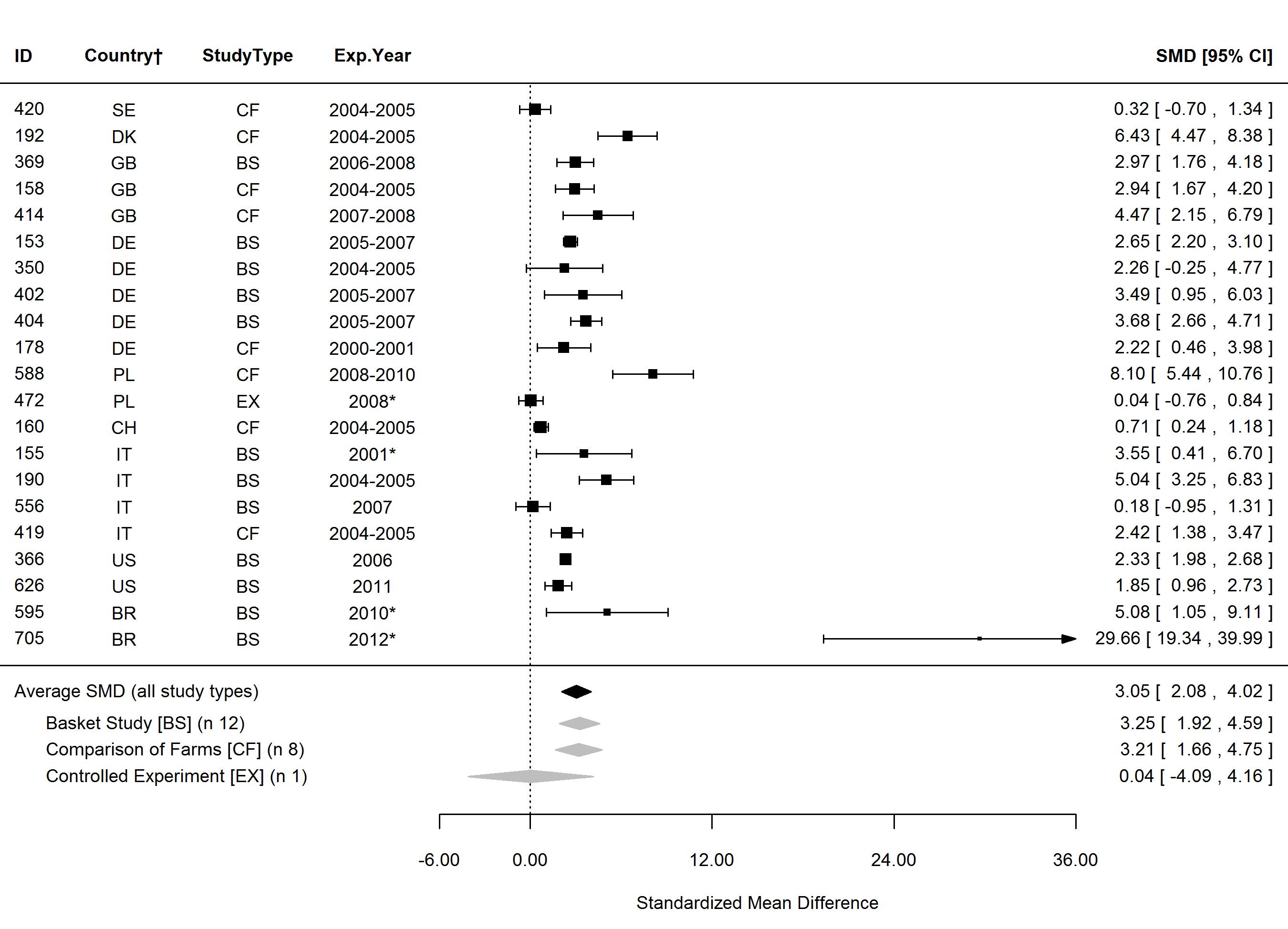


**Figure S16.** Forest plot showing the results of studies examining the α-linolenic fatty acid (*cis*-9,12,15-18:3, ALA) in organic and conventional bovine milk. The figure shows the standardised mean differences (SMDs) with 95% confidence intervals, for studies included in standard meta-analysis. The estimated average SMD for all studies and SMDs for different study types are indicated at the bottom of the figure. Sign of the SMD indicates if the analysed parameter is higher (+) or lower (-) in organic milk. ID, Paper unique identification number (see supplementary Table S1 for references); CF, comparison of farms, BS, basket study, EX, controlled experiment. *No information about the experimental year (estimated as publication year -2), †Country codes according ISO 3166-2 (see [*http://www.iso.org/iso/home/standards/country_codes.htm*](http://www.iso.org/iso/home/standards/country_codes.htm)).


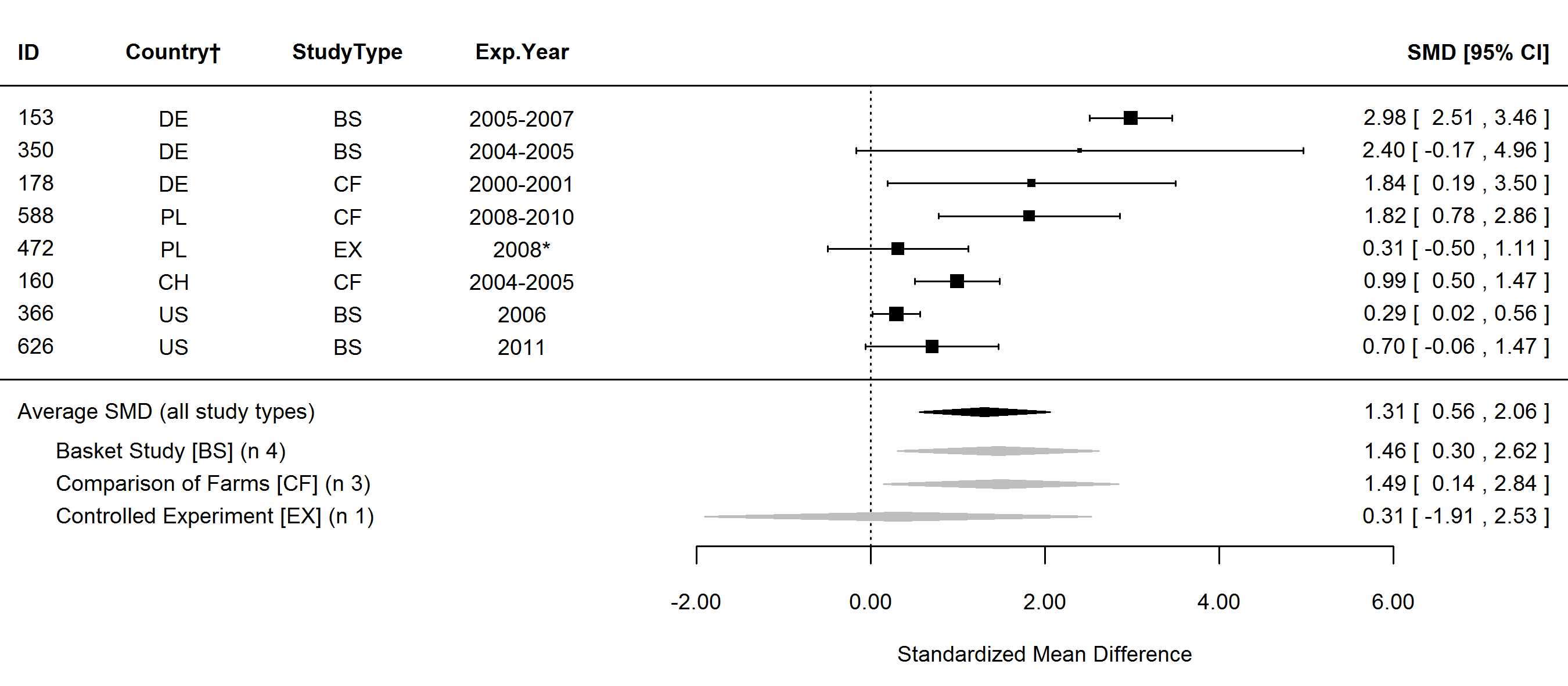


**Figure S17.** Forest plot showing the results of studies examining the eicosapentaenoic fatty acid (*cis*-5,8,11,14,17-20:5, EPA) in organic and conventional bovine milk. The figure shows the standardised mean differences (SMDs) with 95% confidence intervals, for studies included in standard meta-analysis. The estimated average SMD for all studies and SMDs for different study types are indicated at the bottom of the figure. Sign of the SMD indicates if the analysed parameter is higher (+) or lower (-) in organic milk. ID, Paper unique identification number (see supplementary Table S1 for references); CF, comparison of farms, BS, basket study, EX, controlled experiment. *No information about the experimental year (estimated as publication year -2), †Country codes according ISO 3166-2 (see [*http://www.iso.org/iso/home/standards/country_codes.htm*](http://www.iso.org/iso/home/standards/country_codes.htm)).


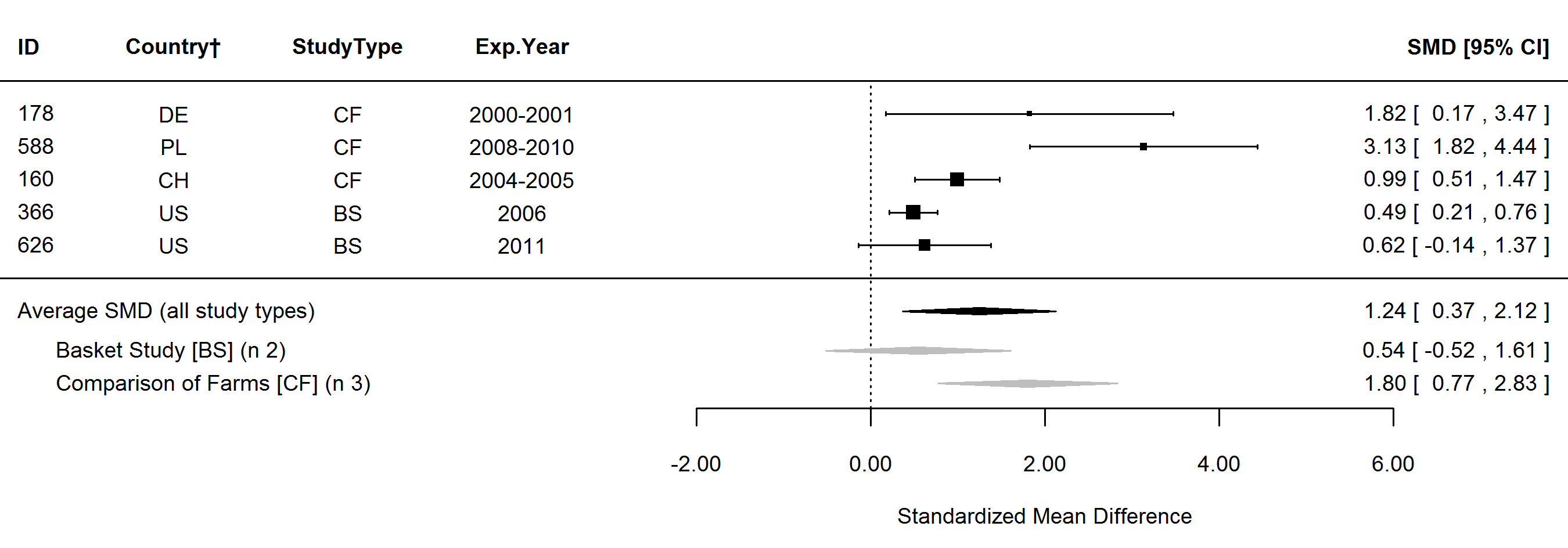


**Figure S18.** Forest plot showing the results of studies examining the docosapentaenoic fatty acid (*cis*-7,10,13,16,19-22:5, DPA) in organic and conventional bovine milk. The figure shows the standardised mean differences (SMDs) with 95% confidence intervals, for studies included in standard meta-analysis. The estimated average SMD for all studies and SMDs for different study types are indicated at the bottom of the figure. Sign of the SMD indicates if the analysed parameter is higher (+) or lower (-) in organic milk. ID, Paper unique identification number (see supplementary Table S1 for references); CF, comparison of farms, BS, basket study, EX, controlled experiment. *No information about the experimental year (estimated as publication year -2), †Country codes according ISO 3166-2 (see [*http://www.iso.org/iso/home/standards/country_codes.htm*](http://www.iso.org/iso/home/standards/country_codes.htm)).


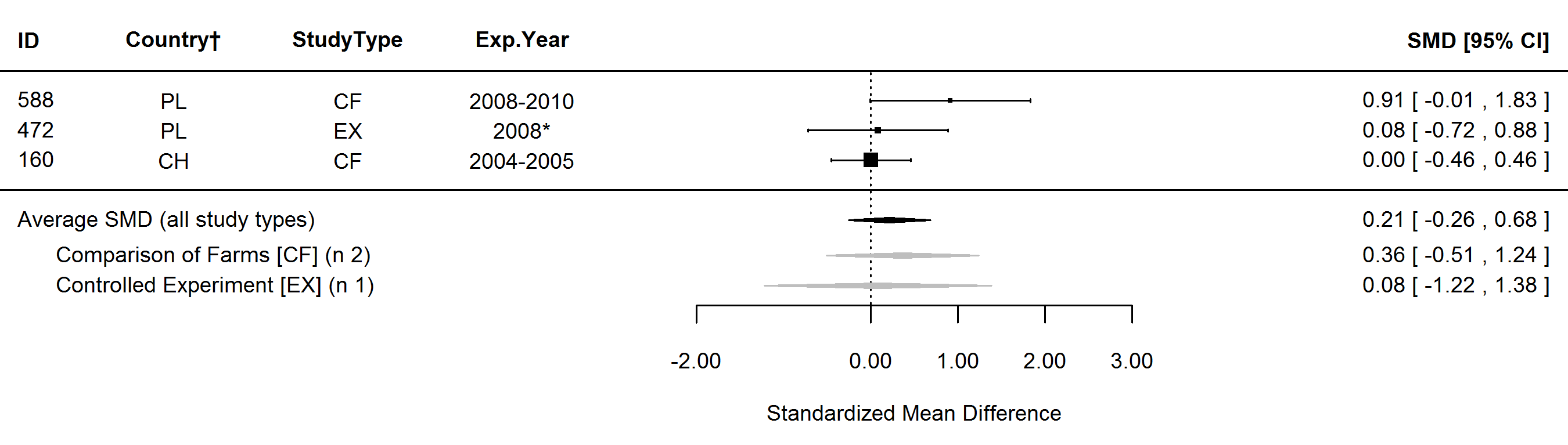


**Figure S19.** Forest plot showing the results of studies examining the docosahexaenoic fatty acid (*cis*-4,7,10,13,16,19-22:6, DHA) in organic and conventional bovine milk. The figure shows the standardised mean differences (SMDs) with 95% confidence intervals, for studies included in standard meta-analysis. The estimated average SMD for all studies and SMDs for different study types are indicated at the bottom of the figure. Sign of the SMD indicates if the analysed parameter is higher (+) or lower (-) in organic milk. ID, Paper unique identification number (see supplementary Table S1 for references); CF, comparison of farms, BS, basket study, EX, controlled experiment. *No information about the experimental year (estimated as publication year -2), †Country codes according ISO 3166-2 (see [*http://www.iso.org/iso/home/standards/country_codes.htm*](http://www.iso.org/iso/home/standards/country_codes.htm)).


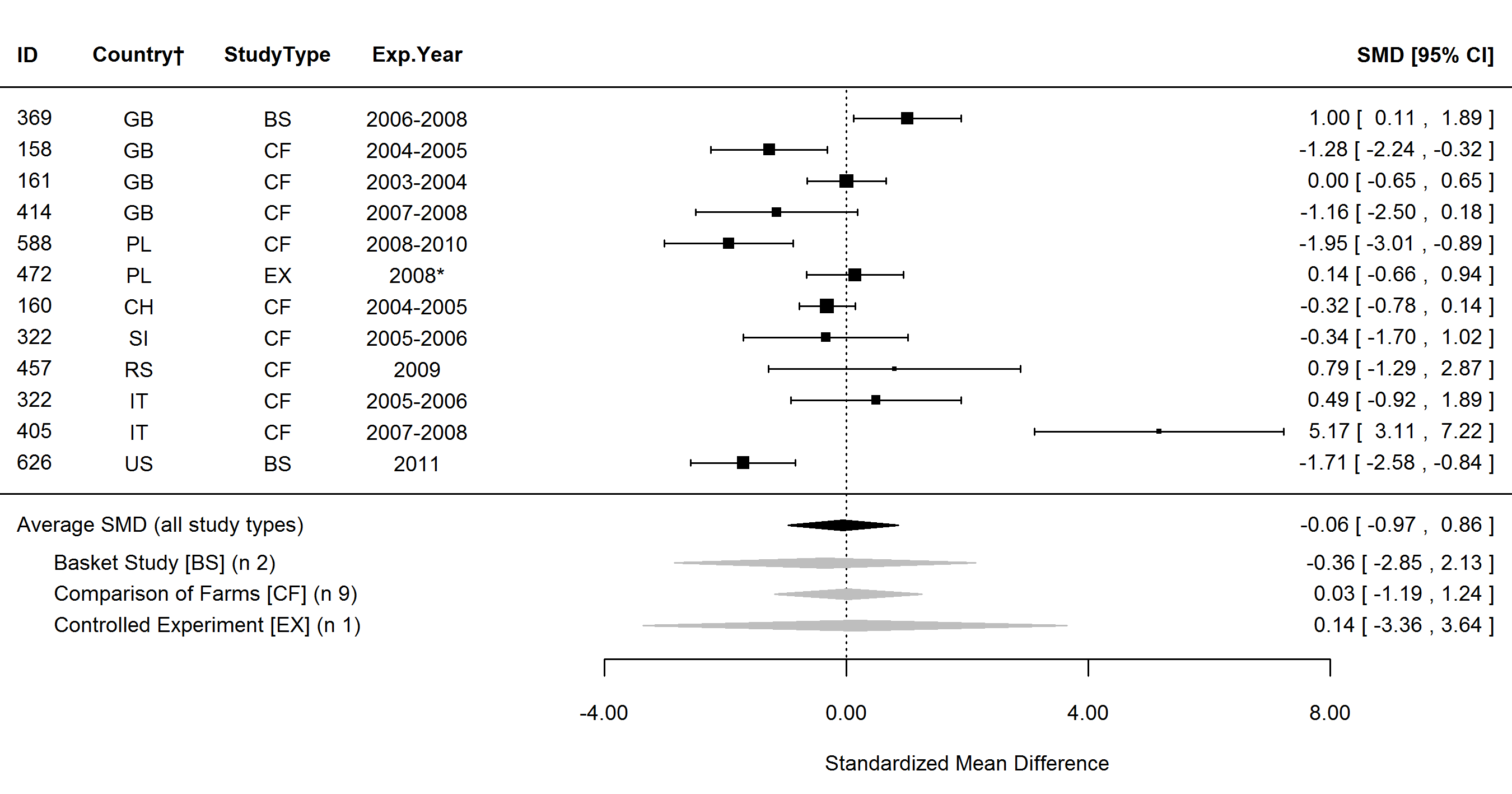


**Figure S20.** Forest plot showing the results of studies examining the omega-6 fatty acids (*n*-6) in organic and conventional bovine milk. The figure shows the standardised mean differences (SMDs) with 95% confidence intervals, for studies included in standard meta-analysis. The estimated average SMD for all studies and SMDs for different study types are indicated at the bottom of the figure. Sign of the SMD indicates if the analysed parameter is higher (+) or lower (-) in organic milk. ID, Paper unique identification number (see supplementary Table S1 for references); CF, comparison of farms, BS, basket study, EX, controlled experiment. *No information about the experimental year (estimated as publication year -2), †Country codes according ISO 3166-2 (see [*http://www.iso.org/iso/home/standards/country_codes.htm*](http://www.iso.org/iso/home/standards/country_codes.htm)).


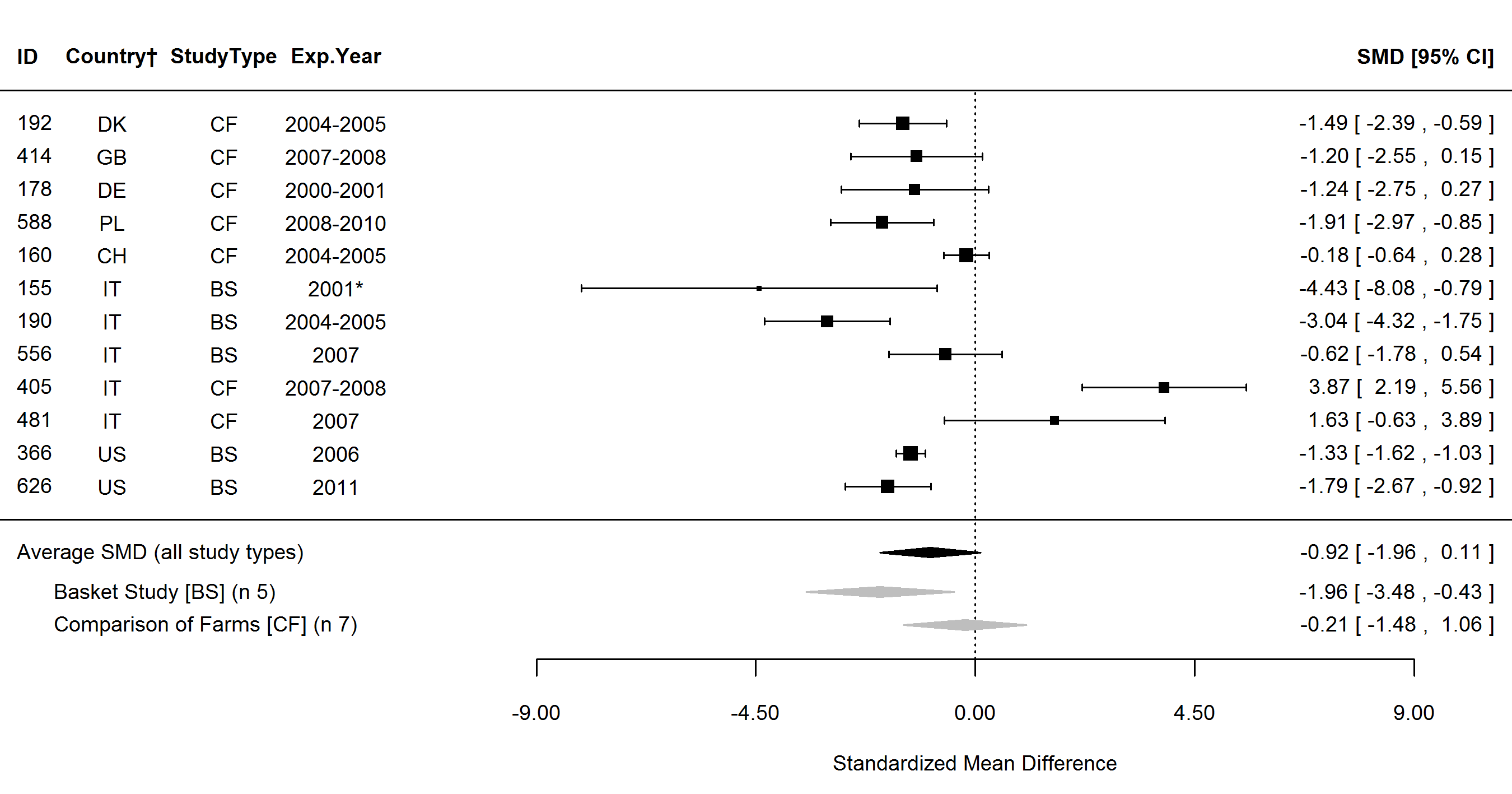


**Figure S21.** Forest plot showing the results of studies examining the linoleic fatty acid (*cis*-9,12-18:2, LA) in organic and conventional bovine milk. The figure shows the standardised mean differences (SMDs) with 95% confidence intervals, for studies included in standard meta-analysis. The estimated average SMD for all studies and SMDs for different study types are indicated at the bottom of the figure. Sign of the SMD indicates if the analysed parameter is higher (+) or lower (-) in organic milk. ID, Paper unique identification number (see supplementary Table S1 for references); CF, comparison of farms, BS, basket study, EX, controlled experiment. *No information about the experimental year (estimated as publication year -2), †Country codes according ISO 3166-2 (see [*http://www.iso.org/iso/home/standards/country_codes.htm*](http://www.iso.org/iso/home/standards/country_codes.htm)).


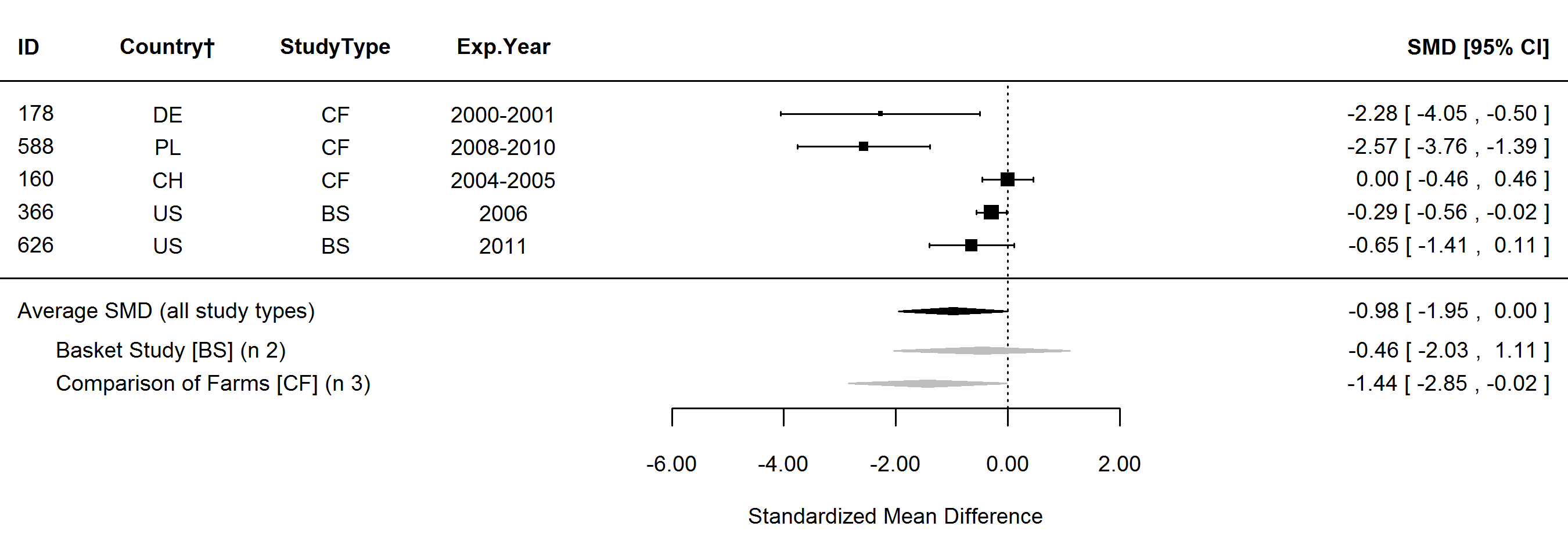


**Figure S22.** Forest plot showing the results of studies examining the arachidonic fatty acid (*cis*-5,8,11,14-20:4, AA) in organic and conventional bovine milk. The figure shows the standardised mean differences (SMDs) with 95% confidence intervals, for studies included in standard meta-analysis. The estimated average SMD for all studies and SMDs for different study types are indicated at the bottom of the figure. Sign of the SMD indicates if the analysed parameter is higher (+) or lower (-) in organic milk. ID, Paper unique identification number (see supplementary Table S1 for references); CF, comparison of farms, BS, basket study, EX, controlled experiment. *No information about the experimental year (estimated as publication year -2), †Country codes according ISO 3166-2 (see [*http://www.iso.org/iso/home/standards/country_codes.htm*](http://www.iso.org/iso/home/standards/country_codes.htm)).


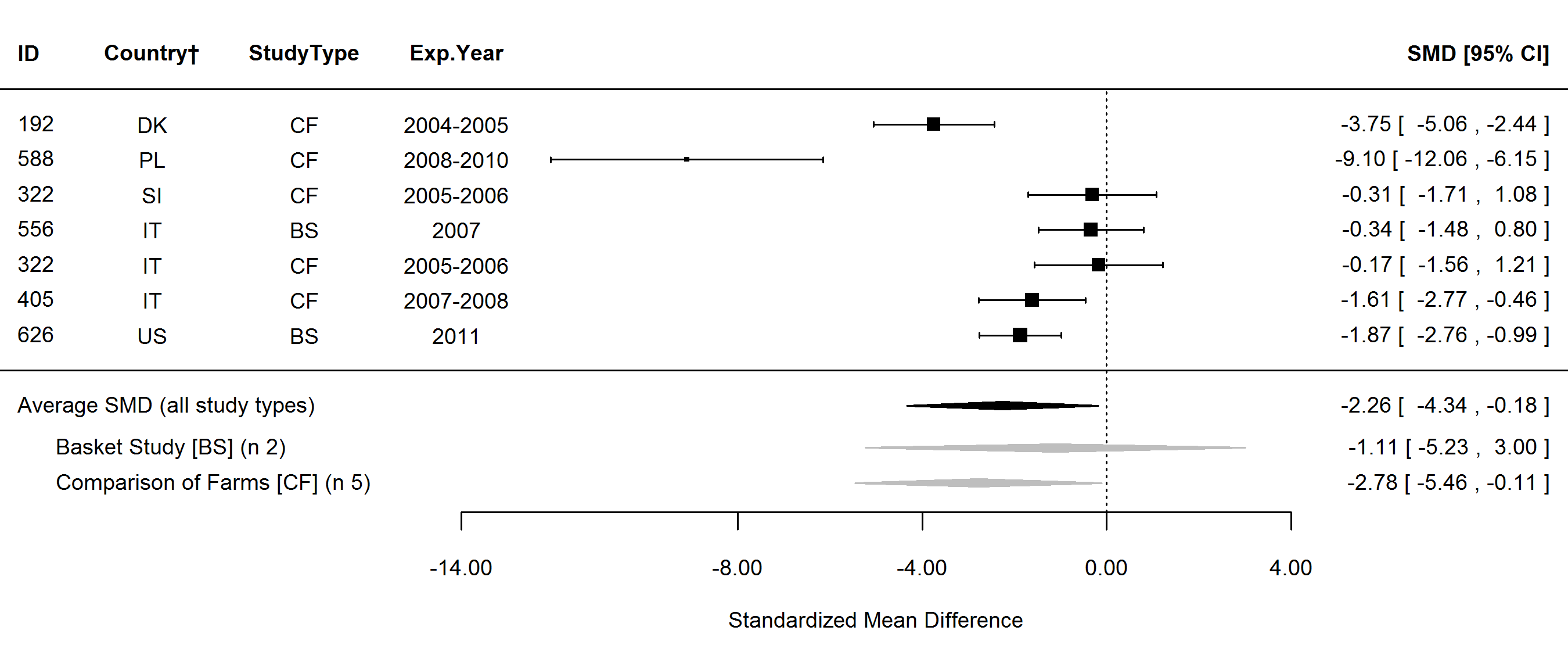


**Figure S23.** Forest plot showing the results of studies examining the omega-6/omega-3 fatty acids ratio (*n*-6/*n*-3) in organic and conventional bovine milk. The figure shows the standardised mean differences (SMDs) with 95% confidence intervals, for studies included in standard meta-analysis. The estimated average SMD for all studies and SMDs for different study types are indicated at the bottom of the figure. Sign of the SMD indicates if the analysed parameter is higher (+) or lower (-) in organic milk. ID, Paper unique identification number (see supplementary Table S1 for references); CF, comparison of farms, BS, basket study, EX, controlled experiment. *No information about the experimental year (estimated as publication year -2), †Country codes according ISO 3166-2 (see [*http://www.iso.org/iso/home/standards/country_codes.htm*](http://www.iso.org/iso/home/standards/country_codes.htm)).


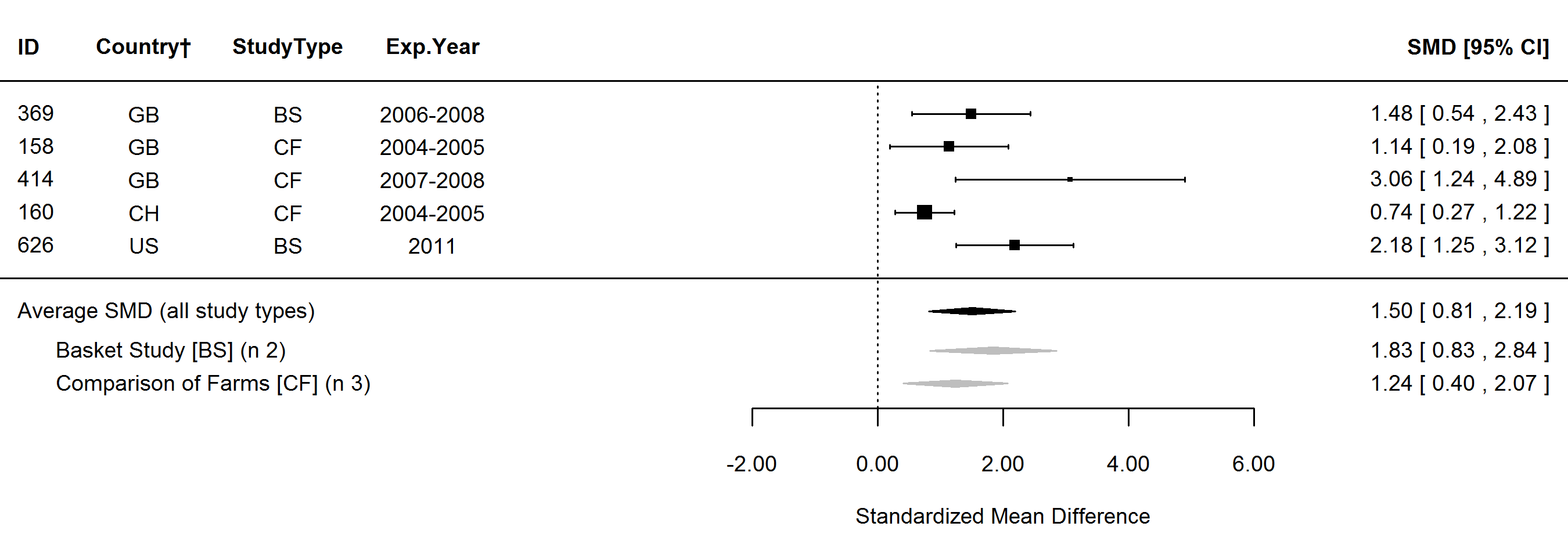


**Figure S24.** Forest plot showing the results of studies examining the omega-3/omega-6 fatty acids ratio (*n*-3/*n*-6) in organic and conventional bovine milk. The figure shows the standardised mean differences (SMDs) with 95% confidence intervals, for studies included in standard meta-analysis. The estimated average SMD for all studies and SMDs for different study types are indicated at the bottom of the figure. Sign of the SMD indicates if the analysed parameter is higher (+) or lower (-) in organic milk. ID, Paper unique identification number (see supplementary Table S1 for references); CF, comparison of farms, BS, basket study, EX, controlled experiment. *No information about the experimental year (estimated as publication year -2), †Country codes according ISO 3166-2 (see [*http://www.iso.org/iso/home/standards/country_codes.htm*](http://www.iso.org/iso/home/standards/country_codes.htm)).


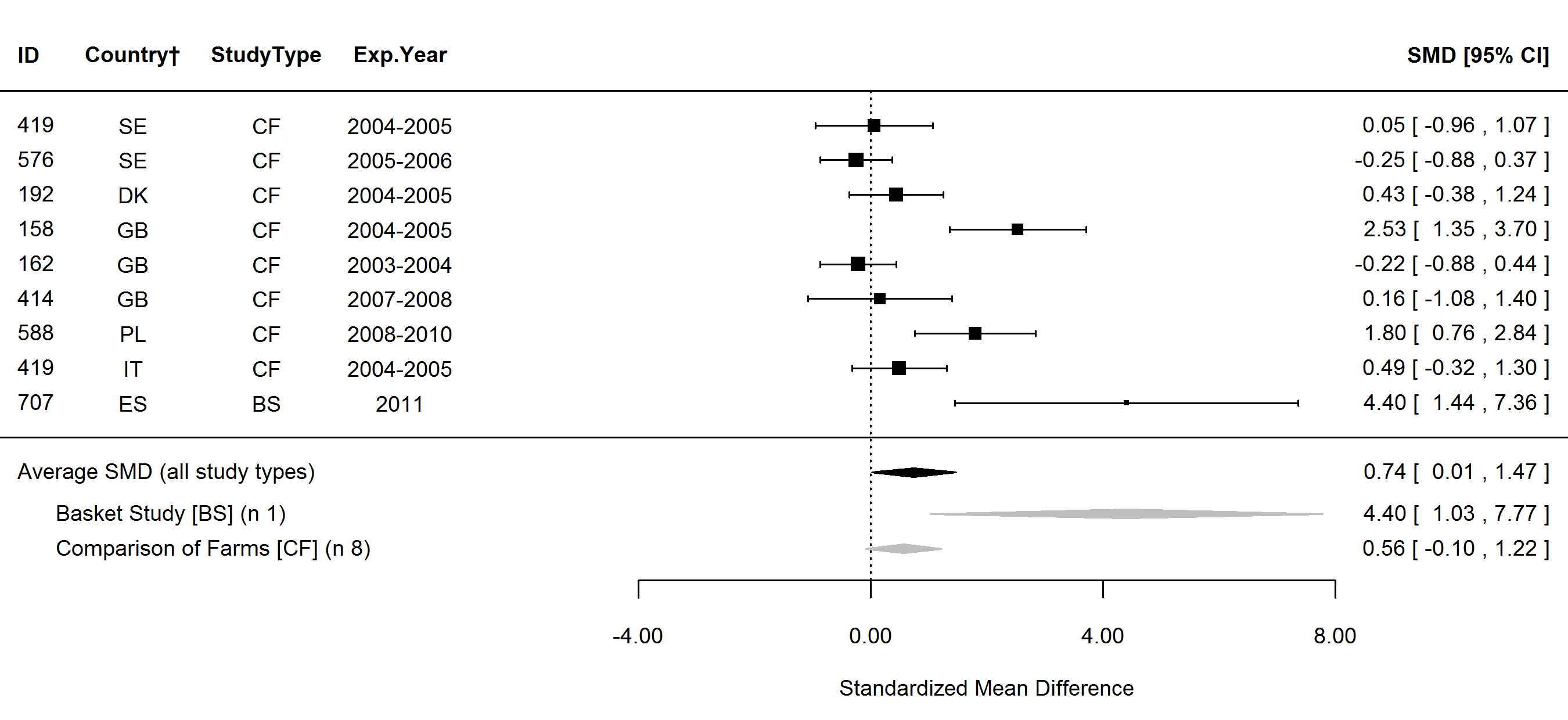


**Figure S25.** Forest plot showing the results of studies examining the α-tocopherol in organic and conventional bovine milk. The figure shows the standardised mean differences (SMDs) with 95% confidence intervals, for studies included in standard meta-analysis. The estimated average SMD for all studies and SMDs for different study types are indicated at the bottom of the figure. Sign of the SMD indicates if the analysed parameter is higher (+) or lower (-) in organic milk. ID, Paper unique identification number (see supplementary Table S1 for references); CF, comparison of farms, BS, basket study, EX, controlled experiment. *No information about the experimental year (estimated as publication year -2), †Country codes according ISO 3166-2 (see [*http://www.iso.org/iso/home/standards/country_codes.htm*](http://www.iso.org/iso/home/standards/country_codes.htm)).


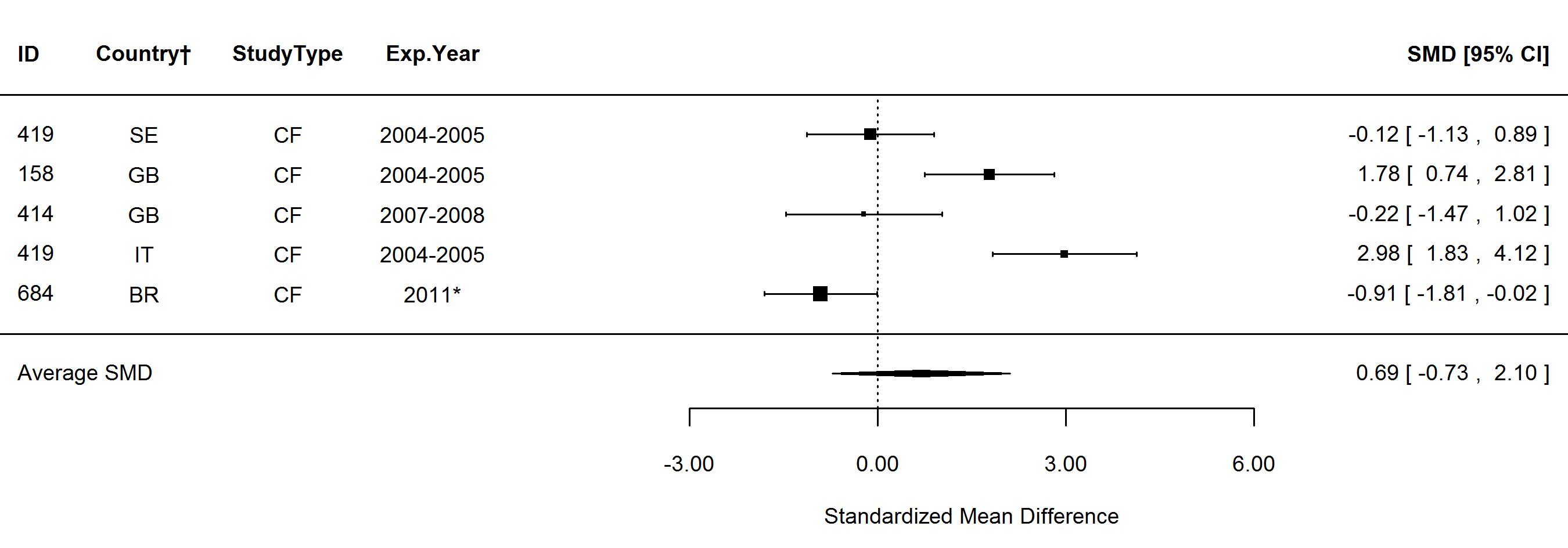


**Figure S26.** Forest plot showing the results of studies examining the total carotenoids in organic and conventional bovine milk. The figure shows the standardised mean differences (SMDs) with 95% confidence intervals, for studies included in standard meta-analysis. The estimated average SMD for all studies and SMDs for different study types are indicated at the bottom of the figure. Sign of the SMD indicates if the analysed parameter is higher (+) or lower (-) in organic milk. ID, Paper unique identification number (see supplementary Table S1 for references); CF, comparison of farms, BS, basket study, EX, controlled experiment. *No information about the experimental year (estimated as publication year -2), †Country codes according ISO 3166-2 (see [*http://www.iso.org/iso/home/standards/country_codes.htm*](http://www.iso.org/iso/home/standards/country_codes.htm)).


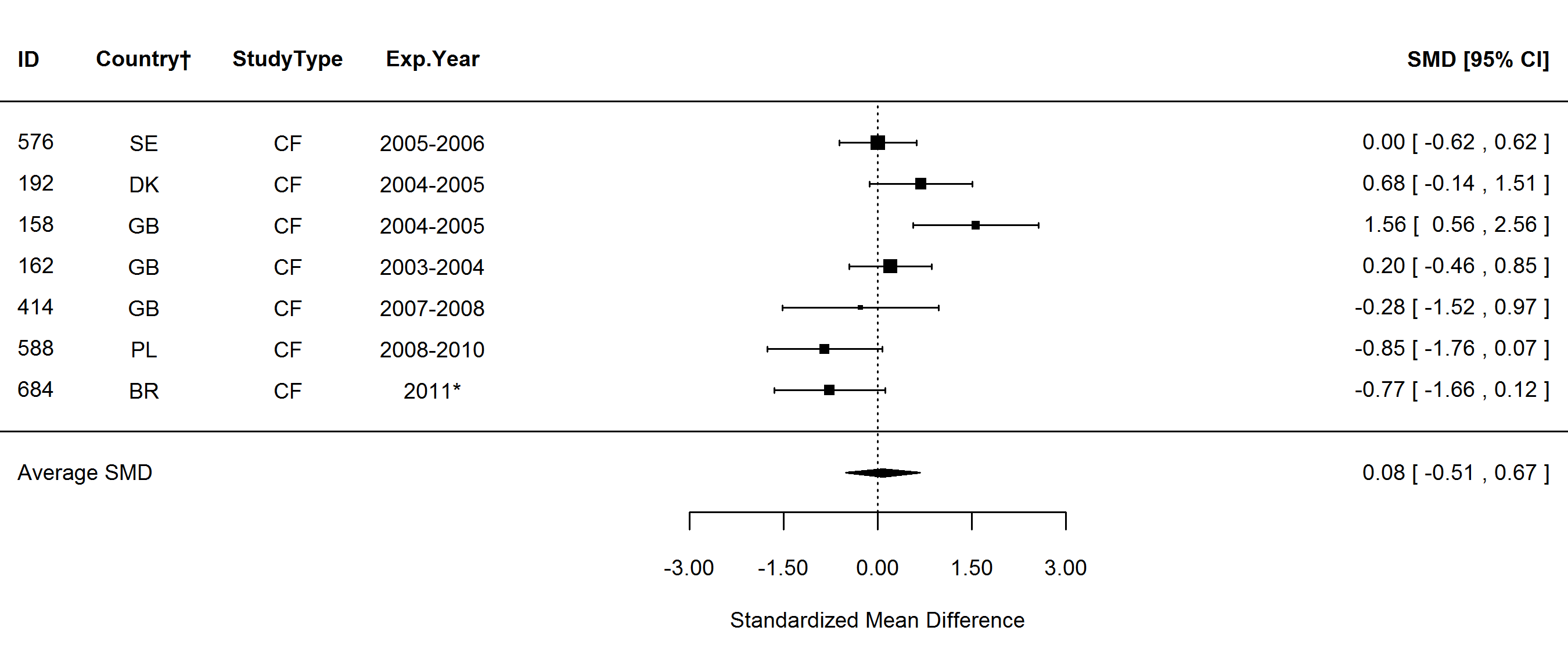


**Figure S27.** Forest plot showing the results of studies examining the β-carotene in organic and conventional bovine milk. The figure shows the standardised mean differences (SMDs) with 95% confidence intervals, for studies included in standard meta-analysis. The estimated average SMD for all studies and SMDs for different study types are indicated at the bottom of the figure. Sign of the SMD indicates if the analysed parameter is higher (+) or lower (-) in organic milk. ID, Paper unique identification number (see supplementary Table S1 for references); CF, comparison of farms, BS, basket study, EX, controlled experiment. *No information about the experimental year (estimated as publication year -2), †Country codes according ISO 3166-2 (see [*http://www.iso.org/iso/home/standards/country_codes.htm*](http://www.iso.org/iso/home/standards/country_codes.htm)).


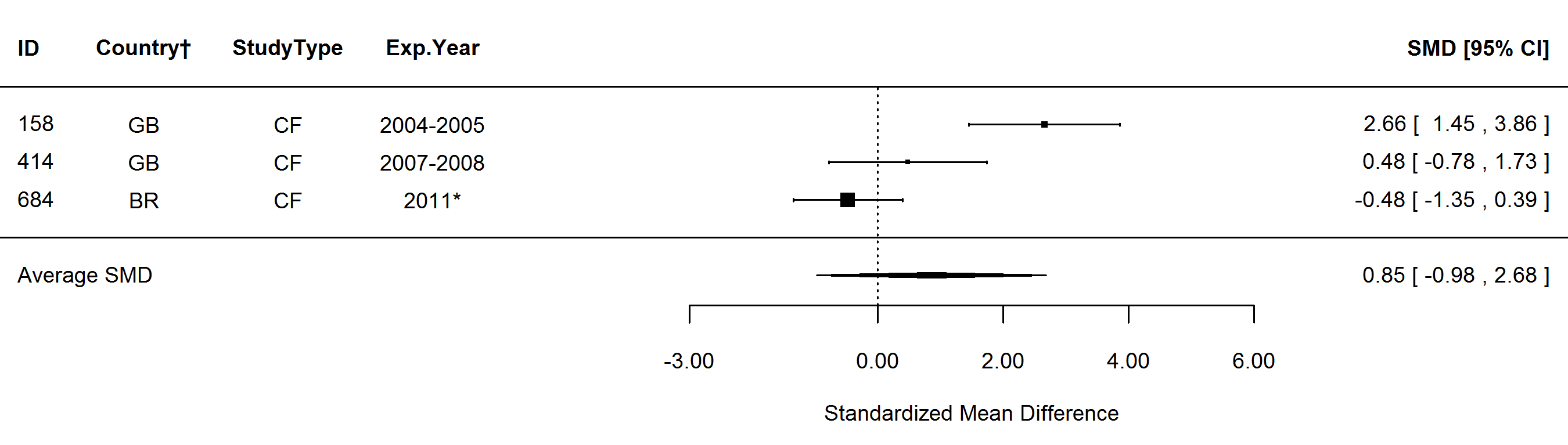


**Figure S28.** Forest plot showing the results of studies examining the lutein in organic and conventional bovine milk. The figure shows the standardised mean differences (SMDs) with 95% confidence intervals, for studies included in standard meta-analysis. The estimated average SMD for all studies and SMDs for different study types are indicated at the bottom of the figure. Sign of the SMD indicates if the analysed parameter is higher (+) or lower (-) in organic milk. ID, Paper unique identification number (see supplementary Table S1 for references); CF, comparison of farms, BS, basket study, EX, controlled experiment. *No information about the experimental year (estimated as publication year -2), †Country codes according ISO 3166-2 (see [*http://www.iso.org/iso/home/standards/country_codes.htm*](http://www.iso.org/iso/home/standards/country_codes.htm)).


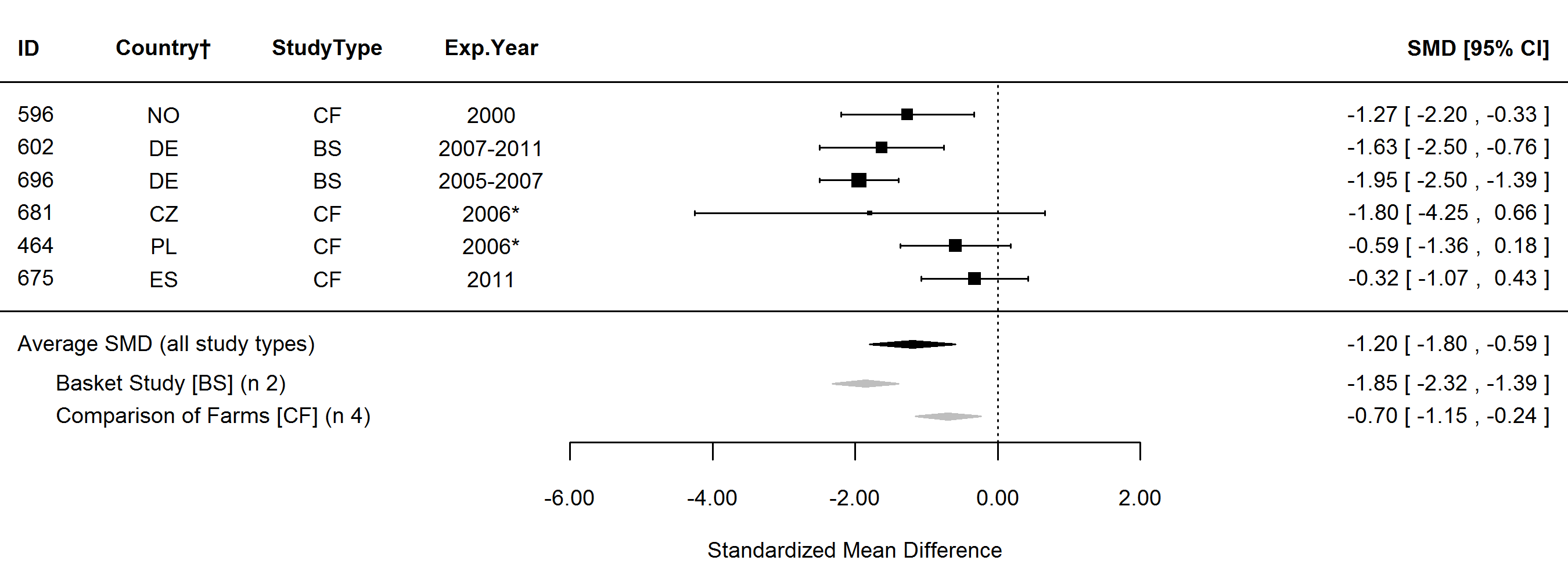


**Figure S29.** Forest plot showing the results of studies examining the iodine (I) in organic and conventional bovine milk. The figure shows the standardised mean differences (SMDs) with 95% confidence intervals, for studies included in standard meta-analysis. The estimated average SMD for all studies and SMDs for different study types are indicated at the bottom of the figure. Sign of the SMD indicates if the analysed parameter is higher (+) or lower (-) in organic milk. ID, Paper unique identification number (see supplementary Table S1 for references); CF, comparison of farms, BS, basket study, EX, controlled experiment. *No information about the experimental year (estimated as publication year -2), †Country codes according ISO 3166-2 (see [*http://www.iso.org/iso/home/standards/country_codes.htm*](http://www.iso.org/iso/home/standards/country_codes.htm)).


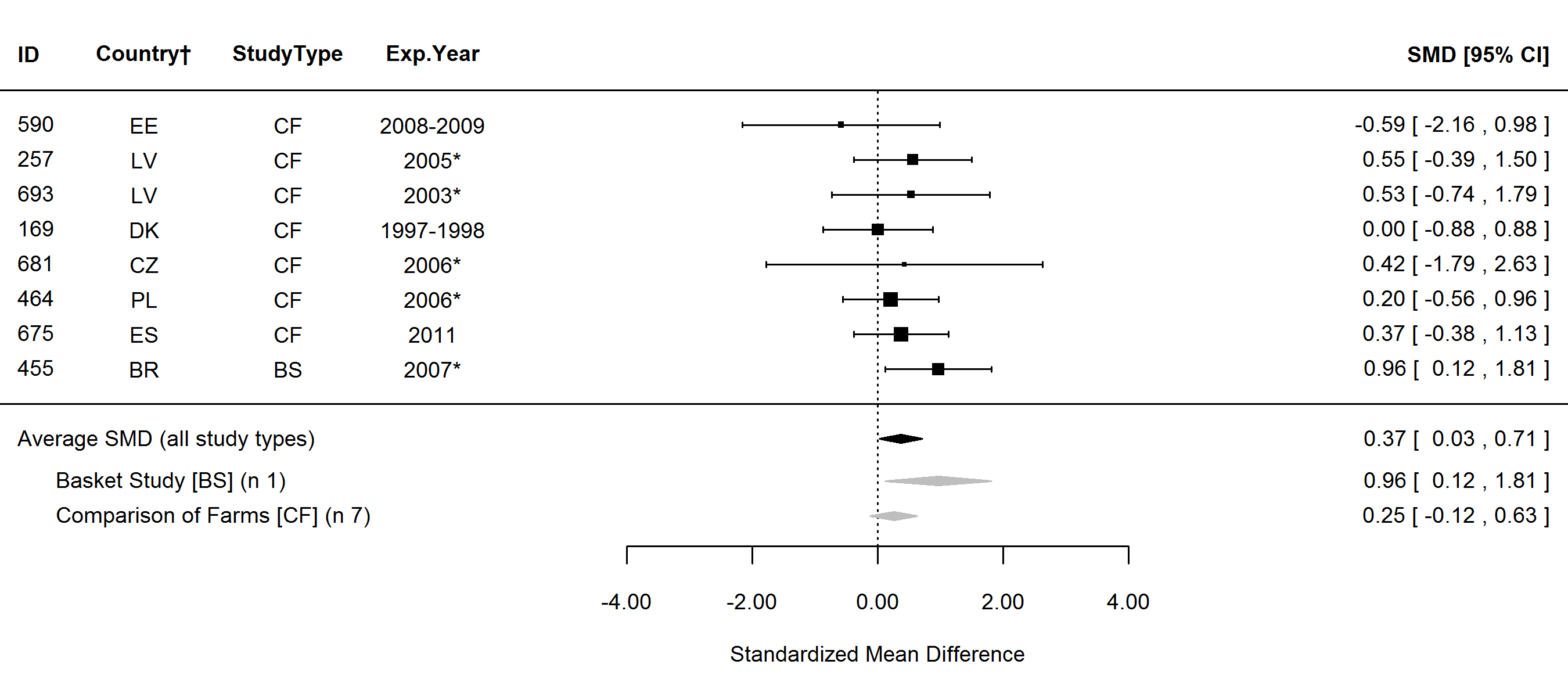


**Figure S30.** Forest plot showing the results of studies examining the iron (Fe) in organic and conventional bovine milk. The figure shows the standardised mean differences (SMDs) with 95% confidence intervals, for studies included in standard meta-analysis. The estimated average SMD for all studies and SMDs for different study types are indicated at the bottom of the figure. Sign of the SMD indicates if the analysed parameter is higher (+) or lower (-) in organic milk. ID, Paper unique identification number (see supplementary Table S1 for references); CF, comparison of farms, BS, basket study, EX, controlled experiment. *No information about the experimental year (estimated as publication year -2), †Country codes according ISO 3166-2 (see [*http://www.iso.org/iso/home/standards/country_codes.htm*](http://www.iso.org/iso/home/standards/country_codes.htm)).


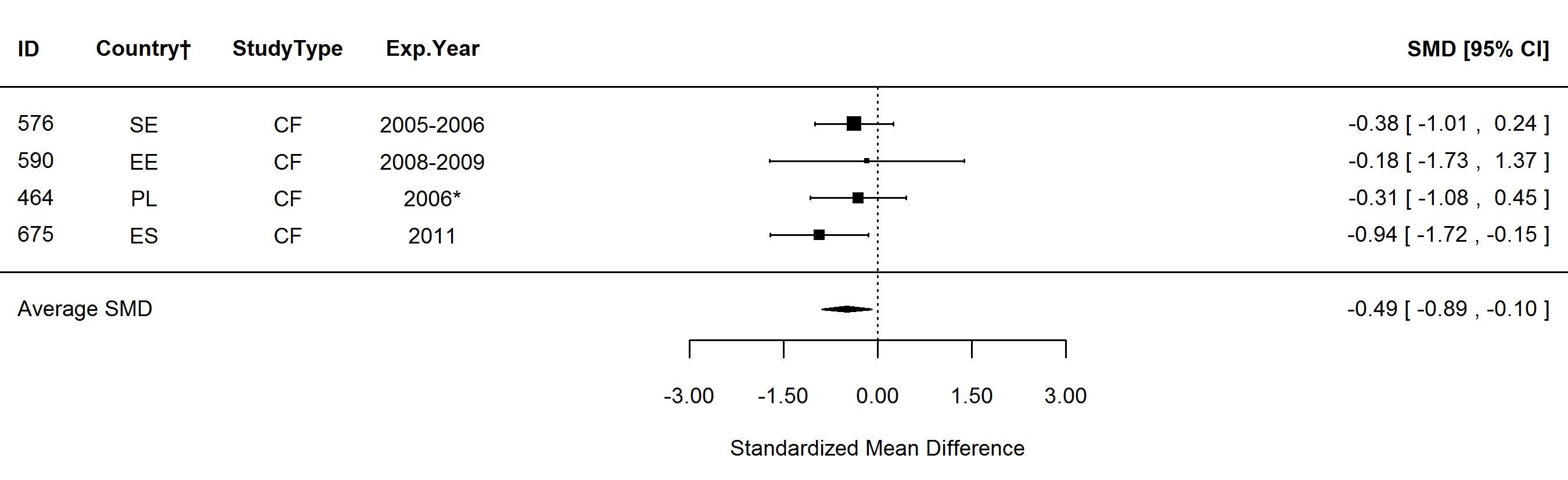


**Figure S31.** Forest plot showing the results of studies examining the selenium (Se) in organic and conventional bovine milk. The figure shows the standardised mean differences (SMDs) with 95% confidence intervals, for studies included in standard meta-analysis. The estimated average SMD for all studies and SMDs for different study types are indicated at the bottom of the figure. Sign of the SMD indicates if the analysed parameter is higher (+) or lower (-) in organic milk. ID, Paper unique identification number (see supplementary Table S1 for references); CF, comparison of farms, BS, basket study, EX, controlled experiment. *No information about the experimental year (estimated as publication year -2), †Country codes according ISO 3166-2 (see [*http://www.iso.org/iso/home/standards/country_codes.htm*](http://www.iso.org/iso/home/standards/country_codes.htm)).


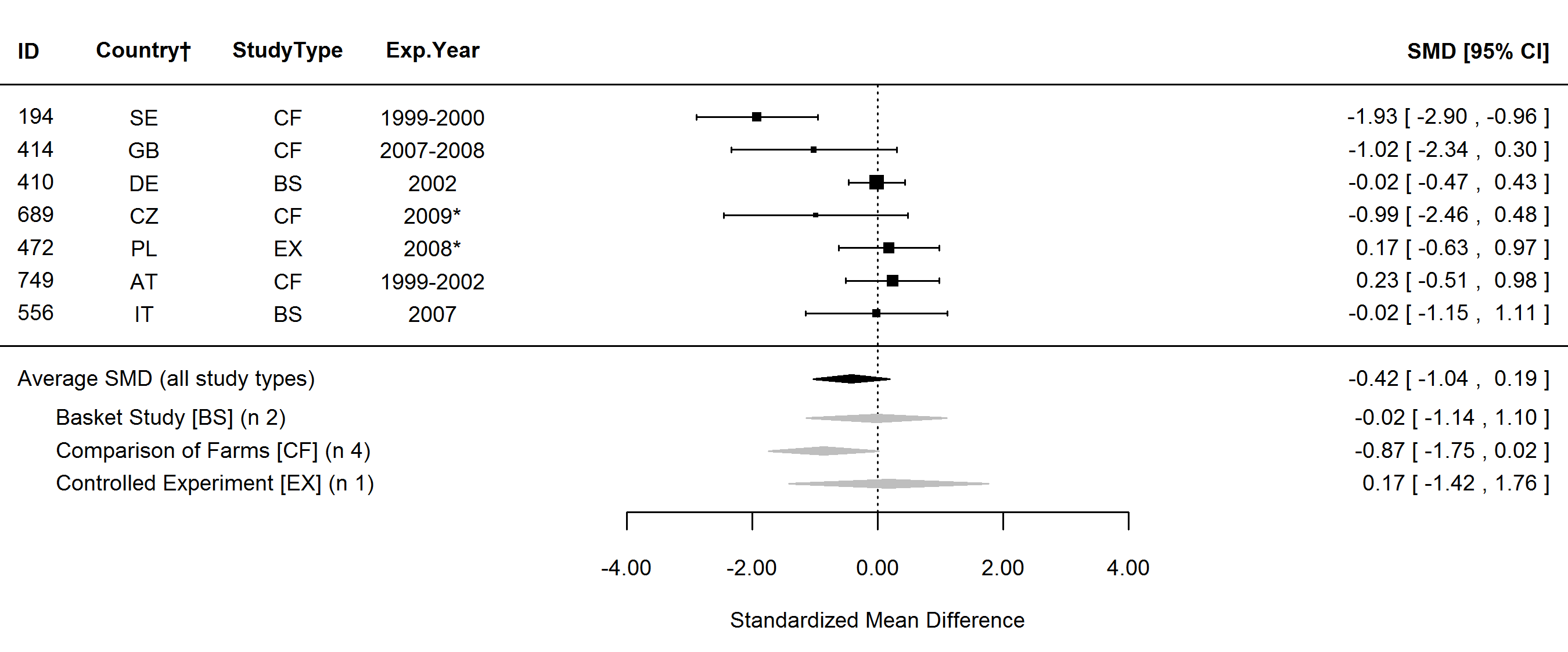


**Figure S32.** Forest plot showing the results of studies examining the urea in organic and conventional bovine milk. The figure shows the standardised mean differences (SMDs) with 95% confidence intervals, for studies included in standard meta-analysis. The estimated average SMD for all studies and SMDs for different study types are indicated at the bottom of the figure. Sign of the SMD indicates if the analysed parameter is higher (+) or lower (-) in organic milk. ID, Paper unique identification number (see supplementary Table S1 for references); CF, comparison of farms, BS, basket study, EX, controlled experiment. *No information about the experimental year (estimated as publication year -2), †Country codes according ISO 3166-2 (see [*http://www.iso.org/iso/home/standards/country_codes.htm*](http://www.iso.org/iso/home/standards/country_codes.htm)).


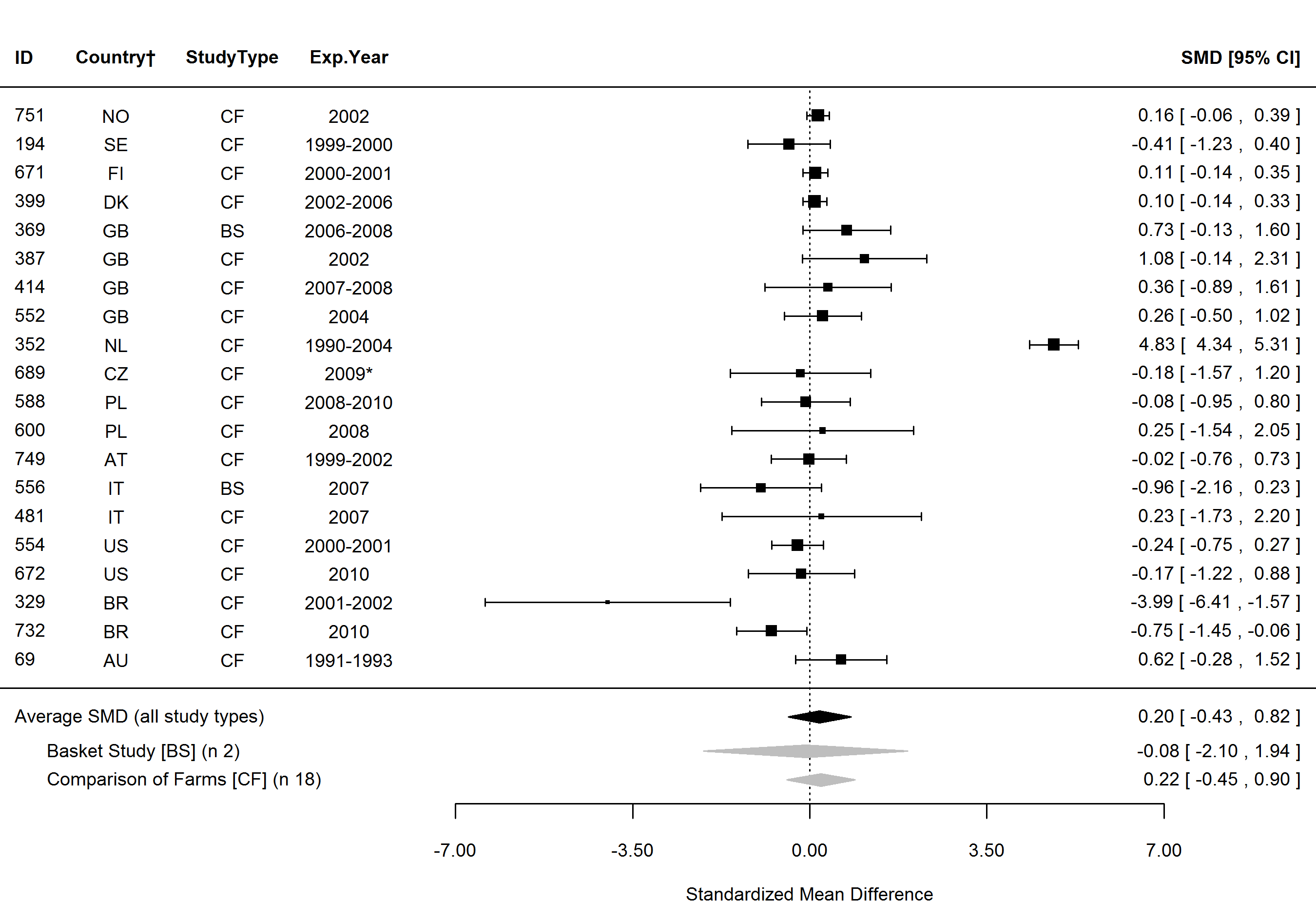


**Figure S33.** Forest plot showing the results of studies examining the somatic cell count (SCC) in organic and conventional bovine milk. The figure shows the standardised mean differences (SMDs) with 95% confidence intervals, for studies included in standard meta-analysis. The estimated average SMD for all studies and SMDs for different study types are indicated at the bottom of the figure. Sign of the SMD indicates if the analysed parameter is higher (+) or lower (-) in organic milk. ID, Paper unique identification number (see supplementary Table S1 for references); CF, comparison of farms, BS, basket study, EX, controlled experiment. *No information about the experimental year (estimated as publication year -2), †Country codes according ISO 3166-2 (see [*http://www.iso.org/iso/home/standards/country_codes.htm*](http://www.iso.org/iso/home/standards/country_codes.htm)).

| **Table S12.** Results of the standard meta-analysis and sensitivity analysis 1 for parameters where none of the protocols identified significant effects. | | | | | | | | | |
| --- | --- | --- | --- | --- | --- | --- | --- | --- | --- |
|  | **Standard meta-analysis** | | | | |  | **Sensitivity analysis 1** | | |
| **Parameter** | ***n*** | **SMD** | **95% CI** | ***P**** | **Heterogeneity**† |  | ***n*** | **Ln ratio**‡ | ***P**** |
| *Major components* |  |  |  |  |  |  |  |  |  |
| Ash | 4 | 0.10 | -0.62, 0.83 | 0.778 | No (0%) |  | 6 | 4.60 | 0.357 |
| Casein | 7 | -0.56 | -1.95, 0.82 | 0.426 | Yes (88%) |  | 11 | 4.61 | 0.462 |
| Lactose | 17 | 0.00 | -0.42, 0.42 | 0.999 | Yes (80%) |  | 31 | 4.61 | 0.463 |
| Protein (whey) | - | - | - | - | - |  | 3 | 4.64 | 0.496 |
| α-lactalbumin | - | - | - | - | - |  | 3 | 4.57 | 0.252 |
| β-lactoglobulin | 3 | -0.09 | -0.77, 0.59 | 0.790 | No (0%) |  | 3 | 4.66 | 0.497 |
| *Fatty acids* |  |  |  |  |  |  |  |  |  |
| 4:0 (butyric acid) | 10 | 0.17 | -0.22, 0.56 | 0.399 | Yes (58%) |  | 15 | 4.62 | 0.255 |
| 6:0 (caproic acid) | 9 | -0.77 | -2.23, 0.68 | 0.296 | Yes (97%) |  | 14 | 4.60 | 0.434 |
| 10:0 (capric acid) | 10 | 0.74 | -1.74, 3.23 | 0.556 | Yes (99%) |  | 17 | 4.60 | 0.487 |
| 13:0 (tridecylic acid) | - | - | - | - | - |  | 3 | 4.57 | 0.499 |
| 18:0 (stearic acid) | 13 | -0.09 | -0.91, 0.72 | 0.825 | Yes (90%) |  | 20 | 4.58 | 0.254 |
| 12:0+14:0+16:0§ | - | - | - | - | - |  | 14 | 4.59 | 0.291 |
| USFA | 3 | 0.69 | -0.90, 2.28 | 0.396 | Yes (92%) |  | 3 | 4.61 | 0.503 |
| 18:1 | 4 | -11.96 | -38.16, 14.23 | 0.371 | Yes (100%) |  | 4 | 4.60 | 0.442 |
| 18:2 | 4 | -3.59 | -9.92, 2.74 | 0.266 | Yes (99%) |  | 6 | 4.40 | 0.145 |
| 18:3 | - | - | - | - | - |  | 3 | 4.46 | 0.381 |
| 10:1 (4-cis-decenoic acid) | 5 | -0.05 | -0.44, 0.34 | 0.805 | No (0%) |  | 5 | 4.47 | 0.198 |
| *n*, number of data points included in the comparison; SMD, standardised mean difference; USFA, unsaturated fatty acids. **P* value <0.05 indicates significance of the difference in composition between organic and conventional milk; †Heterogeneity and the I^2^ Statistic; ‡Ln ratio = Ln(ORG/CONV × 100%); §Calculated based on published fatty acids composition data. | | | | | | | | | |

| **Table S12 cont.** Results of the standard meta-analysis and sensitivity analysis 1 for parameters where none of the protocols identified significant effects. | | | | | | | | | |
| --- | --- | --- | --- | --- | --- | --- | --- | --- | --- |
|  | **Standard meta-analysis** | | | | |  | **Sensitivity analysis 1** | | |
| **Parameter** | ***n*** | **SMD** | **95% CI** | ***P**** | **Heterogeneity**† |  | ***n*** | **Ln ratio**‡ | ***P**** |
| 12:1 (lauroleic acid) | 3 | -0.36 | -1.17, 0.46 | 0.390 | No (0%) |  | 3 | 4.44 | 0.250 |
| 14:1 (myristoleic acid) | 7 | 0.15 | -0.44, 0.74 | 0.619 | Yes (65%) |  | 13 | 4.62 | 0.281 |
| 16:1 (palmitoleic acid) | 9 | -0.38 | -1.08, 0.32 | 0.292 | Yes (85%) |  | 17 | 4.58 | 0.172 |
| 17:1 (heptadecenoic acid) | 3 | 0.53 | -0.55, 1.60 | 0.336 | Yes (33%) |  | 4 | 4.80 | 0.439 |
| cis-11-18:1 (cis-vaccenic acid) | - | - | - | - | - |  | 5 | 4.54 | 0.281 |
| cis-12-18:1 | - | - | - | - | - |  | 3 | 4.77 | 0.494 |
| cis-13-18:1 | - | - | - | - | - |  | 3 | 4.79 | 0.491 |
| trans-9-18:1 (elaidic acid) | 3 | 0.24 | -1.52, 2.00 | 0.787 | Yes (97%) |  | 4 | 4.70 | 0.375 |
| trans-12-18:1 | 3 | -0.14 | -1.67, 1.40 | 0.862 | Yes (96%) |  | 3 | 4.79 | 0.507 |
| trans-6-8-18:1 | 3 | 0.00 | -1.34, 1.35 | 0.999 | Yes (94%) |  | 3 | 4.71 | 0.498 |
| CLA (trans-7,9-18:2) | - | - | - | - | - |  | 3 | 5.07 | 0.499 |
| CLA (trans-9,11-18:2) | - | - | - | - | - |  | 3 | 5.34 | 0.123 |
| CLA (trans-11,13-18:2) | - | - | - | - | - |  | 3 | 5.61 | 0.125 |
| CLA (trans-12,14-18:2) | - | - | - | - | - |  | 3 | 5.55 | 0.121 |
| cis-11,14-20:2 | - | - | - | - | - |  | 3 | 4.74 | 0.506 |
| ETE (cis-11,14,17-20:3) | - | - | - | - | - |  | 4 | 4.70 | 0.495 |
| Long chain FA | 5 | 0.07 | -1.18, 1.32 | 0.917 | Yes (88%) |  | 6 | 4.63 | 0.188 |
| Medium chain FA | 5 | 0.10 | -0.25, 0.45 | 0.567 | No (0%) |  | 7 | 4.57 | 0.205 |
| Short chain FA | 5 | 0.31 | -1.43, 2.04 | 0.728 | Yes (93%) |  | 6 | 4.61 | 0.463 |
| *n*, number of data points included in the comparison; SMD, standardised mean difference; CLA, conjugated linoleic acids; ETE, eicosatrienoic acid; FA, fatty acids. **P* value <0.05 indicates significance of the difference in composition between organic and conventional milk; †Heterogeneity and the I^2^ Statistic; ‡Ln ratio = Ln(ORG/CONV × 100%). | | | | | | | | | |

| **Table S12 cont.** Results of the standard meta-analysis and sensitivity analysis 1 for parameters where none of the protocols identified significant effects. | | | | | | | | | |
| --- | --- | --- | --- | --- | --- | --- | --- | --- | --- |
|  | **Standard meta-analysis** | | | | |  | **Sensitivity analysis 1** | | |
| **Parameter** | ***n*** | **SMD** | **95% CI** | ***P**** | **Heterogeneity**† |  | ***n*** | **Ln ratio**‡ | ***P**** |
| *Vitamins and antioxidants* |  |  |  |  |  |  |  |  |  |
| Vitamin C | - | - | - | - | - |  | 3 | 4.84 | 0.131 |
| Vitamin D | 3 | 0.14 | -1.00, 1.28 | 0.805 | Yes (56%) |  | 3 | 4.52 | 0.369 |
| Vitamin E activity | - | - | - | - | - |  | 4 | 4.82 | 0.061 |
| *Minerals and undesirable metals* |  |  |  |  |  |  |  |  |  |
| Cadmium (Cd) | 4 | -0.29 | -0.73, 0.16 | 0.204 | No (2%) |  | 8 | 4.62 | 0.476 |
| Calcium (Ca) | 7 | -0.12 | -0.47, 0.23 | 0.512 | No (0%) |  | 12 | 4.62 | 0.217 |
| Cobalt (Co) | 3 | 0.01 | -0.50, 0.51 | 0.983 | No (0%) |  | 3 | 4.45 | 0.254 |
| Lead (Pb) | 4 | -0.21 | -0.65, 0.23 | 0.348 | No (0%) |  | 7 | 4.58 | 0.327 |
| Magnesium (Mg) | 6 | -64.62 | -194.47, 65.24 | 0.329 | Yes (100%) |  | 9 | 4.58 | 0.131 |
| Manganese (Mn) | 4 | -0.44 | -1.10, 0.22 | 0.188 | Yes (45%) |  | 4 | 4.50 | 0.244 |
| Molybdenum (Mo) | 3 | 0.51 | -0.18, 1.21 | 0.147 | Yes (54%) |  | 3 | 4.74 | 0.123 |
| Phosphorus (P) | 5 | 0.00 | -0.30, 0.30 | 0.997 | No (0%) |  | 9 | 4.60 | 0.315 |
| Sodium (Na) | 3 | -0.15 | -0.69, 0.38 | 0.571 | No (0%) |  | 5 | 4.59 | 0.159 |
| Zinc (Zn) | 9 | -0.21 | -0.49, 0.08 | 0.155 | No (9%) |  | 12 | 4.56 | 0.059 |
| *n*, number of data points included in the comparison; SMD, standardised mean difference. **P* value <0.05 indicates significance of the difference in composition between organic and conventional milk; †Heterogeneity and the I^2^ Statistic; ‡Ln ratio = Ln(ORG/CONV × 100%). | | | | | | | | | |

| **Table S12 cont.** Results of the standard meta-analysis and sensitivity analysis 1 for parameters where none of the protocols identified significant effects. | | | | | | | | | |
| --- | --- | --- | --- | --- | --- | --- | --- | --- | --- |
|  | **Standard meta-analysis** | | | | |  | **Sensitivity analysis 1** | | |
| **Parameter** | ***n*** | **SMD** | **95% CI** | ***P**** | **Heterogeneity**† |  | ***n*** | **Ln ratio**‡ | ***P**** |
| *Pesticides, mycotoxins and other contaminants* |  |  |  |  |  |  |  |  |  |
| Aflatoxin M1 | - | - | - | - | - |  | 5 | 4.79 | 0.191 |
| Dieldrin | - | - | - | - | - |  | 3 | 3.98 | 0.246 |
| Hexachlorobenzene (HCB) | - | - | - | - | - |  | 5 | 4.75 | 0.255 |
| α-esachlorciclohexane (α-HCH) | - | - | - | - | - |  | 3 | 4.48 | 0.379 |
| γ-esachlorciclohexane (γ-HCH) | - | - | - | - | - |  | 4 | 4.05 | 0.252 |
| *Other* |  |  |  |  |  |  |  |  |  |
| Atherogenicity Index | - | - | - | - | - |  | 3 | 4.41 | 0.126 |
| Bacteria count | 8 | -0.05 | -0.29, 0.19 | 0.682 | Yes (35%) |  | 12 | 4.59 | 0.458 |
| Dry mass | - | - | - | - | - |  | 5 | 4.58 | 0.184 |
| Lactoferrin | 3 | 4.20 | -3.13, 11.53 | 0.261 | Yes (98%) |  | 3 | 4.80 | 0.256 |
| Lysozyme | 3 | 1.08 | -3.04, 5.19 | 0.608 | Yes (96%) |  | 3 | 4.71 | 0.506 |
| pH | 5 | 0.34 | -0.36, 1.04 | 0.346 | No (18%) |  | 7 | 4.61 | 0.500 |
| Thrombogenicity index | - | - | - | - | - |  | 3 | 4.43 | 0.125 |
| Titratable acidity | 3 | 0.79 | -0.14, 1.73 | 0.096 | No (0%) |  | 4 | 4.71 | 0.065 |
| *n*, number of data points included in the comparison; SMD, standardised mean difference. **P* value <0.05 indicates significance of the difference in composition between organic and conventional milk; †Heterogeneity and the I^2^ Statistic; ‡Ln ratio = Ln(ORG/CONV × 100%). | | | | | | | | | |

| **Table S13.** Results of the statistical test for publication bias reported in Table 1 of the main paper. | | | | | |
| --- | --- | --- | --- | --- | --- |
|  | **Trim and fill test*** | | **No of missing *n* in Rosenthal’s Fail-safe N test**† | **No of missing *n* in Orwin’s Fail-safe N test**‡ | ***P* from Egger’s test for  funnel plot asymetry**§ |
| **Parameter** | **No of missing *n*** | **funnel plot side** |  |  |  |
| Milk yield | 0 | right | 5697 | 32 | 0.253 |
| SFA | 2 | left | 0 | 19 | 0.003 |
| 12:0 (lauric acid) | 0 | left | 0 | 11 | 0.039 |
| 14:0 (myristic acid)\|\| | 0 | left | 96 | 12 | <0.001 |
| 16:0 (palmitic acid) | 1 | left | 0 | 14 | <0.001 |
| MUFA | 2 | right | 0 | 19 | 0.003 |
| OA (cis-9-18:1) | 0 | left | 0 | 10 | 0.012 |
| VA (trans-11-18:1) | - | - | 514 | 12 | <0.001 |
| PUFA | 0 | left | 211 | 19 | 0.118 |
| CLA (total) | 0 | left | 146 | 11 | 0.003 |
| CLA9 (cis-9-trans-11-18:2) | 0 | left | 416 | 14 | 0.002 |
| CLA10 (trans-10-cis-12-18:2) | 0 | left | 8 | 3 | 0.028 |
| n-3 FA | 0 | left | 492 | 12 | <0.001 |
| ALA (cis-9,12,15-18:3) | 0 | left | 3146 | 21 | <0.001 |
| EPA (cis-5,8,11,14,17-20:5) | 3 | left | 291 | 8 | 0.403 |
| DPA (cis-7,10,13,16,19-22:5) | 0 | left | 89 | 5 | 0.005 |
| DHA (cis-4,7,10,13,16,19-22:6) | 0 | left | 0 | 3 | 0.228 |
| VLC n-3 PUFA¶ | - | - | - | - | - |
| n-6 FA | 0 | left | 0 | 12 | 0.043 |
| LA (cis-9,12-18:2) | 3 | right | 233 | 12 | 0.956 |
| AA (cis-5,8,11,14-20:4) | 2 | right | 36 | 5 | 0.002 |
| LA/ALA ratio¶ | - | - | - | - | - |
| n-6/n-3 ratio | 0 | right | 138 | 7 | 0.002 |
| n-3/n-6 ratio | 0 | left | 94 | 5 | 0.002 |
| SFA, saturated fatty acids; MUFA, monounsaturated fatty acids; OA, oleic acid; VA, vaccenic acid; PUFA, polyunsaturated fatty acids; CLA, conjugated linoleic acid; FA, fatty acids; ALA, α-linolenic acid; EPA, eicosapentaenoic acid; DPA, docosapentaenoic acid; DHA, docosahexaenoic acid; VLC n-3 PUFA, very long chain n-3 PUFA (EPA+DPA+DHA); LA, linoleic acid; AA, arachidonic acid. *The method used to estimate the number of data points missing from a meta-analysis due to the suppression of the most extreme results on one side of the funnel plot; †Number of missing data points that need to be retrieved and incorporate in the meta-analysis before the results become nonsignificant; ‡Number of missing data point that need to be retrieved and incorporate in the meta-analysis before the estimated value of the standardised mean (SMD) difference reaches a specified level (here SMD/2); §*P* value <0.05 indicates funnel plot asymmetry; \|\|Outlying data pairs (where the MPD between ORG and CONV was over fifty times greater than the mean value including the outliers) were removed; ¶Calculated based on published fatty acids composition data. | | | | | |

| **Table S13 cont.** Results of the statistical test for publication bias reported in Table 1 of the main paper. | | | | | |
| --- | --- | --- | --- | --- | --- |
|  | **Trim and fill test*** | | **No of missing *n* in Rosenthal’s Fail-safe N test**† | **No of missing *n* in Orwin’s Fail-safe N test**‡ | ***P* from Egger’s test for  funnel plot asymetry**§ |
| **Parameter** | **No of missing *n*** | **funnel plot side** |  |  |  |
| α-tocopherol | 0 | left | 42 | 9 | 0.001 |
| Carotenoids | 0 | left | 8 | 5 | 0.485 |
| β-carotene | 0 | right | 0 | 7 | 0.970 |
| Lutein | 0 | left | 3 | 3 | 0.390 |
| Zeaxanthin | - | - | - | - | - |
| Iodine (I) | 0 | right | 101 | 6 | 0.815 |
| Iron (Fe) | 0 | right | 3 | 8 | 0.641 |
| Selenium (Se) | 1 | left | 4 | 4 | 0.857 |
| Urea | 0 | right | 6 | 7 | 0.192 |
| SCC\|\| | 9 | right | 122 | 20 | 0.084 |
| SCC, somatic cell count. *The method used to estimate the number of data points missing from a meta-analysis due to the suppression of the most extreme results on one side of the funnel plot; †Number of missing data points that need to be retrieved and incorporate in the meta-analysis before the results become nonsignificant; ‡Number of missing data point that need to be retrieved and incorporate in the meta-analysis before the estimated value of the standardised mean (SMD) difference reaches a specified level (here SMD/2); §*P* value <0.05 indicates funnel plot asymmetry; \|\|Outlying data pairs (where the MPD between ORG and CONV was over fifty times greater than the mean value including the outliers) were removed; ¶Calculated based on published fatty acids composition data. | | | | | |


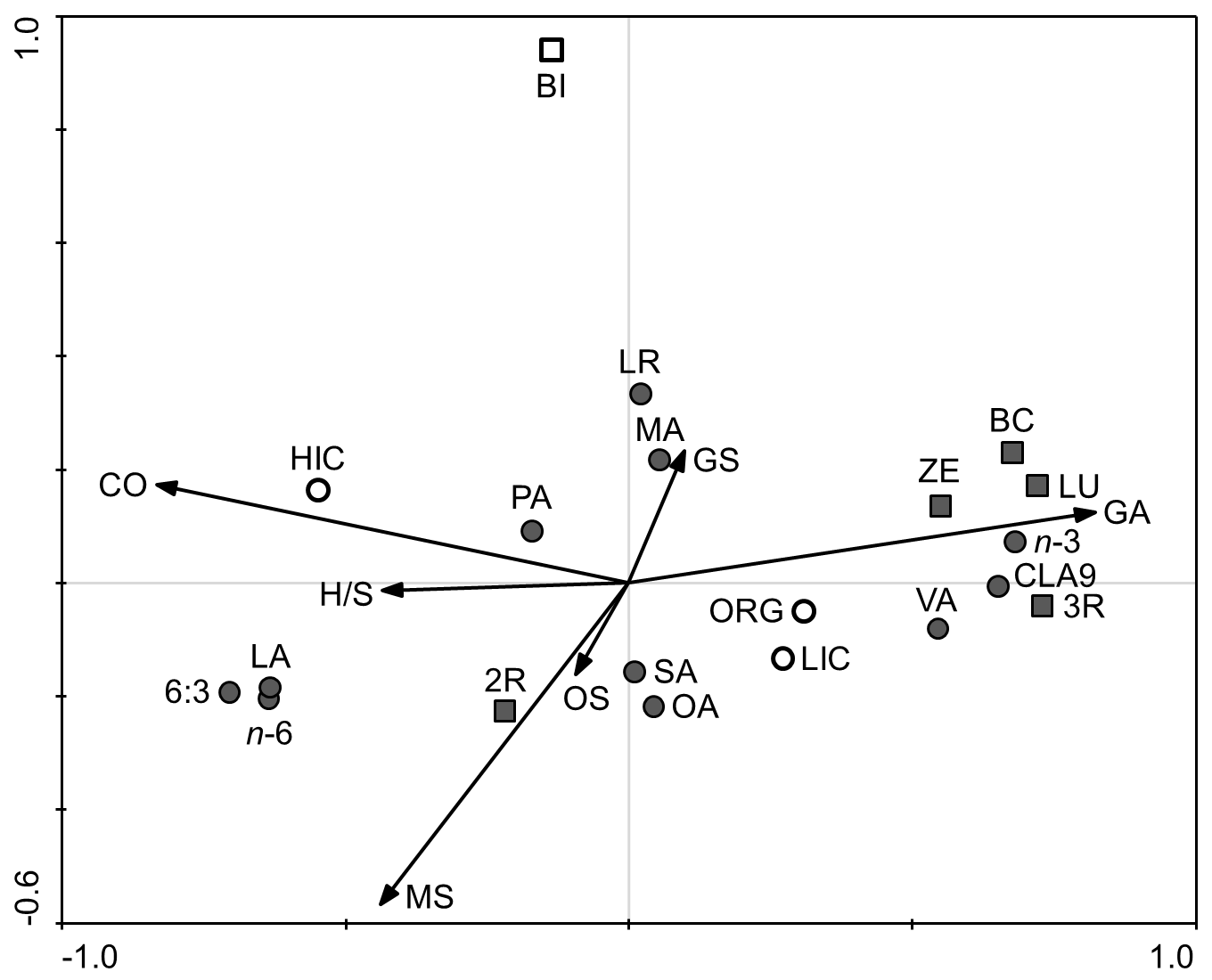


**Figure S34**. Bi-plot derived from the redundancy analysis showing the relationship between milk composition parameters (fatty acids (●) and antioxidants (■)) and cows feeding and rearing parameters (categorical explanatory variables (○,□)) and quantitative explanatory variables (🡲). 6:3, *n*-3/*n*-6 fatty acids ratio; 2R, synthetic isomers of α-tocopherol; 3R, natural isomers of α-tocopherol; BC, β-carotene; BI, breed index; CLA9, rumenic acid (*cis*-9,*trans*-11-18:2); CO, concentrate feeds; GA, grazing intake; GS, grass silage; HIC, high-input conventional production system; H/S, hay or straw; LA, linoleic acid (*cis*-9,12-18:2); LIC, low-input conventional production system; LU, lutein; LR, lauristic acid (12:0); MA, myristic acid (14:0); MS, maize silage; *n*-3, omega-3 fatty acids; *n*-6, omega-6 fatty acids; OA, oleic acid (*cis*-9-18:1); ORG, organic production system; OS, other silage; PA, palmitic acid (16:0); SA, stearic acid (18:0); VA, vaccenic acid (trans-11-18:1); ZE, zeaxanthin.


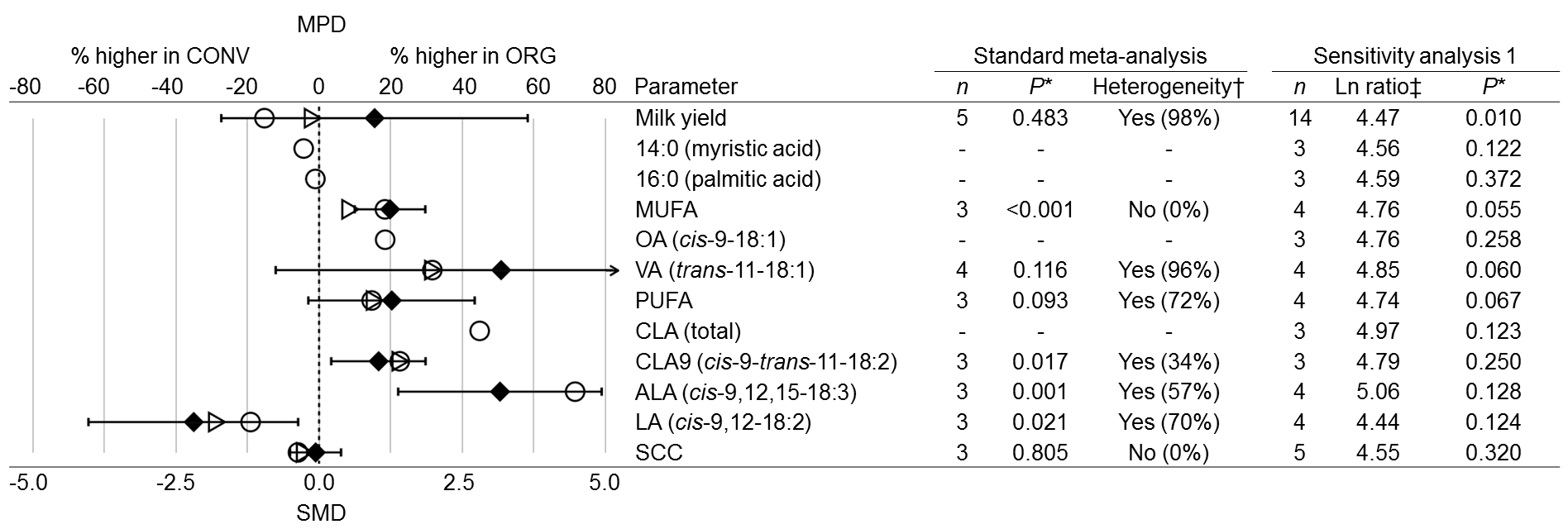


**Figure S35**. Results of standard meta-analysis and sensitivity analysis 1 for milk yield, fat composition and somatic cells in goat, sheep and buffalo milk. MPD, mean percent difference; CONV, conventional samples; ORG, organic samples; *n*, number of datapoints included in meta-analysis; MUFA, monounsaturated fatty acids; OA, oleic acid; VA, trans-vaccenic acid; PUFA, polyunsaturated fatty acids; CLA, conjugated linoleic acid; ALA, α-linolenic acid; LA, linoleic acid; SCC, somatic cell count; SMD, standardised mean difference. **P* value <0.05 indicates a significant difference between ORG and CONV; †Heterogeneity and the I^2^ Statistic; ‡Ln ratio = Ln(ORG/CONV × 100%); ○, MPD calculated using data included in sensitivity analysis 1;
▷, MPD calculated using data included in standard meta-analysis; ◆, SMD from the standard meta-analysis with 95% confidence intervals represented by horizontal bars.

| **Table S14.** Mean percentage differences (MPD) for individual studies (study ID in parentheses, see Table S1 for references) calculated using the data for goat, sheep and buffalo milk and cheese of composition parameters shown in Fig. 2 and 3 of the main paper. | | | | |
| --- | --- | --- | --- | --- |
| **Parameter** | **goat milk** | **sheep milk** | **buffalo milk** | **buffalo cheese** |
| SFA | -2^NS^ | -9* |  |  |
|  | (386) | (456) |  |  |
| 12:0 (lauric acid) | -3^NS^ | -20^NS^ |  |  |
|  | (386) | (456) |  |  |
| 14:0 (myristic acid) |  |  |  | -6^NS^ |
|  |  |  |  | (155) |
| 16:0 (palmitic acid) |  |  |  | 1^NS^ |
|  |  |  |  | (155) |
| OA (cis-9-18:1) |  |  |  | -7* |
|  |  |  |  | (155) |
| VA (trans-11-18:1) |  |  |  | 105* |
|  |  |  |  | (155) |
| CLA (total) |  |  |  | 45** |
|  |  |  |  | (155) |
| n-3 FA | 200* | 71* |  |  |
|  | (385) | (385) |  |  |
| ALA (cis-9,12,15-18:3) |  |  |  | 40.63* |
|  |  |  |  | (155) |
| n-6 FA | -22^NS^ | 3^NS^ |  |  |
|  | (385) | (385) |  |  |
| LA (cis-9,12-18:2) |  |  |  | -48* |
|  |  |  |  | (155) |
| n-6/n-3 ratio | -5.36^NS^ | -53^NS^ |  |  |
|  | (385) | (385) |  |  |
| n-3/n-6 ratio | 267^NS^ | 67* |  |  |
|  | (385) | (385) |  |  |
| Atherogenicity Index | -18* | -18* |  |  |
|  | (385) | (385) |  |  |
| α-tocopherol |  |  | 52* | 44* |
|  |  |  | (155) | (155) |
| Iron (Fe) |  | -33^NS^ |  |  |
|  |  | (456) |  |  |
| Urea |  | 5^NS^ | 10^NR^ |  |
|  |  | (616) | (474) |  |
| SFA, saturated fatty acids; OA, oleic acid; VA, vaccenic acid; CLA, conjugated linoleic acid; FA, fatty acids; ALA, α-linolenic acid; . *Indicates significant difference between organic (ORG) and conventional (CONV) samples reported by the author when *P*≤0.05; **Indicates significant difference between ORG and CONV samples reported by the author when *P*≤0.01; ^NS^Indicates that no significant difference between ORG and CONV samples were detected by the author; ^NR^Indicates that the author did not reported significance of difference between ORG and CONV samples. | | | | |

| **Table S15.** Mean percentage differences (MPD) for individual studies (study ID in parentheses, see Table S1 for references) calculated using the data for bovine dairy products of composition parameters shown in Fig. 2 and 3 of the main paper. | | | | | | | |
| --- | --- | --- | --- | --- | --- | --- | --- |
| **Parameter** | **fermented milk** | **yoghurt** | **cheese** | **curd** | **butter** | **milk+cheese +butter** | **desalted milk, whey** |
| SFA | 1^NR^ | -5* | -2* | 1^NS^ |  |  |  |
|  | (455) | (455) | (190) | (190) |  |  |  |
|  | -1^NR^ | -2^NR^ | -7^NR^ |  |  |  |  |
|  | (591) | (591) | (406) |  |  |  |  |
|  | -6^NR^ |  | 7^NS^ |  |  |  |  |
|  | (595) |  | (556) |  |  |  |  |
|  | -1^NR^ |  |  |  |  |  |  |
|  | (705) |  |  |  |  |  |  |
| 12:0 (lauric acid) |  |  | 18^NS^ |  |  |  |  |
|  |  |  | (556) |  |  |  |  |
| 14:0 (myristic acid) |  |  | 13^NS^ |  | -1^NS^ |  |  |
|  |  |  | (556) |  | (666) |  |  |
| 16:0 (palmitic acid) | -14^NR^ |  | 6^NS^ |  | -1^NS^ |  |  |
|  | (595) |  | (556) |  | (666) |  |  |
| MUFA | 1^NR^ | -8* | 13^NR^ | -1^NS^ |  |  |  |
|  | (455) | (455) | (406) | (190) |  |  |  |
|  | 1^NR^ | 2^NR^ | -11^NS^ |  |  |  |  |
|  | (591) | (591) | (556) |  |  |  |  |
|  | 15^NR^ |  |  |  |  |  |  |
|  | (595) |  |  |  |  |  |  |
|  | 2^NR^ |  |  |  |  |  |  |
|  | (705) |  |  |  |  |  |  |
| OA (cis-9-18:1) |  |  | -12^NS^ |  |  |  |  |
|  |  |  | (556) |  |  |  |  |
| VA (trans-11-18:1) | 50^NR^ | 54^NR^ | 41^NR^ | 44^NS^ | 42^NR^ |  |  |
|  | (591) | (591) | (155) | (190) | (155) |  |  |
|  | 73^NR^ |  | 60* |  | 27** |  |  |
|  | (595) |  | (190) |  | (666) |  |  |
|  | 73^NR^ |  | 46^NR^ |  |  |  |  |
|  | (705) |  | (406) |  |  |  |  |
| SFA, saturated fatty acids; MUFA, monounsaturated fatty acids; OA, oleic acid; VA, vaccenic acid; PUFA, polyunsaturated fatty acids. *Indicates significant difference between organic (ORG) and conventional (CONV) samples reported by the author when *P*≤0.05; **Indicates significant difference between ORG and CONV samples reported by the author when *P*≤0.01; ^NS^Indicates that no significant difference between ORG and CONV samples were detected by the author; ^NR^Indicates that the author did not reported significance of difference between ORG and CONV samples. | | | | | | | |

| **Table S15 cont.** Mean percentage differences (MPD) for individual studies (study ID in parentheses, see Table S1 for references) calculated using the data for bovine dairy products of composition parameters shown in Fig. 2 and 3 of the main paper. | | | | | | | |
| --- | --- | --- | --- | --- | --- | --- | --- |
| **Parameter** | **fermented milk** | **yoghurt** | **cheese** | **curd** | **butter** | **milk+cheese +butter** | **desalted milk, whey** |
| PUFA | 4^NR^ | -9^NS^ | 4^NS^ | -2^NS^ |  |  |  |
|  | (455) | (455) | (190) | (190) |  |  |  |
|  | 33^NR^ | 40^NR^ | 27^NR^ |  |  |  |  |
|  | (591) | (591) | (406) |  |  |  |  |
|  | -3^NR^ |  | -9^NS^ |  |  |  |  |
|  | (595) |  | (556) |  |  |  |  |
|  | 1^NR^ |  |  |  |  |  |  |
|  | (705) |  |  |  |  |  |  |
| CLA (total) | 129^NR^ |  | 61^NR^ |  | 72^NR^ |  |  |
|  | (595) |  | (155) |  | (155) |  |  |
|  | 4^NR^ |  | 53^NR^ |  |  |  |  |
|  | (705) |  | (406) |  |  |  |  |
|  |  |  | 38^NR^ |  |  |  |  |
|  |  |  | (674) |  |  |  |  |
| CLA9 |  |  | 56* | 33^NS^ | 96** |  |  |
| (cis-9-trans-11-18:2) |  |  | (190) | (190) | (666) |  |  |
|  |  |  | -18^NS^ |  |  |  |  |
|  |  |  | (556) |  |  |  |  |
| n-3 FA |  |  | 28^NR^ |  |  |  |  |
|  |  |  | (406) |  |  |  |  |
| ALA | 101^NR^ | 114^NR^ | 51^NR^ | 51^NS^ | 91^NR^ |  |  |
| (cis-9,12,15-18:3) | (591) | (591) | (155) | (190) | (155) |  |  |
|  | 21^NR^ |  | 68* |  | 68* |  |  |
|  | (595) |  | (190) |  | (666) |  |  |
|  | 111^NR^ |  | 18^NS^ |  |  |  |  |
|  | (705) |  | (556) |  |  |  |  |
| n-6 FA |  |  | 18^NR^ |  |  |  |  |
|  |  |  | (406) |  |  |  |  |
| LA (cis-9,12-18:2) |  |  | -40^NR^ | -25^NS^ | -37^NR^ |  |  |
|  |  |  | (155) | (190) | (155) |  |  |
|  |  |  | -19* |  | -37* |  |  |
|  |  |  | (190) |  | (666) |  |  |
|  |  |  | -3^NS^ |  |  |  |  |
|  |  |  | (556) |  |  |  |  |
| CLA, conjugated linoleic acid; FA, fatty acids; ALA, α-linolenic acid; LA, linoleic acid. *Indicates significant difference between organic (ORG) and conventional (CONV) samples reported by the author when *P*≤0.05; **Indicates significant difference between ORG and CONV samples reported by the author when *P*≤0.01; ^NS^Indicates that no significant difference between ORG and CONV samples were detected by the author; ^NR^Indicates that the author did not reported significance of difference between ORG and CONV samples. | | | | | | | |

| **Table S15 cont.** Mean percentage differences (MPD) for individual studies (study ID in parentheses, see Table S1 for references) calculated using the data for bovine dairy products of composition parameters shown in Fig. 2 and 3 of the main paper. | | | | | | | |
| --- | --- | --- | --- | --- | --- | --- | --- |
| **Parameter** | **fermented milk** | **yoghurt** | **cheese** | **curd** | **butter** | **milk+cheese +butter** | **desalted milk, whey** |
| n-6/n-3 ratio |  |  | -27^NR^ |  |  |  |  |
|  |  |  | (406) |  |  |  |  |
|  |  |  | -20^NS^ |  |  |  |  |
|  |  |  | (556) |  |  |  |  |
| n-3/n-6 ratio |  |  | 27^NR^ |  |  |  |  |
|  |  |  | (406) |  |  |  |  |
|  |  |  | 20^NR^ |  |  |  |  |
|  |  |  | (556) |  |  |  |  |
| α-tocopherol |  |  | 33^NR^ |  |  | 49* |  |
|  |  |  | (406) |  |  | (155) |  |
| β-carotene |  |  |  |  |  | 101* |  |
|  |  |  |  |  |  | (155) |  |
| Iron (Fe) |  |  | 12^NR^ |  |  |  |  |
|  |  |  | (406) |  |  |  |  |
| Selenium (Se) |  |  |  |  |  |  | -59* |
|  |  |  |  |  |  |  | (669) |
|  |  |  |  |  |  |  | -36*** |
|  |  |  |  |  |  |  | (669) |
| CLA, conjugated linoleic acid; FA, fatty acids; ALA, α-linolenic acid; LA, linoleic acid. *Indicates significant difference between organic (ORG) and conventional (CONV) samples reported by the author when *P*≤0.05; **Indicates significant difference between ORG and CONV samples reported by the author when *P*≤0.01; ^NS^Indicates that no significant difference between ORG and CONV samples were detected by the author; ^NR^Indicates that the author did not reported significance of difference between ORG and CONV samples. | | | | | | | |

# 3. ADDITIONAL DISCUSSION

Three previous systematic literature reviews^(^[^1-3^](#_ENREF_1)^)^ used meta-analyses methods to synthesise published information on composition differences between organic and conventional milk and/or dairy products, but report contrasting results and conclusions. The main results these studies are described and discussed below.

Dangour *et al*.^(^[^1^](#_ENREF_1)^)^ combined data for milk, meat and eggs extracted from 25 publications (11 deemed to be of satisfactory quality) and carried out unweighted meta-analyses (T-test with “robust standard deviation”). For livestock products their published paper only reports results for total fat and ash contents which were not significantly different. However, in their report to the sponsor of their study (UK Food Standard Agency)^(^[^4^](#_ENREF_4)^)^ meta-analyses results for total SFA, MUFA, PUFA, *n*-3 PUFA and *n*-6 PUFA were also reported. For most of these parameters no significant difference between organic and conventional livestock products was found in meta-analysis using all available data or only data from studies the authors deemed to be satisfactory. However, significantly higher concentration (*P*=0.001; *n*=12; MPD=10) and a trend towards higher concentrations (*P*=0.07; *n*=5; MPD=11) of total PUFA in organic livestock products were detected when all available data or only data from studies the authors deemed satisfactory, were used in meta-analyses respectively. Also, a trend towards higher *n*-3 PUFA concentrations (*P*=0.10; *n*=13; MPD=67) was detected when only data from studies the authors deemed satisfactory were used in meta-analyses. The Dangour *et al*.^(^[^1^](#_ENREF_1)^)^ study concluded: *“On the basis of a systematic review of studies of satisfactory quality, there is no evidence for difference in nutrient quality between organic and conventionally produced foodstuffs”*.

Palupi *et al*.^(^[^2^](#_ENREF_2)^)^ extracted data from 14 studies published between March 2008 and April 2011 reporting data for bovine milk (13 studies) and bovine dairy products (1 study) and carried out weighted meta-analyses. Results showed significantly higher (*P*<0.001) concentrations of fat, protein, SFA, PUFA, *n*-3 PUFA, ALA, EPA, DPA, CLA, vaccenic acid (VA), α-tocopherol and β-carotene, but lower concentrations of MUFA, stearic acid (18:0), oleic acid (18:1), *n*-6 PUFA and LA in organic compared to non-organic milk/dairy products. Palupi *et al*.^(^[^2^](#_ENREF_2)^)^ concluded that *“current regulation on dairy farming indeed enables the driving of organic farming to produce organic dairy products with different nutritional quality from conventional products”*.

Smith-Spangler *et al*.^(^[^3^](#_ENREF_3)^)^ extracted data from 37 studies reporting data for milk (30 on raw and 7 on pasteurised milk) and carried out weighted meta-analysis. Their published paper reported results for only 2 parameters, with significantly higher concentrations of *n*-3 PUFA (*P*<000.1; *n*=5) and VA (*P*<0.031; *n*=5) found in organic milk. Meta-analysis results for other milk quality parameters are said to be available as Supplement 6 on a website ([*www.annals.org*](http://www.annals.org)) but could not be obtained from either the website or the authors. Despite showing organic milk has significantly higher *n*-3 PUFA concentrations, which Smith-Spangler *et al*.^(^[^3^](#_ENREF_3)^)^ describe as “*beneficial*” they conclude: *“The published literature lacks strong evidence that organic foods are significantly more nutritious than conventional foods.”* and describe *“Studies were heterogeneous and limited in number, and publication bias may be present”* as a main limitation of their study.

# 4. ADDITIONAL REFERENCES

1. Dangour AD, Dodhia SK, Hayter A *et al.* (2009) Nutritional quality of organic foods: a systematic review. *Am J Clin Nutr* **90**, 680-685.

2. Palupi E, Jayanegara A, Ploeger A *et al.* (2012) Comparison of nutritional quality between conventional and organic dairy products: a meta-analysis. *J Sci Food Agric* **92**, 2774-2781.

3. Smith-Spangler C, Brandeau ML, Hunter GE *et al.* (2012) Are Organic Foods Safer or Healthier Than Conventional Alternatives?A Systematic Review. *Ann Intern Med* **157**, 348-366.

4. Dangour AD, Dodhia S, Hayter A *et al.* (2009) *Comparison of composition (nutrients and other substances) of organically and conventionally produced foodstuffs: a systematic review of the available literature*. *Report for the Food Standards Agency*. Nutrition and Public Health Intervention Research Unit London School of Hygiene & Tropical Medicine.
